# Harnessing the promiscuity of a nonulosonic acid synthase for the chemoenzymatic synthesis of pseudaminic acid derivatives and specificity mapping of an anti-Pse antibody

**DOI:** 10.1039/d6cb00217j

**Published:** 2026-09-07

**Authors:** Natasha E. Hatton, Niccolay Madiedo Soler, Ethan D. Goddard-Borger, Martin A. Fascione

**Affiliations:** a Department of Chemistry, University of York York YO10 5DD UK martin.fascione@york.ac.uk; b The Walter and Eliza Hall Institute of Medical Research Parkville Victoria 3052 Australia; c Department of Medical Biology, University of Melbourne Parkville Victoria 3010 Australia

## Abstract

Herein we report a divergent route from a single diazido precursor to four 6-deoxy-l-altdiamides, converted on preparative scale by promiscuous *C. jejuni* PseI into pseudaminic acid derivatives Pse5Ac7Ac, Pse5Ac7Fm, Pse5Ac7Hb and Pse5Hb7Fm. Surface plasmon resonance revealed the 1E8 monoclonal antibody tolerates varied C7 amides but not a C5 3-hydroxybutyryl group.

## Abstract 2

Chemoenzymatic synthesis of pseudaminic acid derivatives using a synthase enzyme enables epitope specificity mapping of an anti-Pse antibody.

## Graphical abstract

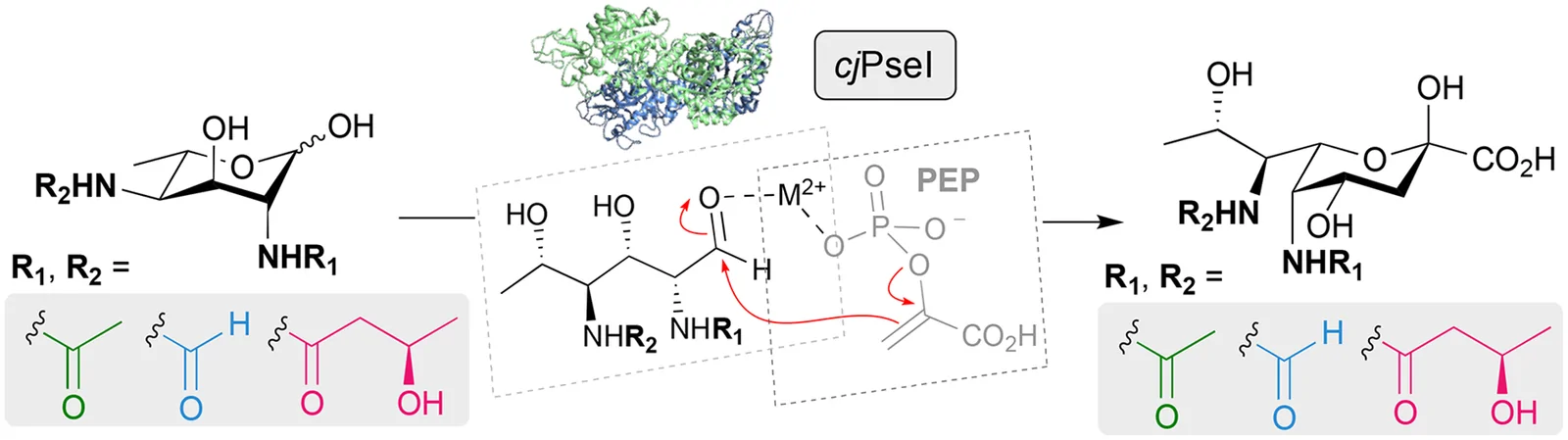

Nonulosonic acids^1,2^ are among the most abundant and important sugars on Earth, epitomised by the widely studied Neu5Ac 1 (Fig. 1a), which is essential for host cell recognition by the human immune system.^3^ However, this ‘sialic acid’, as it is also known, only represents one member of this broader nonulosonic acid family.^4^ In fact at least 20% of all microbial phylotypes encode genes for the biosynthesis of these acidic 9-carbon sugars,^5^ many likely still uncharacterised, constituting a potential uncharted ‘dark matter’ of the glycome. To explore this chemical and biological space it is therefore essential we develop new approaches to discover and sythesise these sugars.

**Fig. 1:**
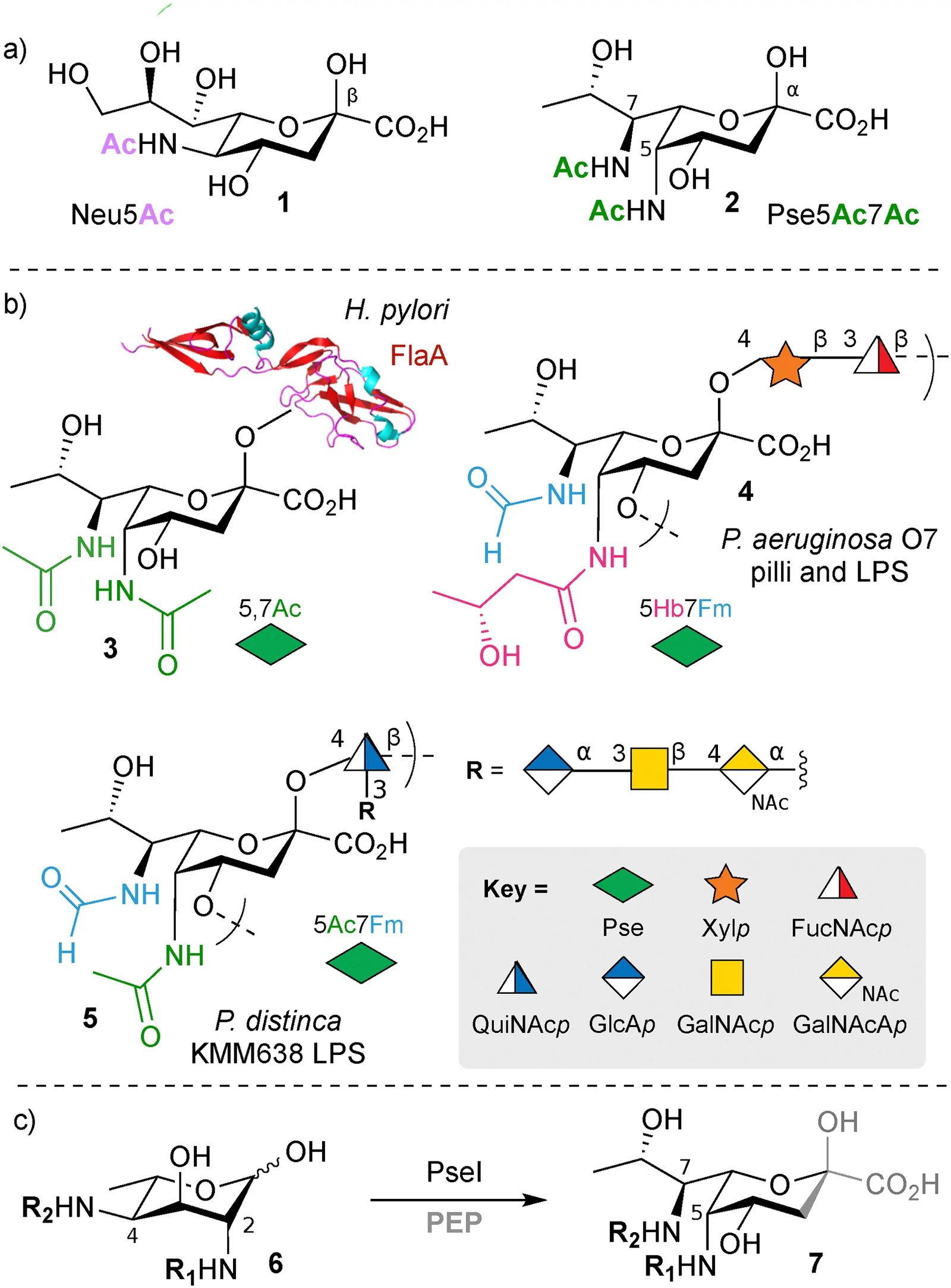
(a) A structural comparison of Neu5Ac 1 and Pse5Ac7Ac 2. (b) Examples of Pse bacterial glycoconjugates showcasing differential substitution at C5 and C7. (c) Conversion of 6-deoxy-*alt*-2,4-diamides 6 into Pse derivatives 7 by pseudaminic acid synthase enzymes (PseI) in the final step of the Pse biosynthesis.

Pseudaminic acids (Pse), such as Pse5Ac7Ac 2 (Fig. 1a),^6,7^ are among the most widespread microbial versions of this class, playing important roles in the virulence and immunoevasion^8,9^ of numerous human bacterial pathogens which they adorn.^4^ Although ostensibly similar to Neu5Ac 1, the Pse backbone contains two amines which can be modified with different functionality (Fig. 1b) including acetyl (Ac), formyl (Fm) and 3-hydroxybutyryl (Hb) amides.^10^ Evidence suggests these different varieties may tune the biological activity of the sugar and can be found in different contexts. For instance, sheathed flagella of gut pathogen *H. pylori* are only glycosylated with Pse5Ac7Ac 3,^11,12^ which is essential for motility, whilst *C. jejuni* flagella proteins can also be modified with different Pse derivatives^13–15^ which have seemingly evolved to facilitate evasion of the human immune system through interaction with host Siglec proteins.^9^ Similarly, pathogenic *P. aeruginosa* strains decorate their pili and lipopolysaccharide (LPS) with the Pse5Hb7Fm derivative 4,^16^ which is essential for virulence, whilst the LPS of marine symbiont *Pseudoalteromonas distincta* KMM638 contains Pse5Ac7Fm 5 instead.^17^ Such diversity typifies the complex microbial presentation of Pse and has catalysed a drive to develop new tools to aid detection and dissection of the ‘Pseome’. Recent progress in this area is exemplified by the generation of anti-Pse monoclonal antibodies,^18^ which have furthered our understanding of bacterial pseudaminylation. However our ability to validate and develop such tools is currently limited by the challenge of easily accessing differentially modifed versions of the Pse sugar backbone, despite previous tremendous work in Pse chemical synthesis.^2,19–26^ Herein we address this challenge using chemoenzymatic synthesis and validate that a single late stage precursor can be used as a branch point to efficiently access differentially modified Pse derivatives, notably harnessing the promiscuity of a wild type pseudaminic acid synthase enzyme (PseI).^27,28^ We then utilise these derivatives to map the epitope specificity of a Pse monoclonal antibody.

PseI performs the final enzymatic step in Pse biosynthesis (Fig. 1c), catalysing the aldol condensation of the ring-opened aldehyde form of a 6-deoxy-altdiamide 6 with phosphoenolpyruvate (PEP) to construct the 9-carbon backbone before cyclisation to the ring-closed form of Pse 7, an approach we had previously deployed^28–30^*in vitro* to access Pse5Ac7Ac 2 but as yet no other derivatives. Therefore in order to explore the substrate promiscuity of PseI towards different amide derivatives we required a synthetic route to the different 6-deoxy-l-altdiamide substrates. To maximise synthetic efficiency we targeted a single precursor that could then be divergently derivatised and thus utilised Ling and co-worker's scalable synthesis^31^ (Scheme S1) to access protected 6-deoxy-altdiazide benzyl glycoside 8 (Fig. 2a). Subsequent 3-OH acylation could then afford formyl substituted diazide 9, acetyl substituted diazide 10, or (*R*)-3-hydroxybutyryl diazide 11. Staudinger reduction of both azides triggered regiospecific 3-O to 4-*N* acyl migration^32^ to afford 2-amino derivatives which could then be acylated to afford di-derivatised benzyl glycosides with formyl and (*R*)-3-hydroxybutyryl substituents (12), formyl and acetyl (13), diacetyl (14) and (*R*)-3-hydroxybutyryl and acetyl substituents (15). Finally, hydrogenation of the anomeric benzyl group afforded the diverse 6-deoxy altdiamide substrates for chemoenzymatic synthesis using PseI, 2Hb4Fm-l-alt*p*16, 2Ac4Fm-l-alt*p*17, 2Ac4Ac-l-alt*p*18, and 2Ac4Hb-l-alt*p*19.

**Fig. 2:**
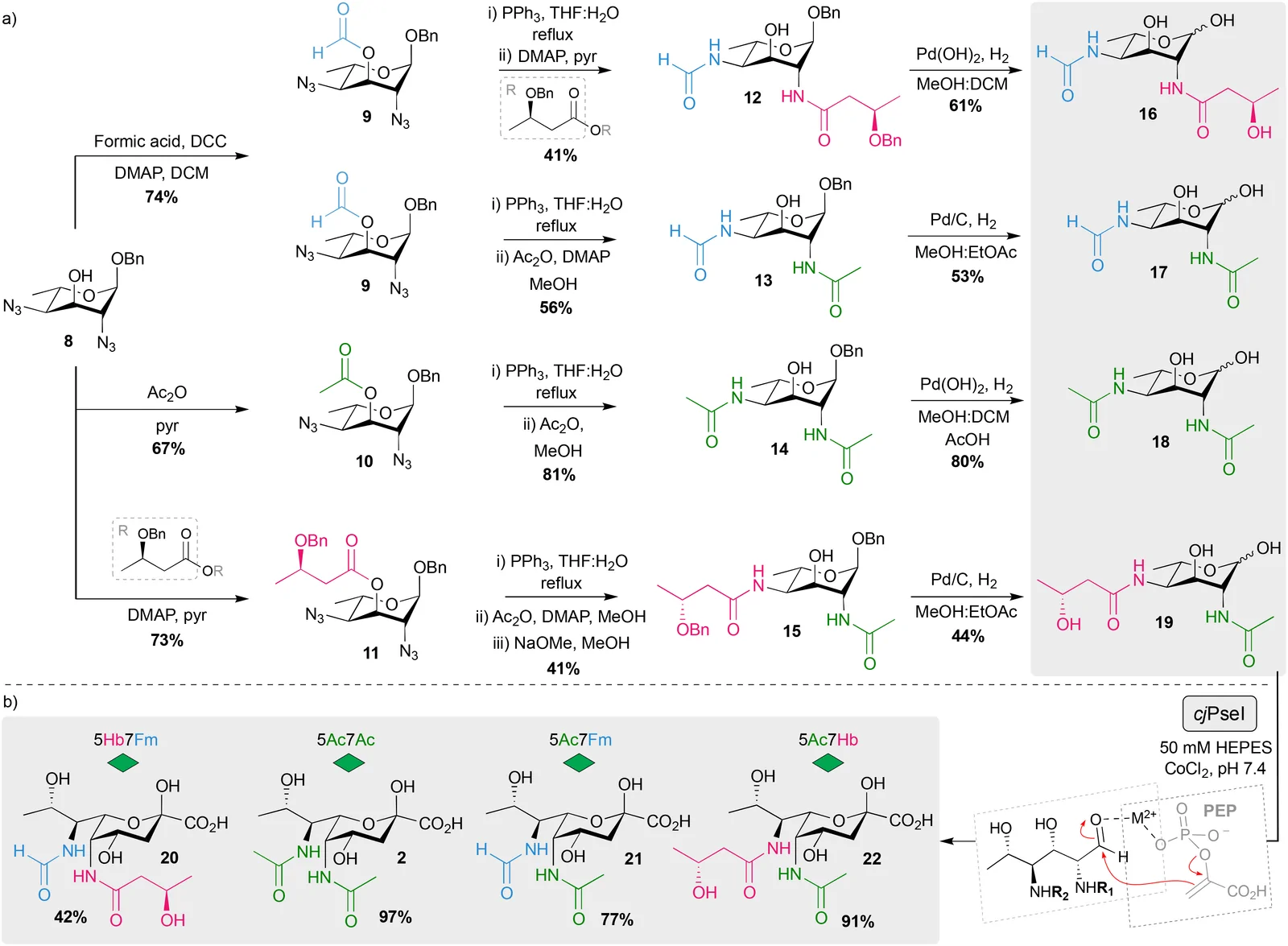
(a) Multi-step chemical synthesis of 6-deoxy-l-altdiamide derivatives 16–19 from a single diazido precursor 8 using regiospecific 3-O to 4-*N* acyl migration. (b) *cj*PseI enzyme catalysed synthesis of 5N, 7N differentially substituted Pse derivatives 2 and 20–22 from 6-deoxy-l-altdiamide derivatives 16–19 and phosphoenolpyruvate (PEP).

With the 6-deoxy-l-altdiamides 16–19 in hand, next we subjected these substrates to a preparative scale enzymatic reaction using the PseI enzyme from *C. jejuni*,^27,33^ which is a divalent metal dependant synthase that catalyses the condensation reaction between the *si*-face of the substrate in its aldehyde form and the *si*-face of phosphenolpyruvate (PEP) (Fig. 2b). To maximise yield we utilised Co^2+^ as the metal ion, which despite solubility issues has shown highest activity with *cj*PseI,^27^ and a pH 7.4 HEPES buffer which we found simplified purification, following addition of alkaline phosphatase at the end of the reaction to degrade excess PEP. We monitored these preparative scale reactions by LCMS and notably in the formation of Pse5Ac7Ac 2, Pse5Ac7Fm 21 and Pse5Ac7Hb 22 observed no starting material remaining after 24 hours (Fig. S2) using 0.2 mg mL^−1^ of enzyme and 1.5 equivalents of PEP at 30 °C, yielding between 9–13 mg (77–97%) of nonulosonic acid product after size exclusion chromatography. However, chemoenzymatic synthesis of the Pse5Hb7Fm 20 proved far more challenging, with starting material still detected by LCMS (Fig. S3) after 24 hours. To drive the reaction forward we therefore added fresh enzyme and PEP every 24 hours and allowed the reaction to proceed for another 6 days. Despite starting material still remaining, purification by size exclusion followed by anion exchange chromatography afforded 7 mg of Pse5Hb7Fm 20 (42% yield). This more challenging synthesis of Pse5Hb7Fm 20 from 2Hb4Fm derivatised 16 could be construed as a reduced tolerance of the larger hydroxybutyryl substituent at position C2 (6-deoxy altdiamide numbering) by the PseI enzyme, with bulky hydrophobic Phe129 and Phe108 residues, identified in the apo structure,^34^ likely in close proximity to this substituent in the enzyme active site. However, in the absence of a substrate bound structure further investigation of this hypothesis is required.

With Pse derivatives synthesised we set out to explore how and if other proteins are able to recognise these different substituents, and focussed on mapping the epitope specificity of the 1E8 monoclonal antibody recently raised against Pse5Ac7Ac glycosides and used to detect pseudaminylation in bacterial contexts.^18^ Previous work had already determined that the Fab fragment of 1E8 was able to bind and detect Pse glycosides modifed with larger derivatives, such as hydroxybutyryl in 22, at C7 (Pse numbering),^18^ so we focused on determining whether the 1E8 Fab could also tolerate larger hydroxybutyryl substituents at C5 by specifically comparing binding to Pse5Ac7Fm 21*versus* Pse5Hb7Fm 20. Using surface plasmon resonance, we first benchmarked 1E8 Fab binding to Pse5Ac7Ac 2 (Fig. 3), demonstrating that this antibody binds to the hemiketal form (*K*_D_ = 356 nM, Fig. 3a) with similar affinity to the α-glycoside form (*K*_D_ = 292 nM).^18^ This further supports the claim that this antibody is not influenced by the nature of the Pse aglycone. A comparative 4.5 fold reduction in binding affinity was observed for Pse5Ac7Fm 21 (*K*_D_ = 1.61 µM, Fig. 3b), indicating that the antibody can indeed recognise different *N*-acyl substituents at C7, albeit with a preference for an acetyl group at this position. However, no binding was observed between Pse5Hb7Fm 20 and 1E8 (Fig. 3c), indicating that the antibody cannot not tolerate the larger *N*-hydroxybutyryl substituent at C5. Consideration of the structure for Pse5Ac7Ac bound to 1E8 reveals the reason for this: the C5 *N*-acetyl substituent sits tightly in a hydrophobic pocket composed of residues Ala236 and Phe239, leaving no space to accommodate the larger *N*-hydroxybutyryl substituent in Pse5Hb7Fm 20 (Fig. 3d, PDB ID: 9PQS).

**Fig. 3:**
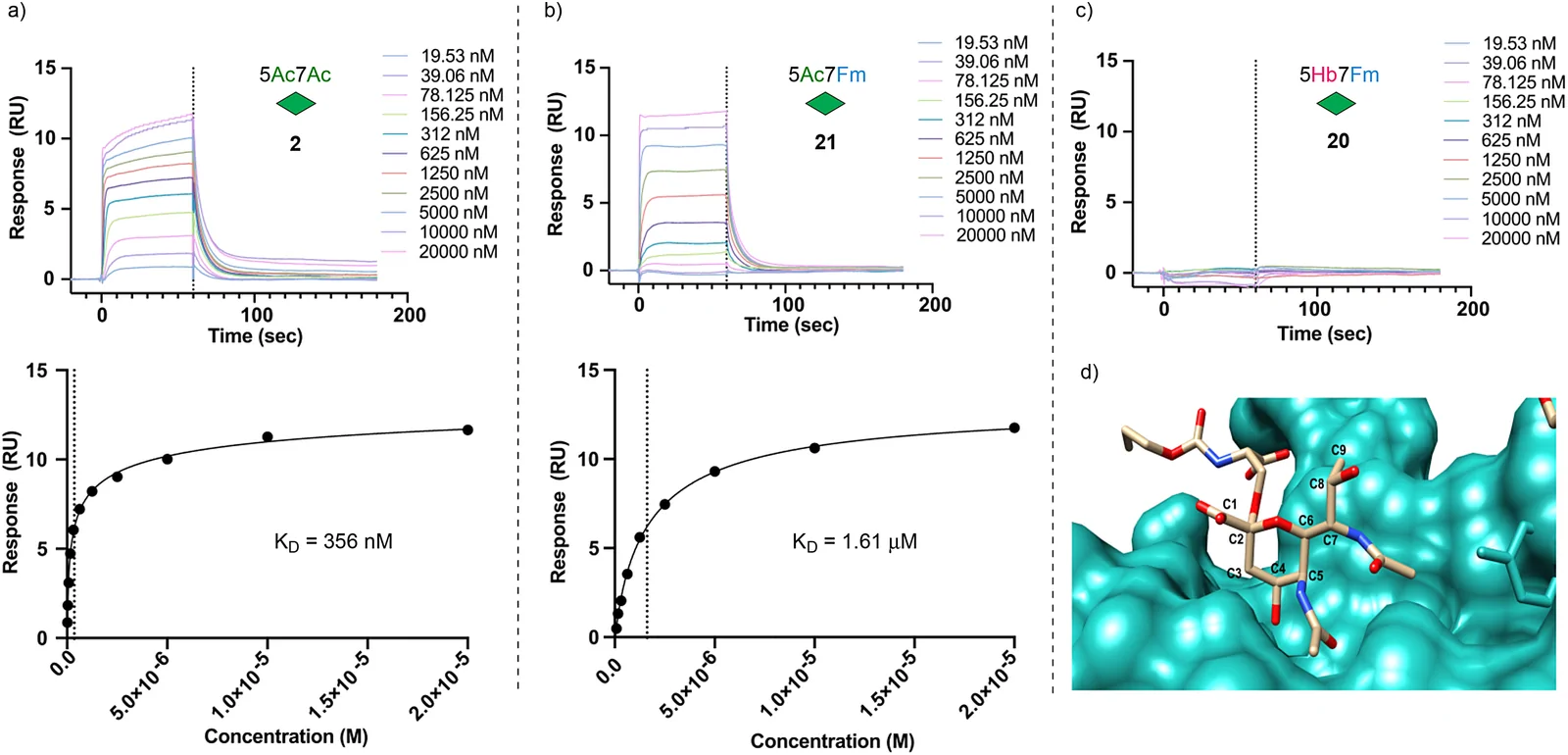
Surface plasmon resonance sensorgrams (top) and steady state response curves (bottom) for binding of immobilised 1E8 Fab to: (a) Pse5Ac7Ac 2; (b) Pse5Ac7Fm 21; and (c) Pse5Hb7Fm 20 (response curve omitted as no binding detected). (d) Crystal structure (PDB ID: 9PQS) of 1E8 antibody binding site bound to an a-Pse5Ac7Ac-Ser glycoside (sticks), demonstrating steric encumbrance around C5 acetyl group which prevents binding of Pse5Hb7Ac.

To conclude, in this work we demonstrate that a divergent synthesis from a single intermediate can be deployed to access differentially *N*-acylated substrates for *C. jejuni* pseudaminic acid synthase (PseI), and the promiscuity of this enzyme harnessed in the efficient synthesis of Pse derivatives. Furthermore we used these Pse derivatives to demonstrate that whilst the 1E8 monoclonal antibody is an effective tool for detecting diverse C7 substituted Pse sugars, due to steric restraints it is unable to tolerate a larger hydroxybutyryl substituent at C5, as is present in the Pse5Hb7Fm derivative presented within the LPS and on the pili of pathogenic *P. aeruginosa* O7 strains.^16^ We believe the chemoenzymatic preparative approach outlined here expediates access to the complex Pse nonulosonic acid architecture, reducing reliance on challenging chemical stereoselective 6 + 3 aldol-like condensations to construct the 9-carbon backbone, and will therefore stimulate development of even more tools to aid in dissection of the Pse*ome*.

## Author contributions

Natasha E. Hatton: investigation, methodology, visualization, writing – original draft, writing – review and editing. Niccolay Madiedo Soler: investigation, methodology, visualization. Ethan D. Goddard-Borger: funding acquisition, supervision, conceptualization, visualization, formal analysis, writing – review and editing. Martin A. Fascione: funding acquisition, supervision, conceptualization, visualization, writing – original draft, writing – review and editing.

## Conflicts of interest

There are no conflicts to declare.

## Supplementary Material

CB-007-D6CB00217J-s001

## Data Availability

The data supporting this article have been included as part of the supplementary information (SI). Supplementary information: experimental section and supplementary figures. See DOI: https://doi.org/10.1039/d6cb00217j.
