# Supplementary material for: Harnessing the promiscuity of a nonulosonic acid synthase for the chemoenzymatic synthesis of pseudaminic acid derivatives and specificity mapping of an anti-Pse antibody
Source: RSC Chem Biol. 2026 Sep 7;7(10):1986–90. doi: 10.1039/d6cb00217j (PMC13576459; doi:10.1039/d6cb00217j)
Supplement: Supplementary file 1 [file CB-007-D6CB00217J-s001.pdf]

Supplementary Information (SI) for RSC Chemical Biology.
This journal is © The Royal Society of Chemistry 2026

Supporting information

for

Harnessing the promiscuity of a nonulosonic acid synthase for the chemoenzymatic synthesis of pseudaminic acid derivatives and specificity mapping of an anti-Pse antibody

Natasha E. Hatton,[a] Niccolay Madiedo Soler,[b,c] Ethan D. Goddard-Borger[b,c] and Martin A. Fascione *[a]

[a] Department of Chemistry, University of York, York, YO10 5DD, UK.
[b] The Walter and Eliza Hall Institute of Medical Research, Parkville, Victoria 3052, Australia
[c] Department of Medical Biology, University of Melbourne, Parkville, Victoria 3010, Australia

General Appendix

General experimental procedures/information

The use of dry solvents is stated in individual methodologies. All dry solvents used were dried according to standard methods and solvents used for flash chromatography purposes were not dried prior to use. All chemical synthesis reactions were carried out in oven-dried glassware. Thin layer chromatography was carried out on Merck silica gel 60 F_{254} pre-coated aluminium foil sheets and were visualised using UV light (254 nm) and stained with a 5% sulphuric acid in EtOH stain (for sugars).

Spectroscopic and spectrometric instruments and standard practices

^{1}H-NMR (400 MHz) and ^{13}C-NMR (101 MHz) experiments were conducted using a JEOL 400 instrument, ^{1}H-NMR (700 MHz) and ^{13}C-NMR (176 MHz) were conducted on a Bruker Avance Neo 700 instrument at The University of York Centre for Magnetic Resonance. Me_4Si was often used as an internal standard at either 0.1% or 1% when using chloroform-d as an NMR solvent. Multiplicities are given as singlet (s), doublet (d), triplet (t), doublet of doublets (dd), doublet of doublet of doublets (ddd), triplet of doublets (td), quartet of doublets (qd) or multiplet (m). Resonances were assigned using HH-COSY and CH-HSQC. All NMR chemical shifts (δ) were recorded in ppm and coupling constants (J) are reported in Hz. Topspin 4.0.6 and MestReNovax64 were primarily used for processing the spectral data. The notation of the chemical environments to which the ^{1}H and ^{13}C NMR data corresponds to pyranose and nonulosonic acid sugars may be assigned as described in Figure S1.

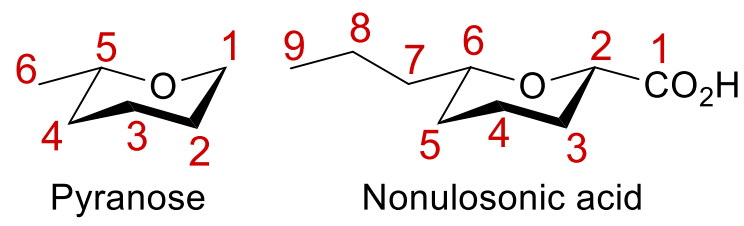

Figure S1. Depicts chemical environment for ^{1}H and ^{13}C assignment

Fourier transform infrared (FTIR) spectra were recorded on a PerkinElmer UATR 2 spectrometer using the attenuated total reflectance (ATR) technique. Optical rotations were measured using a Bellingham and Stanley ADP 450 Automatic Digital Peltier Controlled Polarimeter equipped with a 589 nm LED. Concentration is denoted as “c” and was calculated as grams per 100 millilitres (g / 100 mL) whereas the solvent is indicated in parenthesis (c, solvent).

ESI-MS experiments used for the characterisation of chemically synthesised molecules were conducted using a Bruker Daltonics micrOTOF mass spectrometer coupled to an Agilent 1200 series LC system, at The University of York Centre of Excellence in Mass Spectrometry (CoEMS). All the m/z values obtained are given in Daltons. More details regarding the ESI-LC/MS analysis of enzymatic reactions can be found below.

LCMS Procedures

ESI-LC/MS

LC-MS analysis was performed on a Dionex UltiMate® 3000 Ci Rapid Separation LC system equipped with an UltiMate® 3000 photodiode array detector probing at 250-400 nm coupled to a HCT ultra ETD 11 (Bruker Daltonics) ion trap spectrometer, using Chromeleon® 6.80 SR12 software (ThermoScientific), Compass 1.3 for esquire HCT build 581.3, esquire Control version 6.2, Build 62.24 software (Bruker Daltonics) and Bruker compass HyStar 3.2-SR2, HyStar version 3.2, Build 44 software (Bruker Daltonics) at The University of York Centre of Excellence in Mass Spectrometry (CoEMS). Data analysis was performed using ESI compass 1.3 DataAnalysis, Version 4.4 software (Bruker Daltonics).

General analysis of enzymatically synthesised sugars

Samples were analysed using a Accucore hydrophilic interaction chromatography (HILIC) column (2.6 um particle size, 50 x 2.1 mm). Water + 0.1% formic acid by volume (solvent A) and acetonitrile + 0.1% formic acid (solvent B) were used as a mobile phase; the column temperature was set to 35°C and flow rate was set to 250 µL/min. Samples were analysed using a 15-minute method, for the 15-minute method, a multi-step gradient was programmed as follows: 95% B for 1 min, followed by a linear gradient to 5% B over 9.5 min, followed by 5% B for an additional 0.5 min. Then a linear gradient to 95% B over 1 min, followed by holding at 95% B for 3 min.

Experimental details for *C. jejuni* PseI enzyme production.

Plasmid details, and protein expression and purification procedures can be found in previous publications by our group.[1]

Synthesis of diazido intermediate **8**

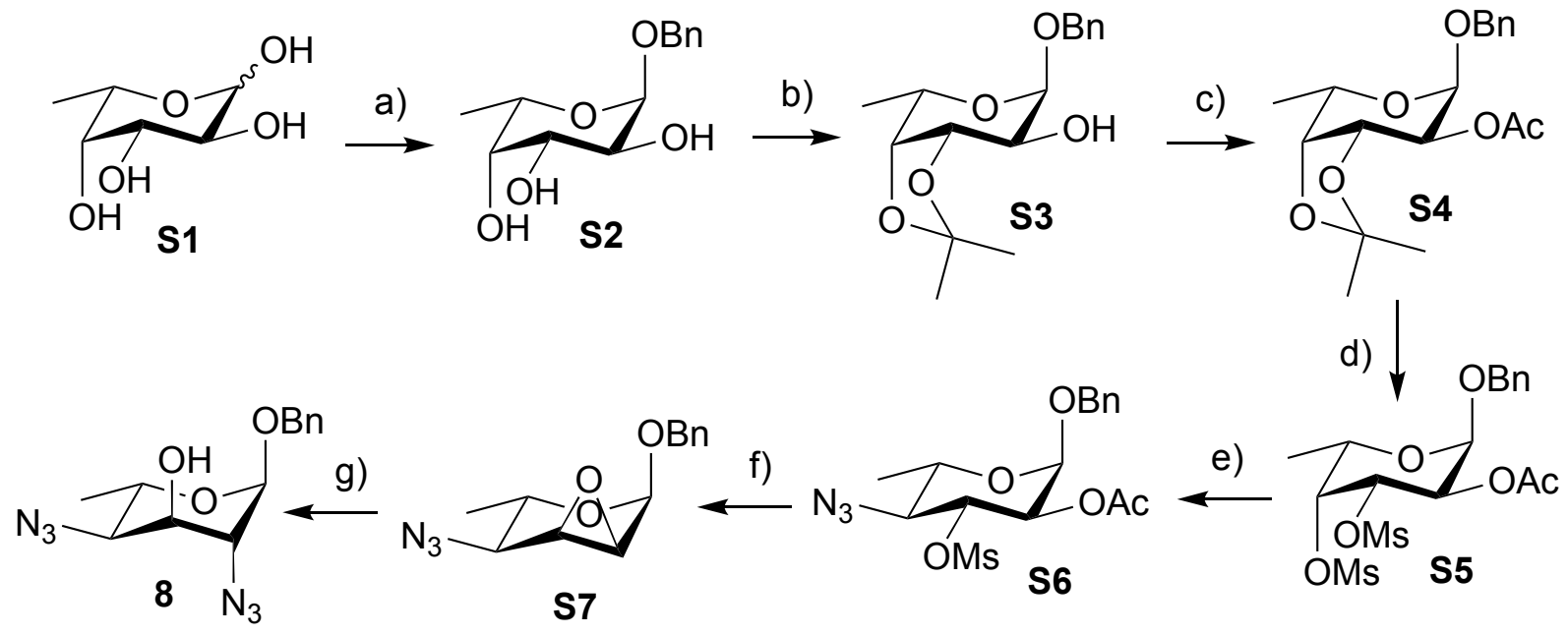

Scheme S1. Synthesis scheme of benzyl 2,4-diazido-2,4,6-trideoxy-α-L-altropyranoside (diazido intermediate **8**) from L-fucose **S1**. Reagents and conditions; a) BnOH, AcCl, 90 °C, 22 h b) 2,2 dimethoxypropane, CSA, Acetone, reflux, 16 h, 20% over two-steps c) Ac_2O, NEt_3, DMAP, DCM, 0°C-RT, 1 h, 62% d) i) 85% AcOH, 80 °C, 2h, ii) MsCl, DCM, pyridine, RT, 16 h, 79% e) NaN_3, DMF, 90 °C, 72 h, 100 °C, 4 h, 67% f) NaOMe, MeOH, RT, 16h, 74% g) NaN_3, $LiClO_4$, MeCN, 85 °C, 48 h, 59%.

Synthesis of benzyl-α/β-L-fucopyranoside (S2)[2]

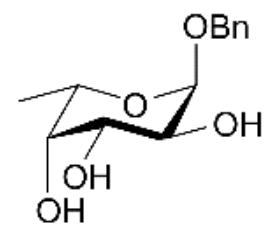

Acetyl chloride (1.48 mL, 20.9 mmols, 0.38 eq) was added dropwise to a solution of benzyl alcohol (45 mL, 440 mmols, 8 eq) at 0 °C, the reaction mixture was stirred for 10 minutes affording a colourless solution. L-Fucose (9 g, 55 mols, 1 eq) was added and the reaction mixture was heated to 90 °C affording a yellow solution, which was stirred for 22 h under N_2. The reaction mixture was cooled to RT and poured into hexane (600 mL) to afford a crude yellow wet solid containing **S2**. The solid was used crude in further reactions.

(ESI)HRMS – $C_{13}H_{18}NaO_5^+$ ($[M+Na]^+$) requires m/z 277.11: found m/z 277.1047

Synthesis of benzyl 3,4-*O*-isopropylidene-α-L-fucopyranoside (S3)[2]

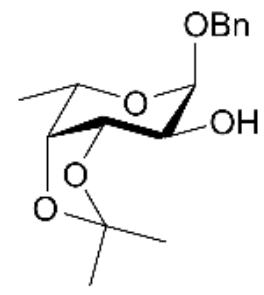

A crude sample of **S2** (13.97 g, 55 mmols, 1eq) in acetone (44 mL) was charged with CSA (0.178 g, 0.77 mmols, 0.014 eq) and 2,2 dimethoxypropane (44 mL, 357.5 mmols, 6.5 eq) at 0 °C. The reaction mixture was refluxed for 16 h under N_2 affording a light-yellow solution. The solution was cooled to RT and neutralized using NEt_3. The crude solution was concentrated *in vauco* to give a light-yellow oil which was purified by column chromatography eluting with 100% hexane to 100% EtOAc affording a colourless oil of **S3** (3.166 g, 10.76 mmols, 20% over two steps); R_f = 0.56 (40 % EtOAc in Hexane).

^{1}H NMR (400 MHz, $CDCl_3$): δ_H 7.39-7.28 (m, 5H, HAr), 4.94 (d, $J_{1,2}$ = 4.0 Hz, 1H, H-1), 4.78 (d, J_{CH2} = 11.9 Hz, 1H, CH_2), 4.57 (d, J_{CH2} = 11.9 Hz, 1H, CH_2), 4.22 (dd, $J_{3,4}$ = 6.1 Hz, $J_{2,3}$ = 6.3 Hz, 1H, H-3), 4.15 (dq, $J_{5,6}$ = 6.8 Hz, $J_{4,5}$ = 2.3 Hz, 1H, H-5), 4.05 (dd, $J_{3,4}$ = 6.1 Hz, $J_{4,5}$ = 2.3 Hz, 1H, H-4), 3.81 (dd, $J_{2,3}$ = 6.3 Hz, $J_{1,2}$ = 4.0 Hz, 1H, H-2), 2.42 (d, 1H, OH), 1.51 (s, 3H, CH_3), 1.35 (s, 3H, CH_3), 1.31 (3H, d, $J_{5,6}$ = 6.8 Hz, H-6). **^{13}C NMR** (101 MHz, $CDCl_3$): δ_C 137.24 (1C), 128.61 (2C), 128.07 (2C), (CAr), 109.29 (1C, $\underline{C}$), 97.02 (1C, C-1), 76.23 (1C, C-3), 75.73 (1C, C-4), 69.78 (1C, CH_2), 69.47 (1C, C-2), 64.18 (1C, C-5), 27.87 (1C, $\underline{C}H_3$), 26.01 (1C, $\underline{C}H_3$), 16.35 (1C, $\underline{C}H_3$).**(ESI)HRMS** – $C_{13}H_{22}NaO_{11}^+$ ($[M+Na]^+$) requires m/z 317.14: found m/z 317.1362. **FT-IR (ATR)**- 3453.70 (O-H), 2985.75 (C-H), 2935.29 (C-H), 1380.23 (C-O), 1217.89 (C-O), 1071.29 (C-O). ***[α]D*^{25}** = -78.3 (*c* 0.465, DCM)

Synthesis of benzyl 2-*O*-acetyl-3,4-*O*-isopropylidene-α-L-fucopyranoside (S4)[2]

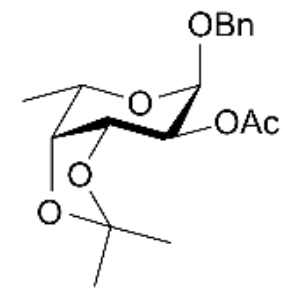

A solution of **S3** (0.47 g, 1.6 mmols, 1 eq) in dry DCM (10 mL) was charged with NEt_3 (0.56 mL, 4 mmols, 2.5 eq), DMAP (cat) and Ac_2O (0.16 mL, 1.76 mmols, 1.1 eq) at 0 °C affording a light-yellow solution. The solution was stirred at RT under N_2 for 1 h affording a light-yellow solution. The solution was concentrated *in vacuo*, dissolved in EtOAc (20 mL), washed with 1 M HCl (20 mL), saturated $NaHCO_3$ (20 mL) and saturated NaCl (20 mL), dried ($MgSO_4$) and concentrated *in vacuo* affording a colourless oil. This oil was purified by column chromatography eluting from 100% Hexane to 100% EtOAc affording a white solid of **S4** (0.335 g, 1.0 mmols, 62%); R_f = 0.57 (20 % EtOAc in Hexane). **^{1}H NMR** (400 MHz, $CDCl_3$): δ_H 7.37-7.26 (m, 5H, HAr), 4.99 (d, $J_{1,2}$ = 3.6 Hz, 1H, H-1), 4.91 (dd, $J_{2,3}$ = 8.2 Hz, $J_{1,2}$ = 3.6 Hz, 1H, H-2), 4.70 (d, J_{CH2} = 12.6 Hz, 1H, CH_2), 4.52 (d, J_{CH2} = 12.6 Hz, 1H, CH_2), 4.36 (dd, $J_{2,3}$ = 8.2 Hz, $J_{3,4}$ = 5.2 Hz, 1H, H-3), 4.16 (dq, $J_{5,6}$ = 6.5 Hz, $J_{4,5}$ = 2.4 Hz, 1H, H-5), 4.09 (dd, $J_{3,4}$ = 5.2 Hz, $J_{4,5}$ = 2.4 Hz, 1H, H-4), 2.08 (s, 3H, CH_3), 1.53 (s, 3H, CH_3), 1.35 (m, 6H, H-6, CH_3). **^{13}C NMR** (101 MHz, $CDCl_3$): δ_C 170.59 (1C), 137.37 (1C), 128.50 (1C), 127.96 (1C), 127.73 (1C), (CAr), 109.38 (1C, C), 95.50 (1C, C-1), 76.22 (1C, C-4), 73.45 (1C, C-3), 72.03 (1C, C-2), 69.68 (1C, CH_2), 63.47 (1C, C-5), 28.12 (1C, CH_3), 26.49 (1C, CH_3), 21.05 (1C, CH_3), 16.35 (1C, CH_3). **(ESI)HRMS** – $C_{18}H_{24}NaO_6^+$ ($[M+Na]^+$) requires m/z 359.15: found m/z 359.1475. **FT-IR (ATR)** – 2986.49 (C-H), 2936.49 (C-H), 1738.51 (C=O), 1367.71 (C-O), 1219.01 (C-O) 1052.38 (C-O). ***[α]D^{25}*** = -158.0 (*c* 1, DCM)

Synthesis of benzyl 2-*O*-acetyl-3,4-di-*O*-methanesulfonyl-α-L-fucopyranoside (S5)[2]

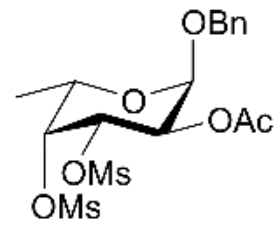

A solution of **S4** (0.9535 g, 2.8 mmols, 1 eq) in 85% acetic acid (20 mL) was heated to 80 °C for 2 h affording a clear solution. The solution was cooled to RT and concentrated *in vacuo* azeotroping with toluene three times to afford a crude white solid. The solid was dissolved in dry DCM (6 mL), cooled to 0 °C and charged with pyridine (3 mL) and MsCl (1.4 mL, 14.18 mmols, 5 eq) and stirred for 16 h at RT under N_2 affording a light brown solution. The reaction was quenched with water (20 mL) and the organic layer was extracted and washed with 1 M HCl (20 mL), saturated $NaHCO_3$ (20 mL) and saturated NaCl (20 mL), dried ($MgSO_4$) and concentrated *in vacuo* to afford a crude oil. This oil was purified by column chromatography eluting with a graduated solvent system of 100% Hexane to 100% EtOAc affording a colourless oil of **S5** (1.010 g, 2.2 mmols, 79%); R_f = 0.5 (40 % EtOAc in Hexane). **^{1}H NMR** (400 MHz, $CDCl_3$): δ_H 7.39-7.29 (m, 5H, HAr), 5.16-5.10 (m, 3H, H-1, H-2, H-3), 5.03 (m, 1H, H-4), 4.69 (d, J_{CH2} = 11.94 Hz, 1H, CH_2), 4.56 (d, J_{CH2} = 11.94 Hz, 1H, CH_2), 4.15 (m, 1H, H-5), 3.19 (s, 3H, CH_3 (Ms)), 3.11 (s, 3H, CH_3 (Ms)), 2.08 (s, 3H, CH_3 (Ac)), 1.28 (s, 3H, H-6). **^{13}C NMR** (101 MHz, $CDCl_3$): δ_C 169.8, 136.7, 128.7, 128.4, 128.1 (HAr), 95.4 (C-1), 80.7 (C-4), 74.5 (C-3), 70.4 (CH_2), 67.8 (C-2), 64.9 (C-5), 39.2 (CH_3 (Ms)), 38.7 (CH_3 (Ms)), 20.82 (CH_3 (Ac)), 16.4 (C-6). **(ESI)HRMS** – $C_{17}H_{24}NaO_{10}S_2^+$ ($[M+Na]^+$) requires m/z 475.07: found m/z 475.0692. **FT-IR (ATR)** – 3033.24 (C-H), 2941.23 (C-H), 1747.79 (C=O), 1358.23 (S=O), 1230.22 (C-O), 1178.24 (C-O)**.** ***$[\alpha]_D^{25}$*** = -132.2 (*c* 1, DCM)

Synthesis of benzyl 2-*O*-acetyl-4-azido-3-*O*-methanesulfonyl-α-L-fucopyranoside (S6)[2]

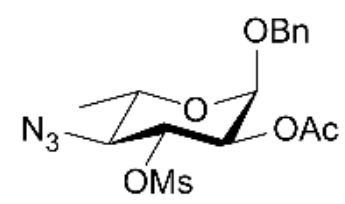

NaN_3 (0.37g, 5.7 mmols, 5.2 eq) was added to a solution of **S5** (0.5 g, 1.1 mmols, 1eq) in dry DMF (10 mL). The reaction mixture was stirred at 90 °C for 72 h at which point the reaction mixture was heated to 100 °C and stirred for 4 h affording a colourless solution containing a white solid. The solution was cooled to RT and concentrated *in vacuo* to afford a crude white oil. The oil was dissolved in EtOAc (20 mL) and washed with saturated NaCl solution (20 mL), the aqueous layer was washed a further three times with EtOAc (20 mL) and the organic layers were combined, dried ($MgSO_4$) and concentrated *in vacuo* to give a colourless oil. The oil was purified using column chromatography eluting with a graduated solvent system of 100% Hexane to 100% EtOAc affording a colourless oil of **S6** (0.2924 g, 0.73 mmols, 67%); Rf = 0.86 (40 % EtOAc in Hexane). **^{1}H NMR** (400 MHz, $CDCl_3$): δ_H 7.37-7.27 (m, 5H, HAr), 5.01 (d, $J_{1,2}$ = 3.6 Hz, 1H, H-1), 4.97 (dd, $J_{2,3}$ = 9.9 Hz, $J_{3,4}$ = 9.9 Hz, 1H H-3), 4.88 (dd, $J_{2,3}$ = 9.9 Hz, $J_{1,2}$ = 3.4 Hz, 1H, H-2), 4.68 (d, J_{CH2} = 12.3 Hz, 1H, CH_2), 4.52 (d, J_{CH2} = 12.3 Hz, 1H, CH_2), 3.75 (dq, $J_{4,5}$ = 9.9 Hz, $J_{5,6}$ = 6.2 Hz, 1H, H-5), 3.24 (dd, $J_{3,4}$ = 9.9 Hz, $J_{4,5}$ = 9.9 Hz, 1H, H-4), 3.09 (s, 3H, CH_3 (Ms)), 2.08 (s, 3H, CH_3 (Ac)), 1.32 (d, $J_{5,6}$ = 6.2 Hz, 3H, H-6). **^{13}C NMR** (101 MHz, $CDCl_3$): δ_C 169.96, 136.67, 128.45, 128.07, 127.84 (CAr), 94.99 (C-1), 78.33 (C-3), 70.42 (C-2), 69.80 (CH_2), 66.53 (C-4), 66.32 (C-5), 38.87 (CH_3 (Ms)), 20.66 (CH_3 (Ac)), 18.07 (1C, C-6). **(ESI)HRMS** – $C_{16}H_{21}N_3NaO_7S^+$ ($[M+Na]^+$) requires m/z 422.10: found m/z 422.0998. **FT-IR (ATR)** – 3032.83 (C-H), 2981.86 (C-H), 2938.68 (C-H), 2109.14 (N_3), 1751.11 (C=O), 1351.68 (S=O), 1224.43 (C-O), 1177.09 (C-O). ***$[\alpha]_D^{25}$*** = -202.0 (*c* 1, DCM)

Synthesis of benzyl 2,3-anhydro-4-azido-4,6-dideoxy-α-L-allopyranoside (S7)[2]

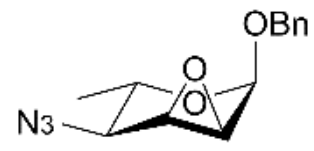

NaOMe (0.075 g, 1.4 mmols, 1.2 eq) was added to a solution of **S6** (0.4621 g, 1.16 mmols, 1 eq) in dry MeOH (4 mL) affording a colourless solution. The solution was stirred for 16 h at RT affording a colourless solution containing a white precipitate which was diluted with MeOH (20 mL) to give a colourless solution. The solution was neutralized with dowex™ (hydrogen form) and concentrated *in vacuo* to give a crude colourless oil. The oil was purified by column chromatography eluting with a graduated solvent system of 100% Hexane to 100% EtOAc affording a colourless oil of **S7** (0.2247 g, 0.86 mmols, 74%); Rf = 0.68 (20 % EtOAc in Hexane). **^{1}H NMR** (400 MHz, $CDCl_3$): δ_H 7.39-7.26 (m, 5H, HAr), 4.98 (d, $J_{1,2}$ = 3.2 Hz, 1H, H-1), 4.75 (d, J_{CH2} = 12.3 Hz, 1H, CH_2), 4.62 (d, J_{CH2} = 12.3 Hz, 1H, CH_2), 3.93 (m, 1H, H-5), 3.51 (dd, $J_{2,3}$ = 3.5 Hz, $J_{3,4}$ = 1.1 Hz, 1H, H-3), 3.45 (dd, $J_{1,2}$ = 3.5 Hz, $J_{2,3}$ = 3.5 Hz, 1H, H-2), 3.14 (dd, $J_{4,5}$ = 9.6 Hz, $J_{3,4}$ = 1.1 Hz, 1H, H-4), 1.21 (d, $J_{5,6}$ = 6.3 Hz, 3H, H-6). **^{13}C NMR** (101 MHz, $CDCl_3$): δ_C 137.5 (1C), 128.5 (2C), 128.1 (2C), 127.9 (1C), (CAr), 92.1 (C-1), 69.5 (CH_2), 63.2 (C-5), 61.5 (C-4), 53.7 (C-2), 52.3 (C-3), 17.85 (C-6). **(ESI)HRMS** – $C_{13}H_{15}N_3NaO_3^+$ ($[M+Na]^+$) requires m/z 284.1011: found m/z 284.1010. **FT-IR (ATR)** – 2978.68 (C-H), 2932.25 (C-H), 2103.21 (N_3), 1047.23 (C-O). ***[α]D^{25}*** = -198.6 (*c* 0.6, DCM)

Synthesis of benzyl 2,4-diazido-2,4,6-trideoxy-α-L-altropyranoside (8)[2]

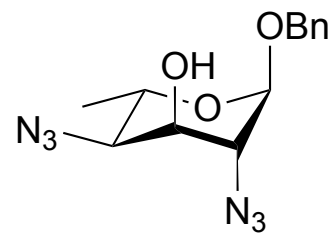

$LiClO_4$ (0.053 g, 0.5 mmols, 4 eq) was added to a solution of **S7** (0.033 g, 0.126 mmols, 1 eq) and NaN_3 (0.032 g, 0.5 mmols, 4 eq) in dry MeCN (5 mL) affording a cloudy solution which was heated to 85 °C and stirred for 48 h affording a cloudy solution. The solution was cooled to RT and concentrated *in vacuo* affording a crude oil. The oil was dissolved in EtOAc (10 mL) and washed with saturated NaCl solution (10 mL), dried ($MgSO_4$) and concentrated *in vacuo* affording a colourless oil. The oil was purified by column chromatography eluting with a graduated solvent system of 100% Hexane to 100% EtOAc affording a colourless oil of **S8** (0.0226 g, 0.07 mmols, 59%); Rf = 0.66 (20 % EtOAc in Hexane). ^{1}**H NMR** (400 MHz, $CDCl_3$): δ_H 7.42-7.31 (m, 5H, HAr), 4.88 (s, 1H, H-1), 4.76 (d, J_{CH2} = 11.9 Hz, 1H, CH_2), 4.58 (d, J_{CH2} = 11.9 Hz, 1H, CH_2), 4.11-4.02 (m, 2H, H-3, H-5), 3.84 (dd, *J* 3.9 Hz, *J* = 1.8 Hz, 1H, H-2), 3.44 (d, $J_{4,OH}$ = 9.8 Hz, 1H, OH), 3.22 (dd, $J_{4,OH}$ = 9.8 Hz, *J* = 3.0 Hz, 1H, H-4), 1.37 (d, 6.1 Hz, 3H, H-6). ^{13}**C NMR** (101 MHz, $CDCl_3$): δ_C 135.9 (1C), 128.9 (2C), 128.7 (1C), 128.4 (2C) (CAr), 97.0 (1C, C-1), 70.4 (1C, CH_2), 69.6 (1C, C-3), 63.2 (1C, C-5), 61.4 (1C, C-4), 60.9 (1C, C-2), 18.3 (1C, CH_3). **(ESI)HRMS** – $C_{13}H_{16}N_6NaO_3^+$ ($[M+Na]^+$) requires m/z 327.1182: found m/z 327.1181. **FT-IR (ATR)** – 3493.57 (O-H), 3033.10 (C-H), 2934.44 (C-H), 2980.27 (C-H), 2102.62 (N_3), 1261.24 (C-O), 1021.23 (C-O). ***[α]D^25^*** = -102.7 (*c* 1, DCM)

Synthesis of (*R*)-3-benzyloxybutanoic anhydride (S10)

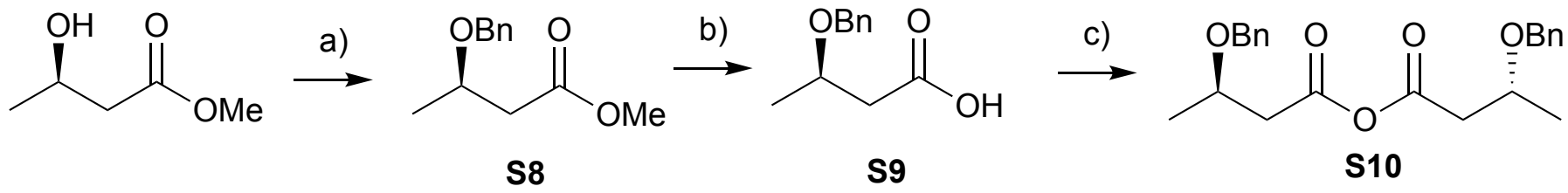

Scheme S2. Synthesis of (R)-3-Benzyloxybutanoic anhydride (**S10**) from Methyl(R)-3-hydroxybutyrate. Reagents and conditions; a) Benzyl 2,2,2-trichloroacetimidate, 4Å MS, 2 : 1 cyclohexane : DCM, TfOH, 0 °C to RT, 16 h, 54%, b) LiOH, H_2O, RT, 16 h, 76%, c) DCC, DCM, 0 °C to RT, 16 h.

Synthesis of methyl (R)-3-(benzyloxy)butanoate (S8)[3]

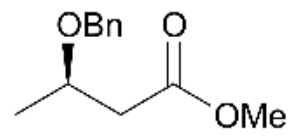

A solution of Methyl-(R)-3-hydroxybutyrate (4.7 mL, 42.37 mmols, 1 eq), Benzyl 2,2,2-trichloroacetimidate (9.42 mL, 50.8 mmols, 1.2 eq) and 4Å MS (1.5 g) in a 2 : 1 solution of dry Cyclohexane : dry DCM (10 : 5 mL) was cooled to 0 °C and stirred for 30 minutes. TfOH (0.75 mL 8.47 mmols, 0.2 eq) was added to the solution dropwise affording a yellow solution which was warmed to RT and stirred for 16 h. The reaction was quenched by the addition of NEt_3 and the solution was filtered through celite and concentrated *in vacuo* to afford a yellow oil. The oil was purified by column chromatography eluting with a graduated solvent system of 100% hexane to 100% EtOAc affording a light-yellow oil of **S8** (4.836 g, 23 mmols, 54%); R_f = 0.7 (20% EtOAc in Hexane). **^{1}H NMR** (400 MHz, $CDCl_3$): δ_H 7.34-7.20 (m, 5H, HAr), 4.56 (d, *J* = 11.4 Hz, 1H, CH_2 (OBn)), 4.49 (d, J = 11.4 Hz, 1H, CH_2 (OBn)), 4.05-3.96 (m, 1H, CH), 3.66 (s, 3H, CH_3 (OMe)), 2.64 (dd, *J* = 15.1, J = 6.5 Hz, 1H, CH_2), 2.43 (dd, *J* = 15.1 Hz, *J* = 6.5 Hz, 1H, CH_2), 1.25 (d, *J* = 6.2, 1H, CH_3). **^{13}C NMR** (101 MHz, $CDCl_3$): δ_C 172.0 (C=O), 138.5, 128.4, 127.7, 127.6 (CAr), 72.0 (CH), 70.9 (CH_2 (OBn)), 51.7 (OCH_3), 41.9 (CH_2), 19.9 (CH_3). **(ESI)HRMS** – $C_{12}H_{16}NaO_3^+$ ($[M+Na]^+$) requires m/z 231.0097: found m/z 231.0991

Synthesis of (*R*)-3-benzyloxybutanoic acid (S9)[3]

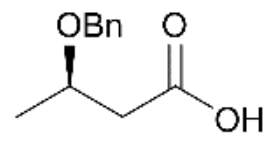

A solution of LiOH in water (1.11 g, 46 mmols, 2 eq, in 15 mL water) was added dropwise to a solution of **S8** (4.83 g, 23 mmols, 1 eq, in 22 mL THF) at 0 °C and the solution was stirred vigorously at RT for 16 h affording a light-yellow solution. The solution was acidified with 1M HCl and washed with EtOAc (100 mL) the organic layer was extracted, dried ($MgSO_4$) and concentrated *in vacuo* affording a colourless oil. The oil was purified by column chromatography eluting with a graduated solvent system of 100% hexane to 100% EtOAc affording a colourless oil of **S9** (3.38 g, 17.4 mmols, 76%); R_f = 0.35 (20% EtOAc in Hexane). **^{1}H NMR** (400 MHz, $CDCl_3$): δ_H 7.35 – 7.17 (m, 5H, HAr), 4.56 (d, *J* = 11.3 Hz, 1H, CH_2 (OBn)), 4.50 (d, *J* = 11.3 Hz, 1H, CH_2 (OBn)), 4.05-3.92 (m, 1, CH), 2.66 (dd, *J* = 15.1 Hz, *J* = 6.0 Hz, 1H, CH_2), 2.46 (dd, *J* = 15.1 Hz, *J* = 6.0 Hz, 1H, CH_2), 1.26 (d, *J* = 6.1 Hz, CH_3). **^{13}C NMR** (101 MHz, $CDCl_3$): δ_C 177.3 (C=O), 138.2, 128.4, 127.8, 127.7 (CAr), 71.7 (CH), 70.9 (CH_2), 41.8 (CH_2), 19.7 (CH_3). **(ESI)HRMS** – $C_{11}H_{14}NaO_3^+$ ($[M+Na]^+$) requires m/z 217.0841: found m/z 217.0834

Synthesis of (*R*)-3-benzyloxybutanoic anhydride (S10)[3]

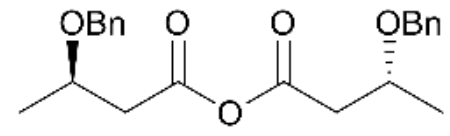

DCC (4.12 g, 20 mmols, 2 eq), was added to a solution of **S9** (2.10 g, 10 mmols, 1 eq) in dry DCM (13 mL) at 0 °C. The solution was warmed to RT and stirred for 16 h affording a clear solution with a white precipitate. The precipitate was removed via filtration and the filtrate was concentrated *in vacuo* affording a colourless oil containing **S10** (4.8 g); Rf = 0.57 (20% EtOAc in Hexane), which was used crude in subsequent reactions. **(ESI)HRMS** – $C_{22}H_{26}NaO_5^+$ ($[M+Na]^+$) requires m/z 392.1678: found m/z 393.1676

Derivatisation to 2,4,6-trideoxy-4-formamido-2-[(*R*)-3-hydroxybutanamido]-α-L-altropyranose (**16**)

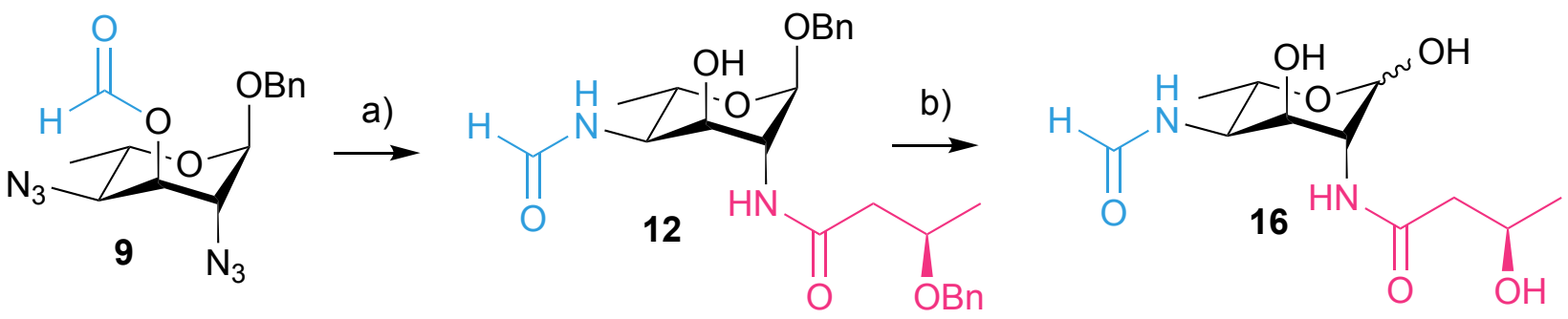

Scheme S3. Synthesis of 2,4,6-trideoxy-4-formamido-2-[(R)-3-hydroxybutanamido]-α-L-altropyranose (**16**) from **9**. Reagents and conditions; a) i) PPh_3, 10 : 1 THF : water, reflux, 16 h, ii) benzyloxybutanoic anhydride **S10**, pyridine, DMAP, RT, 16 h, 41%, b) $Pd(OH)_2/C$, AcOH, H_2, 10 : 1 MeOH : DCM, RT, 16 h, 61%.

Derivatisation to 2-acetamido-2,4,6-trideoxy-4-formamido-α-L-altropyranose (**17**)

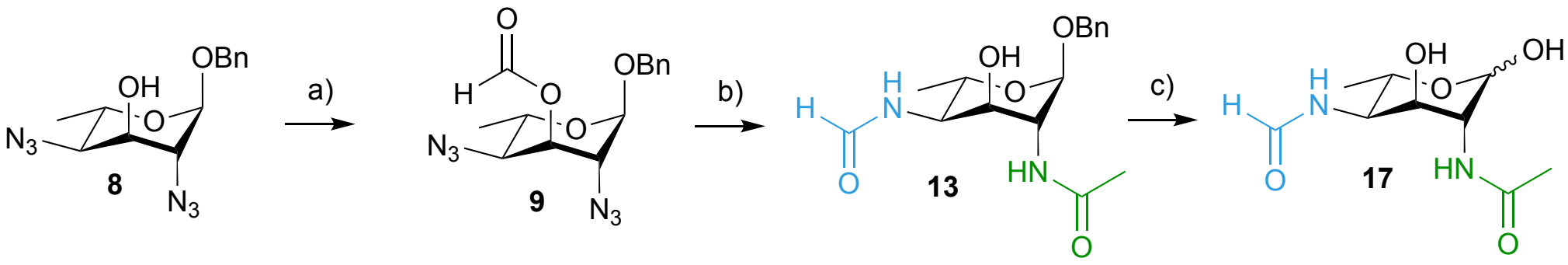

Scheme S4. Synthesis of 2-acetamido-2,4,6-trideoxy-4-formamido-α-L-altropyranose (**17**) from diazido intermediate (**8**). Reagents and conditions; a) DCC, DMAP, Formic acid, DCM, 0 °C to RT, 16 h, 74%; b) i) PPh_3, 10 : 1 THF : H_2O, reflux, 16 h; ii) Ac_2O, MeOH, DMAP, 0 °C to RT, 3 h, 56%; c) Pd/C, 2 : 1 MeOH : EtOAc, H_2, RT, 40 h, 53%

Derivatisation to 2,4-diacetamido-2,4,6-trideoxy-α-L-altropyranose (**18**)

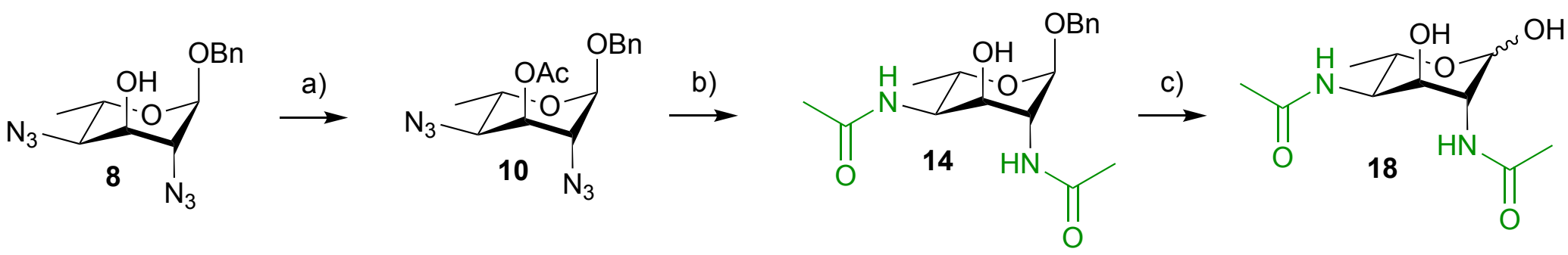

Scheme S5. Synthesis of 2,4-diacetamido-2,4,6-trideoxy-α-L-altropyranose (**18**) from diazido intermediate (**8**). Reagents and conditions; a) Ac_2O, Pyridine, 0 °C-RT, 5 h, 67%; b) i) PPh_3, 10 : 1 THF : H_2O, reflux, 24 h ii) Ac_2O, MeOH, 0 °C - RT, 16 h, 81%; $Pd(OH)_2/C$, AcOH, 5 : 1 MeOH, DCM, RT, 6h, 80%.

Derivatisation to 2-acetamido-2,4,6-trideoxy-4-[(*R*)-3-hydroxybutanamido]-α-L-altropyranose (**19**)

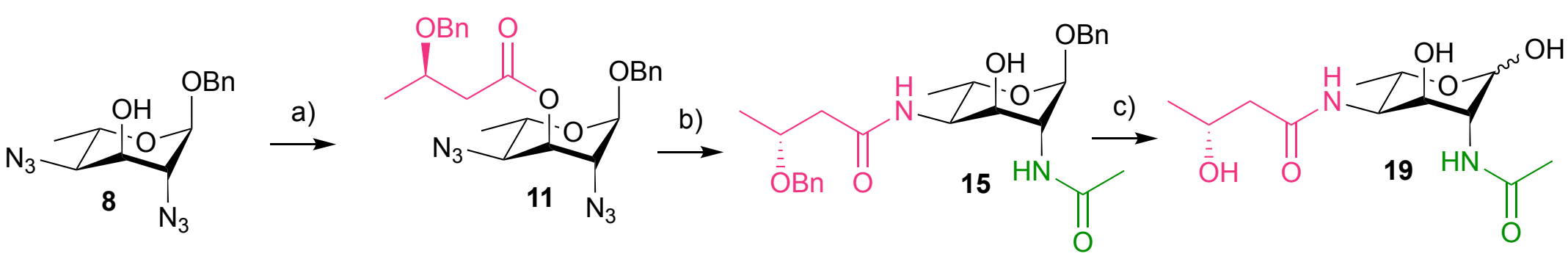

Scheme S6. Synthesis of 2-acetamido-2,4,6-trideoxy-4-[(R)-3-hydroxybutanamido]-α-L-altropyranose (**19**) from 2,4-diazido-2,4,6-trideoxy-α-L-altropyranoside (**8**). Reagents and conditions; a) Benzyloxybutanoic anhydride, DMAP, pyridine, 0°C to RT, 16 h, 73%; b) i) PPh_3, 10 : 1 THF : Water, Reflux, 16 h, ii) MeOH, DMAP, Ac_2O, 0°C to RT, 3h, iii) MeOH, NaOMe, RT, 16 h, 41%; c) Pd/C, 2 : 1 MeOH, EtOAc, H_2, RT, 48 h, 44%.

Synthesis of benzyl 2,4-diazido-3-*O*-formyl-2,4,6-trideoxy-α-L-altropyranoside (9)[4]

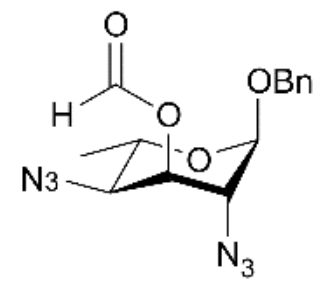

DCC (0.378 g, 1.8 mmols, 2 eq) was added dropwise to a solution of **8** (0.279g, 0.92 mmols, 1 eq), DMAP (cat) and formic acid (68 µL, 1.8 mmols, 2 eq) in dry DCM (5 mL) at 0 °C. The reaction mixture was warmed to RT and stirred for 16 h affording a light-yellow solution. The solution was diluted in EtOAc (20 mL), filtered and concentrated *in vacuo* to afford a light-yellow oil. The oil was purified by column chromatography eluting with a graduated solvent system of 100% hexane to 100% EtOAc to afford a colourless oil containing **9** (0.237 g, 0.68 mmols, 74%); Rf = 0.73 (20 % EtOAc in Hexane). **^{1}H NMR** (400 MHz, $CDCl_3$): δ_H 8.14 (s, 1H, CHO), 7.40-7.29 (m, 5H, HAr), 5.22 (m, 1H, H-3), 4.81-4.75 (m, 2H, 1H, CH_2), 4.56 (d, J_{CH2} = 11.6 Hz, CH_2), 4.16 (td, $J_{4,5}$ = 7.7, $J_{5,6}$ = 6.3 Hz, 1H, H-5), 3.92 (dd, $J_{2,3}$ = 5.0 Hz, $J_{1,2}$ = 3.1 Hz, 1H, H-2), 3.55 (dd, $J_{4,5}$ = 7.7 Hz, $J_{3,4}$ = 4.0 Hz, 1H, H-4), 1.34 (d, $J_{5,6}$ = 6.3 Hz, 3H, CH_3). **^{13}C NMR** (101 MHz, $CDCl_3$): δ_C 160.0 (C=O), 136.9, 128.7, 128.2, 127.9 (CAr), 96.9 (C-1), 70.0 (CH_2), 69.2 (C-3), 65.3 (C-5), 60.4 (C-2), 59.8 (C-4), 18.1 (CH_3). **(ESI)HRMS** – $C_{14}H_{16}N_6NaO_4^+$ ($[M+Na]^+$) requires m/z 355.1131: found m/z 355.1110. **FT-IR (ATR)** – 3033.24 (C-H), 2980.74 (C-H), 2936.14 (C-H), 2100.89 (N_3), 1725.84 (C=O), 1260.77 (C-O), 1137.87 (C-O)

Synthesis of benzyl 3-*O*-acetyl-2,4-diazido-2,4,6-trideoxy-α-L-altropyranoside (10)[4]

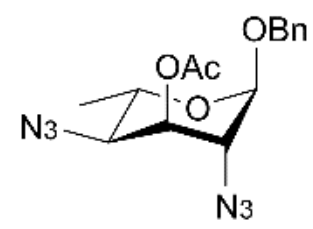

Ac_2O (2 mL) was added dropwise to a solution of **8** (0.489 g, 1.6 mmols, 1 eq), in dry pyridine (4 mL) at 0 °C under nitrogen. The reaction mixture was stirred at RT for 5 h affording a light-yellow solution. The solution was concentrated *in vacuo*, dissolved in DCM (10 mL), washed with water (10 mL), dried ($MgSO_4$) and purified using column chromatography eluting with a graduated solvent system of 100% hexane to 100% EtOAc affording a colourless oil containing mainly **10** (0.37 g, 1.06 mmols, 67%); Rf = 0.87 (20 % EtOAc in Hexane). **^{1}H NMR** (400 MHz, $CDCl_3$): δ_H 7.39-7.29 (m, 5H, HAr), 5.06 (dd, $J_{2,3}$ = 6.0 Hz, $J_{3,4}$ = 3.8 Hz, 1H, H-3), 4.80-4.75 (m, 2H, H-1, CH_2), 4.53 (d, J_{CH2} = 11.8 Hz, 1H, CH_2), 4.15 (dq, $J_{4,5}$ = 8.0 Hz, $J_{5,6}$ = 6.6 Hz, 1H, H-5), 3.92 (dd, $J_{2,3}$ = 6.0 Hz, $J_{1,2}$ = 3.2 Hz, 1H, H-2), 3.49 (dd, $J_{4,5}$ = 8.0 Hz, $J_{3,4}$ = 3.8 Hz, 1H, H-4), 2.10 (s, 3H, CH_3 (OAc)), 1.33 (d, $J_{5,6}$ = 6.6 Hz, 3H, H-6). **^{13}C NMR** (101 MHz, $CDCl_3$): δ_C 170.3 (C=O), 137.1, 128.6, 128.1, 127.8 (CAr), 97.3 (C-1), 70.1 (CH_2), 70.0 (C-3), 65.5 (C-5), 60.4 (C-4), 59.8 (C-2), 20.9 (CH_3), 18.2 (C-6). **(ESI)HRMS** – $C_{15}H_{18}N_6NaO_4^+$ ($[M+Na]^+$) requires m/z 369.1287: found m/z 369.1273. **FT-IR (ATR)** – 2927.41 (C-H), 2105.52 (N_3), 1750.33 (C=O), 1226.42 (C-O), 1046.02 (C-O)

Synthesis of benzyl 3-O-[(*R*)-3-(benzyloxy)butanoyl]-2,4-diazido-2,4,6-trideoxy-α-L-altropyranoside (11)

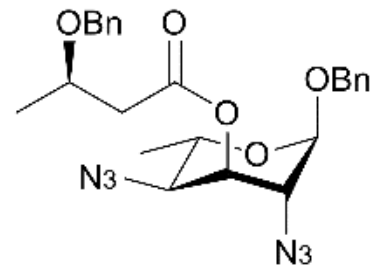

A crude solution of benzyloxybutanoic anhydride (0.79 g, approx. 2.15 mmols, 1.5 eq) was added to a solution of **8** (0.43 g, 1.43 mmols, 1 eq) and DMAP (cat) in dry Pyridine (7.5 mL) at 0 °C. The reaction mixture was stirred for 16 h at RT affording a colourless solution. The solution was concentrated *in vacuo* affording a crude oil which was dissolved in DCM (20 mL) affording a colourless solution. The solution was washed 1 x 1M HCl (20 mL), 1 x $NaHCO_3$ (20 mL) and 1 x NaCl (20 mL), dried ($MgSO_4$) and concentrated *in vacuo* affording a colourless oil. The oil was purified by column chromatography eluting with a graduated solvent system of 100% Hexane to 100% EtOAc affording a colourless oil containing **11** (0.5 g, 1.04 mmols, 73%); R_f = 0.73 (20% EtOAc in Hexane). **^{1}H NMR** (400 MHz, $CDCl_3$): δ_H 7.35-7.25 (m, 10H, H-Ar), 5.11 (dd, $J_{2,3}$ = 5.2 Hz, $J_{3,4}$ = 3.5 Hz, 1H, H-3), 4.76 – 4.72 (m, 2H, H-1, CH_2), 4.51-4.39 (m, 3H, CH2), 4.14 (m, 1H, H-5), 3.99-3.92 (m, 1H, CH), 3.81 (dd, $J_{2,3}$ = 5.2 Hz, J1,2 = 1.0 Hz, 1H, H-2), 3.45 (dd, $J_{4,5}$ = 8.3 Hz, $J_{3,4}$ = 3.5 Hz, 1H, H-4), 2.69 (dd, J_{CH2} = 15.2 Hz, J_{CH} = 7.5 Hz, 1H, CH_2), 2.44 (dd, J_{CH2} = 15.2 Hz, J_{CH} = 5.6 Hz, 1H, CH_2), 1.32 (d, $J_{5,6}$ = 6.5 Hz, 3H, CH_3 (H-6)), 1.19 (d, J_{CH} = 6.2 Hz, 3H, CH_3). **^{13}C NMR** (101 MHz, $CDCl_3$): δ_C 170.6 (C=O), 138.6, 136.9, 128.6, 128.4, 128.2, 127.6 (CAr), 97.1 (C-1), 71.8 (CH), 70.8 (CH_2), 70.1 (CH_2), 69.6 (C-3), 65.2 (C-5), 60.4 (C-4), 59.8 (C-2), 41.9 (CH_2), 19.9 (CH_3), 18.1 (CH_3). **(ESI)HRMS** – $C_{24}H_{28}N_6NaO_5^+$ ($[M+Na]^+$) requires m/z 503.2019: found m/z 503.2014**. FT-IR (ATR)** – 3032.09 (C-H), 2977.25 (C-H), 2933.27 (C-H), 2103.99 (N_3), 1744.17 (C=O), 1136.04 (C-O), 1077.04 (C-O)

Synthesis of benzyl 2-[(*R*)-3-(benzyloxy)butanamido]-2,4,6-trideoxy-4-formamido-α-L-altropyranoside (12)

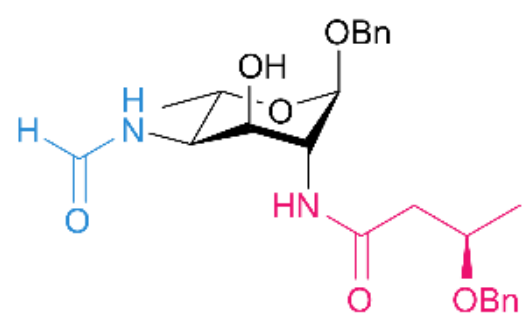

PPh_3 (4.2 g, 16.2 mmols, 1 eq) was added to a solution of **9** (2.7 g, 8.1 mmols, 1 eq) in 10:1 THF : Water (22 mL). Reaction mixture was refluxed for 16 h affording a clear solution, which was concentrated *in vacuo* to give a colourless oil which was dried *in vacuo* for 3.5 h. Benzyloxybutanoic anhydride **S10** was added dropwise to a solution of the crude oil in Pyridine (11 mL) on ice, to which DMAP was subsequently added (cat) and the reaction mixture was warmed to RT and stirred for 16h. Reaction mixture was concentrated *in vacuo* and purified by multiple rounds of column chromatography eluting with a graduated solvent system of 100% DCM to 10% MeOH in DCM affording a white solid of **12** (1.5g, 3.3 mmols, 41%). **^{1}H NMR** (400 MHz, MeOD): δ_H 8.08 (s, 1H, CHO), 7.35-7.22 (m, 10H, HAr), 4.68

(s, 1H, H-1), 4.67-4.64 (d, J_{CH2} = 12.3 Hz, 1H, CH_2), 4.55-4.52 (d, J_{CH2} = 12.3 Hz, 1H, CH_2), 4.50-4.47 (d, J_{CH2} = 12.1 Hz, 1H, CH_2), 4.41-4.38 (d, J_{CH2} = 12.1 Hz, 1H, CH_2), 4.18 (aS, 1H, H-2), 4.03-3.92 (m, 3H, CH, H-4, H-5), 3.68 (aS, 1H, H-3), 2.55-2.48 (dd, *J* = 13.5 Hz, 8.1 Hz, 1H, CH_2), 2.38-2.31 (dd, *J* = 13.5 Hz, 4.7 Hz, 1H, CH_2), 1.21 (d, *J* = 5.9 Hz, 3H, CH_3), 1.14 (d, *J* = 6.1 Hz, 3H, CH_3). **^{13}C NMR** (101 MHz, MeOD): δ_C 173.5 (C=O), 163.6 (C=O), 139.9, 138.6, 129.5, 129.3, 129.1, 128.9, 128.8, 128.7, 128.4 (CAr), 99.3 (C-1), 74.1 (C-4/ C-5/ CH), 71.8 (CH_2), 70.5 (CH_2), 69.5 (C-3), 64.1 (C-4/ C-5/ CH), 53.1 (C-2), 50.5 (C-4/ C-5/ CH), 44.3 (CH_2), 20.1 (CH_3), 18.2 (CH_3). **(ESI)HRMS** – $C_{25}H_{32}N_2NaO_6^+$ ($[M+Na]^+$) requires m/z 479.2158: found m/z 479.2158. **FT-IR (ATR)** – 3300.17 (O-H), 3032.03 (N-H), 2974.76 (C-H), 2932.27 (C-H), 1658.21 (C=O), 1535.77 (C=C), 1089.56 (C-O), 1063.56 (C-O). ***[α]D^{25}*** = -125.1 (*c* 1, MeOH)

Synthesis of benzyl 2-acetamido-2,4,6-trideoxy-4-formamido-α-L-altropyranoside (13)[4]

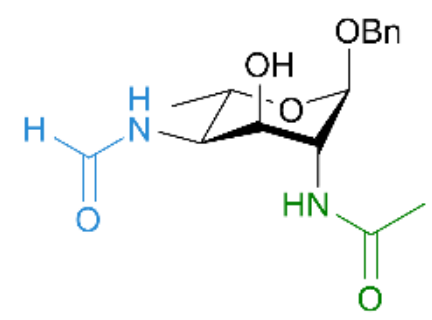

PPh_3 (1.43, 5.45 mmols, 2 eq) was added to a solution of **9** (0.9055 g, 2.73 mmols, 1 eq) in 10 : 1 THF : H_2O (11 mL). The reaction mixture was heated to reflux and stirred for 16 h affording a colourless solution. The solution was concentrated *in vacuo* and dried under vacuum for 3 h affording a crude colourless oil. The oil was dissolved in MeOH (10 mL) and Ac_2O (5 mL) was added dropwise to the solution at 0 °C followed by DMAP (cat), the reaction mixture was warmed to RT and stirred for 3 h affording a light-yellow solution. The solution was concentrated *in vacuo* to afford a yellow oil which was purified by column chromatography eluting with a graduated solvent system of 100% DCM to 10% MeOH in DCM affording a crude oil of **13** (0.489 g, 1.5 mmols, 56%); Rf = 0.51 (5% DCM in MeOH). **^{1}H NMR** (400 MHz, $CDCl_3$): δ_H 8.18 (s, 1H, CHO), 7.37-7.28 (m, 5H, HAr), 4.80 (s, 1H, H-1), 4.69 (d, J_{CH2} = 11.7 Hz, CH_2), 4.53 (d, J_{CH2} = 11.7 Hz, CH_2), 4.40 (m, 1H, H-2), 4.16 (m, 1H, H-4), 3.85 (m, 1H, H-5) 3.73 (m, 1H, H-3), 1.97 (s, 3H, CH_3), 1.24 (d, $J_{5,6}$ = 6.3 Hz, 3H, H-6). **^{13}C NMR** (101 MHz, $CDCl_3$): δ_C 170.2 (C=O), 161.4 (C=O), 136.2, 128.8, 128.5 128.3 (CAr), 98.4 (C-1), 70.2 (CH2), 69.1 (C-3), 63.8 (C-5), 49.9 (C-2), 48.6 (C-4), 23.2 (CH_3), 18.0 (C-6). **(ESI)HRMS** – $C_{16}H_{22}N_2NaO_5^+$ ($[M+Na]^+$) requires m/z 345.1426: found m/z 345.1423. **FT-IR (ATR)** - 3488.4 (N-H), 3283.74 (C-O), 3033.25 (C-H), 2876.60 (C-H), 1655.26 (C=O), 1534.91 (C=C), 1384.66 (C-O). ***[α]D^{25}*** = -109.28 (*c* 1, MeOH)

Synthesis of benzyl 2,4-diacetamido-2,4,6-trideoxy-α-L-altropyranoside (14)[4]

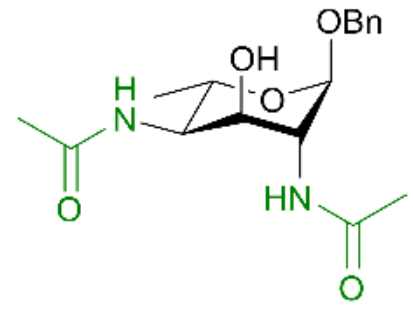

PPh_3 (0.68 g, 2.6 mmols, 2 eq) was added to a solution of **10** (0.455 g, 1.3 mmols, 1 eq) in 10 : 1 THF : H_2O (4.5 mL). The reaction mixture was refluxed for 24 h affording a colourless solution. The solution was concentrated *in vacuo* affording a crude solid. Ac_2O (9.6 mL) was added dropwise to a solution of the crude solid in MeOH (18 mL) on ice affording a colourless solution. The solution was warmed to RT and stirred for 16 h affording a light-yellow solution. The solution was concentrated *in vacuo* and purified by column chromatography eluting with a graduated solvent system of 100% DCM to 10% MeOH in DCM affording a colourless oil of **14** (0.35 g, 1.05 mmols, 81%); Rf = 0.6 (5% MeOH in DCM). **^{1}H NMR** (400 MHz, $CDCl_3$): δ_H 7.39-7.29 (m, 5H, HAr), 6.61 (d, $J_{2,NH}$ = 7.7 Hz, 1H NHAc (H-2)), 6.30 (d, J_{4NHAc} = 7.9 Hz, 1H, NHAc (H-4)), 4.80 (bS, 1H, H-1), 4.70 (d, J_{CH2} = 11.8 Hz, CH_2), 4.53 (d, J_{CH2} = 11.8 Hz, 1H CH_2), 4.40 (m, 1H, H-2), 4.02 (m, 1H, H-4), 3.86 (m, 2H, H-5, OH), 3.74 (m, 1H, H-3), 1.98 (s, 6H, CH_3 (OAc)), 1.24 (d, $J_{5,6}$ = 6.3 Hz, 3H, H-6). **^{13}C NMR** (101 MHz, $CDCl_3$): δ_C 170.2 (1C, C=O), 169.2 (1C, C=O), 136.2, 128.7, 128.4, 128.1 (CAr), 98.3 (1C, C-1), 70.0 (1C, CH_2) 69.2 (1C, C-3), 63.9 (1C, C-5), 50.2 (1C, C-4), 49.8 (1C, C-2), 23.4 (1C, CH_3 (OAc)), 23.1 (1C, CH_3 (OAc)), 17.9 (1C, C-6). **(ESI)HRMS** – $C_{17}H_{24}N_2NaO_5^+$ ($[M+Na]^+$) requires m/z 359.1583: found m/z 359.1572. **FT-IR (ATR)** – 3280.05 (O-H), 3064.47 (N-H), 2979.67 (C-H), 2933.53 (C-H), 1650.75 (C=O), 1539.92 (C=C), 1373.36 (C-O), 1019.60 (C-O). ***[α]D^{25}*** = -65.3 (*c* 1, MeOH)

Synthesis of benzyl 2-acetamido-4-[(*R*)-3-(benzyloxy)butanamido]-2,4,6-trideoxy-α-L-altropyranoside (15)

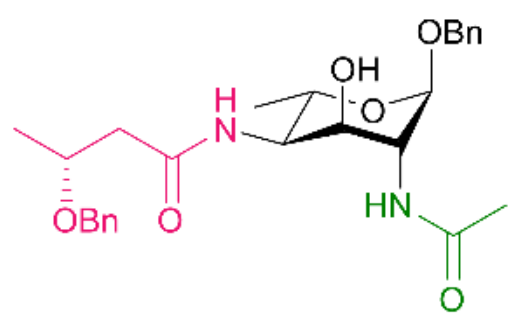

PPh_3 (2.62 g, 10 mmols, 2 eq) was added to a solution of **11** (2.4 g, 5 mmols, 1eq) in 10 : 1 THF : H_2O (25 mL) the resulting solution was heated to reflux and stirred for 16 h affording a light-yellow solution. The solution was concentrated *in vacuo* and dried under vacuum for 3 hours affording a colourless oil. The oil was dissolved in MeOH (20 mL) and placed on ice, DMAP (cat) was added to the solution followed by Ac_2O (10 mL) dropwise affording a light-yellow solution. the solution was stirred at RT for 3 h. The solution was concentrated *in vacuo* and purified by column chromatography eluting with a graduated solvent system of 100% DCM to 10% MeOH in DCM to afford a colourless crude oil of **X** and the doubly acetylated product. The oil was dissolved in MeOH (25 mL) and NaOMe (0.453 g) was added at 0 °C and the reaction mixture was stirred at RT for 16h affording a cloudy white solution. The

solution was diluted with MeOH (50 mL) and neutralised with amberlite (H^+ form) and the solution was concentrated in vacuo to give a colourless oil. The oil was purified by column chromatography eluting with a graduated solvent system of 100% DCM to 10% MeOH in DCM affording a colourless foam of **15** (0.9613 g, 2.04 mmols, 41%); R_f = 0.47 (5% MeOH in DCM). **^{1}H NMR** (400 MHz, MeOD): δ_H 7.37-7.23 (m, 10H, HAr), 4.71 (aS, 1H, H-1), 4.66 (d, J_{CH2} = 12.2 Hz, 1H, CH_2 (OBn)), 4.57 (d, J_{CH2} = 11.2 Hz, 1H, CH_2 (OBn)), 4.51 (d, J_{CH2} = 12.2 Hz, 1H, CH_2 (OBn)), 4.42 (d, d, J_{CH2} = 11.2 Hz, 1H, CH_2 (OBn)), 4.12 (aD, J = 1.6 Hz,1H, H-2), 4.01-3.95 (m, 1H, CH), 3.95-3.91 (m, 2H, H-4, H-5), 3.65 (aS, 1H, H-3), 2.51-2.44 (dd, J = 14.1 Hz, J = 8.0Hz, 1H, CH_2), 2.37-2.31 (dd, J = 14.1 Hz, J = 4.6 Hz, 1H, CH_2), 1.94 (s, 3H, CH_3 (OAc)), 1.22 (d, J = 6.4 Hz, 3H, CH_3), 1.08 (d, J = 5.6 Hz, 3H, H-6). **^{13}C NMR** (101 MHz, MeOD): δ_C 173.6 (C=O), 173.0 (C=O), 139.9, 138.7, 129.5, 129.3, 128.9, 128.6 (CAr), 99.4 (C-1), 73.9 (CH), 71.8 (CH_2 (OBn)), 70.6 (CH_2 (OBn)), 69.5 (C-3), 64.2 (C-4 or C-5 (likely C-5)), 53.2 (C-2), 51.8 (C-4 or C-5 (likely C-4)), 44.8 (CH_2), 22.4 (CH_3 (OMe)), 19.9 (CH_3), 18.3 (C-6). **(ESI)HRMS** – $C_{26}H_{34}N_2NaO_6^+$ ([M+Na]$^+$) requires m/z 493.2315: found m/z 493.2316. **FT-IR (ATR)** - 3287.76 (O-H), 3064.00 (N-H), 2974.86 (C-H), 2932.90 (C-H), 1650.14 (C=O), 1543.83 (C=C), 1376.72 (C-O), 1132.48 (C-O), 1077.09 (C-O). ***[α]D^{25}*** = -114.8 (*c* 1, MeOH)

Synthesis of 2,4,6-trideoxy-4-formamido-2-[(*R*)-3-hydroxybutanamido]-α-L-altropyranose (16)

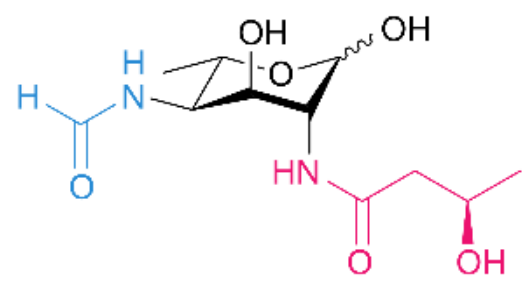

Pd(OH)$_2$/C (cat) and AcOH (47 µL) was added to a solution of **12** (0.156 g, 0.34 mmols) in 10 : 1 MeOH DCM (10 mL : 1 mL). The reaction was flushed with N_2 and backfilled with H_2 affording a black solution. The solution was stirred at RT for 16 h at which point TLC analysis observed a mixture of products, the wanted product and a substantial amount of a single benzyl protected sugar. The solution was filtered through a 0.2-micron syringe filtered and concentrated *in vacuo* and dried *in vacuo* for an hour. The reaction was then reset up as described above and stirred at RT for 16 h. After this point TLC analysis indicated that the reaction was complete. The black solution was filtered through a 0.2-micron syringe filter and the resulting colourless solution was concentrated *in vacuo* affording a crude colourless oil. This oil was purified by column chromatography eluting with a graduated solvent system of 100% DCM to 20% MeOH in DCM affording a colourless oil of **16** (57.5 mg, 0.208 mmols, 61%); Rf = 0.28 (10% MeOH in DCM). **^{1}H NMR** (400 MHz, MeOD): δ_H 8.09 (s, 1H, CHO), 5.20 (d, $J_{1,2}$ = 2.1 Hz, 1H, H-1), 4.15 (m, 1H, CH), 4.06 (m, 1H, H-2), 3.92 (dd, J = 10.5 Hz, J = 3.0 Hz, 1H, H-4), 3.82 (m, 2H, H-3, H-5), 2.43 (m, 2H, CH_2), 1.20 (m, 6H, 2x CH_3). **^{13}C NMR** (101 MHz,

MeOD): δ_C 174.8 (1C, C=O), 163.7 (1C, C=O), 92.3 (1C, C-1), 70.8 (1C, C-5/ C-3), 69.9 (1C, C-5/ C-3), 66.0 (1C, CH), 55.5 (1C, C-2), 50.6 (1C, C-4), 45.9 (1C, CH_2), 23.3 (1C, CH_3), 18.6 (1C, CH_3). **(ESI)HRMS** – $C_{11}H_{20}N_2NaO_6^+$ ([M+Na]$^+$) requires m/z 299.1219: found m/z 299.1216. **FT-IR (ATR)** – 3291.51 (O-H), 2932.75 (C-H), 2975.19 (C-H), 1657.00 (C=O), 1650.00 (C=O), 1645.00 (C=O), 1078.32 (C-O). ***[α]D²⁵*** = -72.46 (*c* 0.75, MeOH)

Synthesis of 2-acetamido-2,4,6-trideoxy-4-formamido-α-L-altropyranose (17)

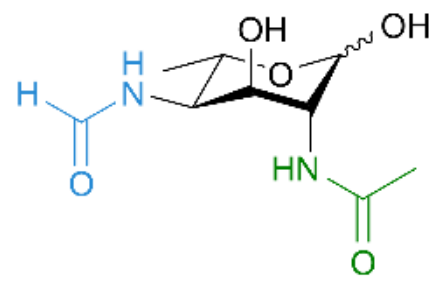

Pd/C (Cat) was added to a solution of **13** (0.13 g, 0.4 mmols, 1 eq) in a 2 : 1 MeOH : EtOAc solution (15 mL), The reaction was flushed with N2 and backfilled with H2 affording a black solution. The solution was stirred at RT for 40 h and the resulting solution was double filtered, concentrated in vacuo and purified by column chromatography eluting with a graduated solvent system of 100% DCM to 10% MeOH in DCM affording a colourless oil of **17** (0.050 g, 0.28 mmols, 53%); Rf = 0.32 (10% MeOH in DCM). **^{1}H NMR** (400 MHz, CDCl3): δH 8.08 (s, 1H, COH), 5.24 (d, J1,2 = 1.8 Hz, 1H, H-1), 4.06 (m, 1H, H-2), 3.92-3.84 (m, 3H, H-3, H-4, H-5), 2.05 (s, 3H, CH3), 1.18 (m, 3H, H-6). **^{13}C NMR** (101 MHz, CDCl3): δC 174.9 (C=O), 164.2 (C=O), 90.6, 69.5, 68.1, 53.5, 49.2, 21.9, 17.2. **(ESI)HRMS** – C9H16N2NaO5+ ([M+Na]+) requires m/z 255.0957: found m/z 255.0947. **FT-IR (ATR)-** 3276.62 (O-H), 2979.38 (C-H), 2917.85 (C-H), 1639.73 (C=O), 1538.00 (C=O), 1375.61 (C-O), 1070.87 (C-O). ***[α]D²⁵*** = -52.88 (c 0.5,

Synthesis of 2,4-diacetamido-2,4,6-trideoxy-α-L-altropyranose (18)[2]

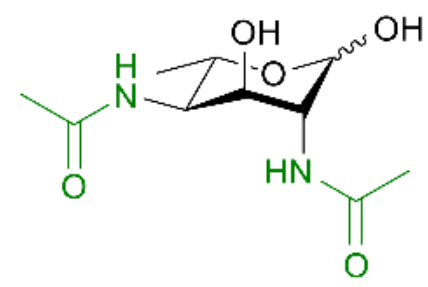

Pd(OH)$_2$/C (cat) was added to a solution of **14** (0.797, 2.37 mmols) and AcOH (160 µL) in a 5 : 1 mixture of MeOH : DCM (16 mL : 3.2 mL). The reaction was flushed with N_2 and backfilled with H_2 affording a black solution. The solution was stirred at RT for 6 h and the resulting solution was filtered through a 0.2 microm syringe filter affording a colourless solution. The solution was concentrated *in vacuo* affording a colourless foam of **18** (0.4692 g, 1.9 mmols, 80%); Rf = 0.4 (10 % MeOH in DCM). **^{1}H NMR** (400 MHz, D_2O): δ_H 5.23 (d, *J* = 1.8 Hz, 1H, H-1), 4.06 (dd, *J* = 3.3 Hz, *J* = 2.2 Hz, 1H, H-2), 3.93-3.85 (m, 2H, H-3, H-5), 3.78 (dd, *J* = 10.4 Hz, *J* = 2.8 Hz, 1H, H-4), 2.05 (s, 3H, CH_3), 1.99 (s, 3H, CH_3), 1.17 (d, *J* = 6.2 Hz, 3H, H-6). **^{13}C NMR** (101 MHz, D_2O): δ_C 174.9, 174.1 (C=O), 90.6 (C-1), 69.4 (C-3), 68.1 (C-5), 53.5 (C-2), 50.7 (C-4), 21.9 (CH_3), 17.2 (CH_3)**.** **(ESI)HRMS** – $C_{10}H_{18}N_2NaO_5^+$ ([M+Na]$^+$) requires m/z 269.1113:

found m/z 269.1113. **FT-IR (ATR)** – 3288.55 (O-H), 3083.3 (N-H), 2987.2 (C-H), 2945.5 (C-H), 1636.26 (C=O), 1534.96 (C=O), 1373.25 (C-O), 1072.83 (C-O). ***[α]D^25^*** = -37.16 (*c* 1, Water)

Synthesis of 2-acetamido-2,4,6-trideoxy-4-[(*R*)-3-hydroxybutanamido]-α-L-altropyranose (19)

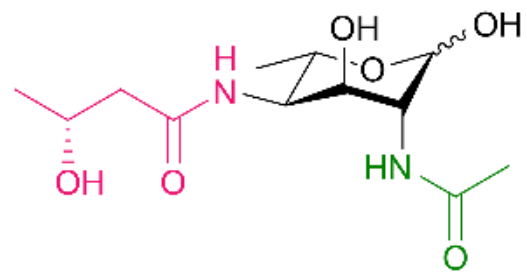

Pd/C (Cat) was added to a solution of **15** (0.5989 g, 1.27 mmols, 1 eq) in a 2 : 1 MeOH : EtOAc solution (30 mL), The reaction was flushed with N_2 and backfilled with H_2 affording a black solution. The solution was stirred at RT for 48 h and the resulting solution was double filtered, concentrated *in vacuo* and purified by column chromatography eluting with a graduated solvent system of 100% DCM to 10% MeOH in DCM affording a colourless oil of **19** (0.162 g, 0.56 mmols, 44%); Rf = 0.32 (10% DCM in MeOH). **^{1}H NMR** (400 MHz, D_2O): δ_H 5.24 (d, $J_{1,2}$ = 2.0 Hz, 1H, H-1), 4.15 (dd, $J_{CH,CH3}$ = 13.6 Hz, $J_{CH,CH2}$ = 6.7 Hz, 1H, CH), 4.06 (dd, $J_{2,3}$ = 2.8 Hz, $J_{1,2}$ = 2.0 Hz, 1H, H-2), 3.93-3.85 (m, 2H, H-4, H-5), 3.82 (dd, $J_{3,4}$ = 10.3, $J_{2,3}$ = 2.8 Hz, 1H, H-3), 2.40 (d, $J_{CH,CH2}$ = 6.7 Hz, 2H, CH_2), 2.05 (s, 3H, CH_3 (OAc)), 1.18 (m, 6H, 2x CH_3). **^{13}C NMR** (101 MHz, D_2O): δ_C 174.9 (C=O), 173.9 (C=O), 90.6 (C-1), 69.5 (C4/C5), 68.1 (C4/C5), 65.0 (CH_2), 53.6 (C-2), 50.2 (C-3), 44.8 (CH_2), 21.9 (CH_3), 21.9 (CH_3), 17.3 (CH_3). **(ESI)HRMS** – $C_{12}H_{22}N_2NaO_6^+$ ($[M+Na]^+$) requires m/z 313.1376: found m/z 313.1367. **FT-IR (ATR)** – 3283.66 (O-H), 2975.88 (C-H), 2934.82 (C-H), 1627.42 (C=O), 1537.68 (C=C), 1372.83, 1071.15 (C-O). ***[α]D^25^*** = - 79.39 (*c* 0.5, Water)

Enzymatic synthesis protocol for the Pse analogues

Synthesis of Pse5Ac7Ac (2)[5]

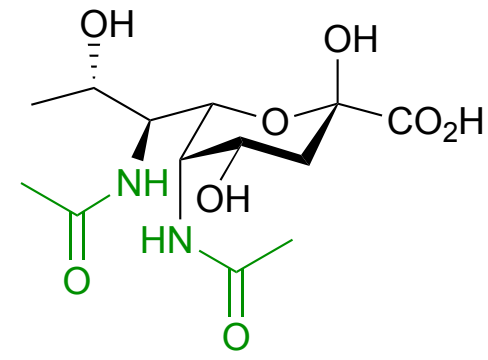

A 20 mL solution of 50 mM HEPES pH = 7.4 was charged with 2 mM **18**, 3 mM PEP, 20 mM $CoCl_2$ and 0.2 mg/mL PseI, the solution was aliquoted into 1 mL samples and incubated at 30 °C for 20 h. The samples were combined and the solutions pH was adjusted to pH = 8.0, the solution was charged with 2 mM $MgCl_2$ and 16.5 units of alkaline phosphatase (Sigma). The solution was aliquoted into 1 mL samples and incubated at 37 °C for 1 h, the reactions were terminated by the addition of 1 mL ice-cold ethanol, the samples were centrifuged at 14,800 rpm for 10 minutes. The supernatant was collected and the protein pellet was resuspended in 1 mL ice-cold ethanol. The samples were centrifuged at 14,800 xrpm and the supernatant was collected and combined with the previous, the ethanol content was diluted to below 10% and the sample was lyophilised to give a crude solid. The crude solid was purified using Bio-gel P2 resin (Biorad) running in HPLC grade water and lyophilised to afford Pse5Ac7Ac **2** (13 mg, 97%). Characterisation as previously reported.[5] **^{1}H NMR** (400 MHz, D_2O): δ_H 4.19 (d, *J* = 5.0 Hz, 1H, H-4), 4.15-4.04 (m, 3H, H-5, H-7, H-8), 3.97 (d, *J* = 10.6 Hz, 1H, H-6), 1.97 (s, 3H, CH_3), 1.93 (s, 3H, CH_3), 1.88 (dd, *J* = 13.0 Hz, 5.7 Hz, 1H, H3eq), 1.73 (t, J = 13.0 Hz, 1H, H-3ax), 1.05 (d, *J* = 6.4 Hz, 3H, H-9). **(ESI)HRMS** – $C_{13}H_{21}N_2O_8^-$ ($[M-H]^-$) requires m/z 333.1303: found m/z 333.1301

Synthesis of Pse5Hb7Fm (20)

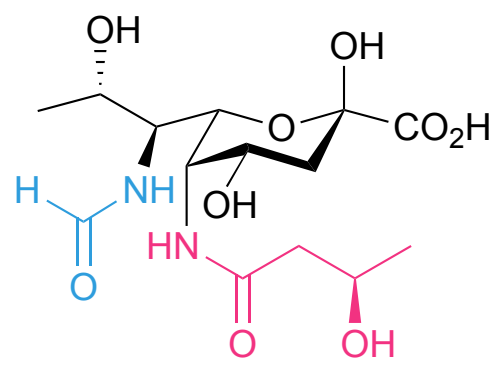

A solution of 20 mL of 50 mM HEPES buffer pH = 7.4 was charged with 2 mM glycoside **16**, 3 mM PEP, 2 mM $CoCl_2$ and 0.2 mg/mL PseI, the solution was aliquoted into 1 mL samples and incubated at 30 °C for 24 h. Analysis by LCMS showed that starting material remained, a further 0.2 mg/mL PseI was added in addition to 3 mM PEP and the sample was incubated for a further 24 h at 30°C. This process was repeated for a further 5 days. The samples were then combined and the pH was adjusted to pH = 8.2 and 2 mM $MgCl_2$ was added along with 17 units of alkaline phosphatase (from bovine intestinal mucosa, Merck PO114). The solution was aliquoted into 1 mL samples and incubated at 37 °C for 1 h, the reaction was terminated by the addition of 1 mL ice cold ethanol to each sample, the samples were centrifuged at 14,800 xrpm for 10 minutes at 4 °C, the supernatant was collected. The protein pellet was resuspended in 1 mL ice cold ethanol, the samples were centrifuged at 14,800 xrpm for 10 minutes at 4 °C and the supernatant was collected and combined with the previous, the ethanol content was diluted to below 10% and the sample was lyophilised. The sample was purified using Bio-gel P2 resin (Biorad) running in HPLC grade water and lyophilised, before further purification using a strong anion exchange column (HiTrap™ 5 mL QFF), eluting using a stepwise gradient of 1M ammonium acetate 5, 10, 15, 20, 30, 70, 100%, before lyophilisation of fractions to afford Pse5Hb7Fm **20** (7 mg, 42%). **^{1}H NMR** (700 MHz, D_2O): δ_H 8.10 (s, 1H, CHO), 4.35-4.30 (m, 1H, CH), 4.28-4.10 (m, 4H,

CH), 4.05 (dd, *J* = 10.6 Hz, *J* = 1.7 Hz, 1H, CH), 2.54-2.40 (m, 2H, CH_2), 1.93 (dd, *J* = 12.8 Hz, *J* = 5.0 Hz, 1H, H3eq), 1.80 (t, *J* = 12.8 Hz, 1H, H3ax), 1.26 (d, *J* = 6.3 Hz, 3H, CH_3), 1.11 (d, *J* = 6.6 Hz, 3H, CH_3). **^{13}C NMR** (176 MHz, D_2O): δ_c 176.3, 174.7, 164.0 (C=O), 96.5 (<u>C</u>), 69.9 (CH), 66.4 (CH), 65.4 (CH), 65.0 (CH), 51.5 (CH), 48.8 (CH), 44.8 (CH_2), 34.7 (CH_2), 22.0 (CH_3), 15.1 (CH_3). **(ESI)HRMS** – $C_{14}H_{23}N_2O_9^-$ ($[M\text{-}H]^-$) requires m/z363.1409: found m/z 363.1396

Synthesis of Pse5Ac7Fm (21)

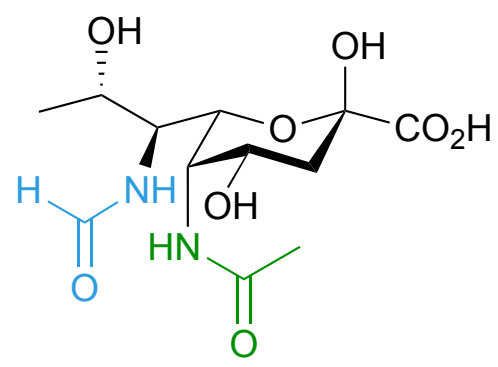

A solution of 50 mM HEPES pH = 7.4 (final volume 18.10 mL) was charged with 2 mM glycoside **17** (0.0362 mmols), 3 mM PEP, 2 mM $CoCl_2$ and 0.2 mg/mL cjPseI, the solution was aliquoted into 1 mL samples and incubated at 30 °C overnight (16 h total). Each sample was charged with a further 3 mM PEP, 2 mM $CoCl_2$ and 0.2 mg/mL cjPseI, the samples were incubated at 30 °C for a further 24 h (40 h total). At this point the formation of a precipitate was observed, thus the samples were spun at 14,800 rpm for 10 minutes at 4 °C and the samples were transferred to clean Eppendorfs. The samples were charged with a further 3 mM PEP, 2 mM $CoCl_2$ and 0.2 mg/mL cjPseI and the samples were incubated at 30 °C for a further 24 h (64 h total). The samples were combined and the pH was adjusted to pH = 8.0 and 12 units of alkaline phosphatase (from bovine intestinal mucosa, Merck PO114) was added along with 2 mM $MgCl_2$. The solution was aliquoted into 1 mL samples and incubated at 37 °C for 2 h. The reaction was terminated by the addition of 1 mL ice cold ethanol to each sample and the samples were spun at 14,800 rpm for 10 minutes at 4 °C. The supernatant was collected and the protein pellet was resuspended in 1 mL ice-cold ethanol, the sample was spun at 14,800 rpm for 10 minutes at 4 °C and the supernatant was removed and combined with the previous. The ethanol content was diluted to below 10% and the sample was lyophilised. The sample was purified using Bio-gel P2 resin (Biorad) running in HPLC grade water and lyophilised to afford Pse5Ac7Fm **21** (9 mg, 77%). **^{1}H NMR** (700 MHz, D_2O): δ_H 8.08 (s, 1H, CHO (H-10)), 4.25 (m, 2H, H-5/H-6/H-7), 4.16 (m, 2H, H-4, H-8), 4.03 (d, *J* = 10.6 Hz, 1H, H-5/H-6/H-7), 2.01 (s, 3H, CH_3 (NHAc)), 1.92 (dd, *J* = 13.0 Hz, 5.7 Hz, 1H, H-3eq), 1.78 (t, *J* = 13.0 Hz, H-3ax), 1.11 (d, *J* = 6.7 Hz, 1H, H-9). **^{13}C NMR** (176 MHz, D_2O): δ_c 176.37, 174.82, 163.99 (C=O), 96.46 (C), 69.96 (CH), 66.37 (CH), 65.26 (CH), 51.58 (CH), 48.81 (CH), 34.53 (CH_2), 21.95 (CH_3), 15.11 (CH_3). **(ESI)HRMS** – $C_{12}H_{19}N_2O_8^-$ ($[M\text{-}H]^-$) requires m/z 319.1147: found m/z 319.1145. **FT-IR (ATR)-** 3280.16 (O-H), 1649.94 (C=O), 1375.41 (C-O), 1064.88 (C-O). ***$[\alpha]_D^{25}$*** = -4.83 (*c* 0.267, Water)

Synthesis of Pse5Ac7Hb (22)

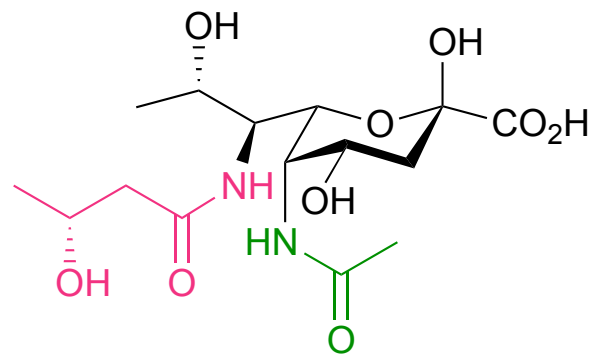

A solution of 50 mM HEPES pH = 7.4 (17.4 mL final volume) was charged with 2 mM glycoside **19** (0.0348 mmols), 3 mM PEP, 2 mM $CoCl_2$ and 0.2 mg/mL cjPseI, the solution was aliquoted into 1 mL samples and incubated at 37 °C for 24 h. The samples were combined and the pH was adjusted to pH = 8.2, the solution was charged with 1.5 units per mL FastAP Thermosensitive Alkaline Phosphatase (ThermoFisher Scientific; EF0654), 2 mM $MgCl_2$ and 2 mM $ZnCl_2$. The samples were aliquoted into 1 mL samples and then incubated at 37 °C for 2 h, the reactions were terminated by the addition of 1 mL ice-cold ethanol and the samples were centrifuged at 14,800 rpm for 10 minutes at 4 °C. The supernatant was collected and the protein pellet was resuspended in 1 mL ice cold ethanol, the samples were spun at 14,800 rpm for 10 minutes at 4 °C and the supernatant was collected and combined with the previous. The ethanol content was diluted to below 10% and the sample was lyophilised. The sample was purified using Bio-gel P2 resin (Biorad) running in HPLC grade water and lyophilised to afford Pse5Ac7Hb **22** (12 mg, 91%). **^{1}H NMR** (700 MHz, D_2O): δ_H 4.23 (dd, *J* = 3.6 Hz, 1H, CH (H-7)), 4.20-4.14 (m, 3H, 3x CH (H-4, CH, 1x(H-5/H-6))), 4.12 (dd, *J* = 6.2 Hz, *J* = 3.6 Hz, CH, H-8), 4.03 (dd, *J* = 10.2 Hz, *J* = 1.6 Hz, CH (H-5/H-6)), 2.41-2.36 (m, 2H, CH_2), 2.01 (s, 3H, CH_3), 1.92 (dd, *J* = 12.6 Hz, *J* = 3.9 Hz, 1H, CH_2 (H3eq)), 1.78 (t, *J* = 12.6 Hz, 1H, CH_2 (H-3 ax)), 1.24 (d, *J* = 6.3 Hz, 3H, CH_3), 1.10 (d, *J* = 6.3 Hz, 3H, CH_3 (H-9)). **^{13}C NMR** (176 MHz, D_2O): δ_c 176.39, 174.64, 173.73 (C=O), 96.46 (C), 69.95 (CH), 66.98 (CH), 65.28 (CH), 64.95 (CH), 52.95 (CH), 48.85 (CH), 45.13 (CH_2), 34.79 (CH_2), 22.09 (CH_3), 21.87 (CH_3), 15.43 (CH_3). **(ESI)HRMS** – $C_{15}H_{25}N_2O_9^-$ ([M-H]$^-$) requires m/z 377.1566: found m/z 377.1563. **FT-IR (ATR)-** 3279.94 (O-H), 2974.80 (C-H), 1627.01 (C=O), 1556.83 (C=O), 1375.66 (C-O), 1046.48 (C-O). ***[α]D^{25}*** = -26.88 (*c* 0.56, Water)

Production of biotinylated 1E8 Fab’

A plasmid encoding the 1E8 antibody reformatted into a mouse IgG2a (Genscript) was transfected and expressed in ExpiCHO cells following the manufacturer’s instructions (ThermoFisher). Seven days post-transfection, cells were removed by centrifugation at 1500 xg and supernatant was clarified using a 0.22 µm filter. 1E8 antibody was purified by affinity chromatography using protein A resin pre-equilibrated in TBS buffer (20 mM Tris pH 7.4, 150 mM NaCl). Resin was washed with 10 column volumes of TBS prior to elution of the IgG with 100 mM glycine pH 2.7. Elution fractions were equilibrated with 1M Tris pH 9.0 and further purified by size exclusion chromatography using the Superdex® 200 increase 10/300 GL column (GE Healthcare) pre-equilibrated in PBS buffer.

Purified 1E8 antibody was cleaved below the hinge region using IdeZ protease. Reactions were set up in PBS buffer and incubated at RT for 1h. 6xHis-tagged IdeZ and cleaved Fc were removed by reverse chromatography using Ni-NTA and protein A resin. Finally, purified 1E8 F(ab’)2 was treated with 50 mM 2-MEA in PBS-EDTA buffer (100mM sodium phosphate,150 mM NaCl, 10mM EDTA, pH 7.2) at RT for 20 min to generate 1E8 Fab’. 2-MEA was removed by buffer exchange into PBS buffer using a Zeba spin desalting column (ThermoFisher) prior to chemical biotinylation of the Fab’ with EZ-Link™ Maleimide-PEG2-Biotin following manufacturer’s recommendations (ThermoFisher). Biotinylated 1E8 Fab’ was further purified by size exclusion chromatography using the Superdex® 200 increase 10/300 GL column (GE Healthcare) pre-equilibrated in PBS buffer.

Surface plasmon resonance (SPR) methodology

Binding experiments were carried out using the Biacore S200 system (GE Healthcare) in 10 mM HEPES pH 7.4, 150 mM NaCl, 3mM EDTA, 0.02% Tween 20. Dilutions of Pse derivatives were passed over biotinylated 1E8 Fab’ immobilised onto a streptavidin-coated sensor chip (Citiva) for 60 sec at 30 µl/min. Dissociation of analytes was achieved with running buffer for 120 sec at 30 µl/min. A reference flow cell (no ligand) was included in the experiments, and the recorded data was subtracted from the experimental flow cells. Data was plotted in Prism (Graphpad).

LCMS analysis of the *cj*PseI mediated enzymatic synthesis of Pse derivatives

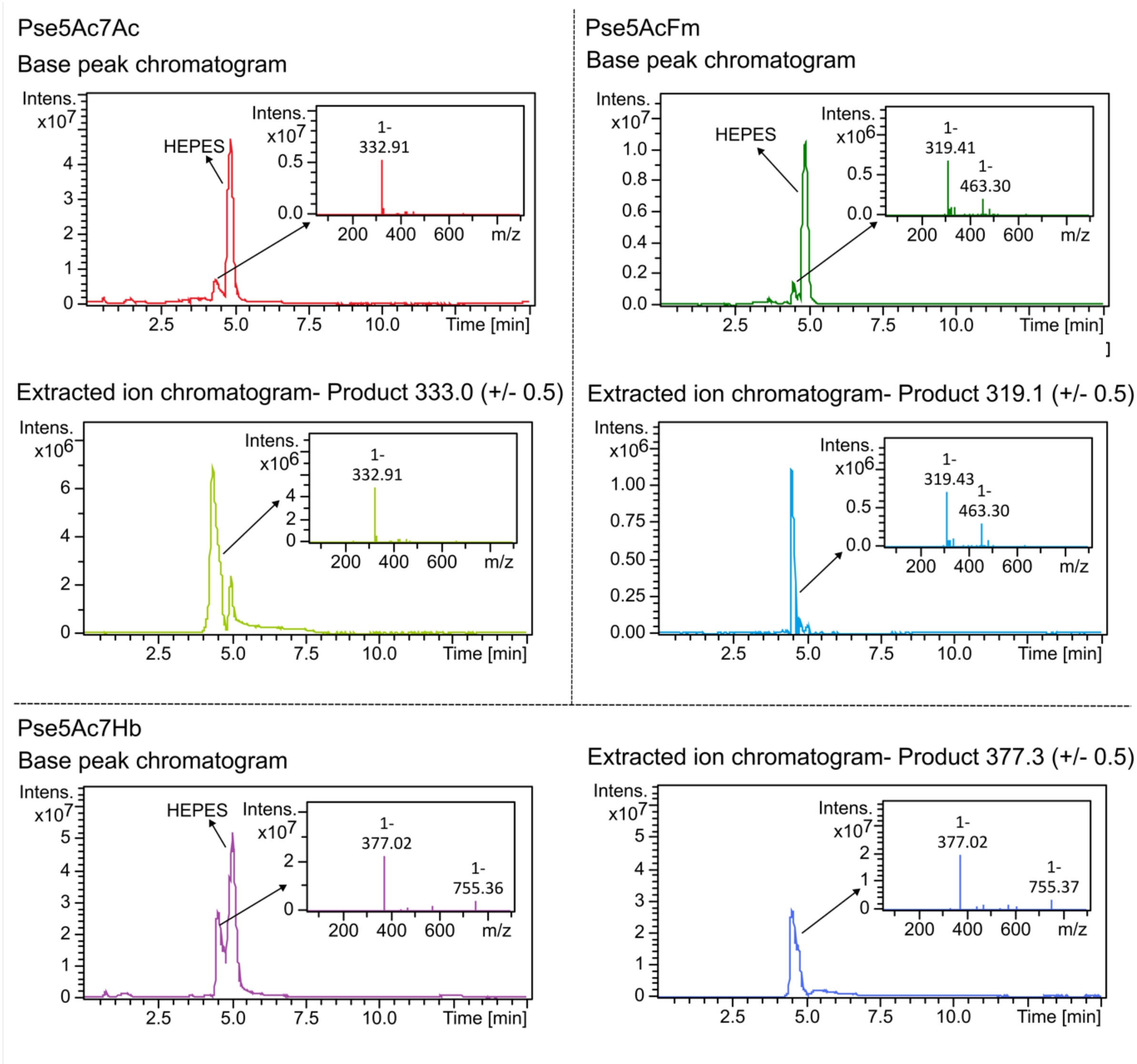

Figure S2. LCMS analysis of reaction *cj*PseI enzymatic reaction mixtures after 24 h for formation of Pse5Ac7Ac **2**, Pse5Ac7Fm **21**, Pse5Ac7Hb **22**, indicating no remaining starting material.

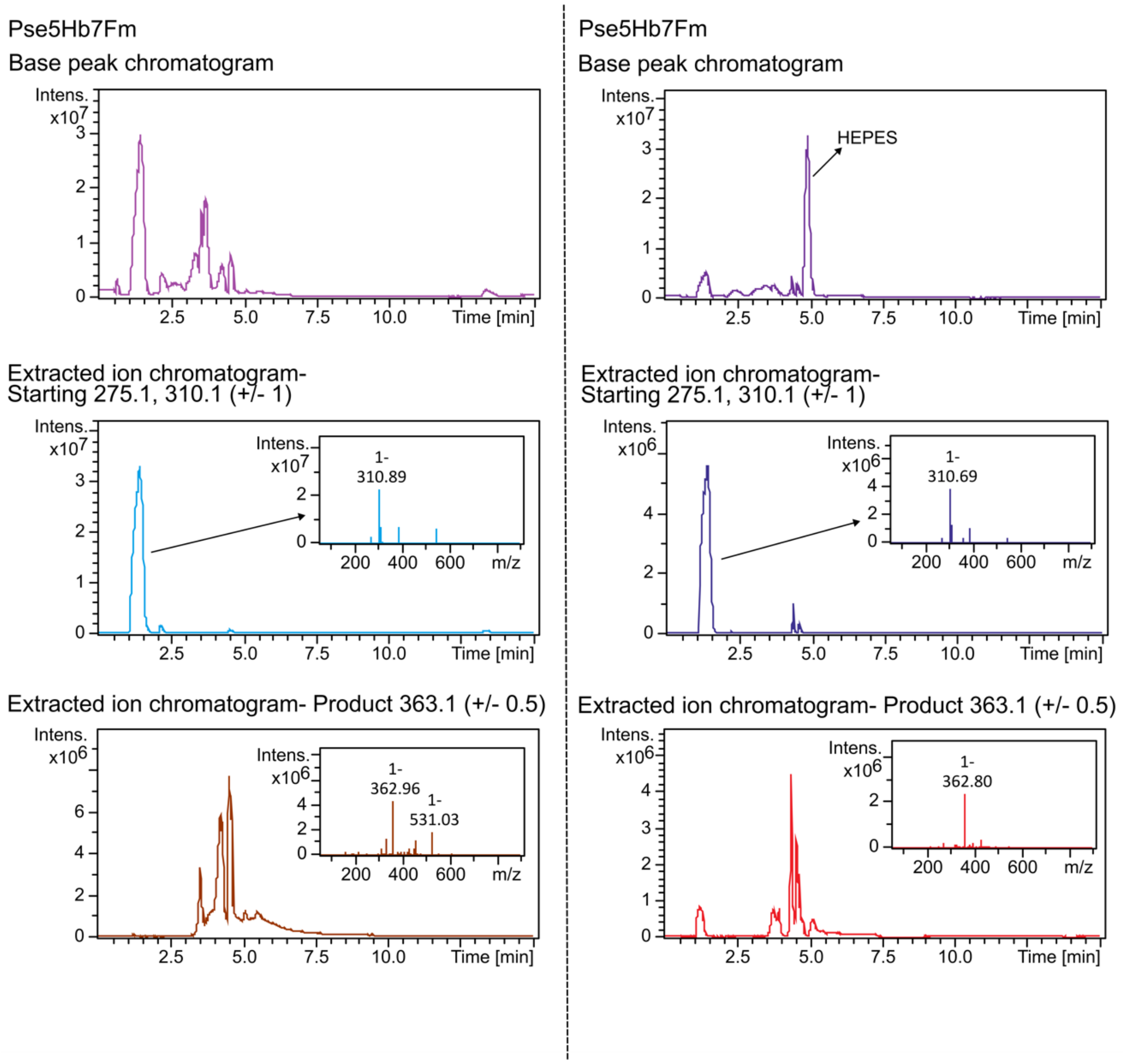

Figure S3. LCMS analysis of reaction *cj*PseI enzymatic reaction mixture after 24 h (left) and 7 days (right) for formation of Pse5Hb7Fm **20**, indicating starting material still remaining. Starting material **16** (m/z = 276) also present as a chloride adduct (m/z = 311) in negative ion mass spectrum.

NMR and mass spectrum data

Benzyl-α/β-L-fucopyranoside (S2)

Mass spectrum of Benzyl-α/β-L-fucopyranoside

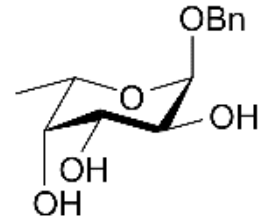

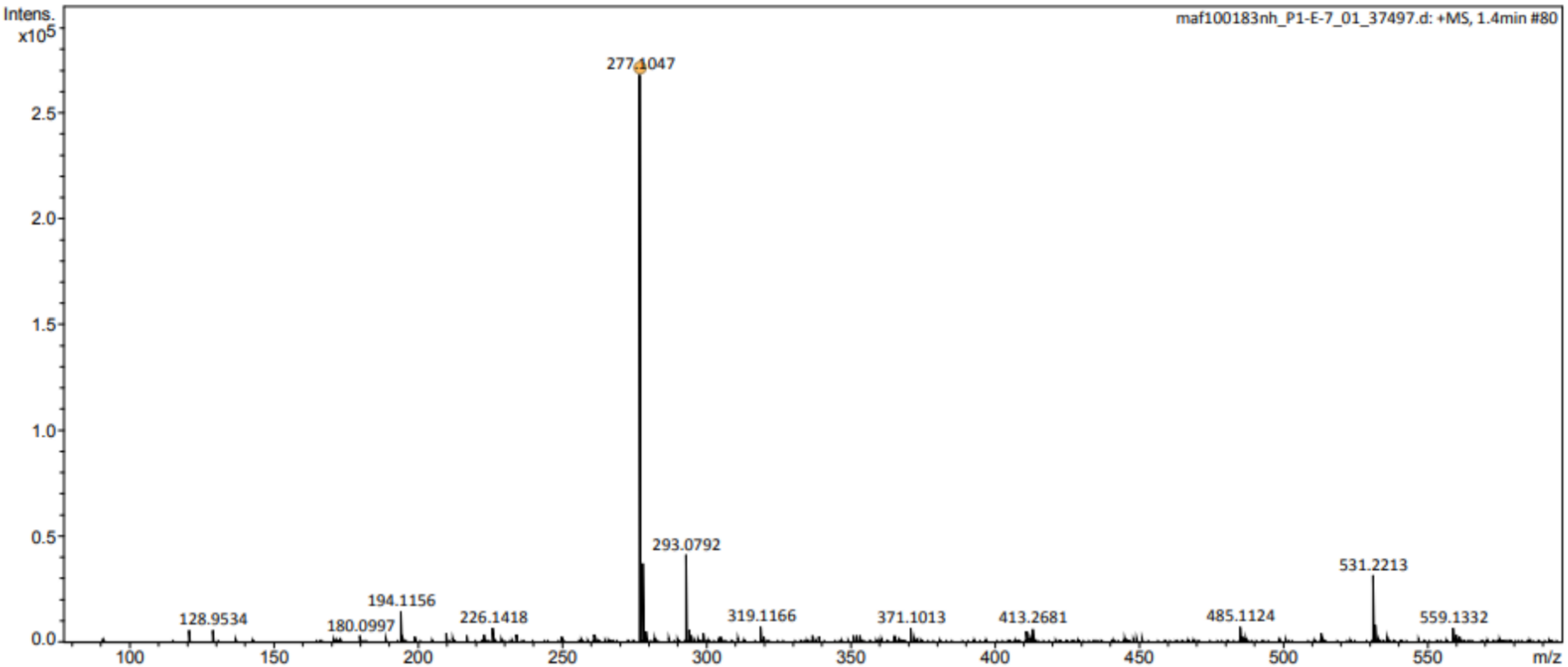

Meas. m/z	#	Ion Formula	m/z	err [ppm]	err [mDa]	mSigma	Mean err [ppm]
277.1047	1	C13H18NaO5	277.1046	-0.1	-0.0	2.8	-0.1

Benzyl 3,4-O-isopropylidene-α-L-fucopyranoside (**S3**)

*Hydrogen NMR of Benzyl 3,4-O-isopropylidene-α-L-fucopyranoside (**S3**)*

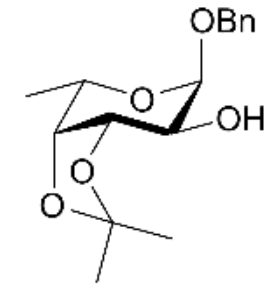

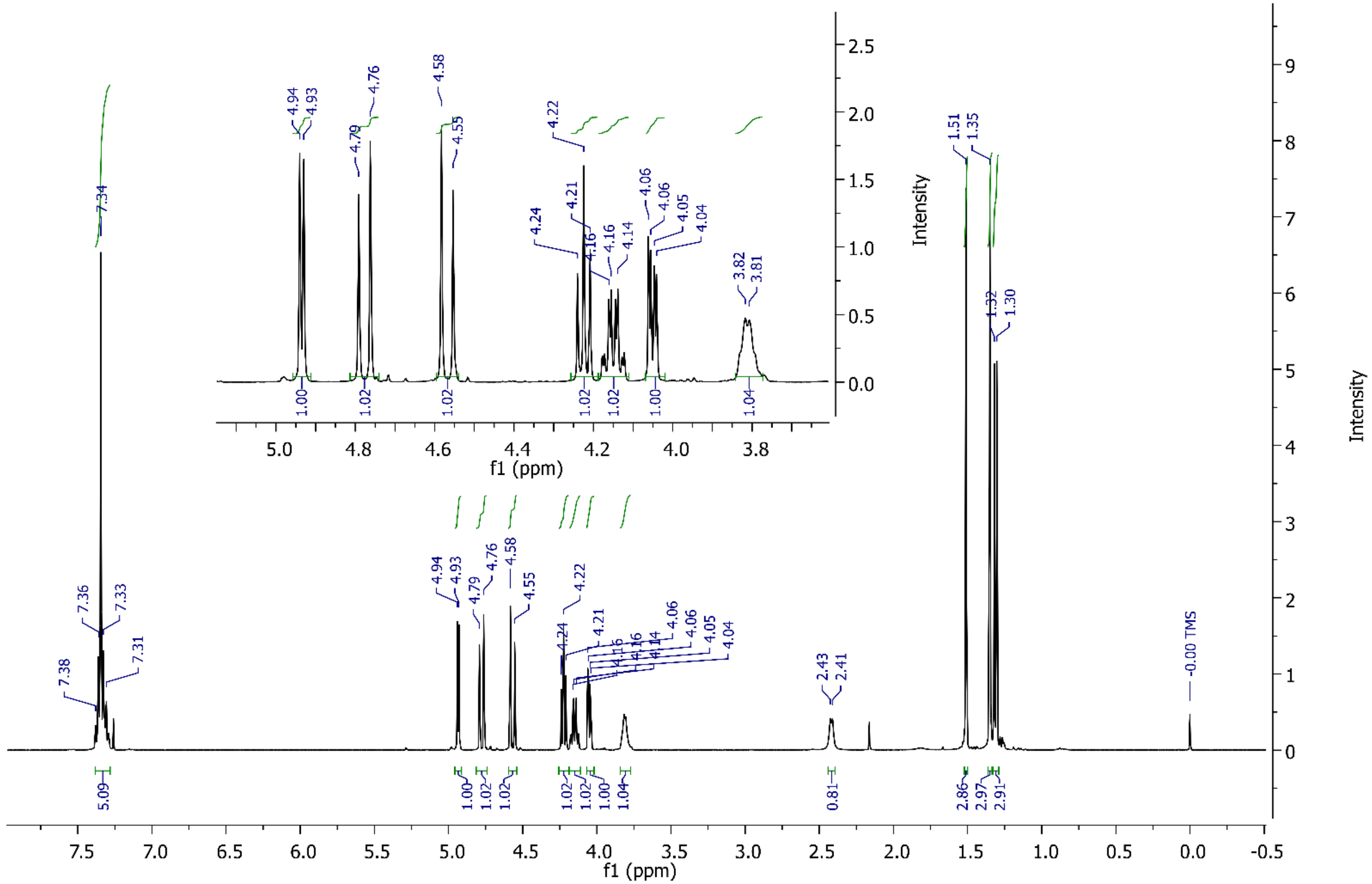

*Carbon NMR of Benzyl 3,4-O-isopropylidene-α-L-fucopyranoside (**S3**)*

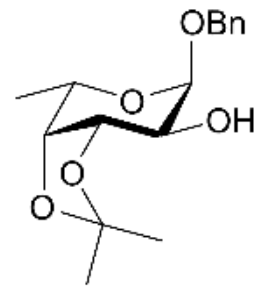

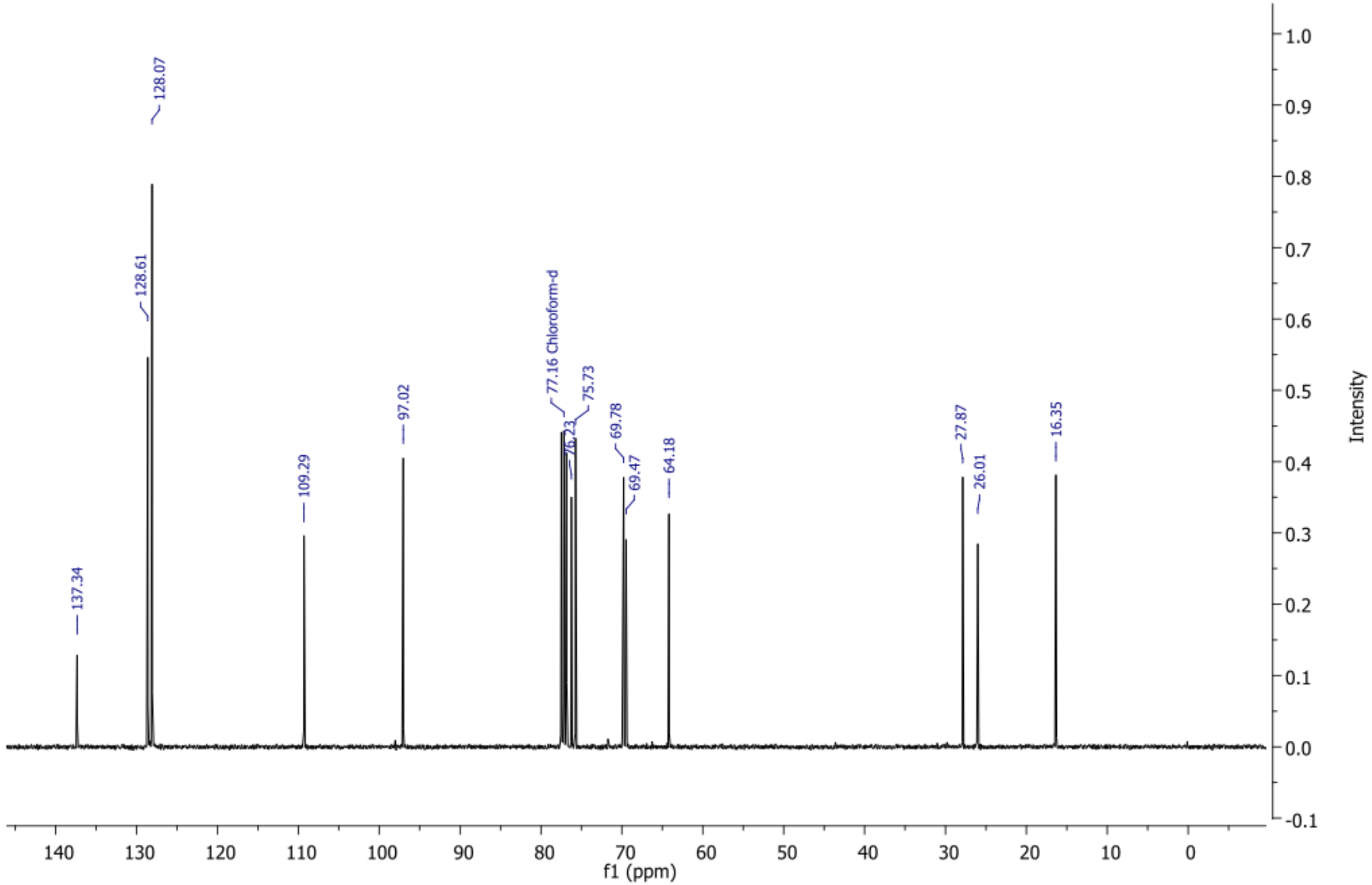

*Mass spectrum of Benzyl 3,4-O-isopropylidene-α-L-fucopyranoside (**S3**)*

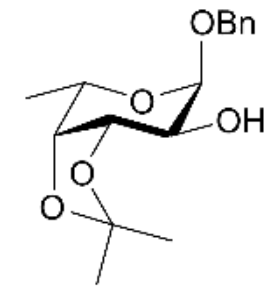

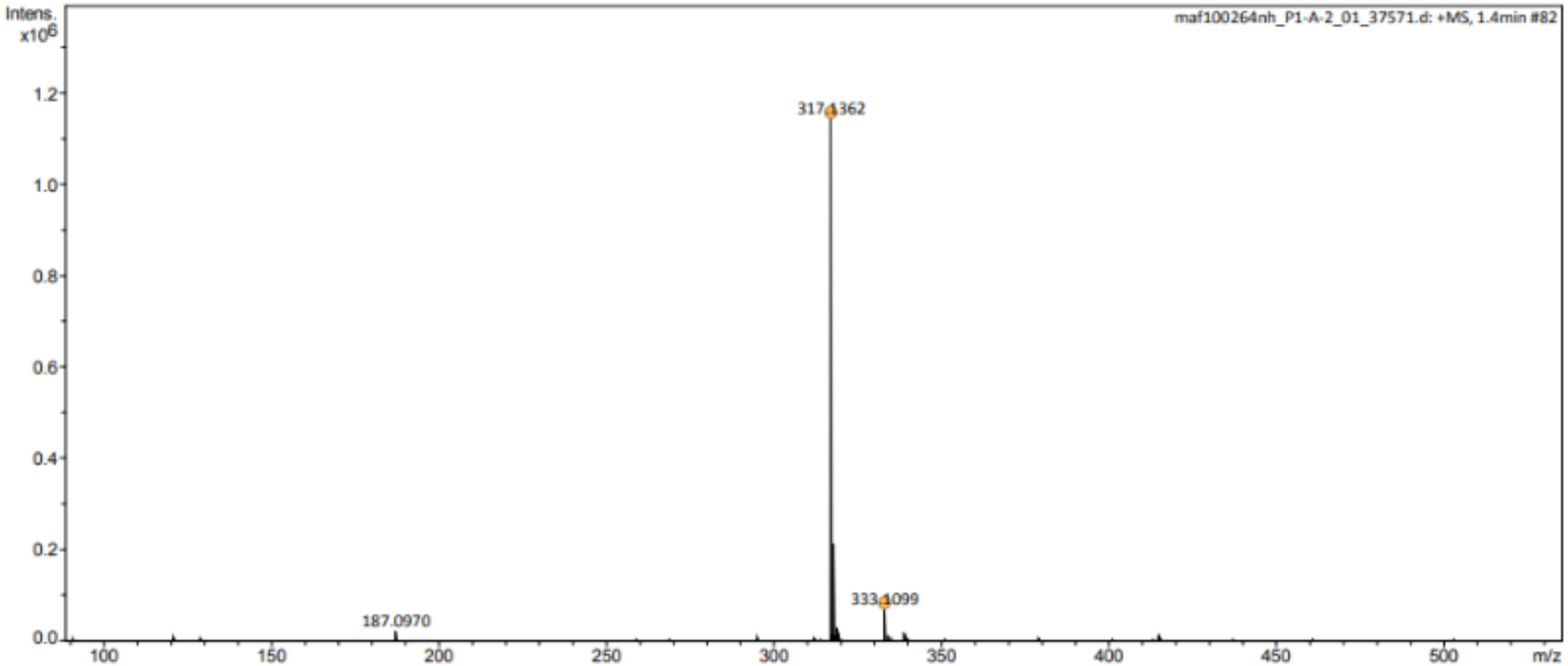

Meas. m/z	#	Ion Formula	m/z	err [ppm]	err [mDa]	mSigma	Mean err [ppm]
317.1362	1	C16H22NaO5	317.1359	-0.8	-0.3	5.6	-1.0
333.1099	1	C16H22KO5	333.1099	-0.0	-0.0	8.8	-3.8

*FT-IR (ATR) Spectrum of Benzyl 3,4-O-isopropylidene-α-L-fucopyranoside (**S3**)*

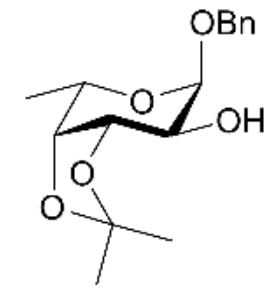

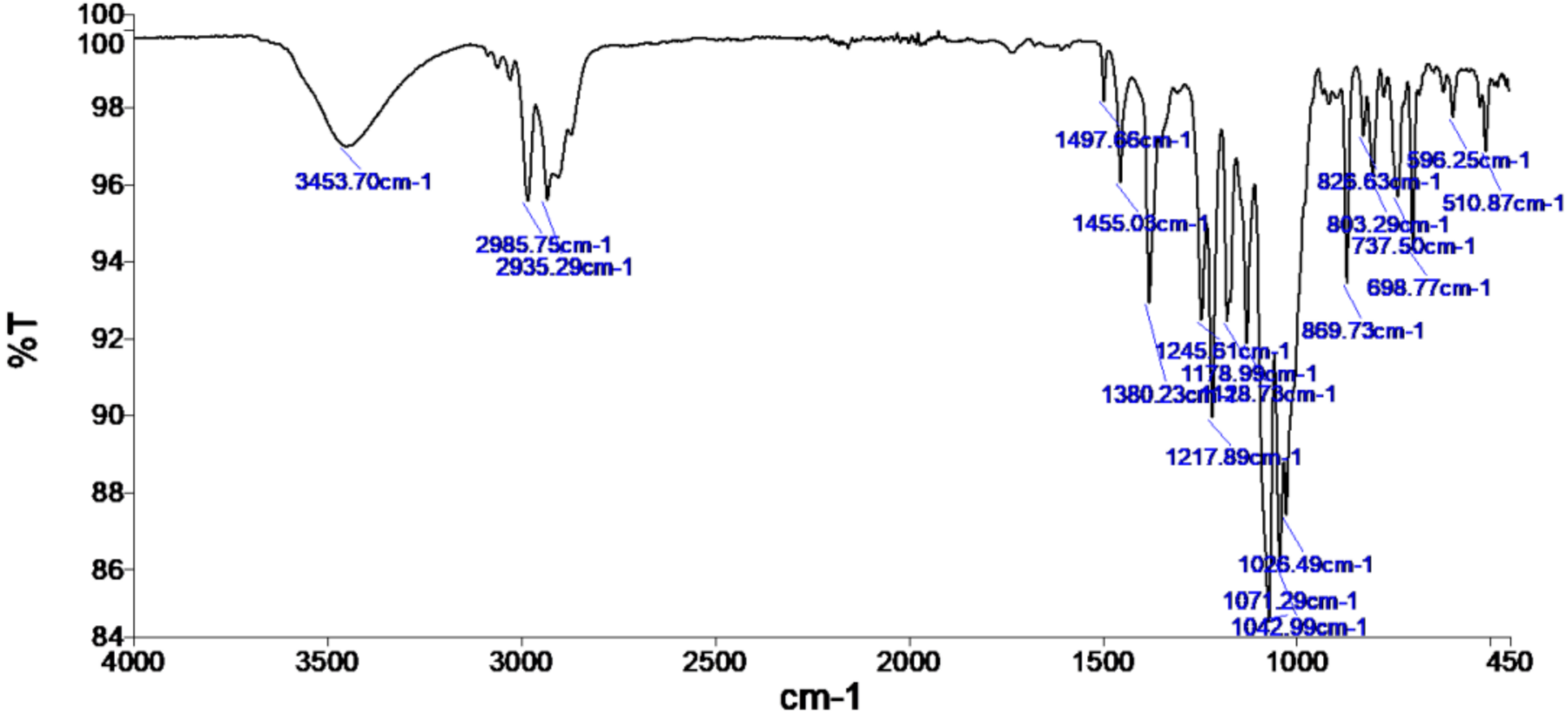

Benzyl 2-*O*-acetyl-3,4-*O*-isopropylidene-α-L-fucopyranoside (**S4**)

*Hydrogen NMR of Benzyl 2-O-acetyl-3,4-O-isopropylidene-α-L-fucopyranoside (**S4**)*

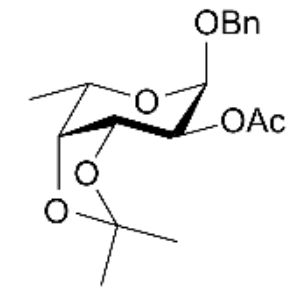

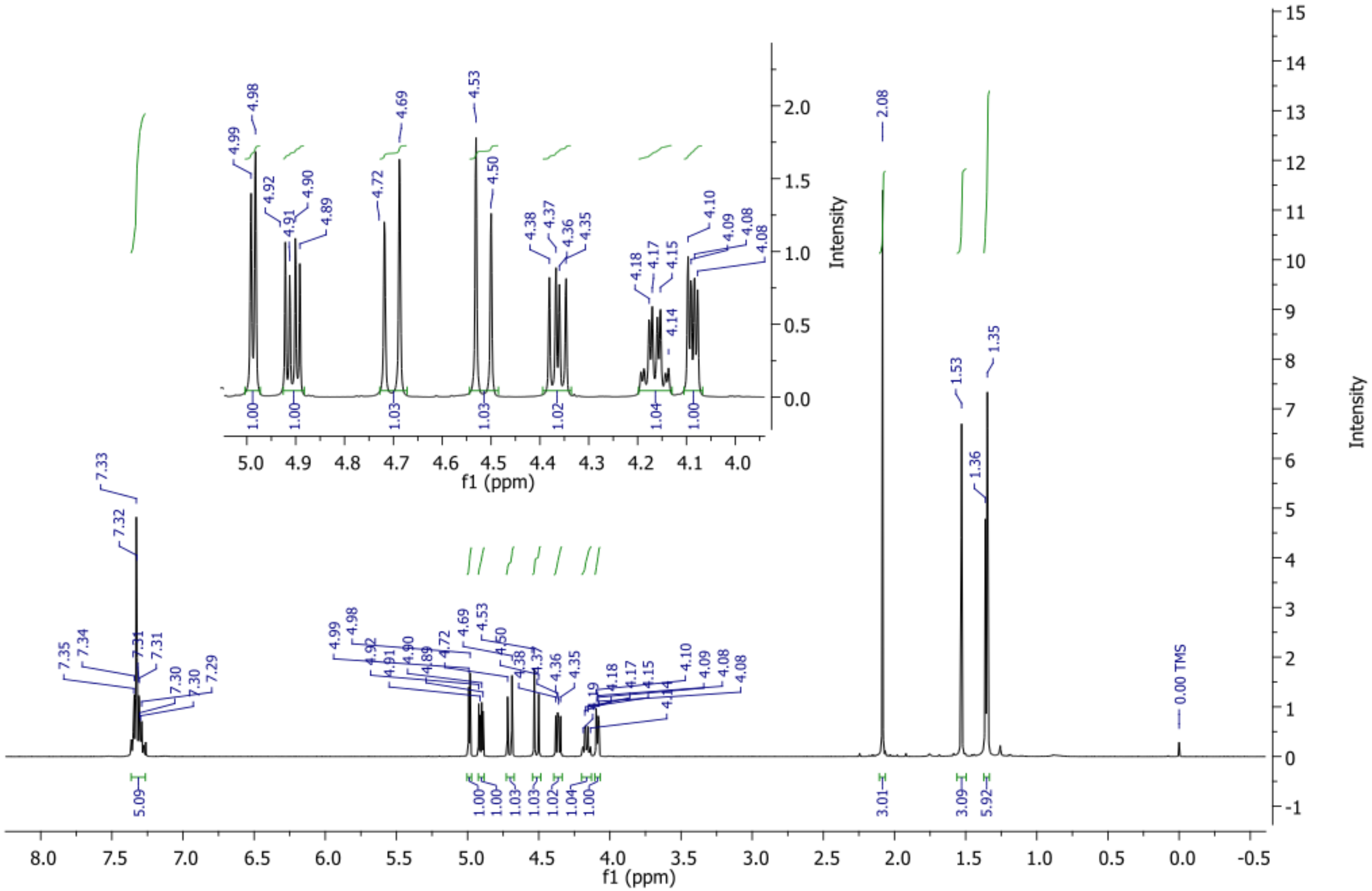

*Carbon NMR of Benzyl 2-O-acetyl-3,4-O-isopropylidene-α-L-fucopyranoside (**S4**)*

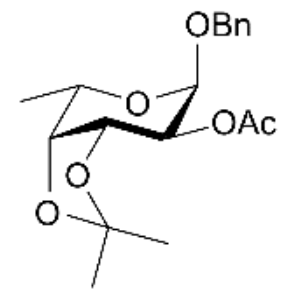

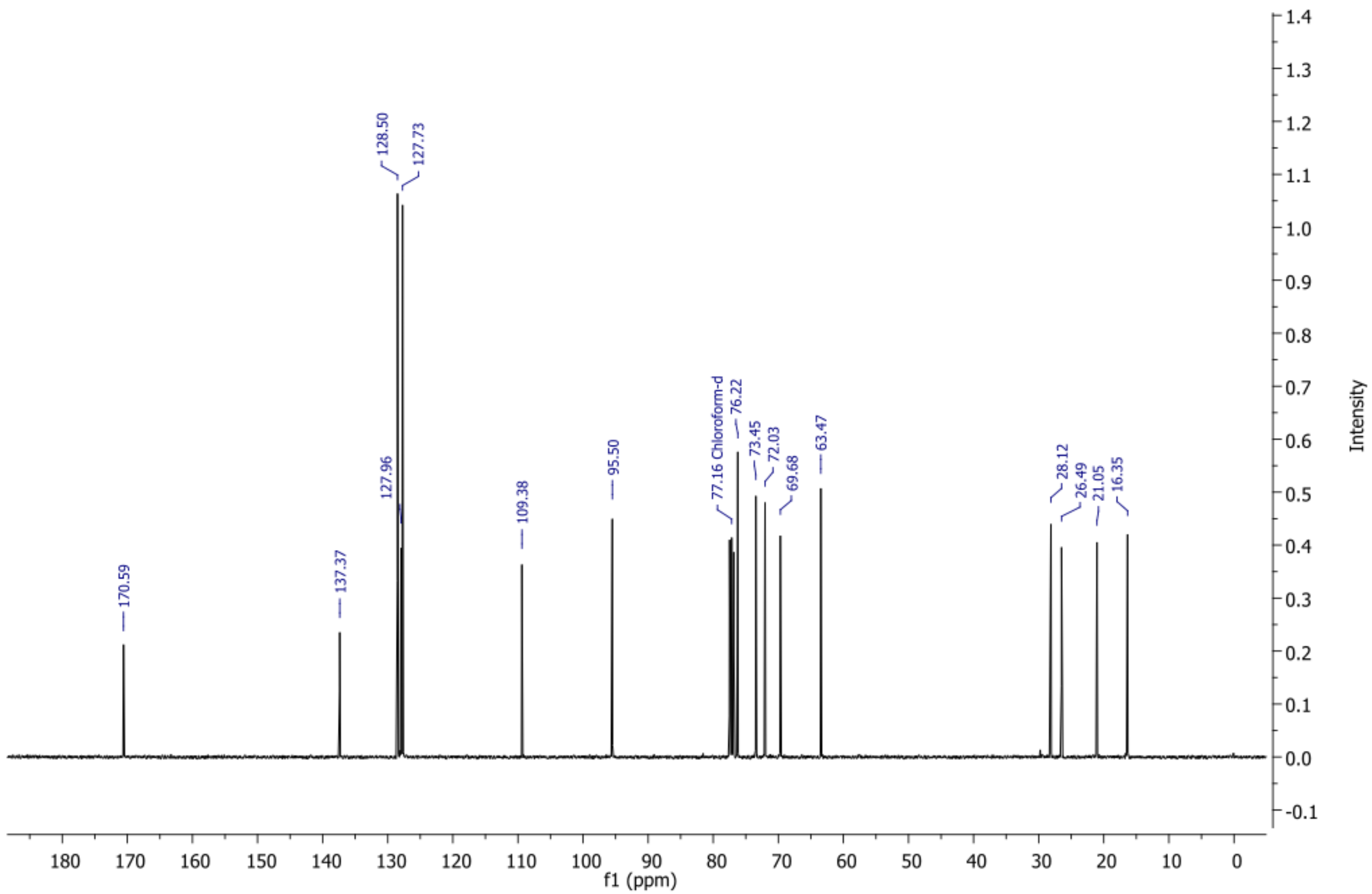

*Mass spectrum of Benzyl 2-O-acetyl-3,4-O-isopropylidene-α-L-fucopyranoside (**S4**)*

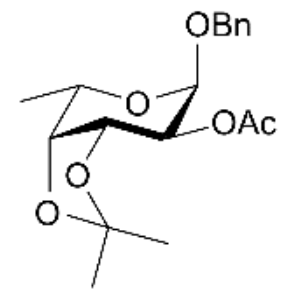

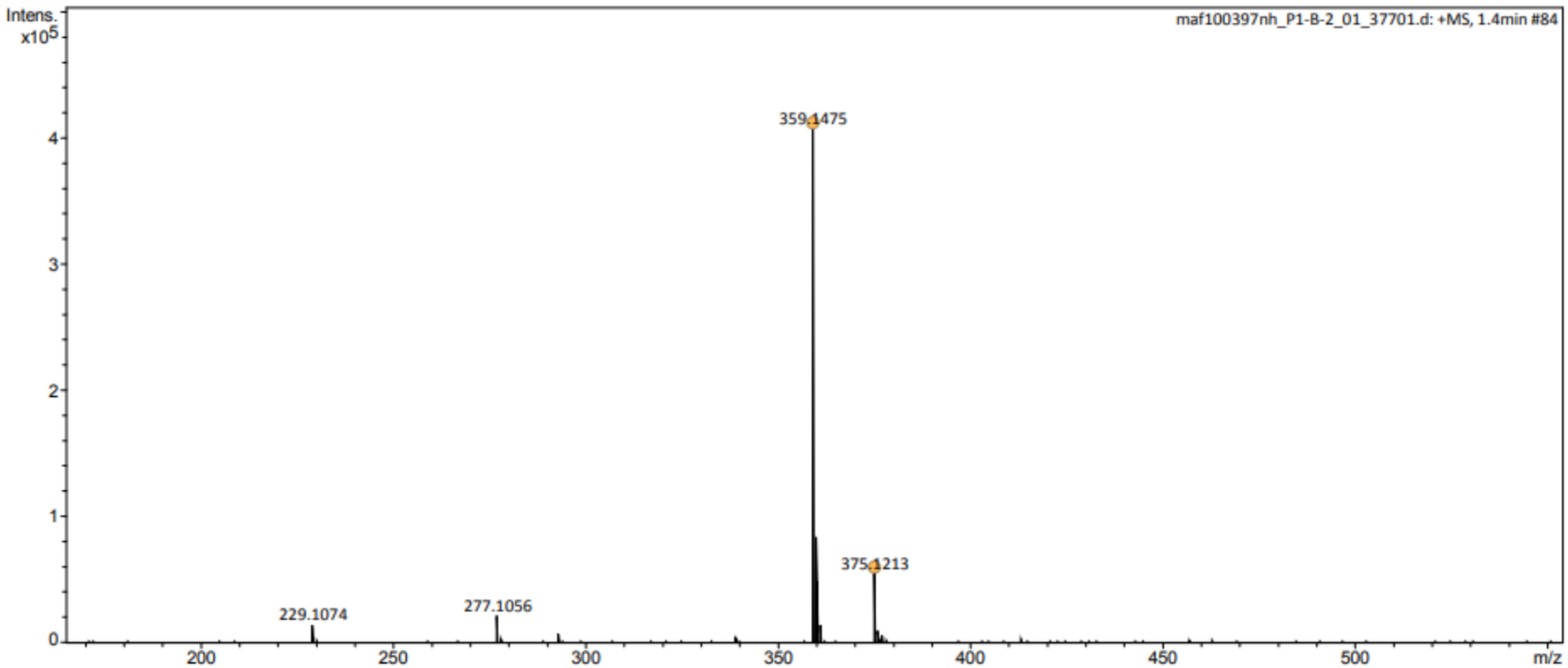

Meas. m/z	#	Ion Formula	m/z	err [ppm]	err [mDa]	mSigma	Mean err [ppm]
359.1475	1	C18H24NaO6	359.1465	-2.8	-1.0	5.1	-3.0
375.1213	1	C18H24KO6	375.1204	-2.3	-0.9	14.3	-1.8

*FT-IR (ATR) of Benzyl 2-O-acetyl-3,4-O-isopropylidene-α-L-fucopyranoside (**S4**)*

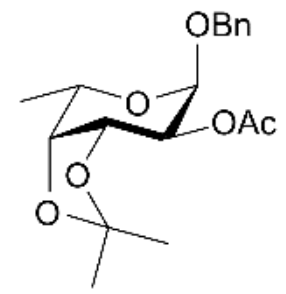

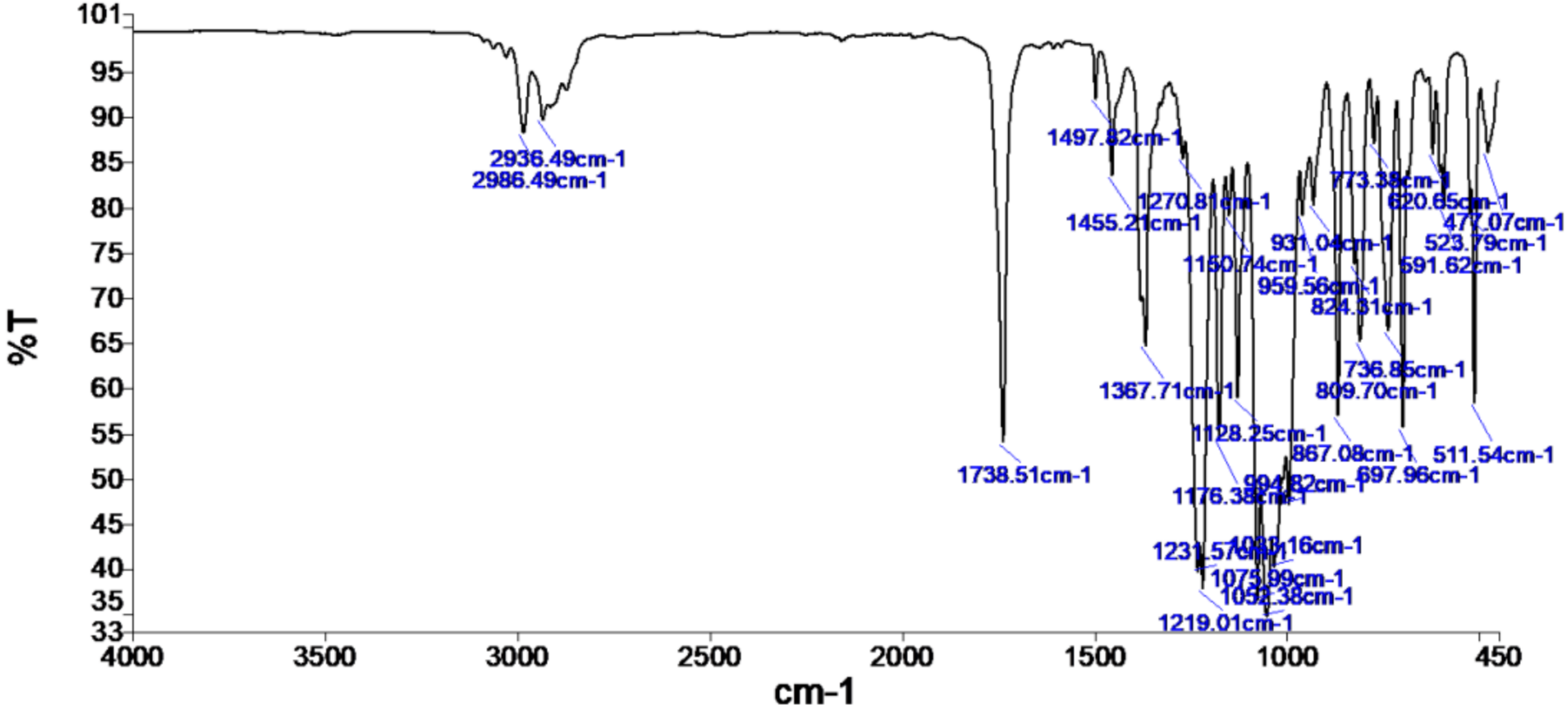

Benzyl 2-*O*-acetyl-3,4-di-*O*-methanesulfonyl-α-L-fucopyranoside (**S5**)

*Hydrogen NMR of Benzyl 2-O-acetyl-3,4-di-O-methanesulfonyl-α-L-fucopyranoside (**S5**)*

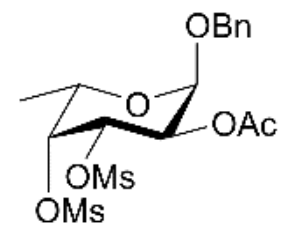

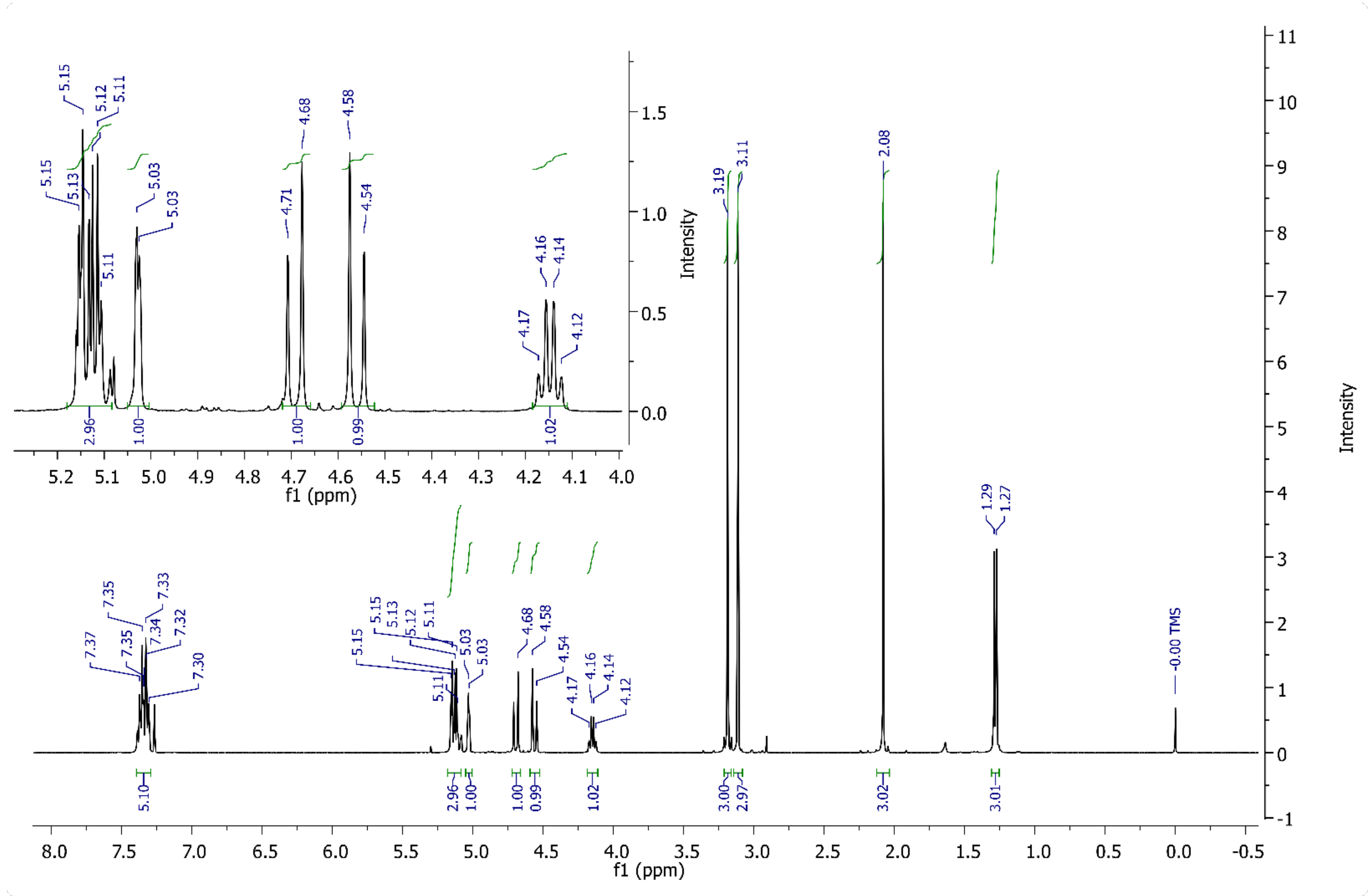

*Carbon NMR of Benzyl 2-O-acetyl-3,4-di-O-methanesulfonyl-α-L-fucopyranoside (**S5**)*

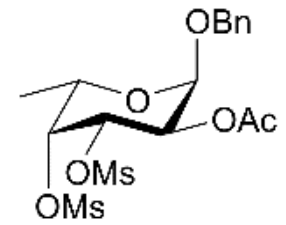

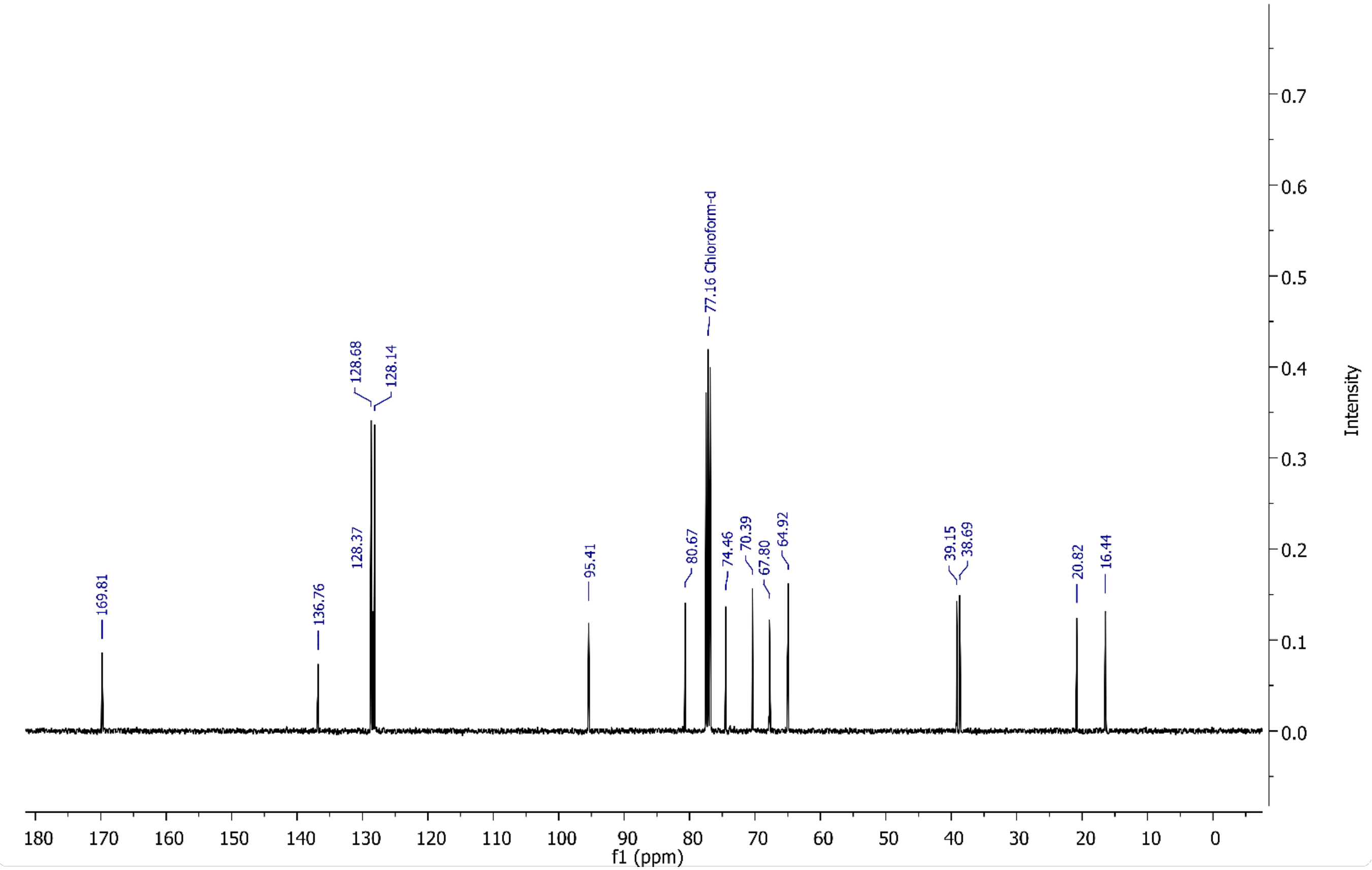

*Mass spectrum of Benzyl 2-O-acetyl-3,4-di-O-methanesulfonyl-α-L-fucopyranoside (**S5**)*

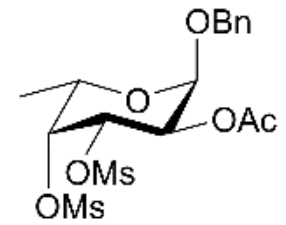

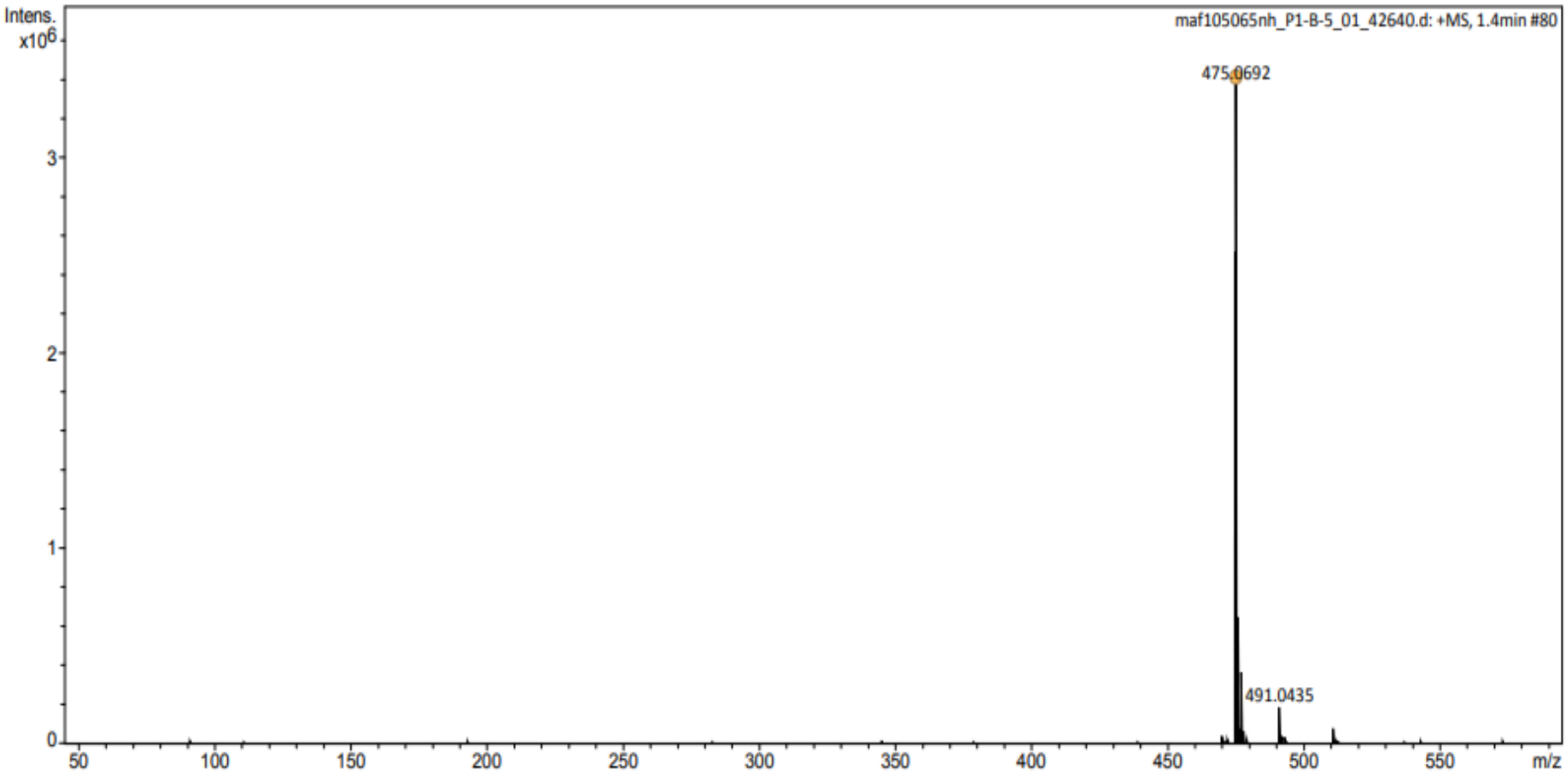

Meas. m/z	#	Ion Formula	m/z	err [ppm]	err [mDa]	mSigma	Mean err [ppm]
475.0692	1	C17H24NaO10S2	475.0703	2.3	1.1	11.1	1.8

*FT-IR (ATR) of Benzyl 2-O-acetyl-3,4-di-O-methanesulfonyl-α-L-fucopyranoside (**S5**)*

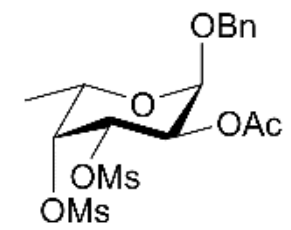

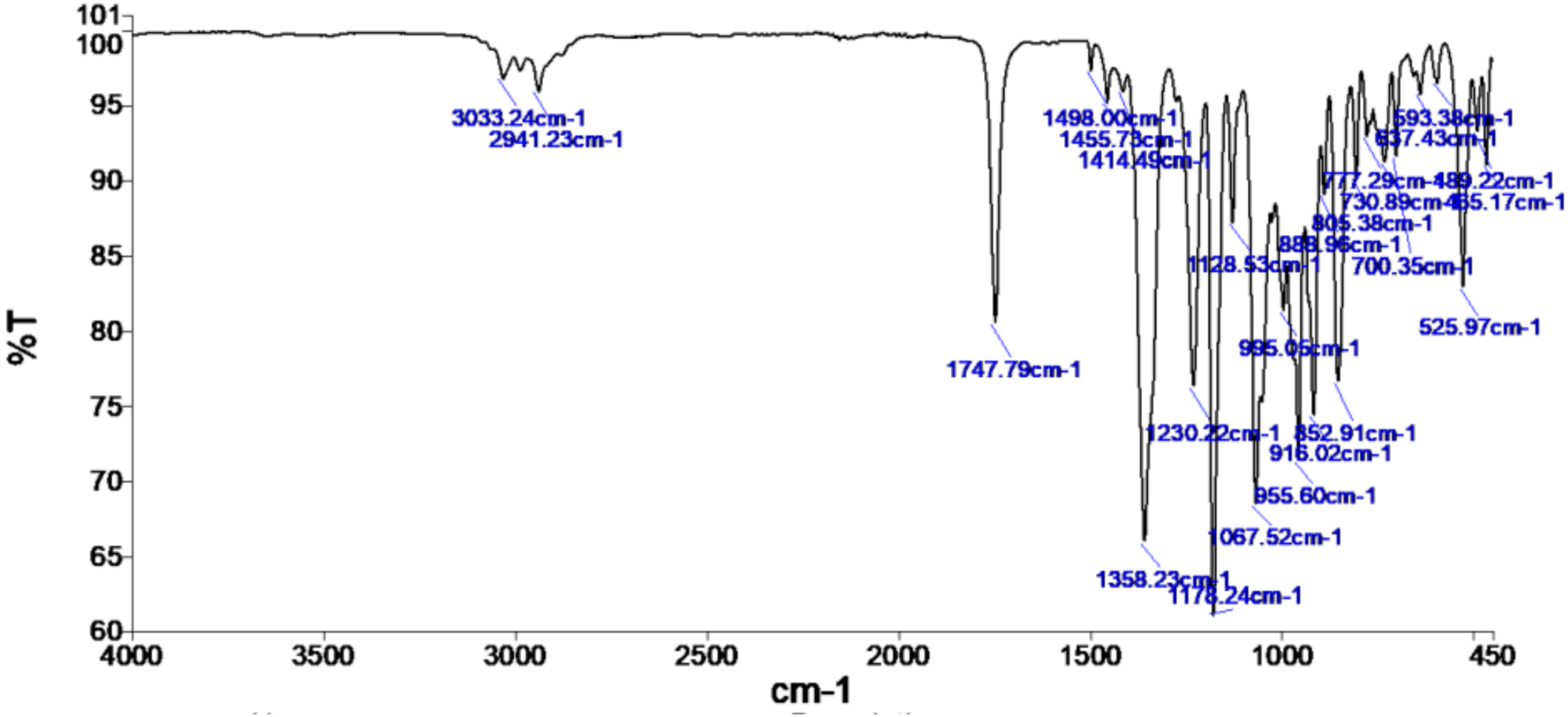

Benzyl 2-*O*-acetyl-4-azido-3-*O*-methanesulfonyl-α-L-fucopyranoside (**S6**)

*Hydrogen NMR spectrum of Benzyl 2-O-acetyl-4-azido-3-O-methanesulfonyl-α-L-fucopyranoside (**S6**)*

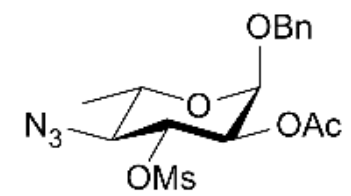

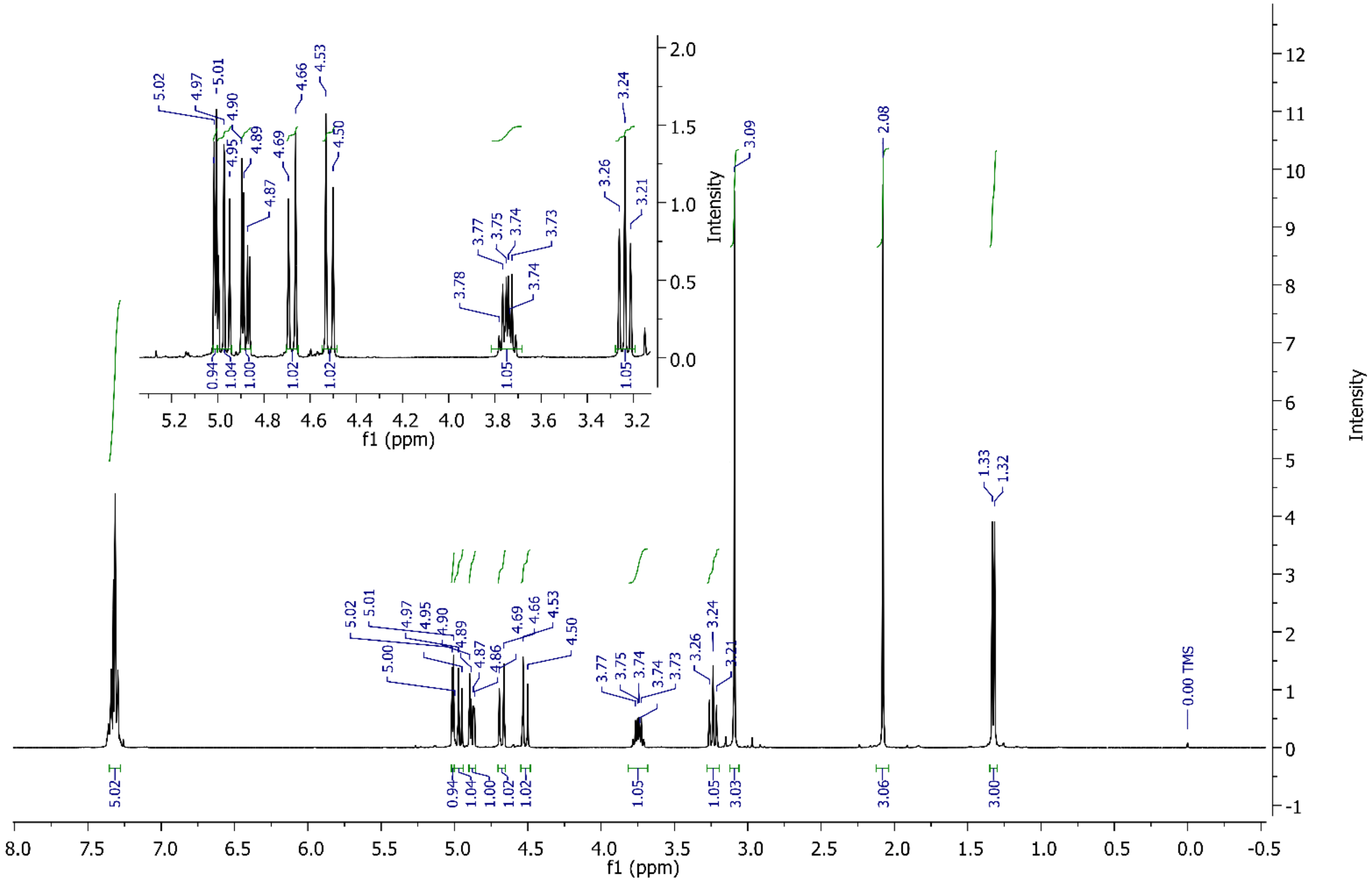

*Carbon NMR spectrum of Benzyl 2-O-acetyl-4-azido-3-O-methanesulfonyl-α-L-fucopyranoside (**S6**)*

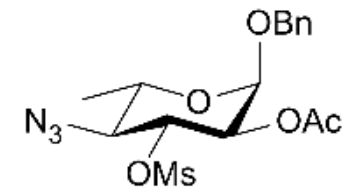

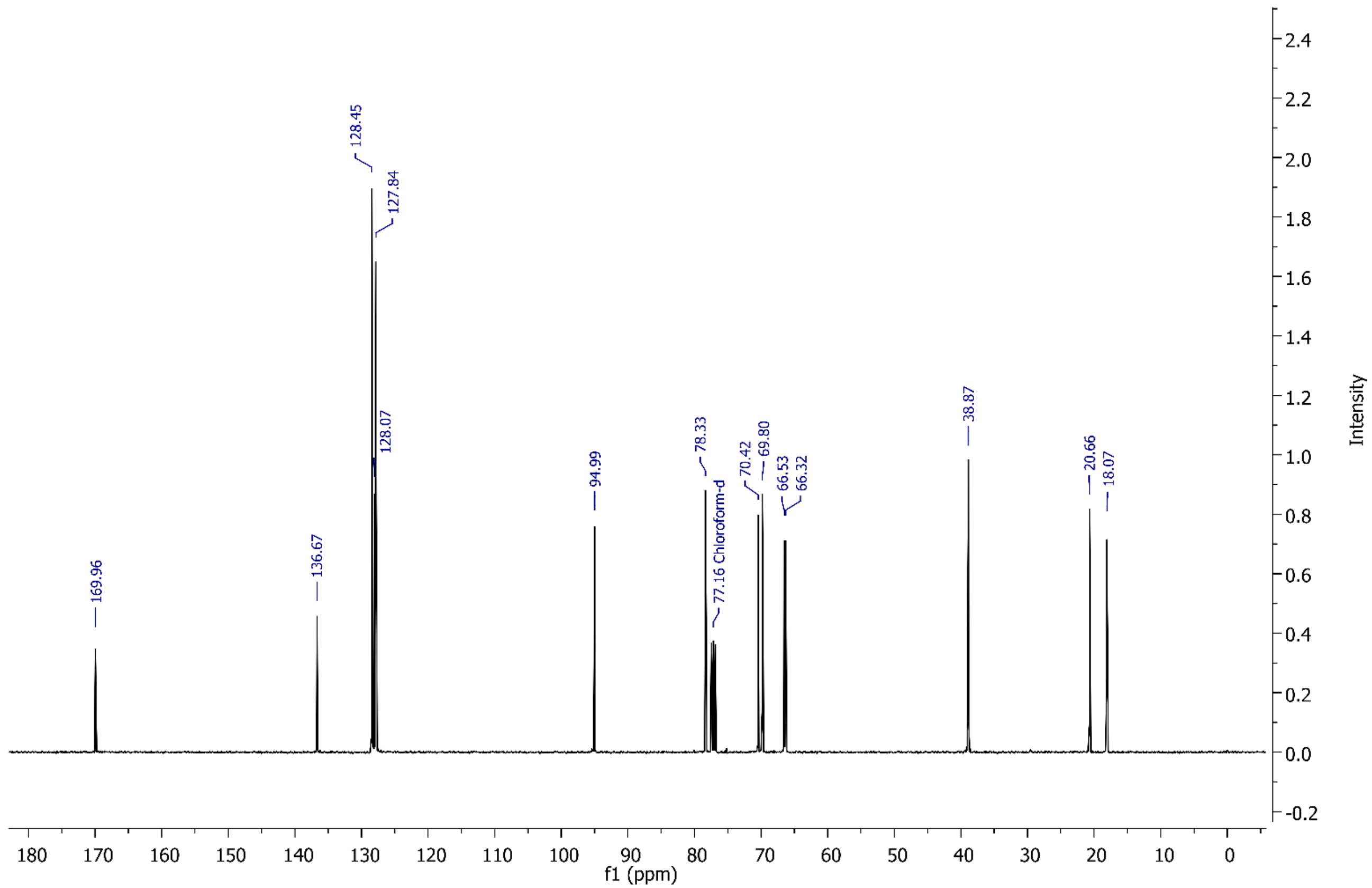

*Mass spectrum of Benzyl 2-O-acetyl-4-azido-3-O-methanesulfonyl-α-L-fucopyranoside (**S6**)*

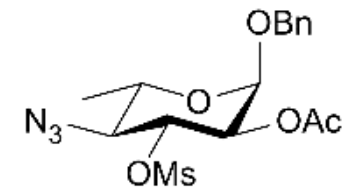

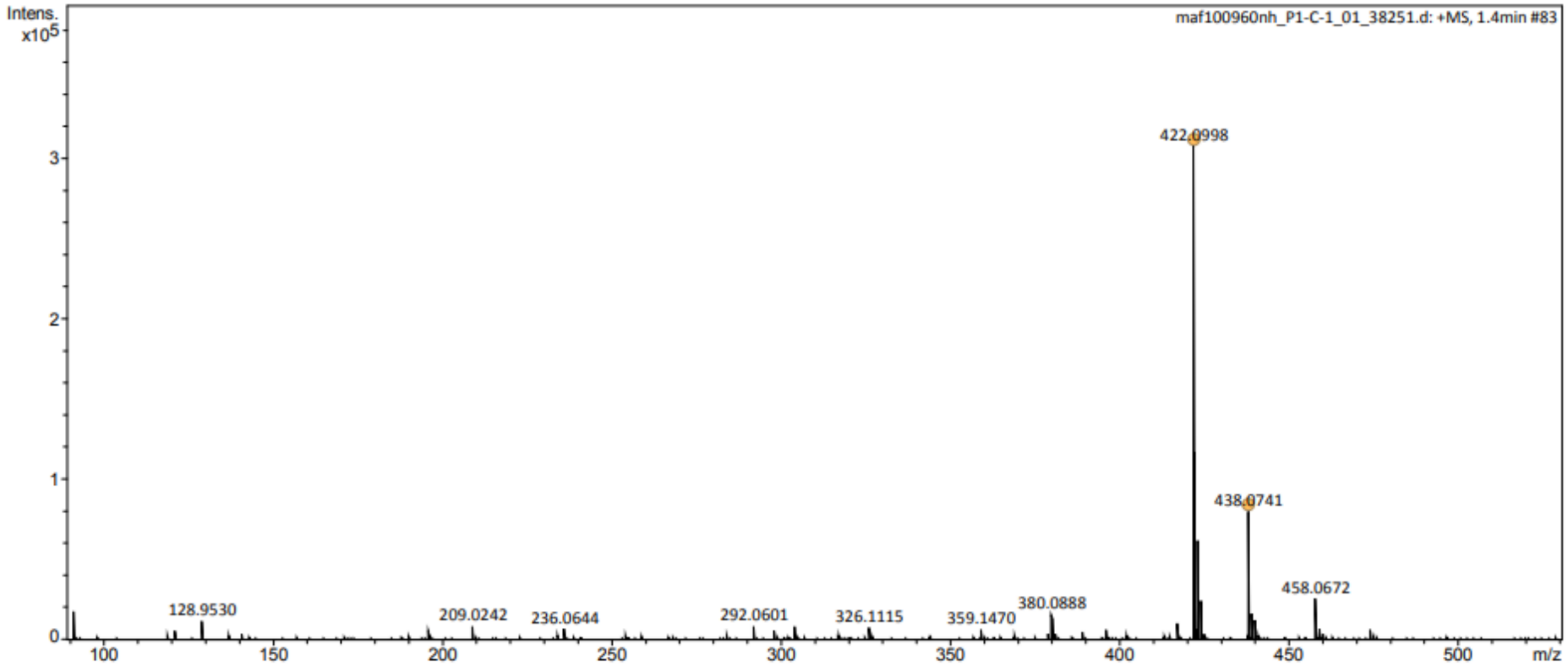

Meas. m/z	#	Ion Formula	m/z	err [ppm]	err [mDa]	mSigma	Mean err [ppm]
422.0998	1	C16H21N3NaO7S	422.0992	-1.4	-0.6	2.8	-2.2
438.0741	1	C16H21KN3O7S	438.0732	-2.1	-0.9	6.8	-5.5

*FT-IR (ATR) spectrum of Benzyl 2-O-acetyl-4-azido-3-O-methanesulfonyl-α-L-fucopyranoside (**S6**)*

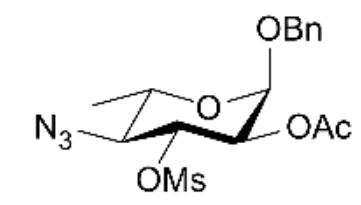

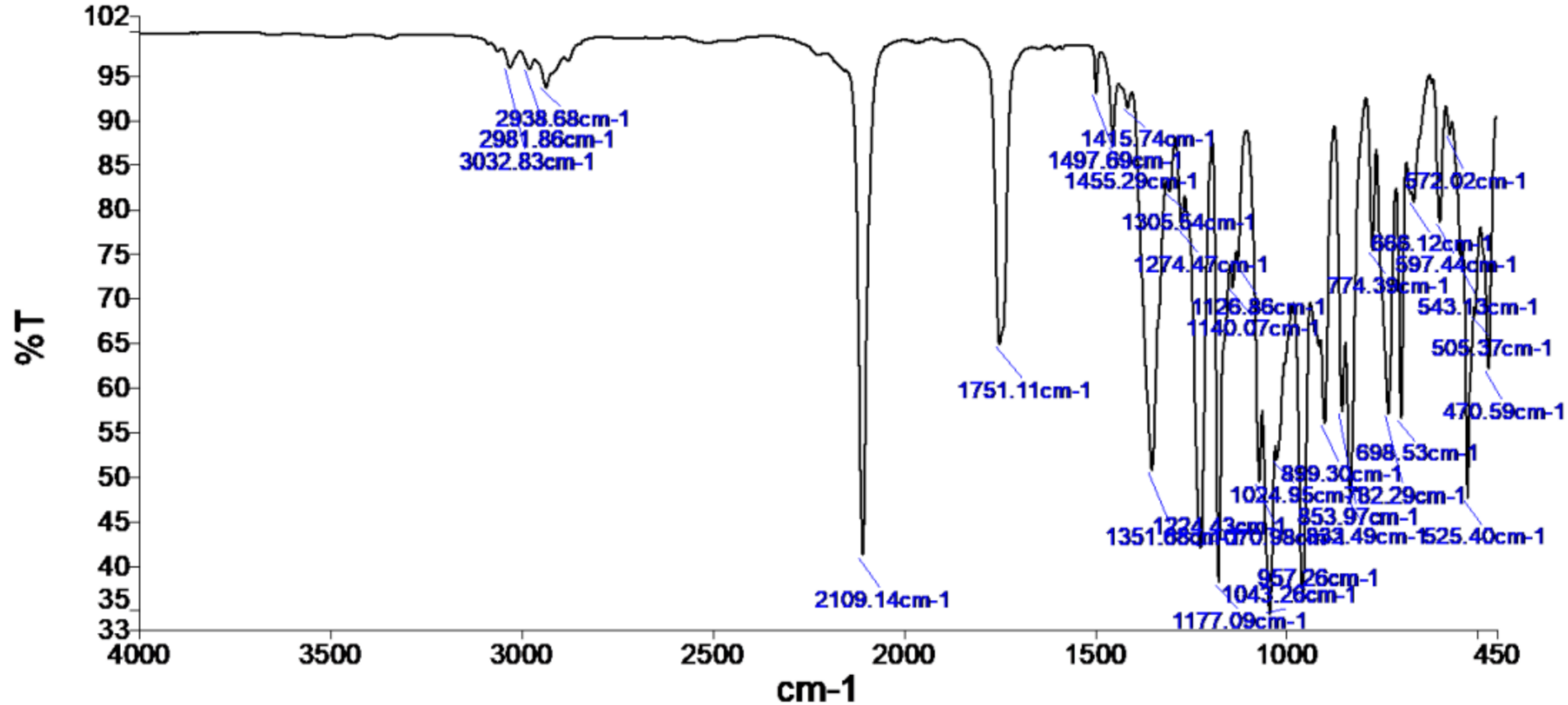

Benzyl 2,3-anhydro-4-azido-4,6-dideoxy-α-L-allopyranoside (**S7**)

*Hydrogen NMR spectrum of Benzyl 2,3-anhydro-4-azido-4,6-dideoxy-α-L-allopyranoside (**S7**)*

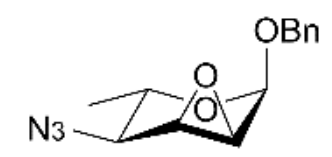

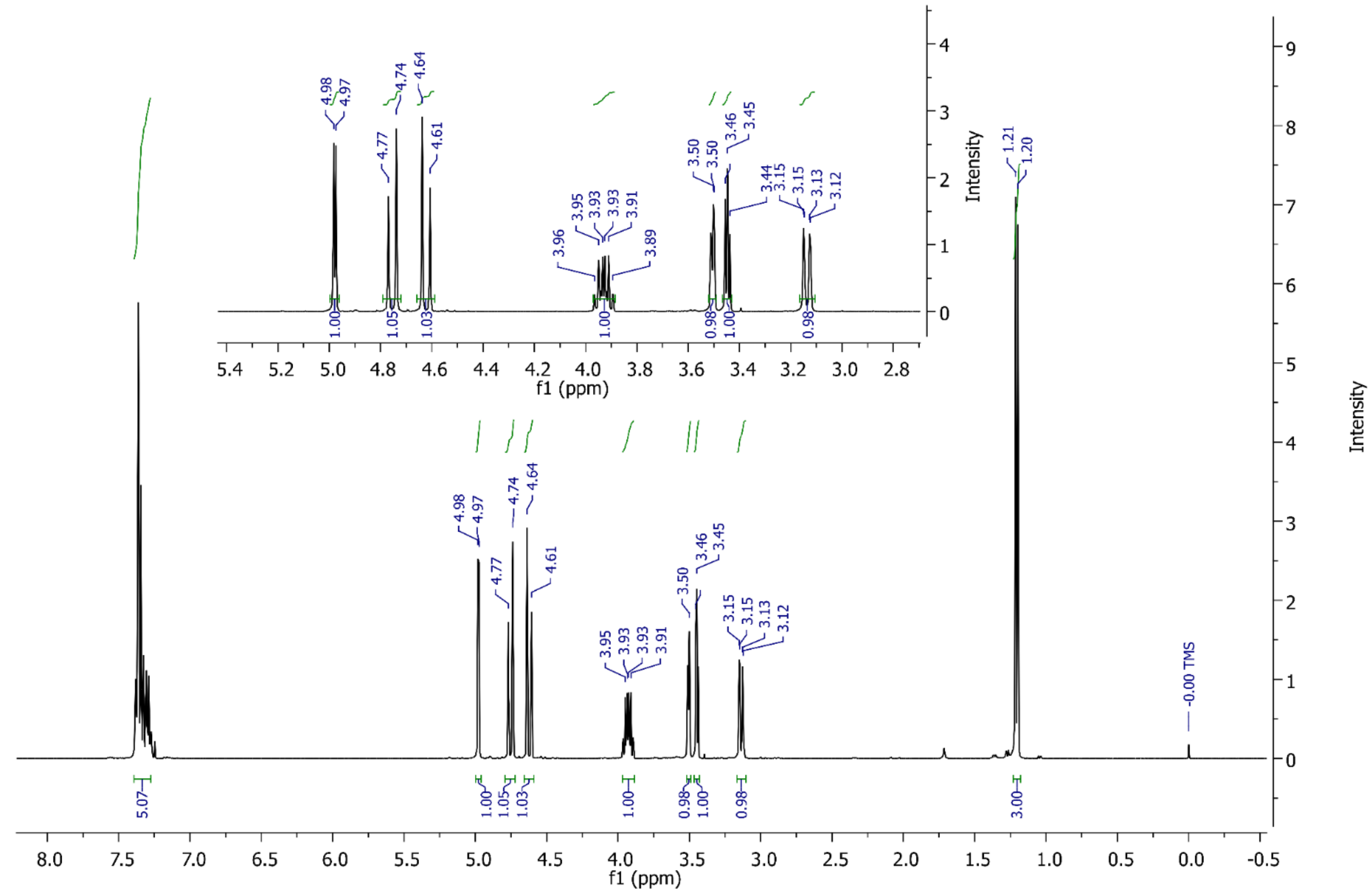

*Carbon NMR spectrum of Benzyl 2,3-anhydro-4-azido-4,6-dideoxy-α-L-allopyranoside (**S7**)*

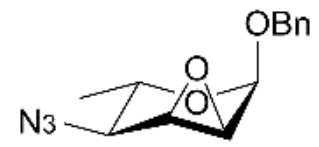

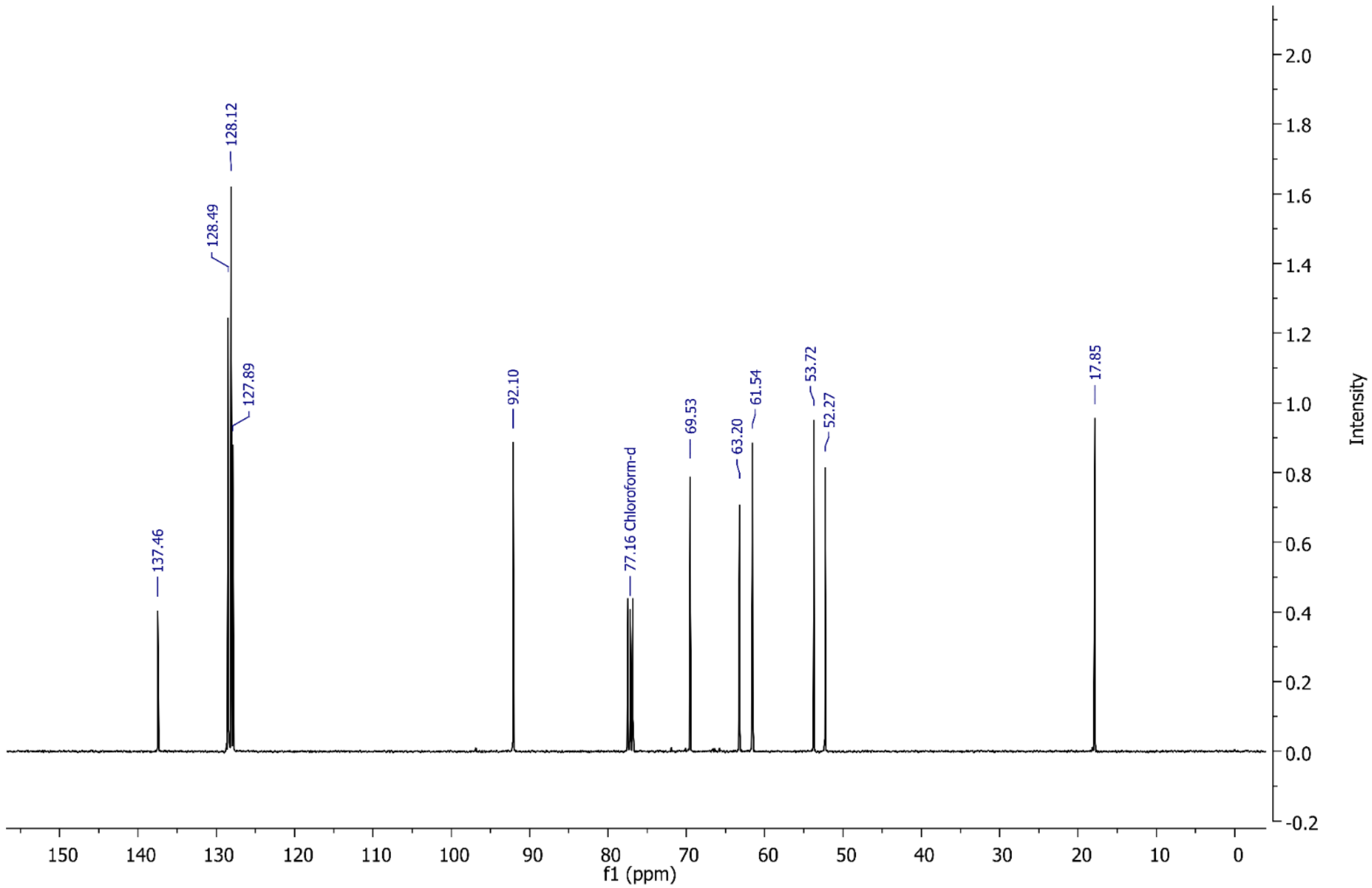

*Mass spectrum of Benzyl 2,3-anhydro-4-azido-4,6-dideoxy-α-L-allopyranoside (**S7**)*

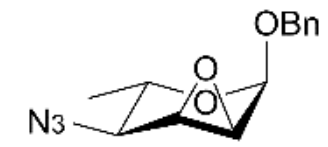

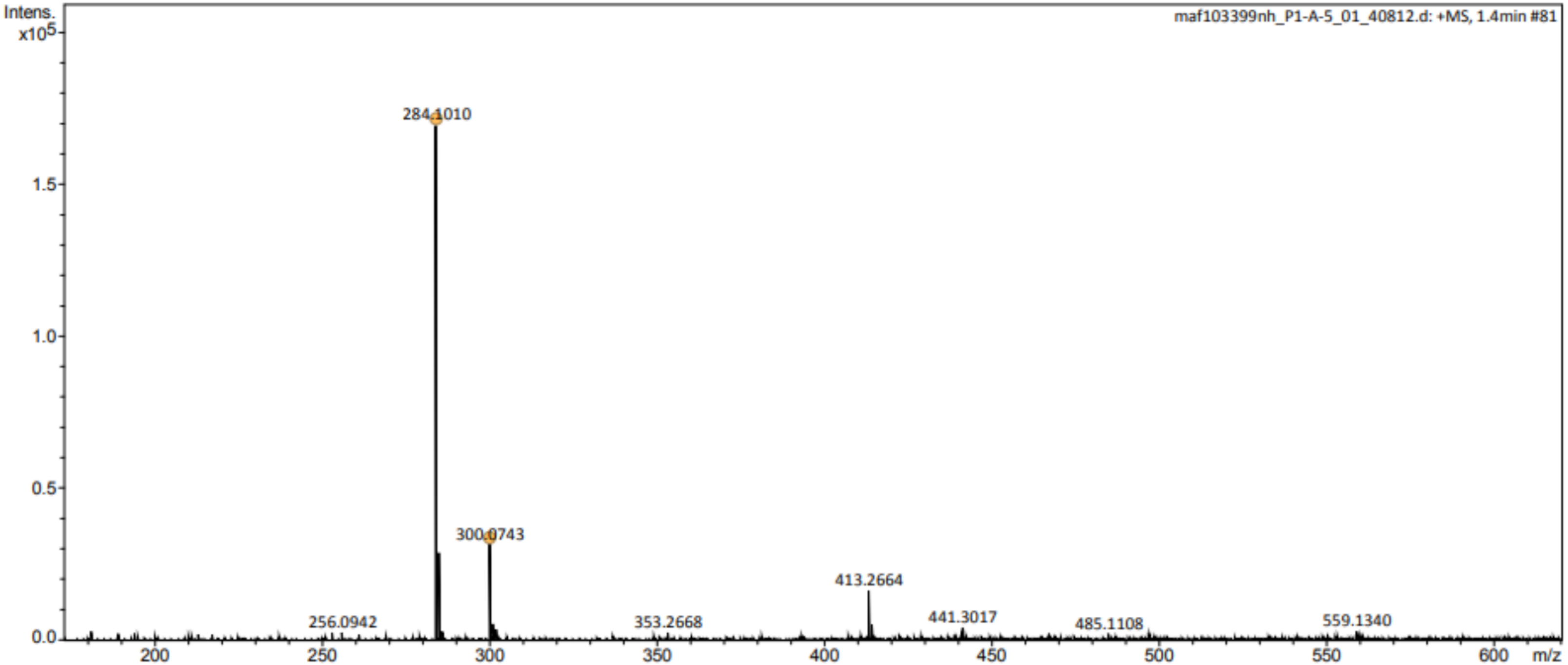

Meas. m/z	#	Ion Formula	m/z	err [ppm]	err [mDa]	mSigma	Mean err [ppm]
284.1010	1	C13H15N3NaO3	284.1006	-1.6	-0.5	9.0	0.3
300.0743	1	C13H15KN3O3	300.0745	0.8	0.2	19.4	-0.2

*IR spectrum of Benzyl 2,3-anhydro-4-azido-4,6-dideoxy-α-L-allopyranoside (**S7**)*

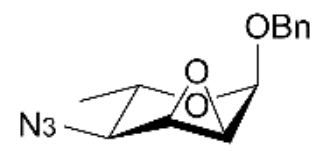

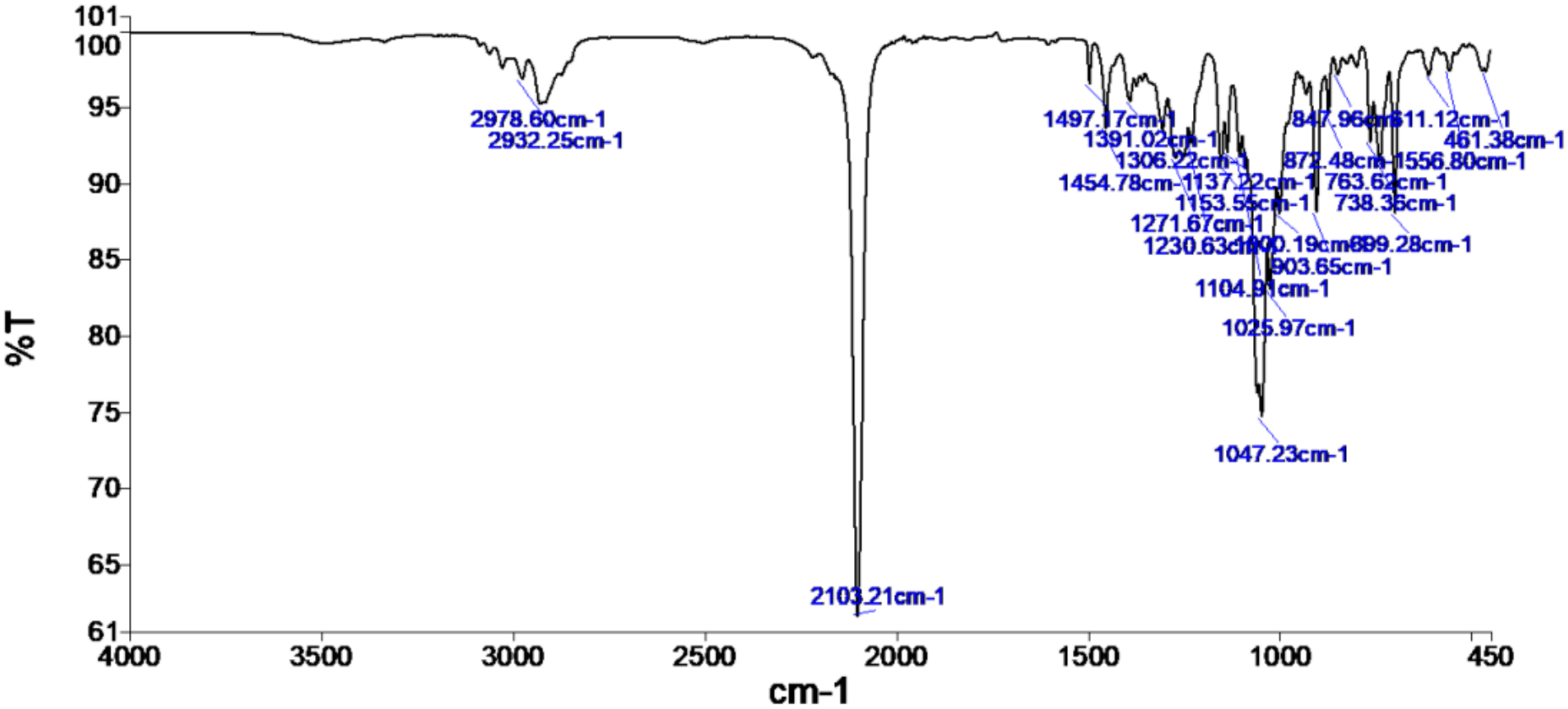

Methyl (*R*)-3-(benzyloxy)butanoate (**S8**)

*Hydrogen NMR spectrum of Methyl (R)-3-(benzyloxy)butanoate (**S8**)*

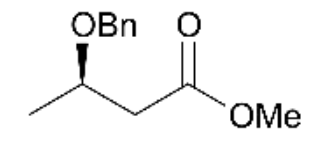

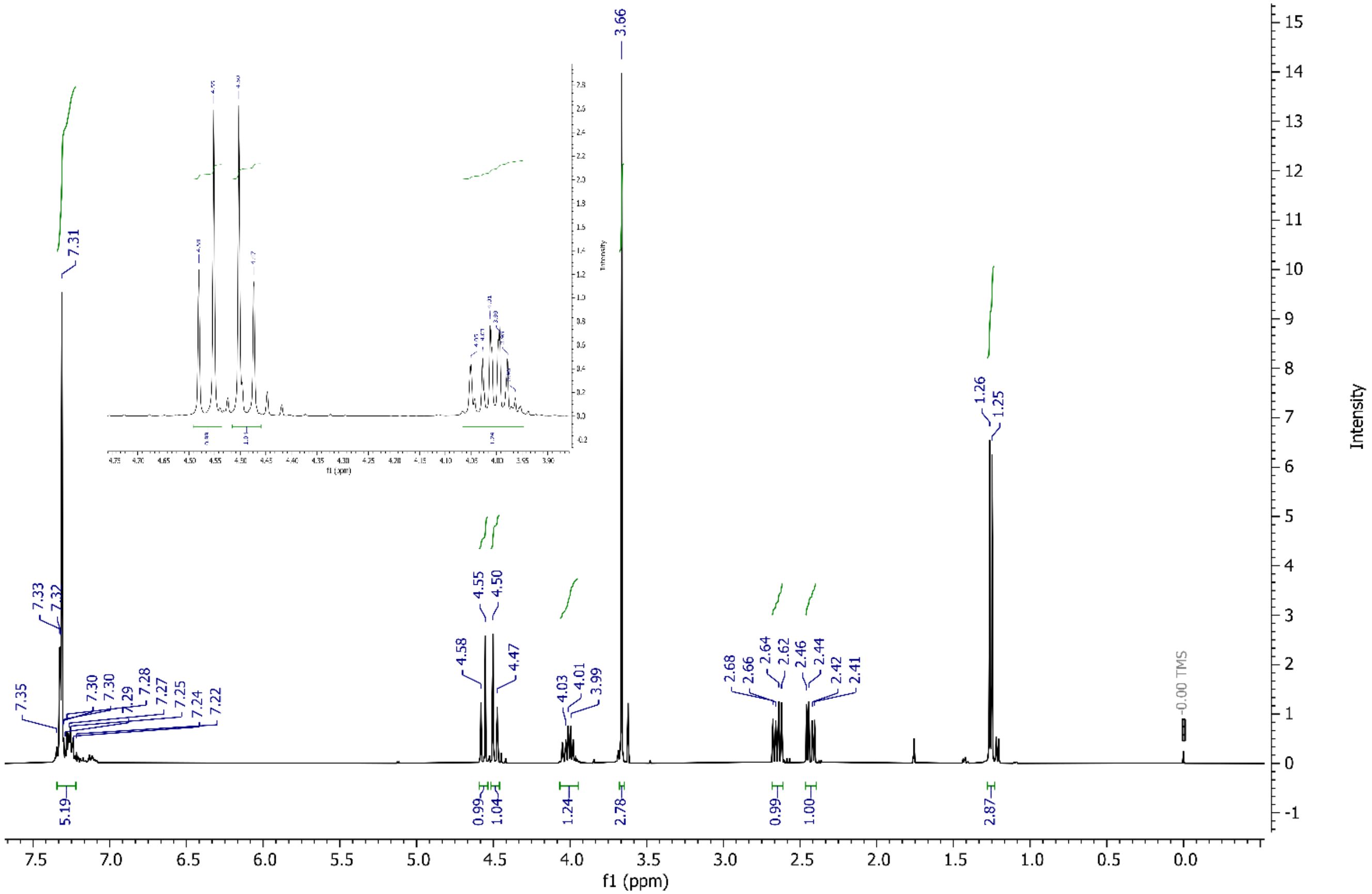

*Carbon NMR spectrum of Methyl (R)-3-(benzyloxy)butanoate (**S8**)*

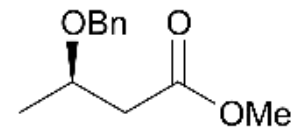

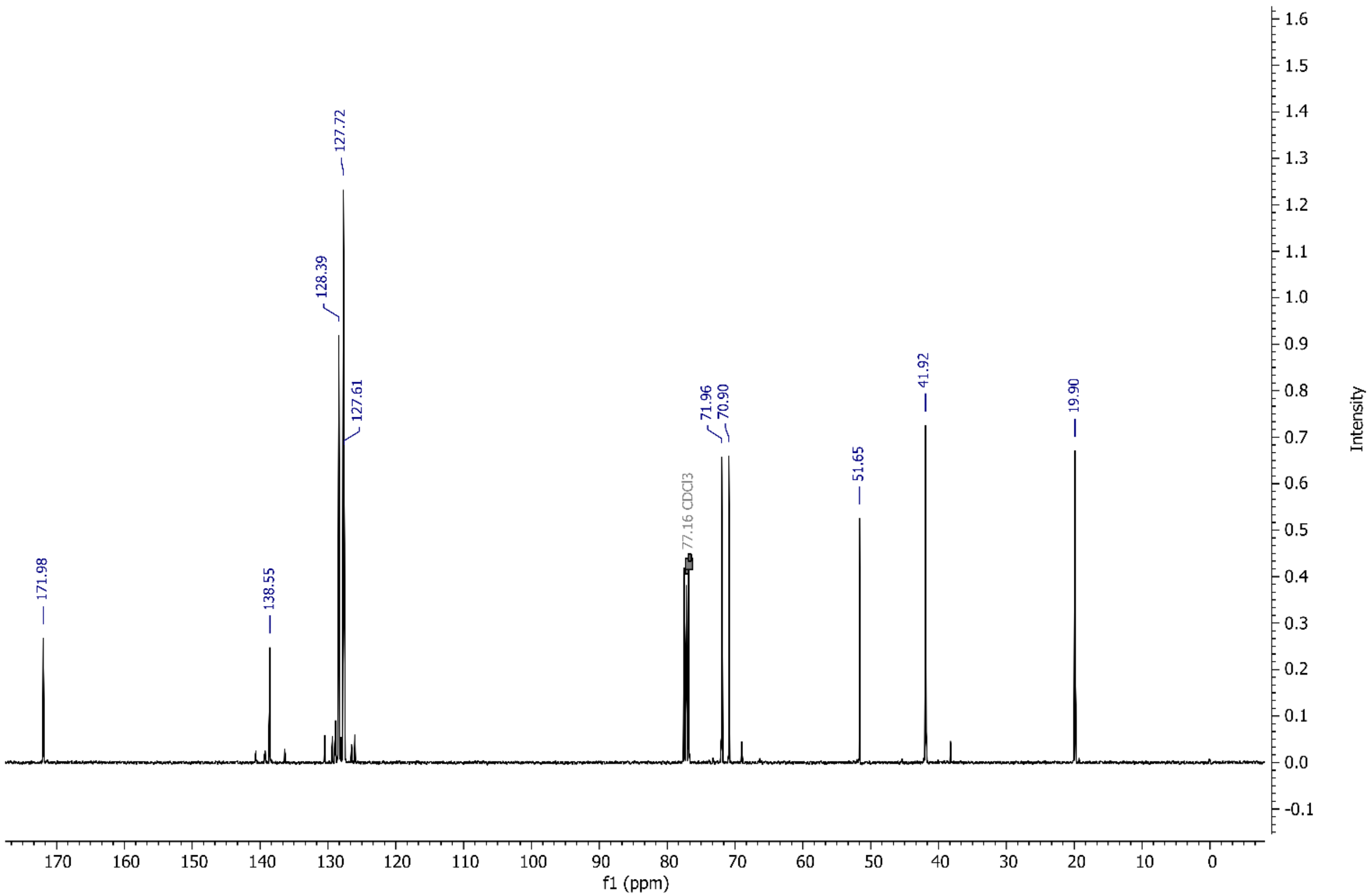

*Mass spectrum of Methyl (R)-3-(benzyloxy)butanoate (**S8**)*

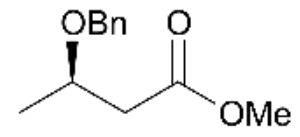

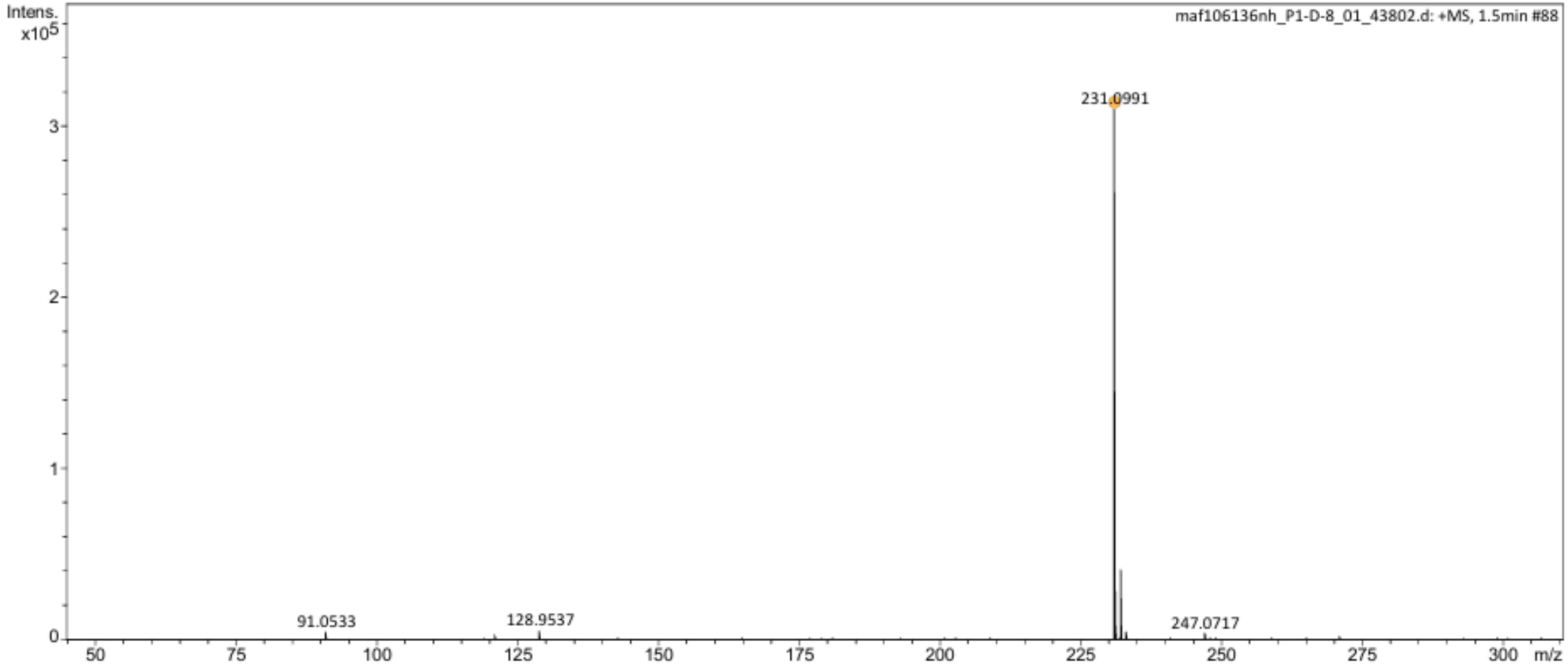

Meas. m/z	#	Ion Formula	m/z	err [ppm]	err [mDa]	mSigma	Mean err [ppm]
231.0991	1	C12H16NaO3	231.0992	0.2	0.0	0.8	-1.1

(*R*)-3-Benzyloxybutanoic acid (**S9**)

*Hydrogen NMR spectrum of (R)-3-Benzyloxybutanoic acid (**S9**)*

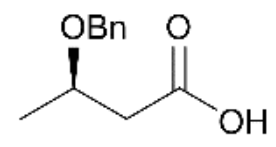

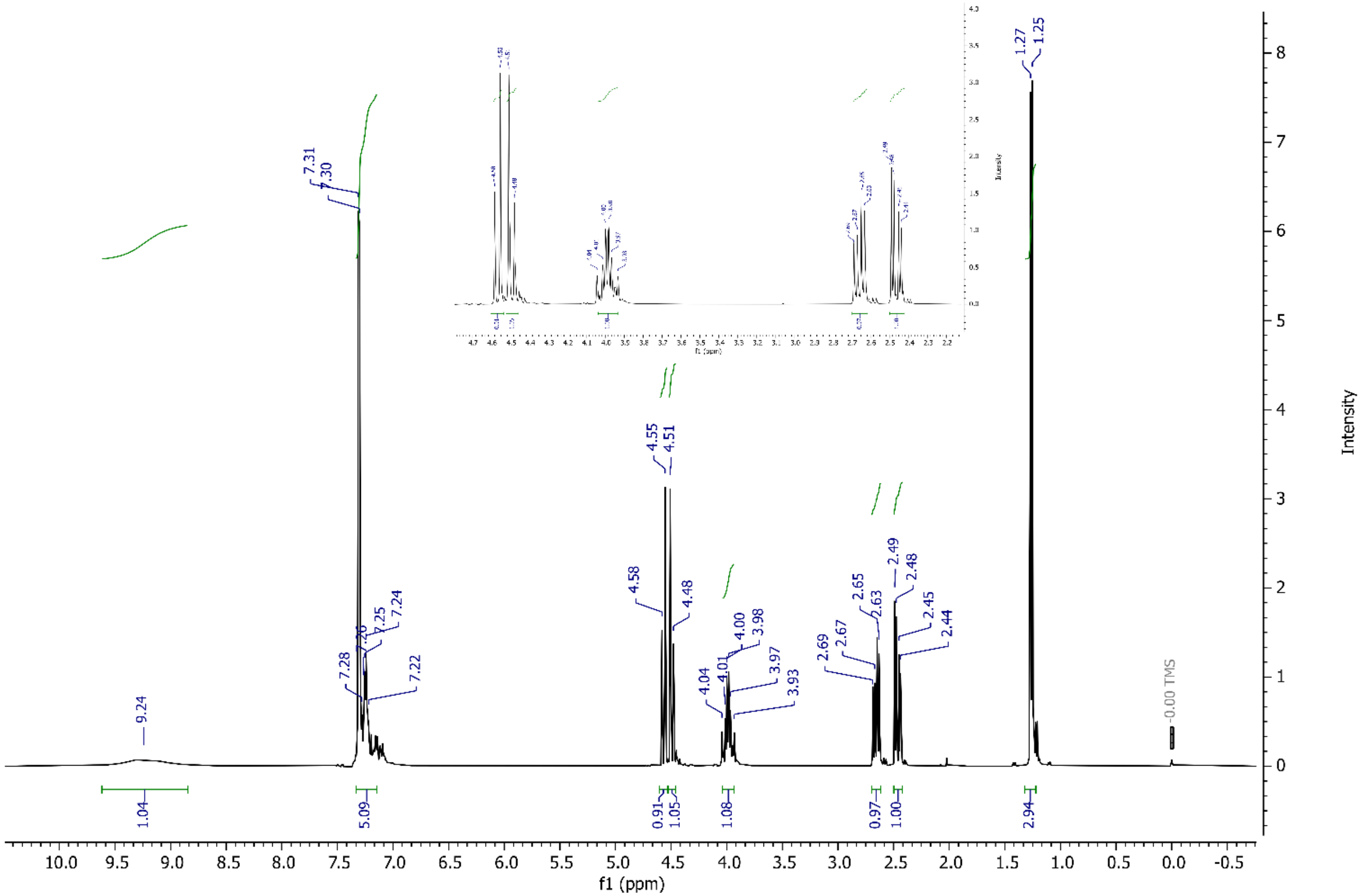

*Carbon NMR spectrum of (R)-3-Benzyloxybutanoic acid (**S9**)*

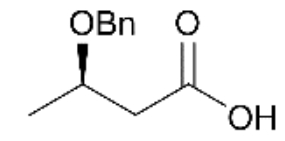

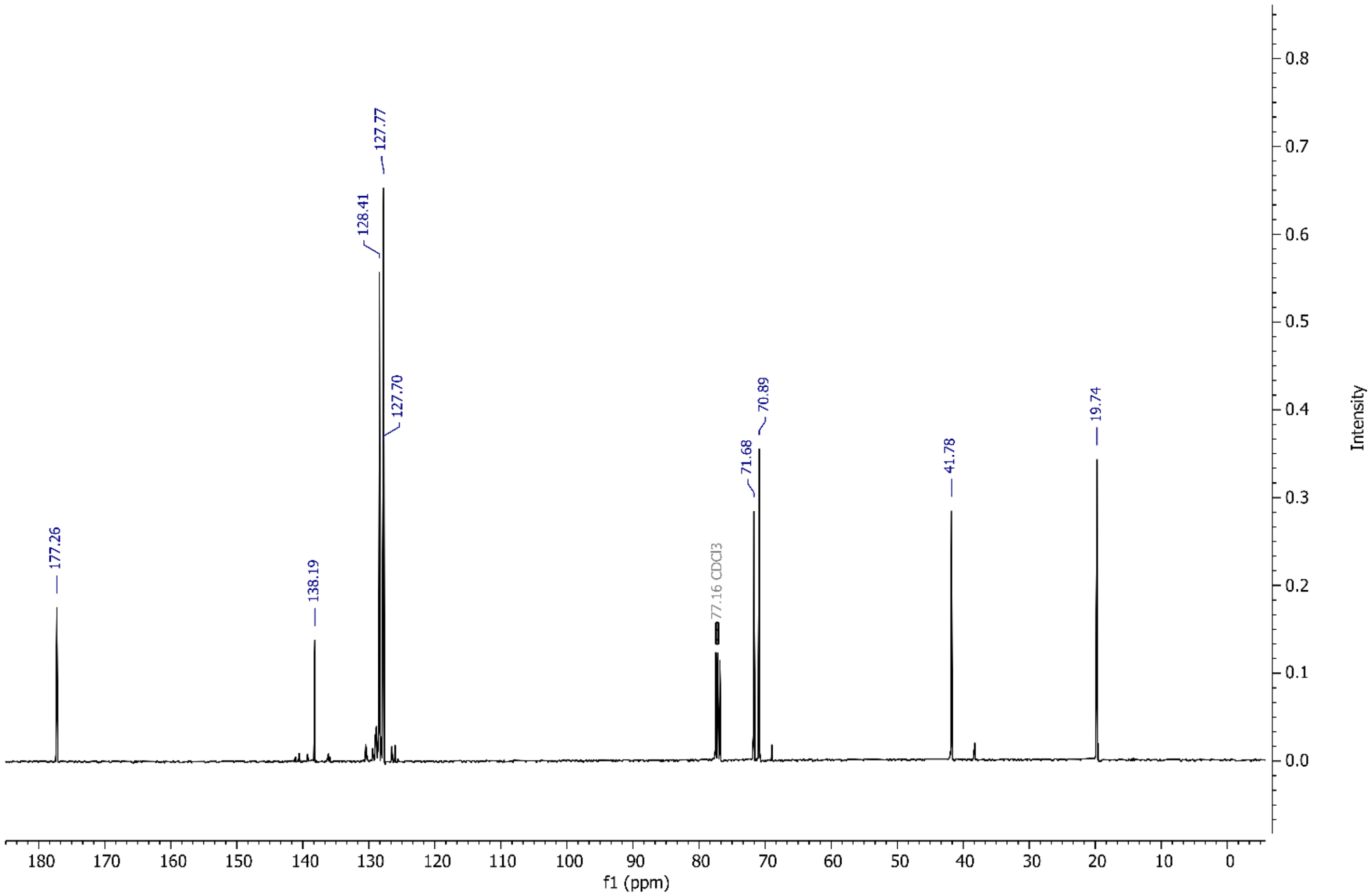

*Mass spectrum of (R)-3-Benzyloxybutanoic acid (**S9**)*

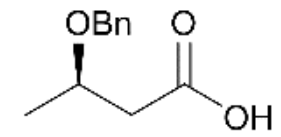

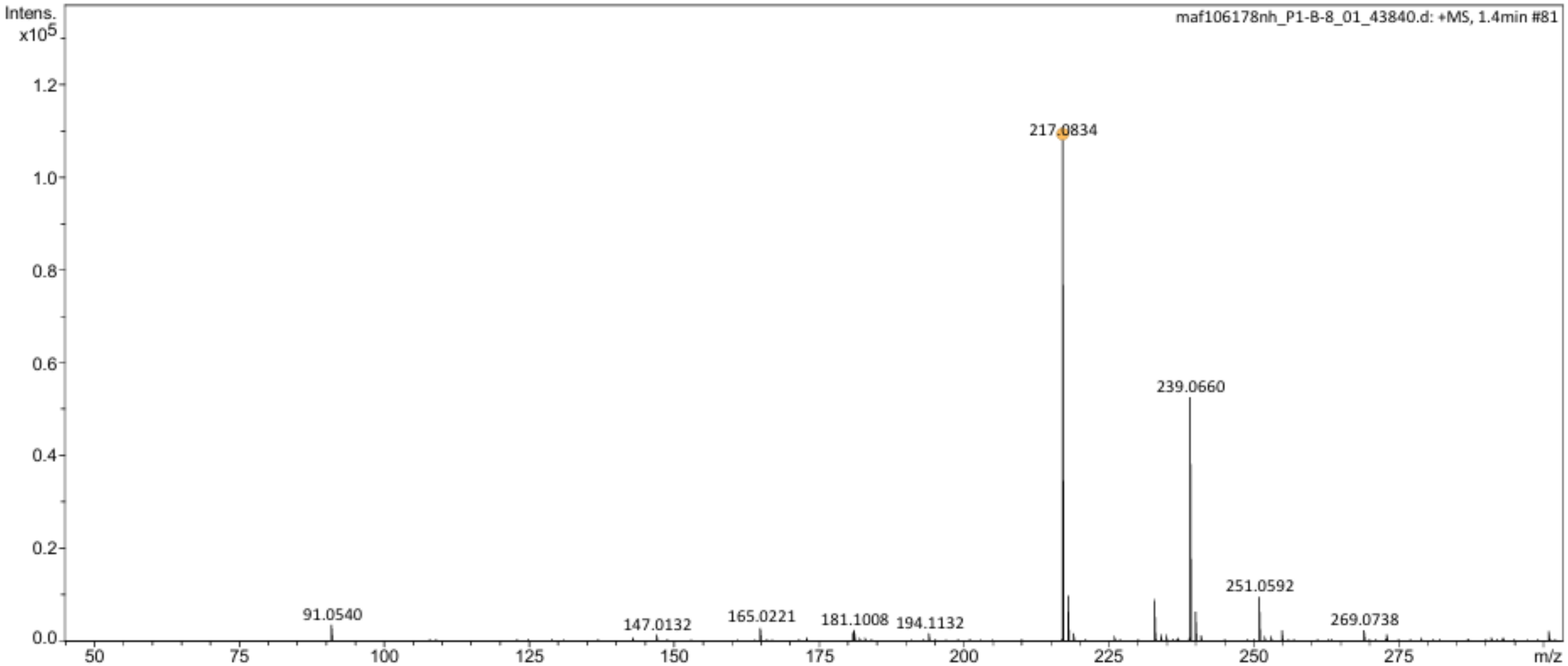

Meas. m/z	#	Ion Formula	m/z	err [ppm]	err [mDa]	mSigma	Mean err [ppm]
217.0834	1	C11H14NaO3	217.0835	0.5	0.1	17.0	-0.2

(*R*)-3-Benzyloxybutanoic anhydride (**S10**)

*Mass spectrum of (R)-3-Benzyloxybutanoic anhydride (**S10**)*

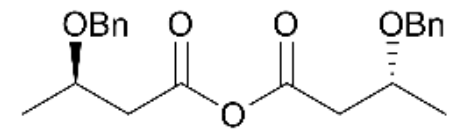

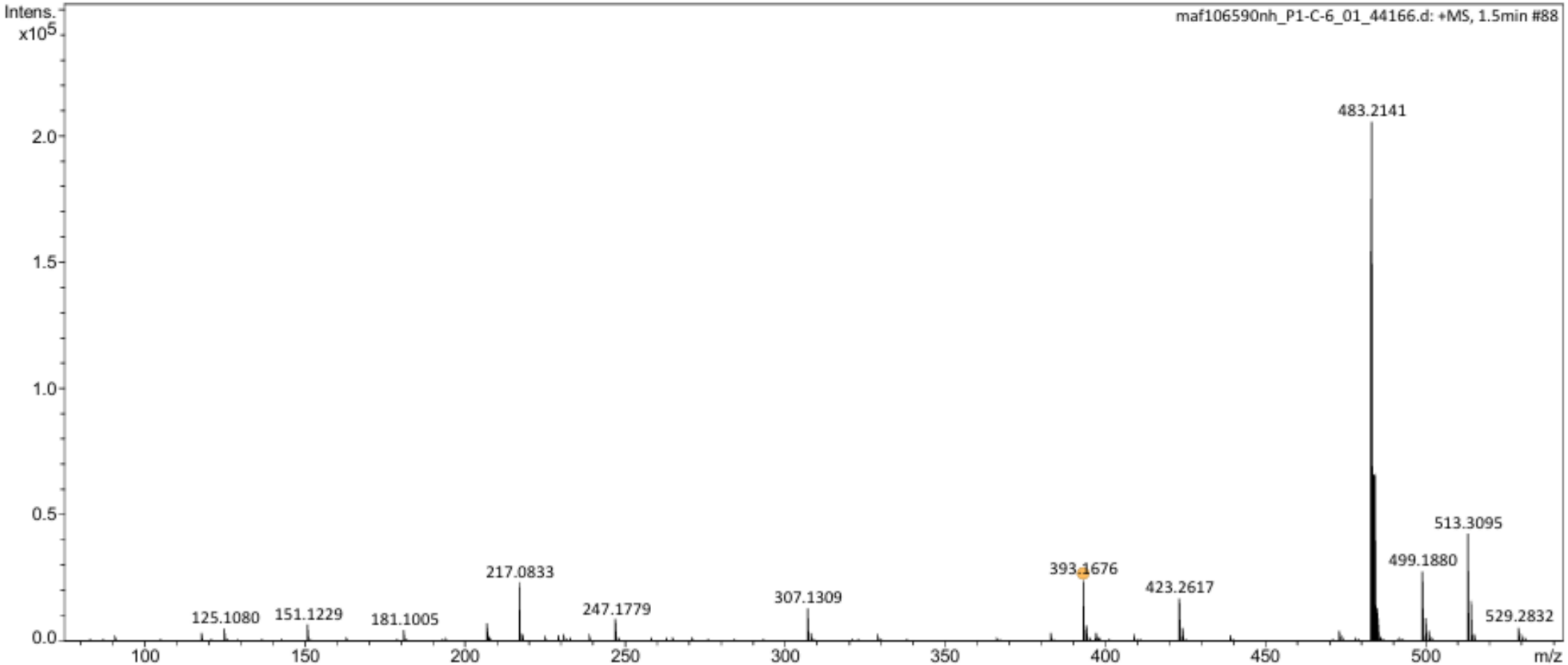

Meas. m/z	#	Ion Formula	m/z	err [ppm]	err [mDa]	mSigma	Mean err [ppm]
393.1676	1	C22H26NaO5	393.1672	-0.8	-0.3	12.8	-1.9

Pse5Ac7Ac

*Hydrogen NMR spectrum of Pse5Ac7Ac (**2**)*

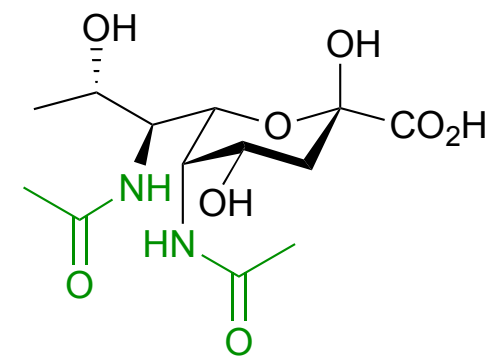

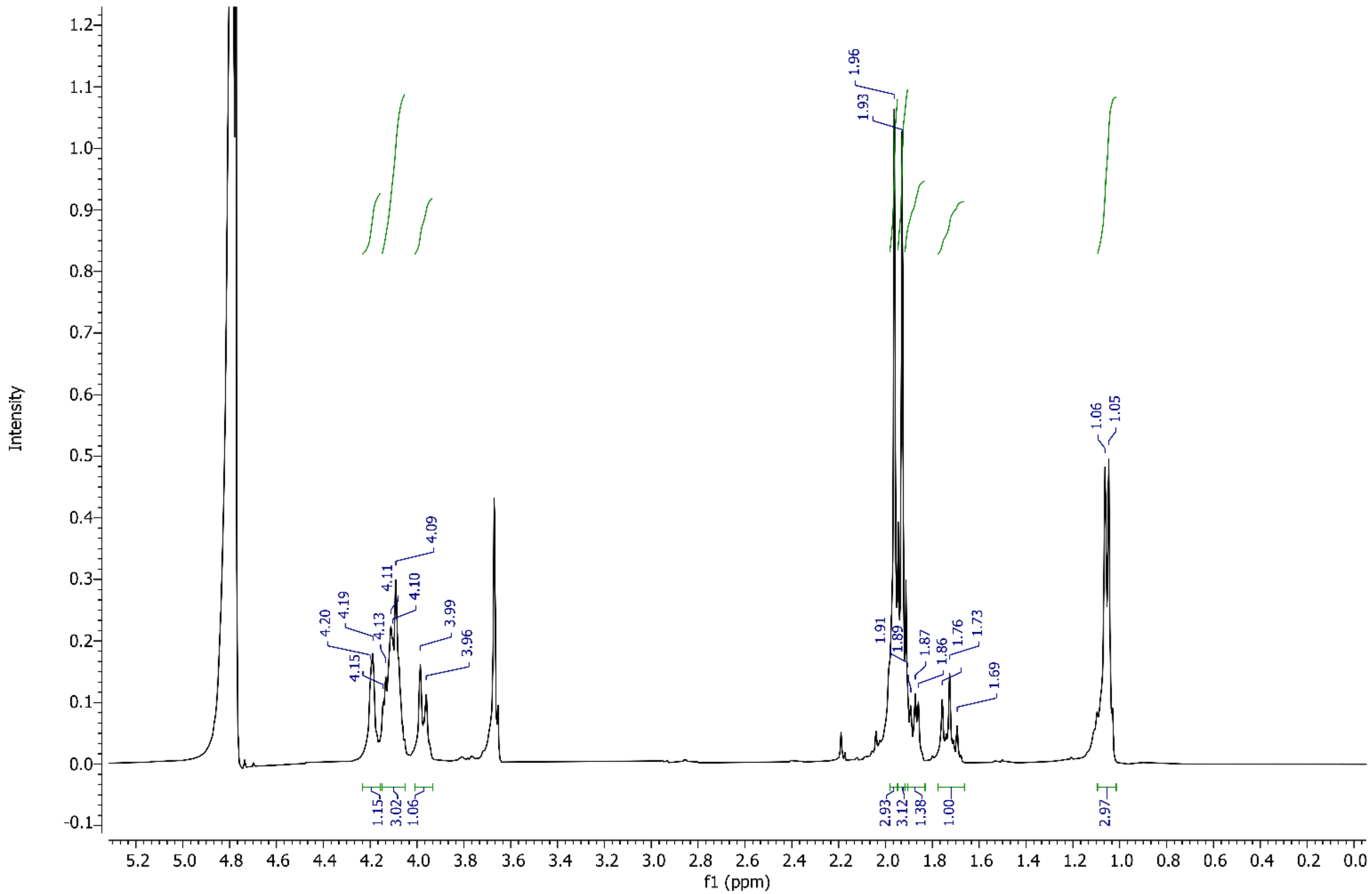

*Mass spectrum of Pse5Ac7Ac (**2**)*

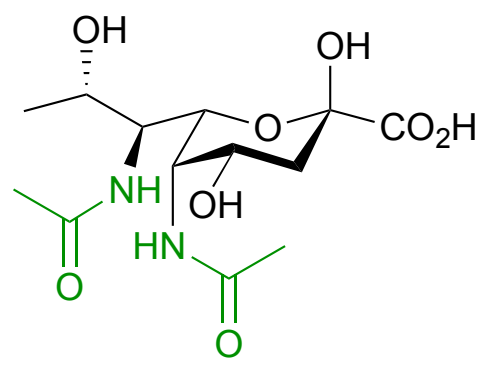

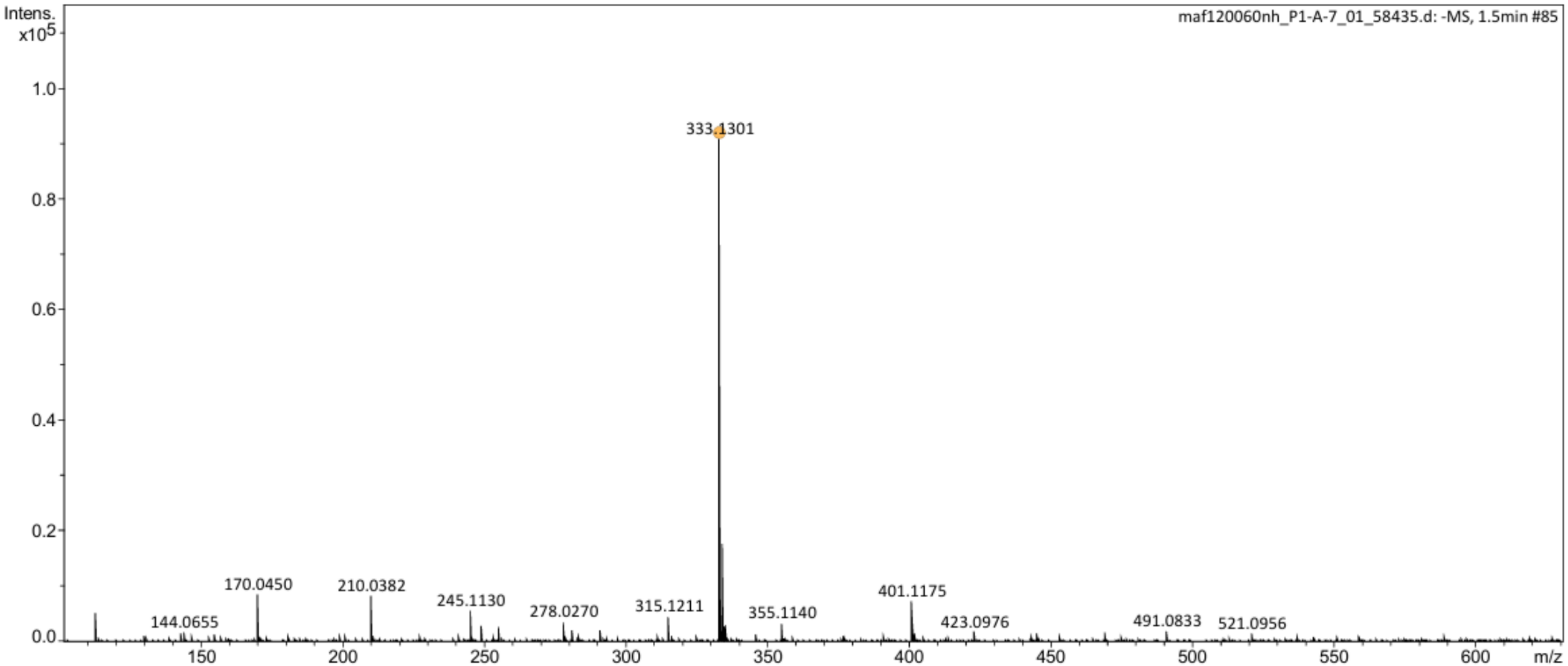

Meas. m/z	#	Ion Formula	m/z	err [ppm]	err [mDa]	mSigma	Mean err [ppm]
333.1301	1	C13H21N2O8	333.1303	0.6	0.2	24.0	1.7

Benzyl 2,4-diazido-2,4,6-trideoxy-α-L-altropyranoside (**8**)

*Hydrogen NMR spectrum of Benzyl 2,4-diazido-2,4,6-trideoxy-α-L-altropyranoside (**8**)*

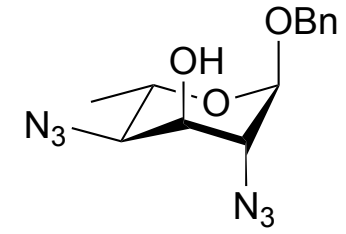

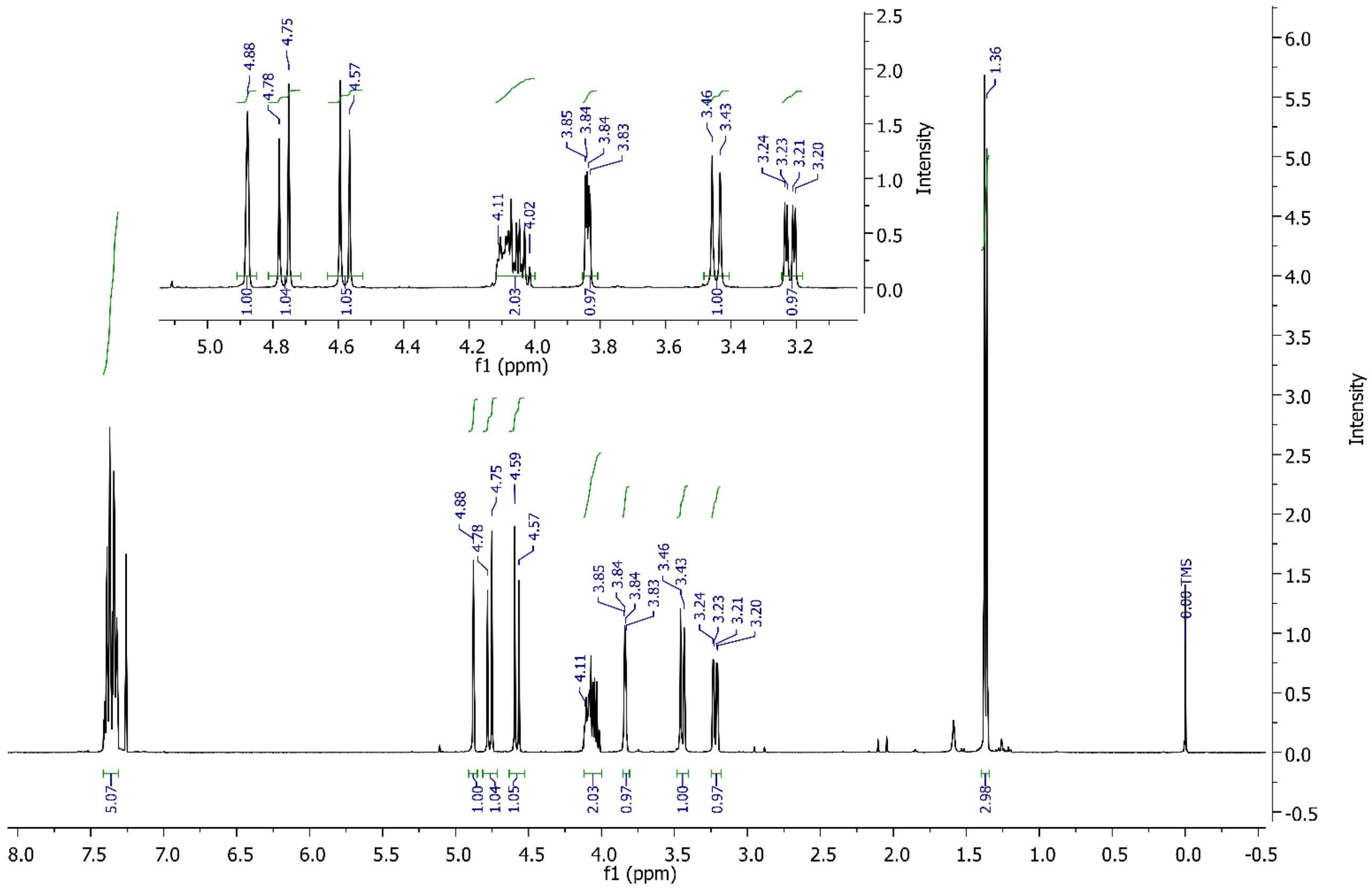

*Carbon NMR spectrum of Benzyl 2,4-diazido-2,4,6-trideoxy-α-L-altropyranoside (**8**)*

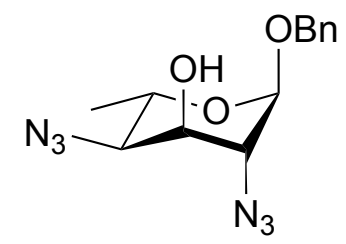

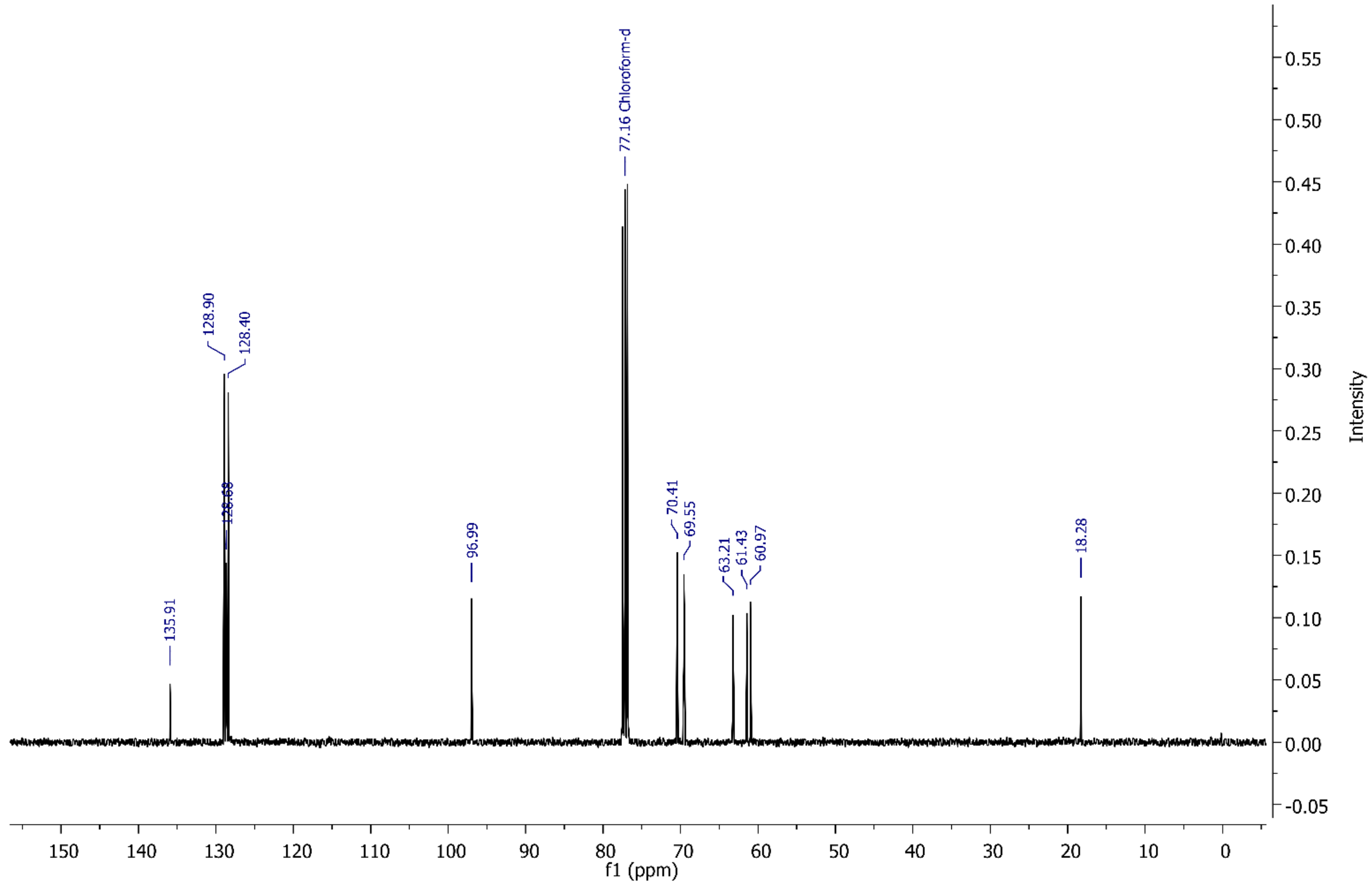

*Mass spectrum of Benzyl 2,4-diazido-2,4,6-trideoxy-α-L-altropyranoside (**8**)*

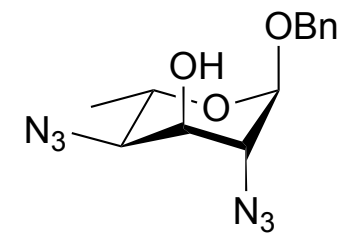

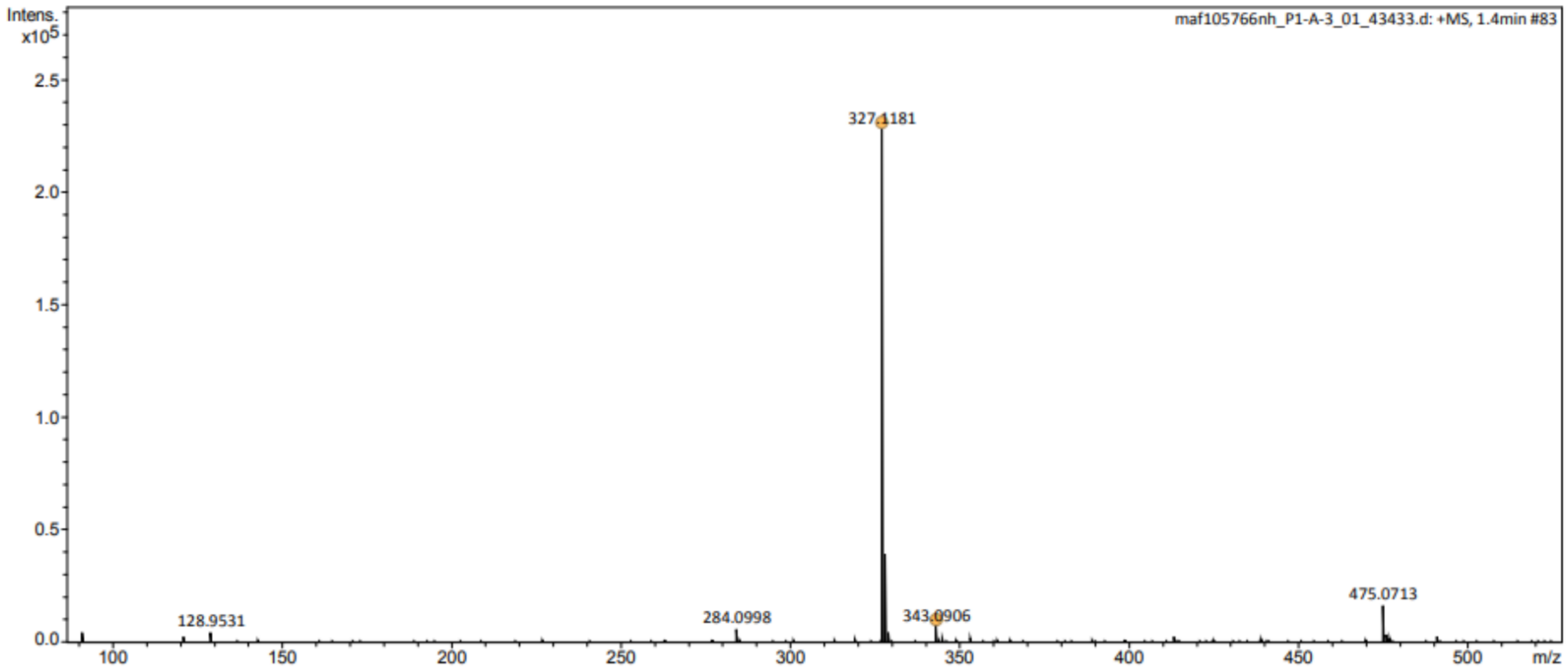

Meas. m/z	#	Ion Formula	m/z	err [ppm]	err [mDa]	mSigma	Mean err [ppm]
327.1181	1	C13H16N6NaO3	327.1176	-1.6	-0.5	4.4	-0.6
343.0906	1	C13H16KN6O3	343.0915	2.9	1.0	48.0	1.8

*IR spectrum of Benzyl 2,4-diazido-2,4,6-trideoxy-α-L-altropyranoside (**8**)*

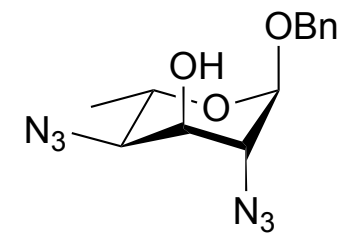

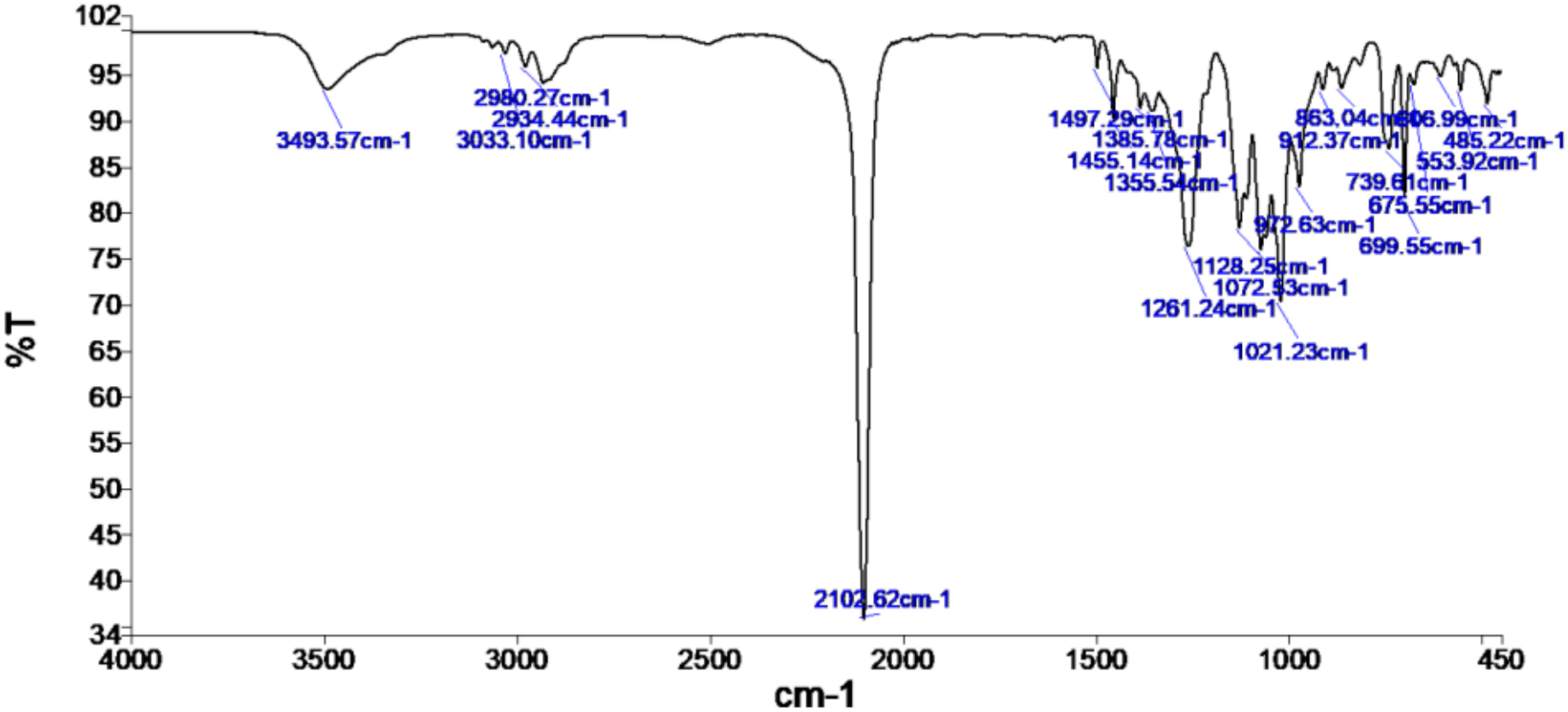

Benzyl 2,4-diazido-3-*O*-formyl-2,4,6-trideoxy-α-L-altropyranoside (**9**)

*Hydrogen NMR spectrum of benzyl 2,4-diazido-3-O-formyl-2,4,6-trideoxy-α-L-altropyranoside (**9**)*

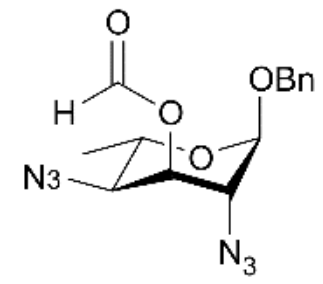

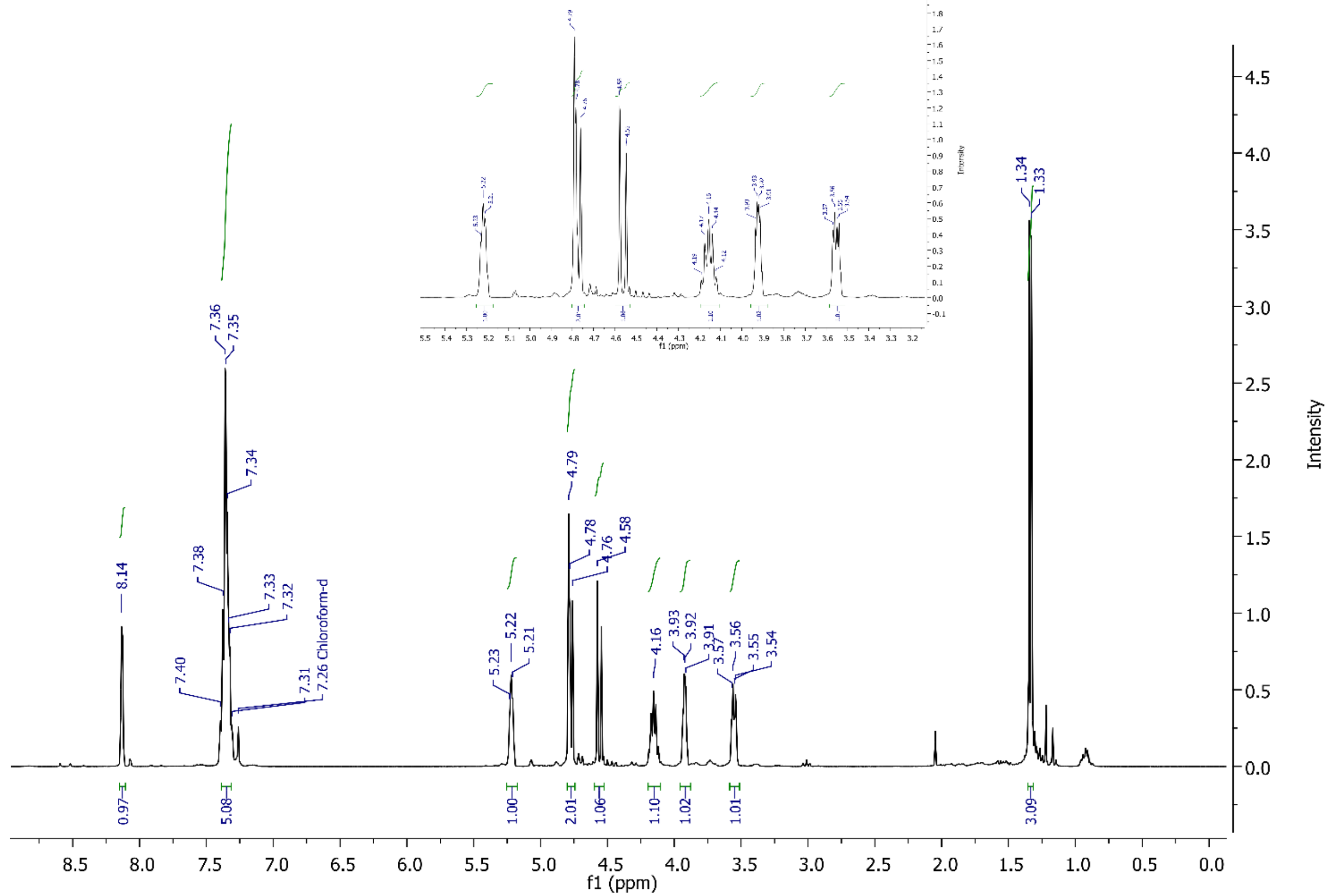

*Carbon NMR spectrum of benzyl 2,4-diazido-3-O-formyl-2,4,6-trideoxy-α-L-altropyranoside (**9**)*

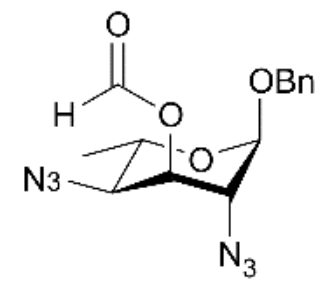

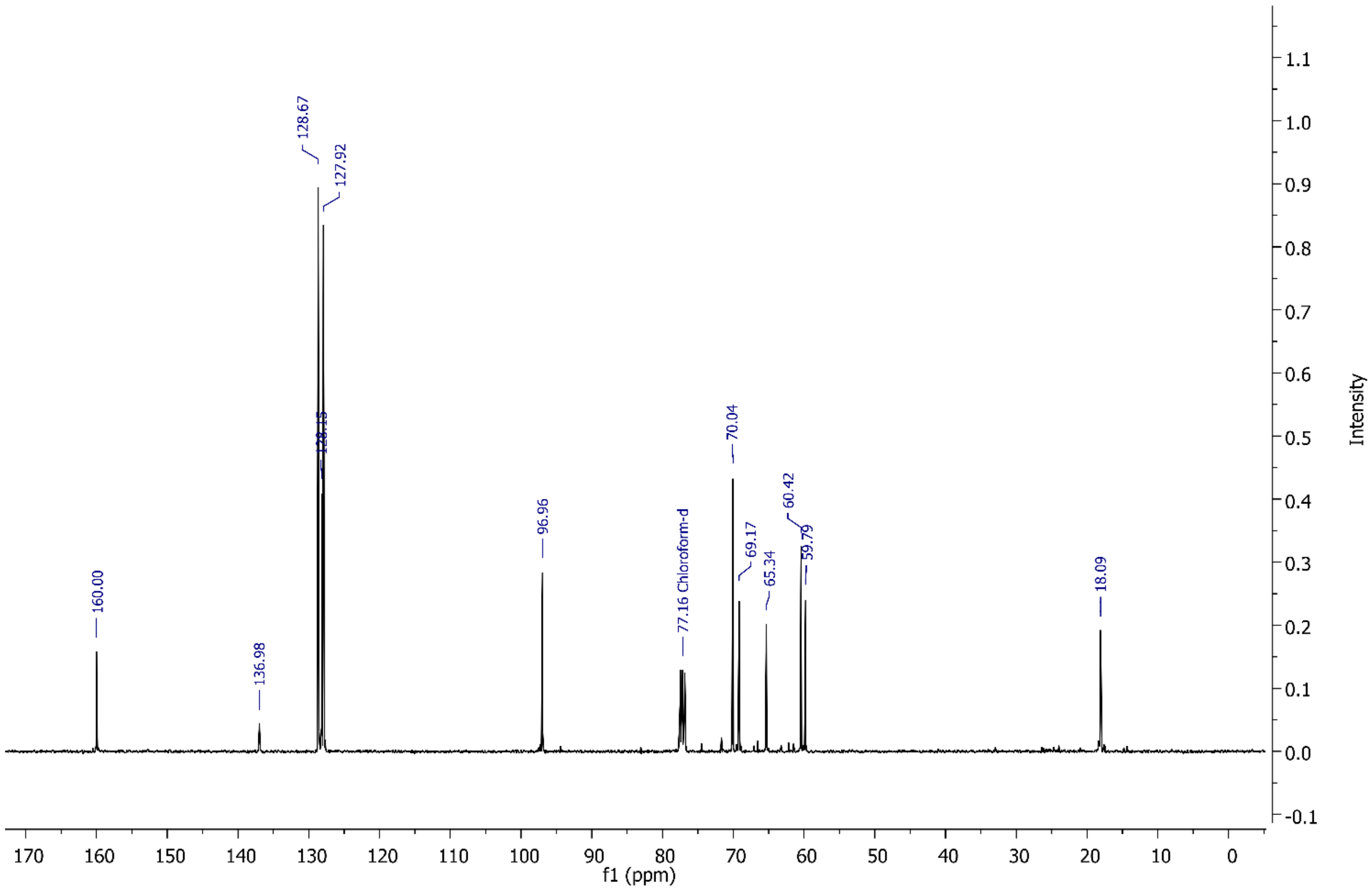

*Mass spectrum of benzyl 2,4-diazido-3-O-formyl-2,4,6-trideoxy-α-L-altropyranoside (**9**)*

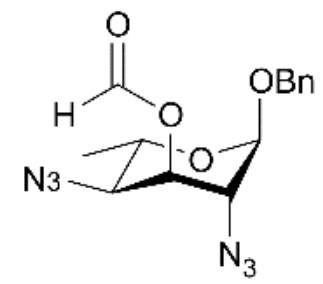

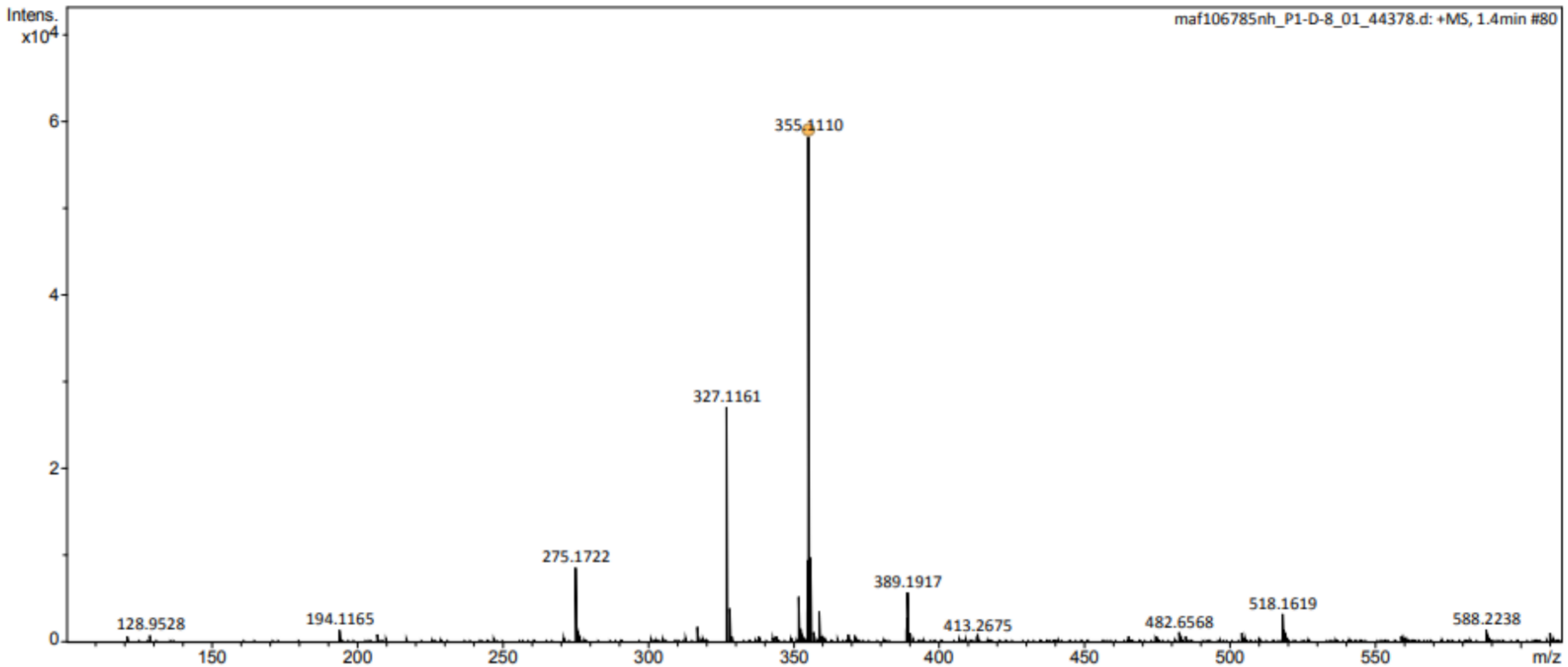

Meas. m/z	#	Ion Formula	m/z	err [ppm]	err [mDa]	mSigma	Mean err [ppm]
355.1110	1	C14H16N6NaO4	355.1125	4.2	1.5	6.4	3.1

*IR spectrum of benzyl 2,4-diazido-3-O-formyl-2,4,6-trideoxy-α-L-altropyranoside (**9**)*

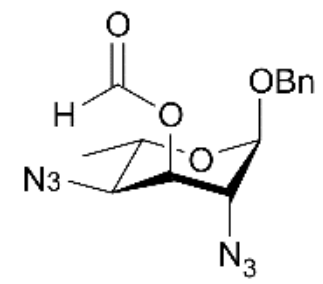

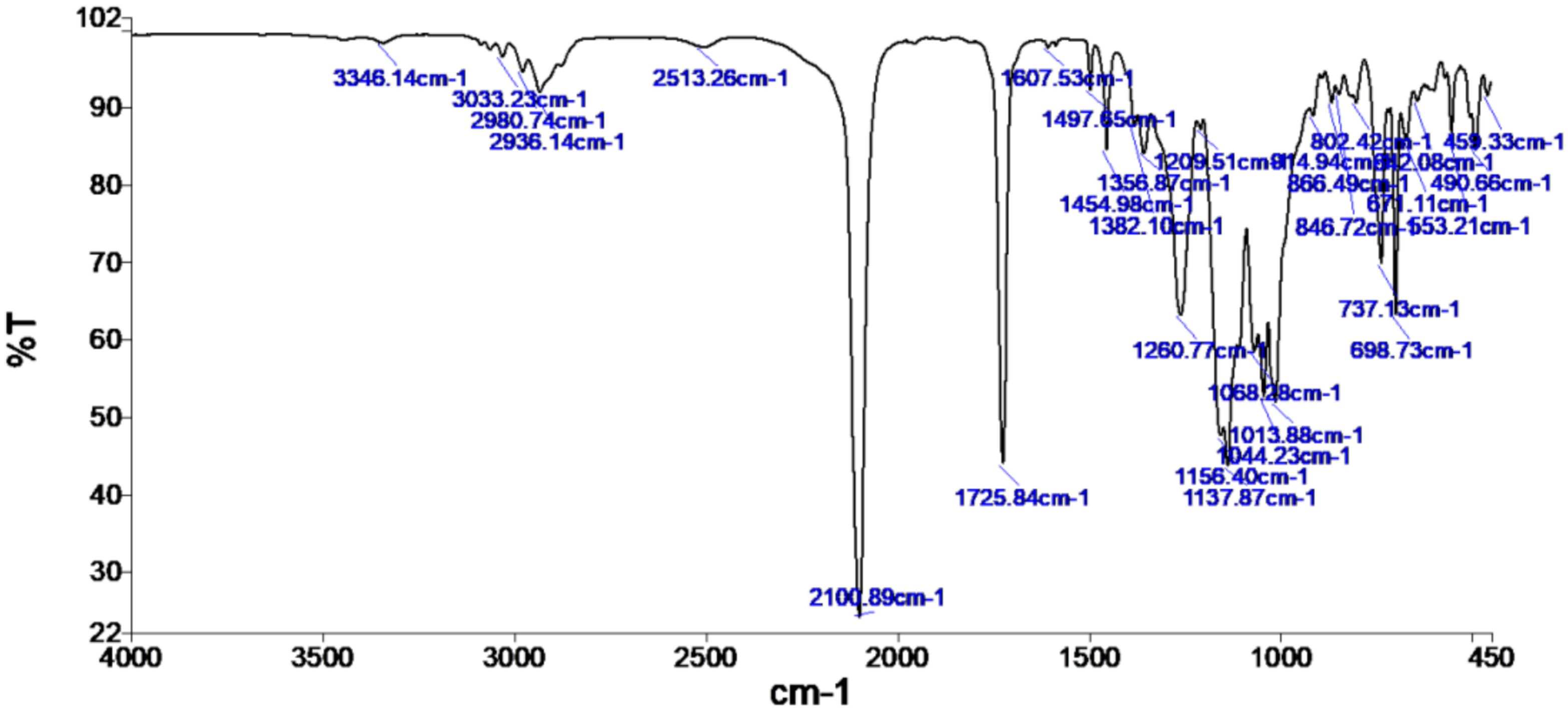

Benzyl 3-O-acetyl-2,4-diazido-2,4,6-trideoxy-α-l-altropyranoside (**10**)

*Hydrogen NMR spectrum of Benzyl 3-O-acetyl-2,4-diazido-2,4,6-trideoxy-α-L-altropyranoside (**10**)*

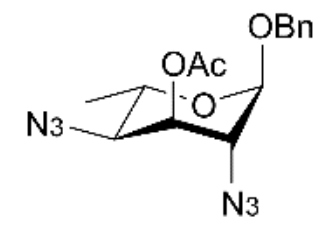

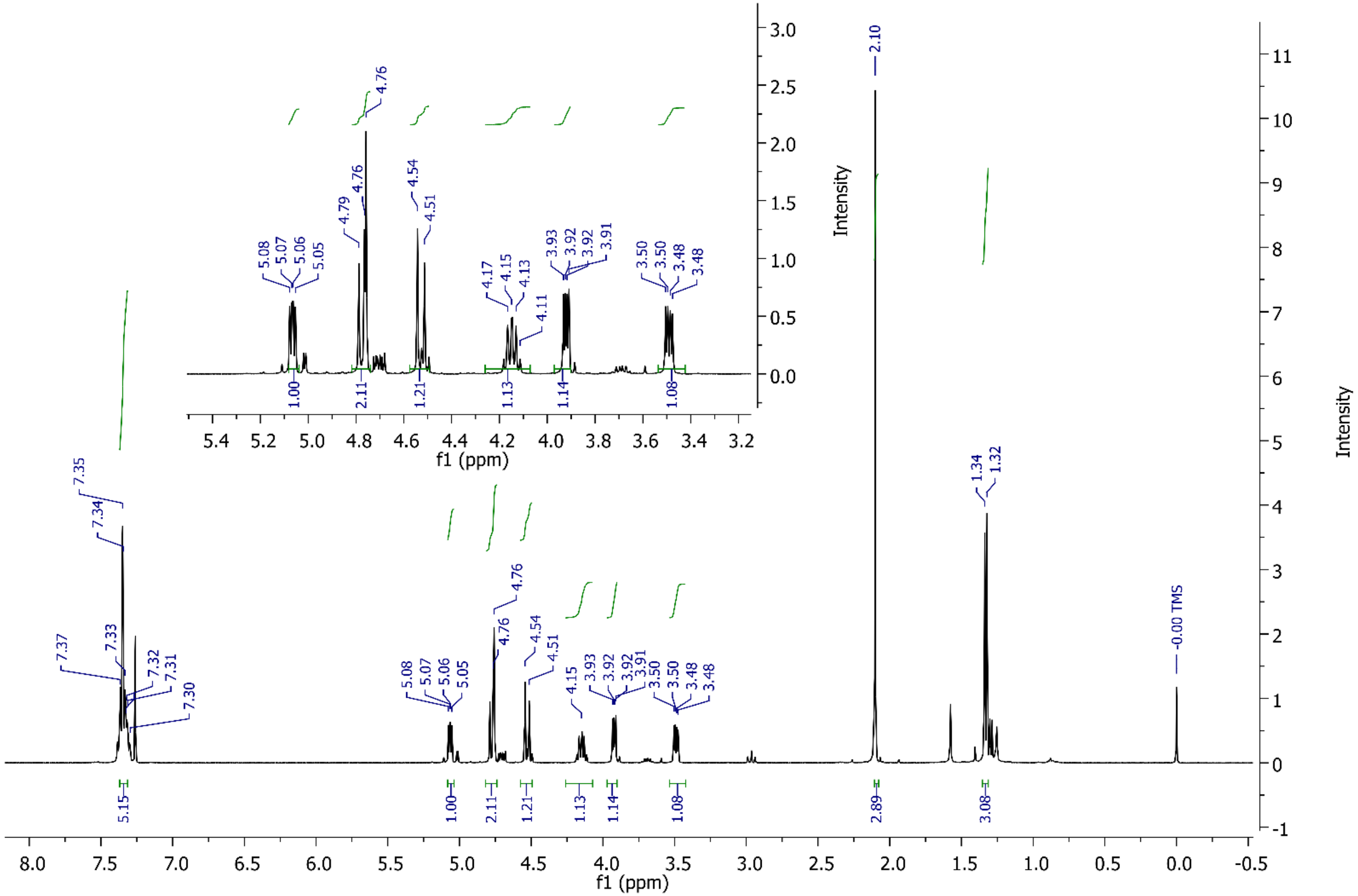

*Carbon NMR spectrum of Benzyl 3-O-acetyl-2,4-diazido-2,4,6-trideoxy-α-L-altropyranoside (**10**)*

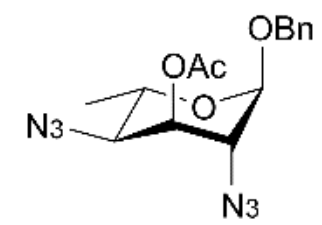

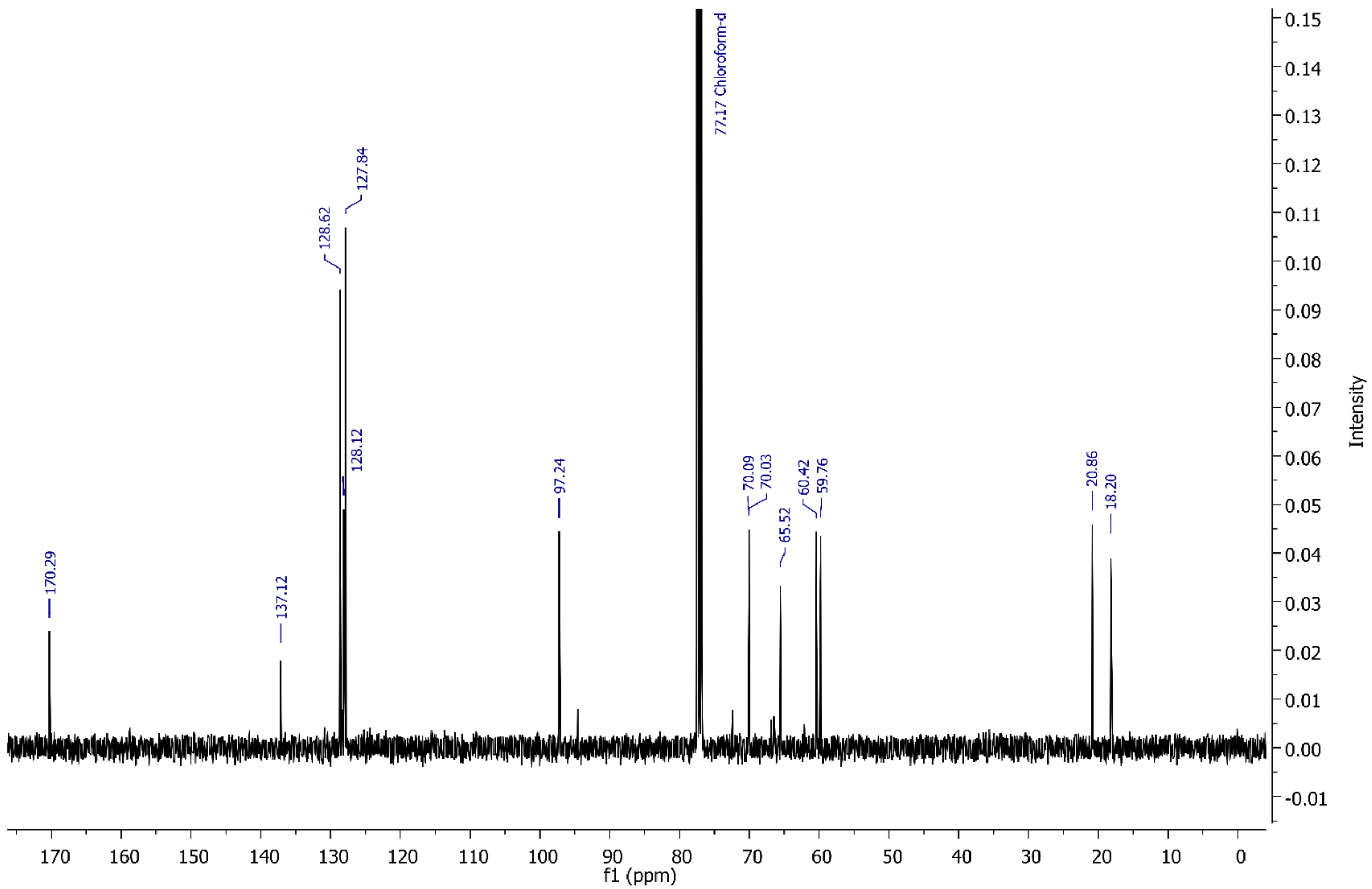

*Mass spectrum of Benzyl 3-O-acetyl-2,4-diazido-2,4,6-trideoxy-α-L-altropyranoside (**10**)*

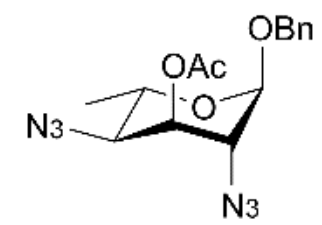

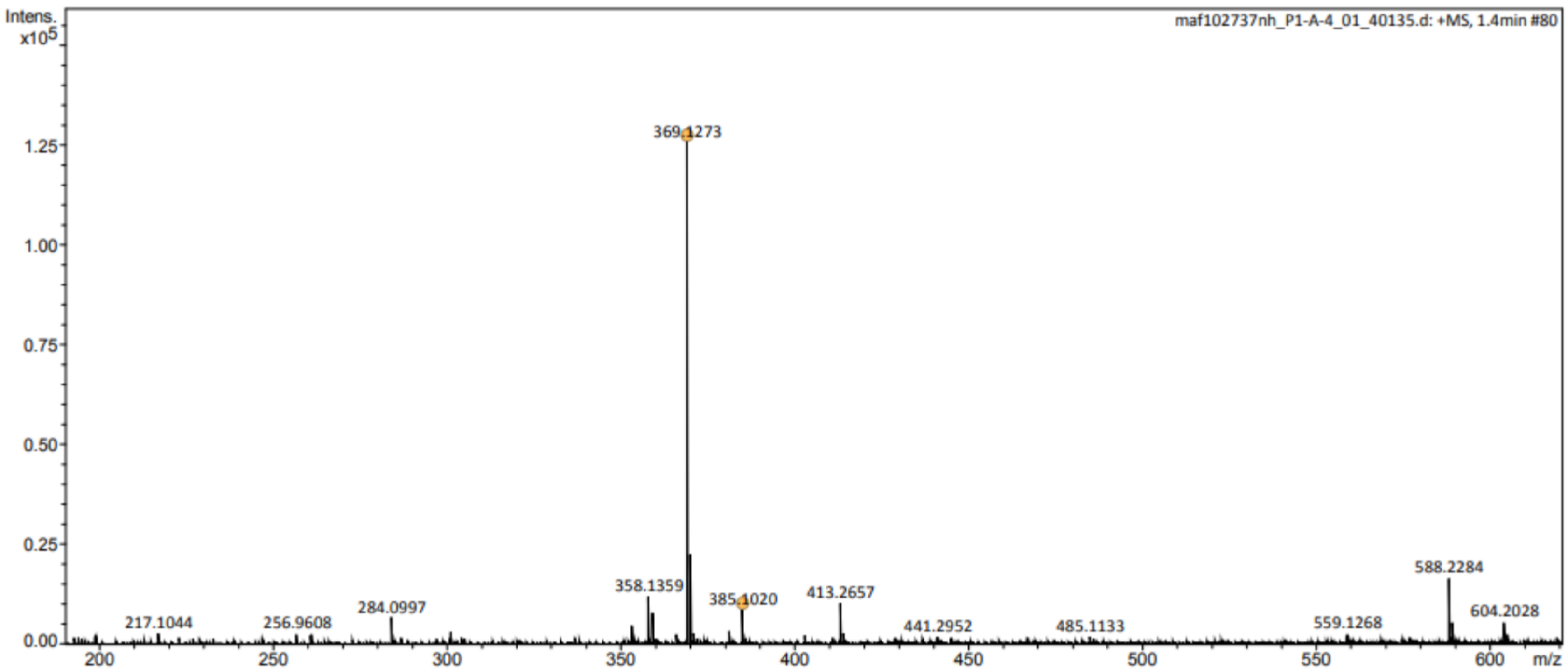

Meas. m/z	#	Ion Formula	m/z	err [ppm]	err [mDa]	mSigma	Mean err [ppm]
369.1273	1	C15H18N6NaO4	369.1282	2.4	0.9	15.3	1.8
385.1020	1	C15H18KN6O4	385.1021	0.2	0.1	12.5	1.9

*IR spectrum of Benzyl 3-O-acetyl-2,4-diazido-2,4,6-trideoxy-α-L-altropyranoside (**10**)*

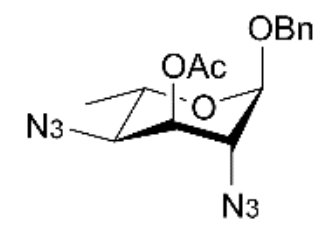

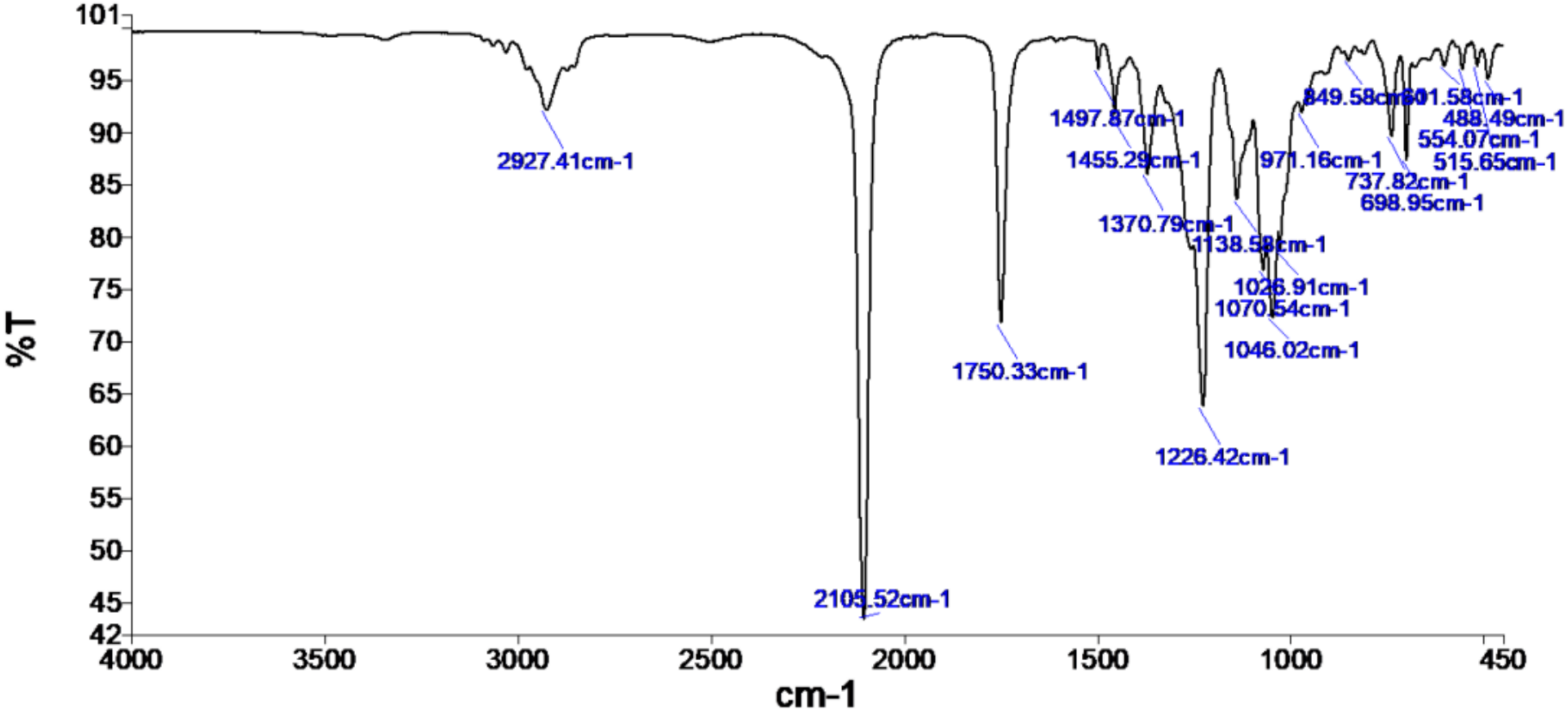

Benzyl 3-*O*-[(*R*)-3-(benzyloxy)butanoyl]-2,4-diazido-2,4,6-trideoxy-α-L-altropyranoside (**11**)

*Hydrogen NMR spectrum of benzyl 3-O-[(R)-3-(benzyloxy)butanoyl]-2,4-diazido-2,4,6-trideoxy-α-L-altropyranoside (**11**)*

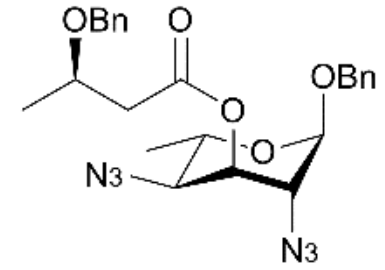

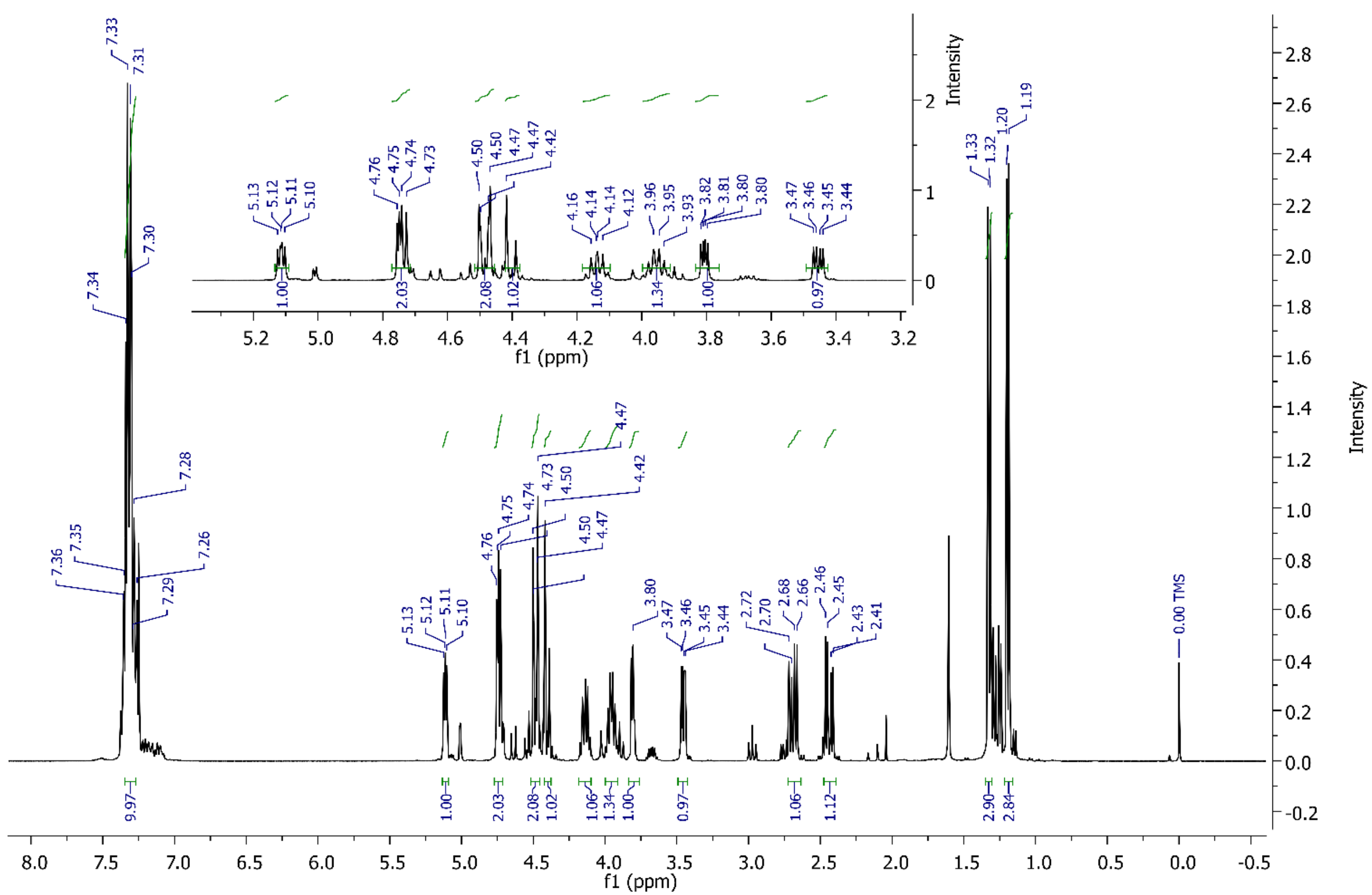

*Carbon NMR spectrum of benzyl 3-O-[(R)-3-(benzyloxy)butanoyl]-2,4-diazido-2,4,6-trideoxy-α-L-altropyranoside (**11**)*

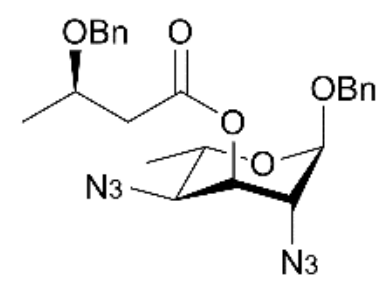

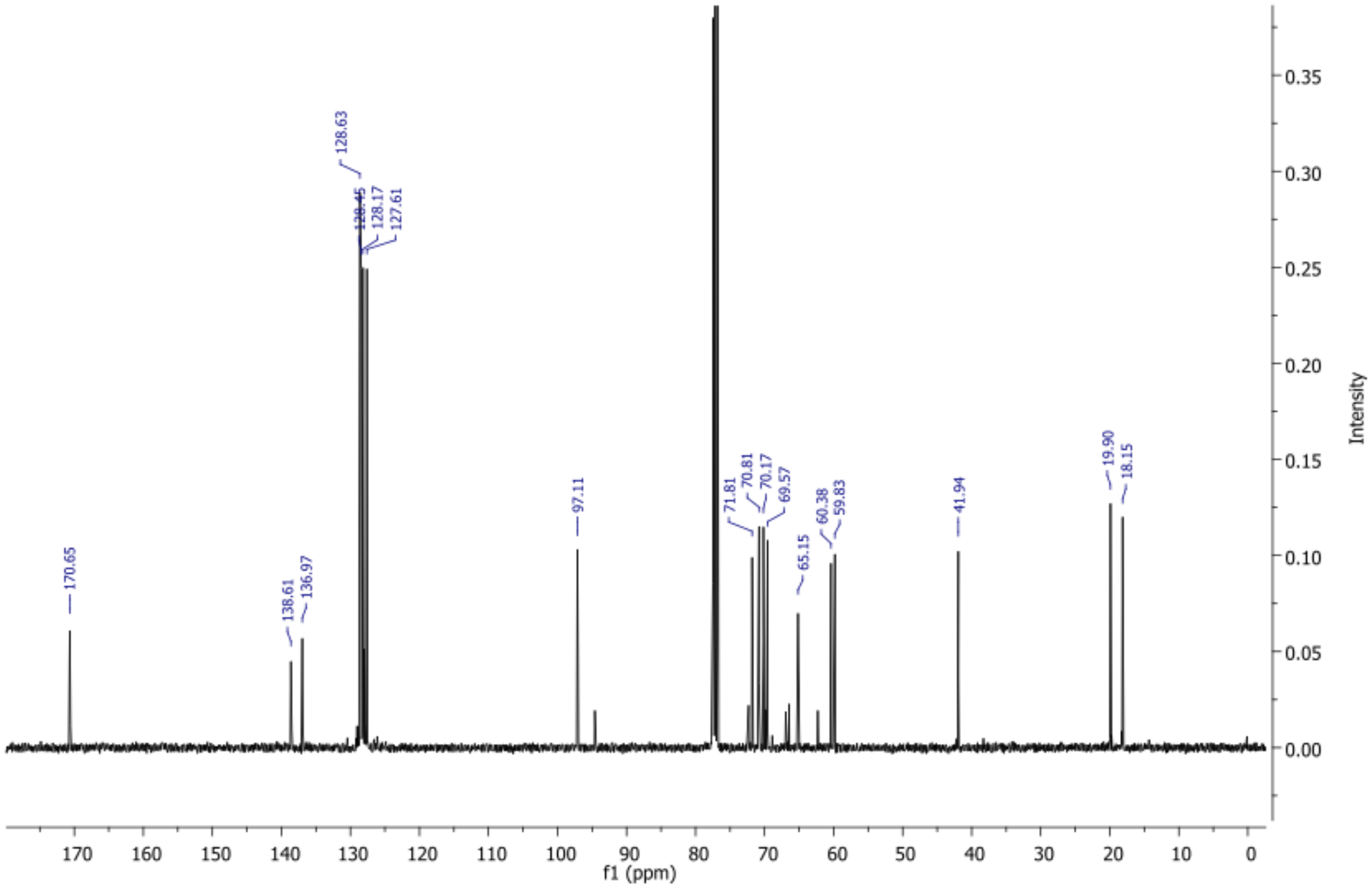

*Mass spectrum of benzyl 3-O-[(R)-3-(benzyloxy)butanoyl]-2,4-diazido-2,4,6-trideoxy-α-L-altropyranoside (**11**)*

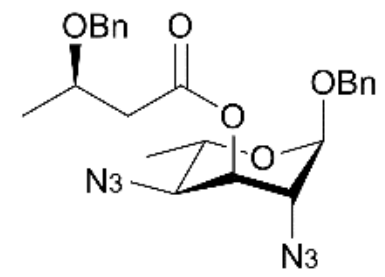

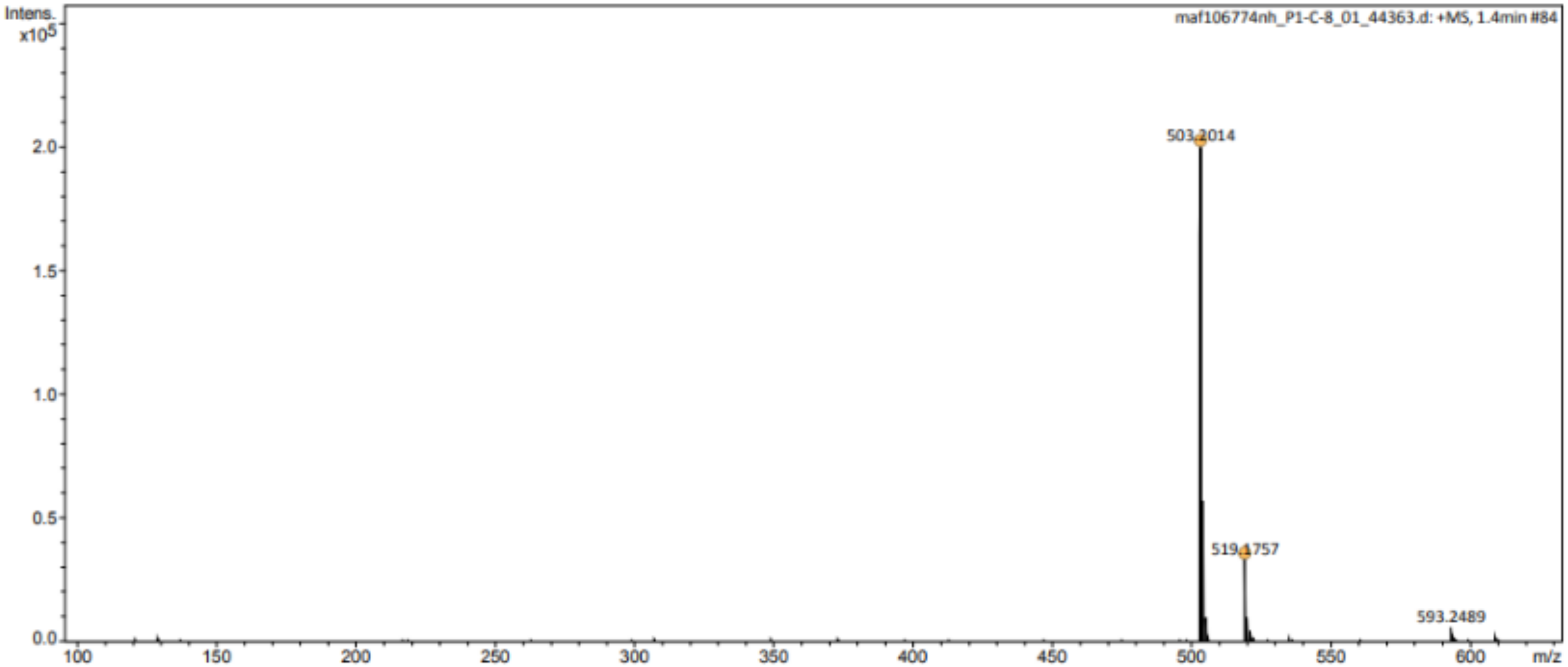

Meas. m/z	#	Ion Formula	m/z	err [ppm]	err [mDa]	mSigma	Mean err [ppm]
503.2014	1	C24H28N6NaO5	503.2013	-0.1	-0.1	0.9	-0.0
519.1757	1	C24H28KN6O5	519.1753	-0.8	-0.4	10.0	-2.8

*FT-IR (ATR) spectrum of benzyl 3-O-[(R)-3-(benzyloxy)butanoyl]-2,4-diazido-2,4,6-trideoxy-α-L-altropyranoside (**11**)*

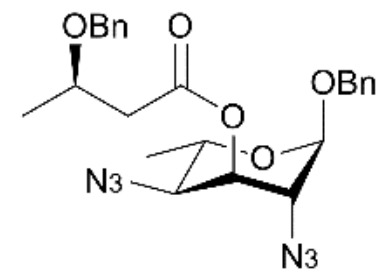

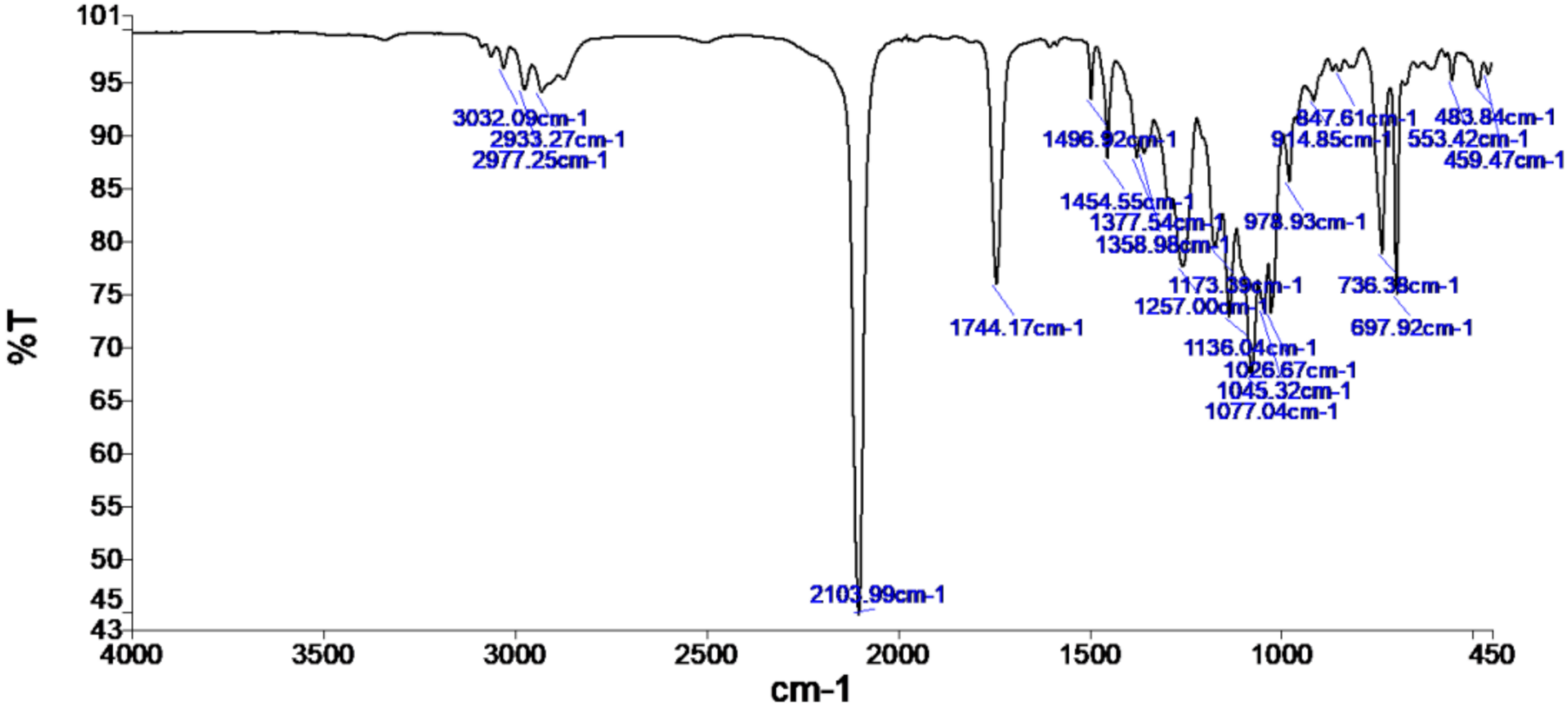

Benzyl 2-[(*R*)-3-(benzyloxy)butanamido]-2,4,6-trideoxy-4-formamido-α-L-altropyranoside (**12**)

*Hydrogen NMR spectrum of Benzyl 2-[(R)-3-(benzyloxy)butanamido]-2,4,6-trideoxy-4-formamido-α-L-altropyranoside (**12**)*

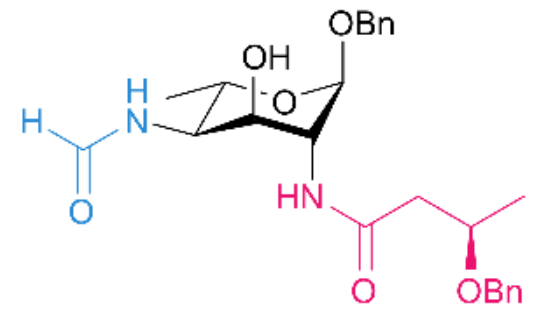

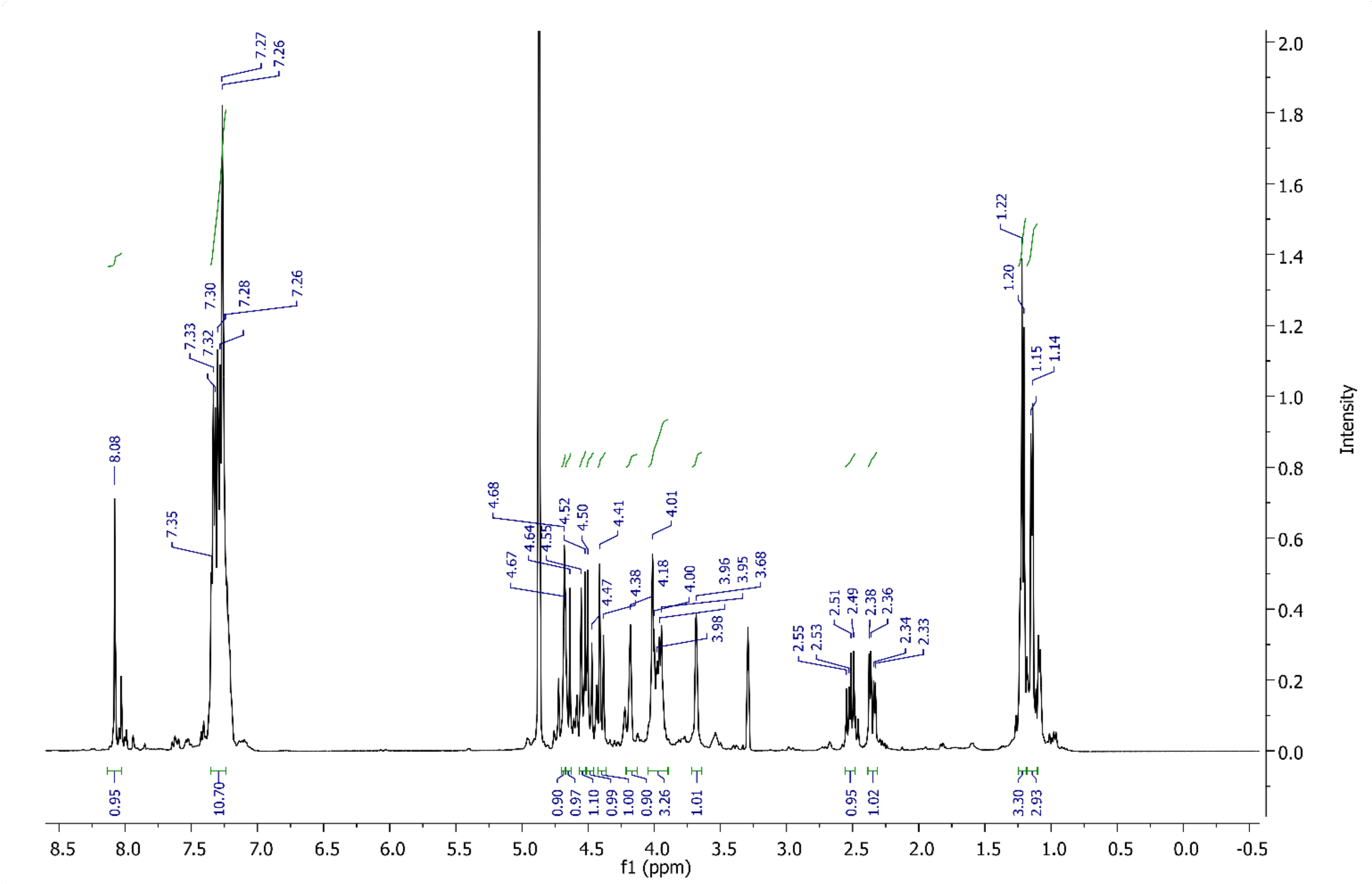

*Carbon NMR spectrum of Benzyl 2-[(R)-3-(benzyloxy)butanamido]-2,4,6-trideoxy-4-formamido-α-L-altropyranoside (**12**)*

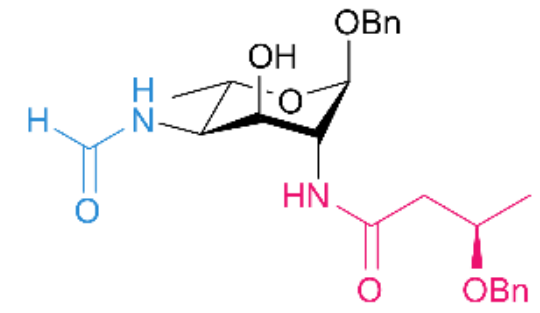

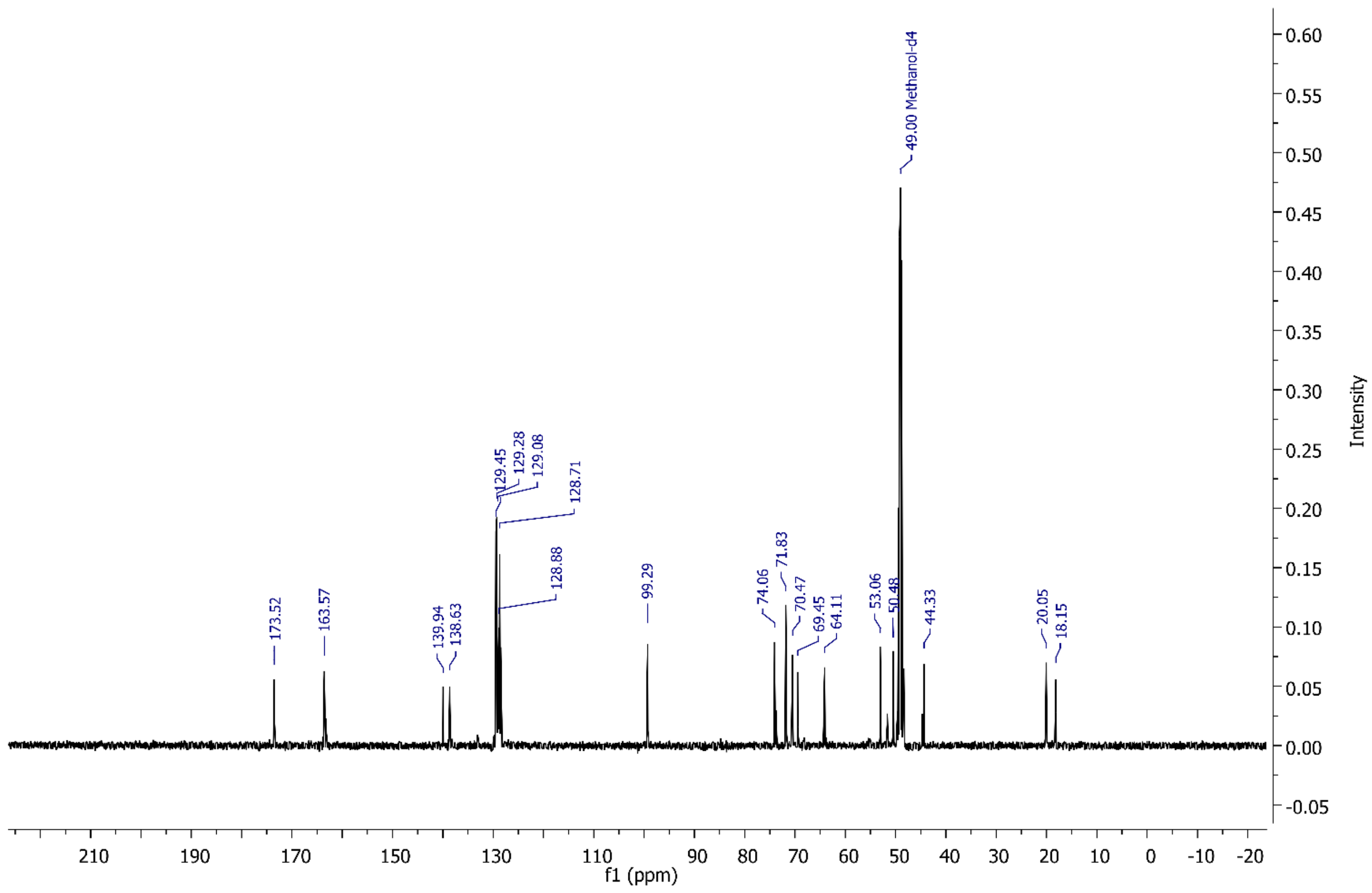

*Mass spectrum of Benzyl 2-[(R)-3-(benzyloxy)butanamido]-2,4,6-trideoxy-4-formamido-α-L-altropyranoside (**12**)*

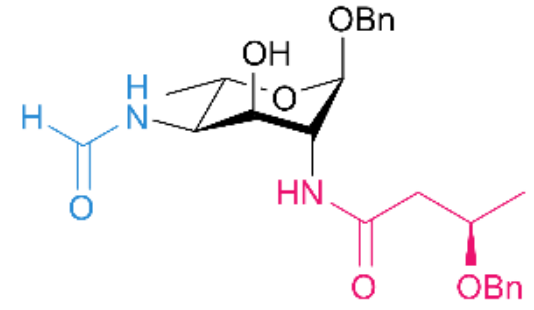

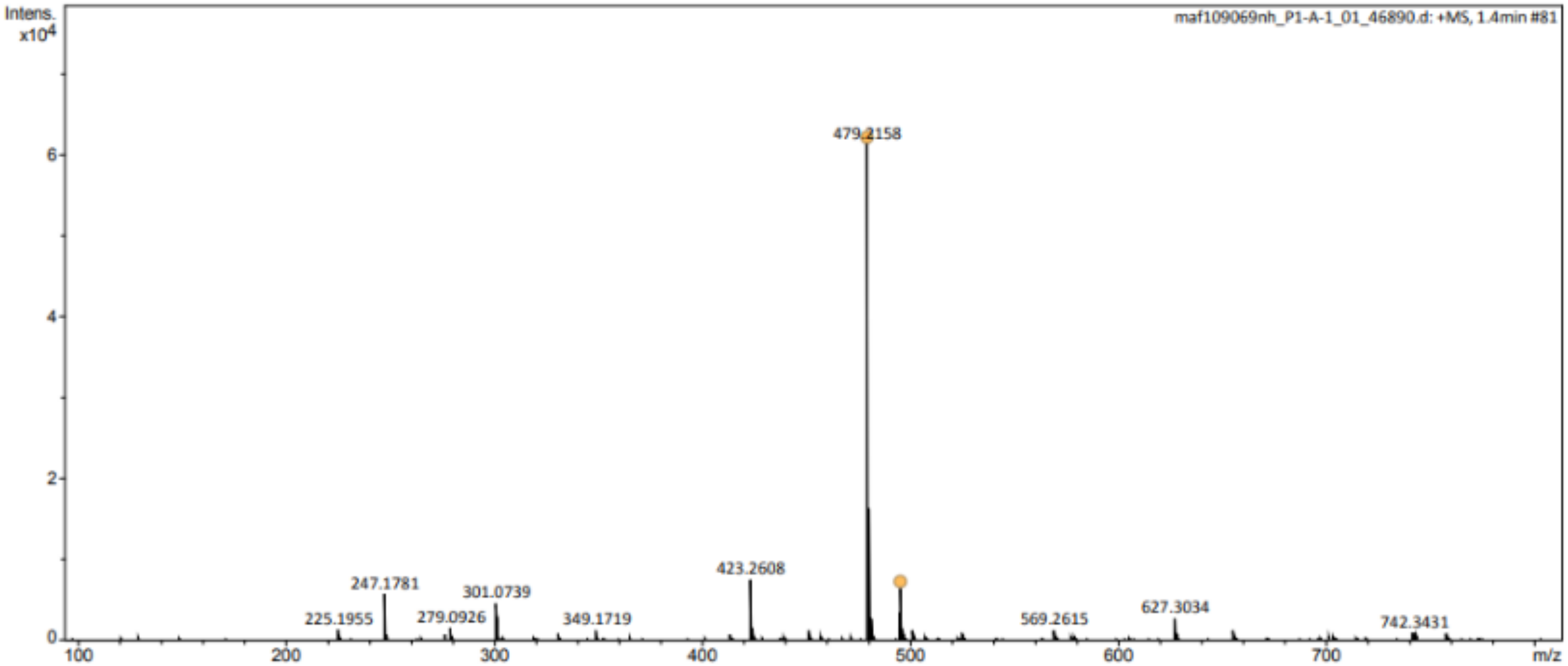

Meas. m/z	#	Ion Formula	m/z	err [ppm]	err [mDa]	mSigma	Mean err [ppm]
479.2158	1	C25H32N2NaO6	479.2153	-1.1	-0.5	9.9	-0.5
495.1884	1	C25H32KN2O6	495.1892	1.7	0.8	31.3	-2.0

*FT-IR (ATR) spectrum of Benzyl 2-[(R)-3-(benzyloxy)butanamido]-2,4,6-trideoxy-4-formamido-α-L-altropyranoside (**12**)*

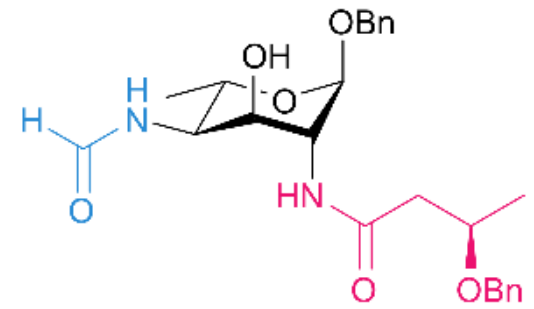

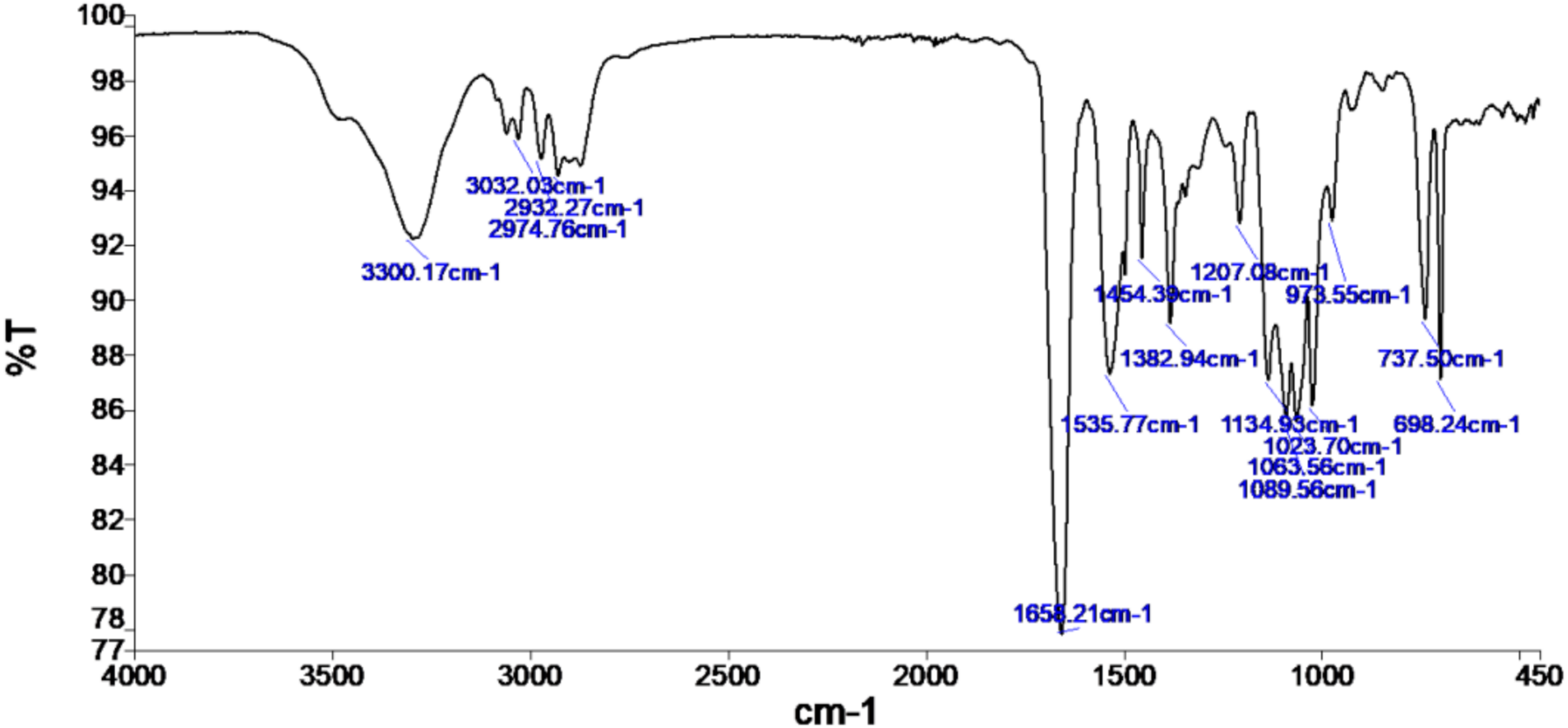

Benzyl 2-acetamido-2,4,6-trideoxy-4-formamido-α-L-altropyranoside (**13**)

*Hydrogen NMR spectrum of benzyl 2-acetamido-2,4,6-trideoxy-4-formamido-α-L-altropyranoside (**13**)*

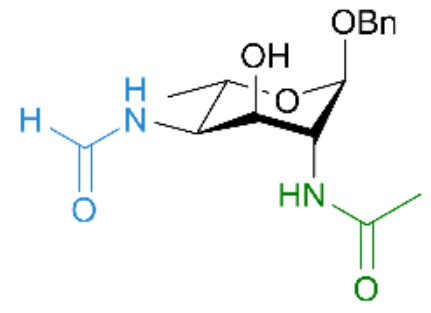

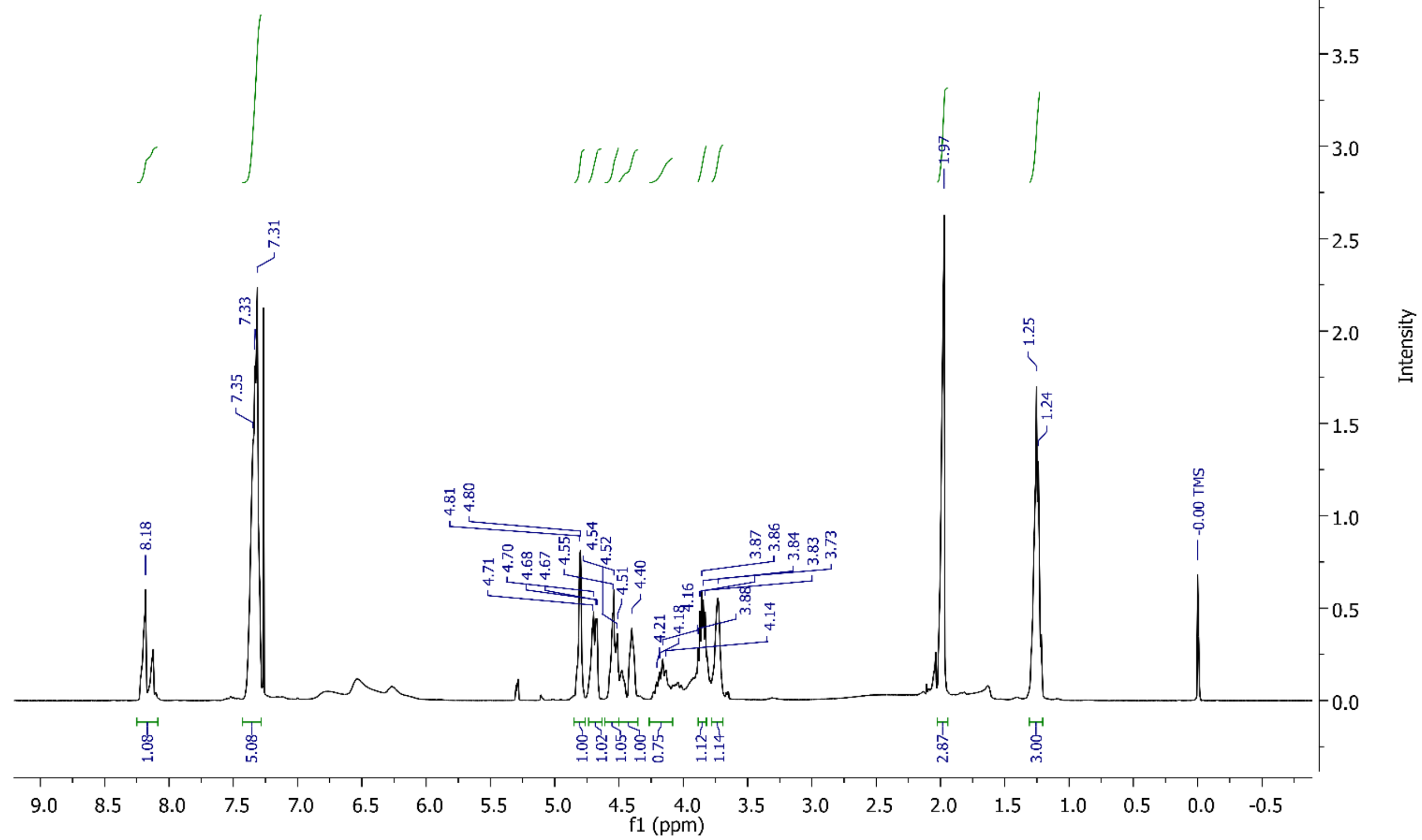

*Carbon NMR spectrum of benzyl 2-acetamido-2,4,6-trideoxy-4-formamido-α-L-altropyranoside (**13**)*

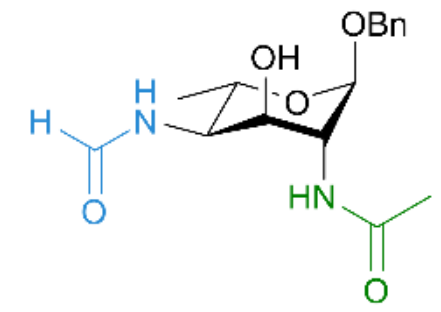

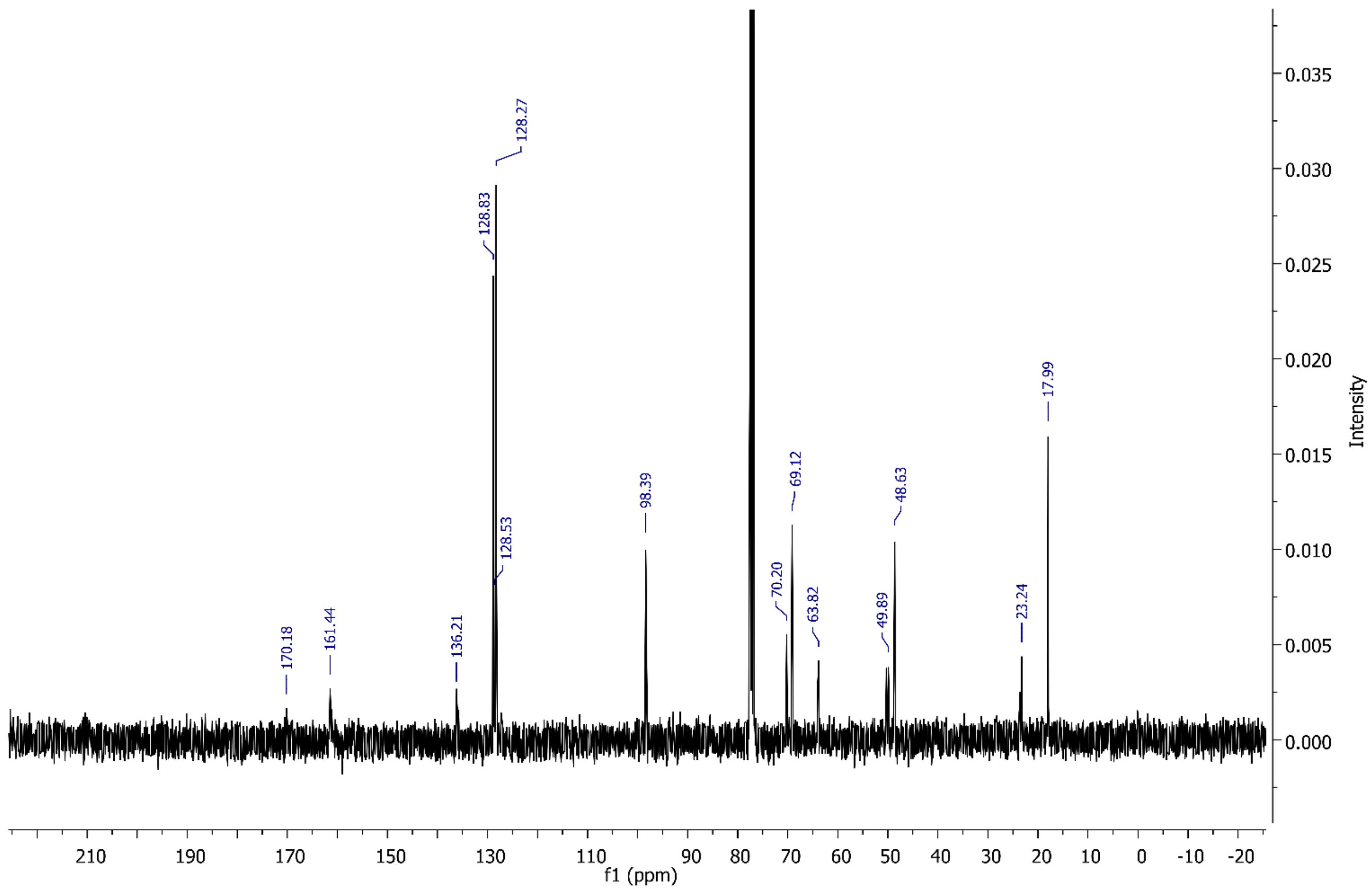

*Mass spectrum of benzyl 2-acetamido-2,4,6-trideoxy-4-formamido-α-L-altropyranoside (**13**)*

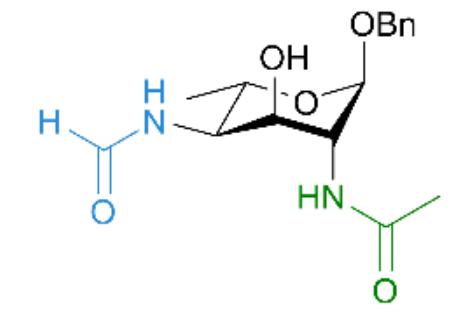

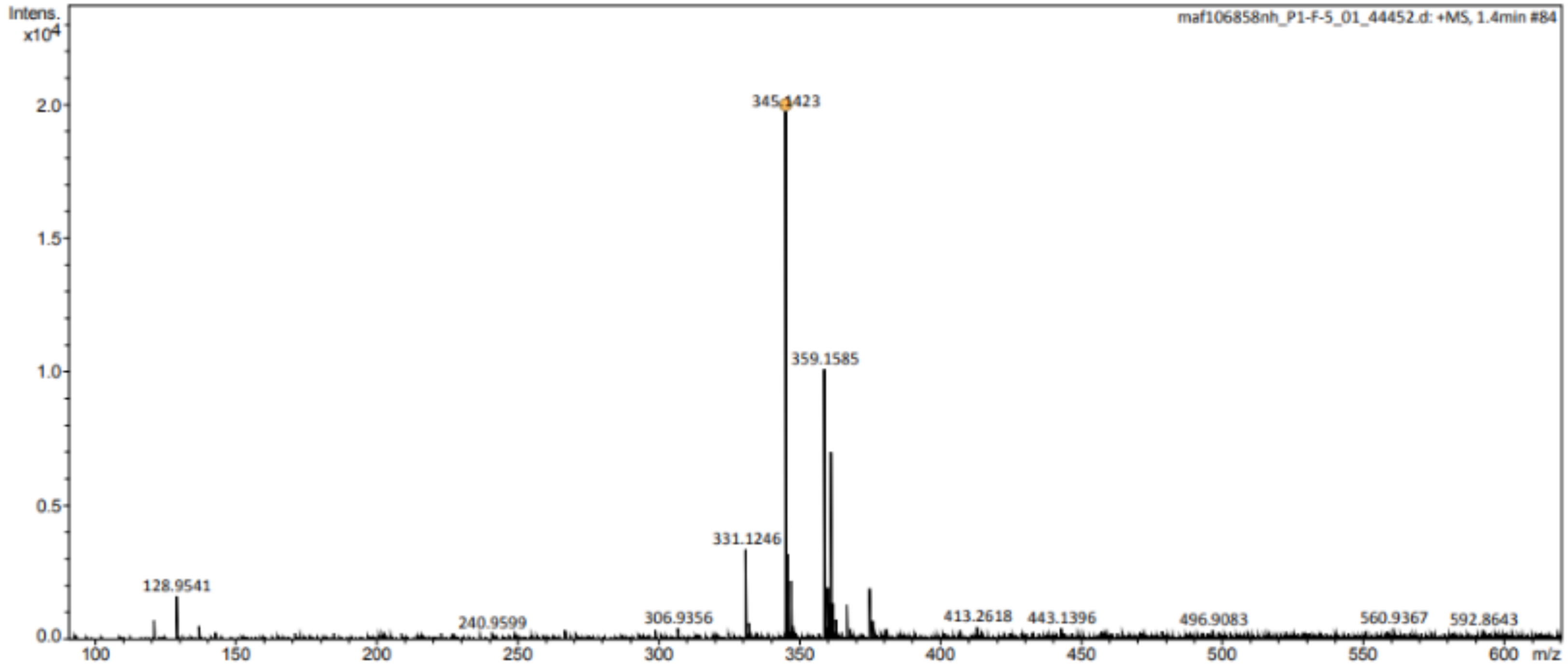

Meas. m/z	#	Ion Formula	m/z	err [ppm]	err [mDa]	mSigma	Mean err [ppm]
345.1423	1	C16H22N2NaO5	345.1421	-0.6	-0.2	13.9	1.8

*FT-IR (ATR) spectrum of benzyl 2-acetamido-2,4,6-trideoxy-4-formamido-α-L-altropyranoside (**13**)*

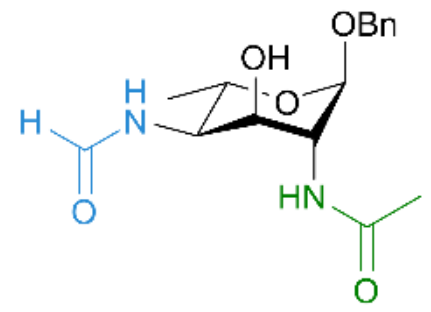

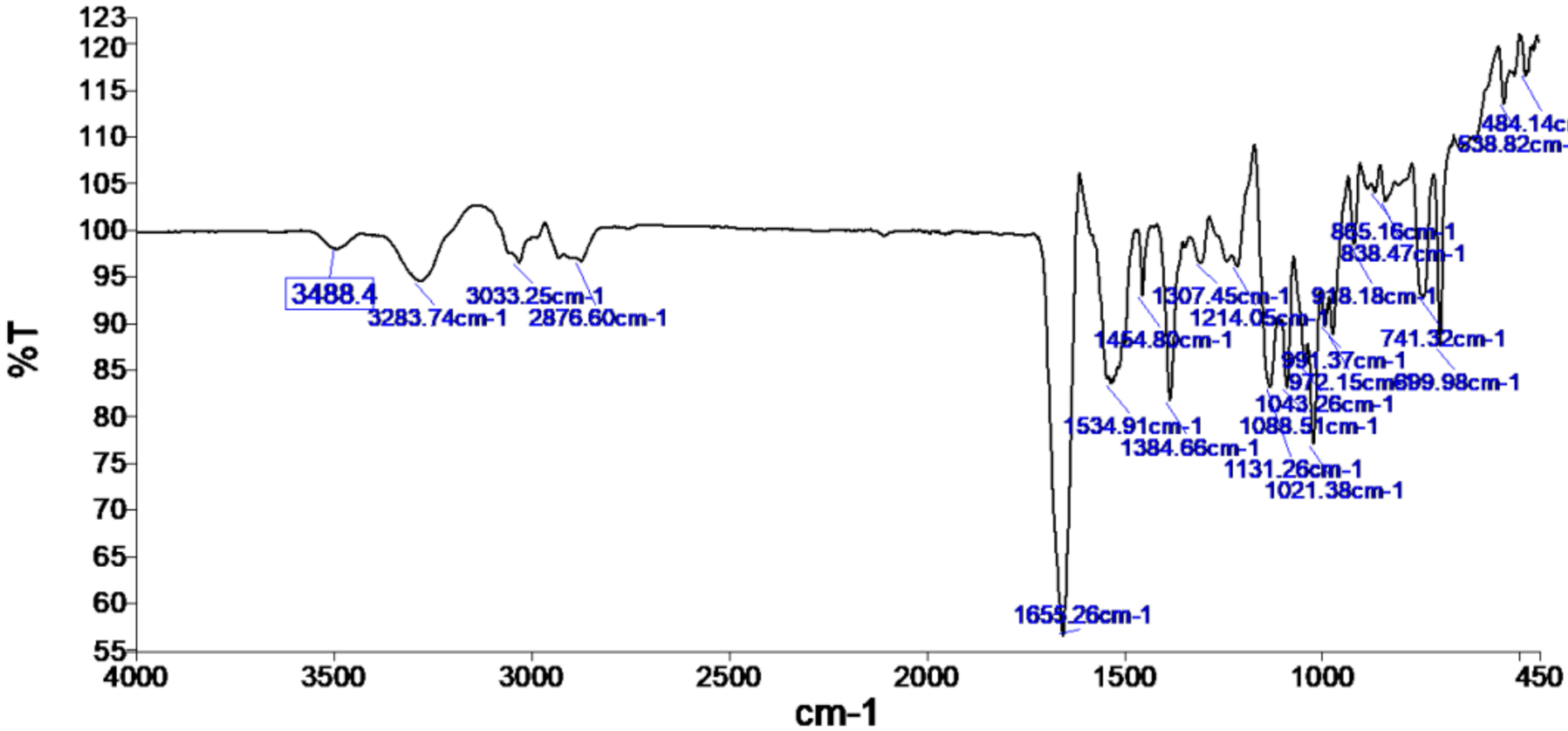

Benzyl 2,4-diacetamido-2,4,6-trideoxy-α-L-altropyranoside (**14**)

*Hydrogen NMR spectrum of benzyl 2,4-diacetamido-2,4,6-trideoxy-α-L-altropyranoside (**14**)*

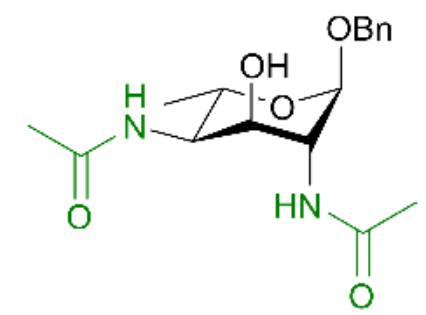

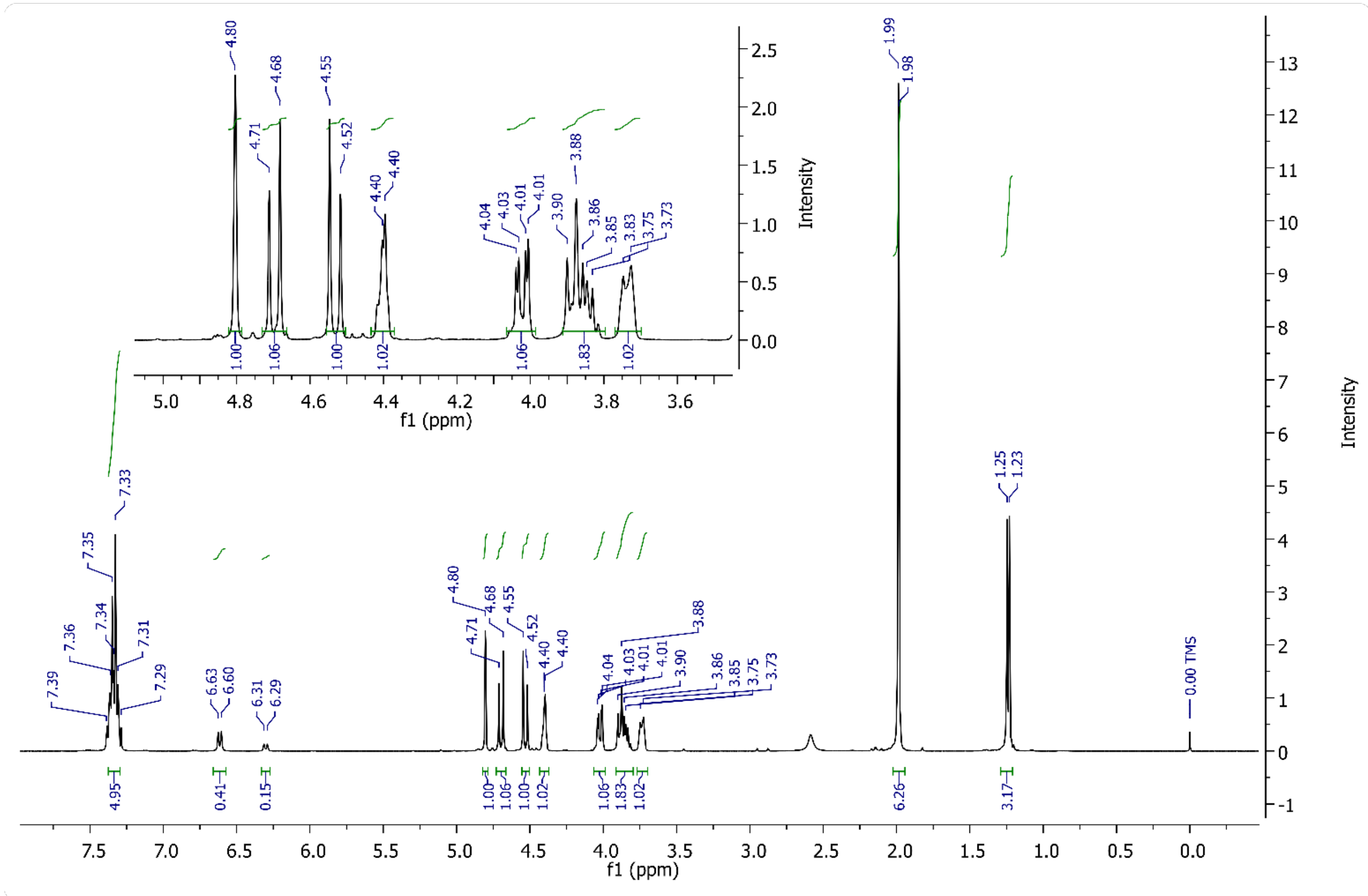

*Carbon NMR spectrum of benzyl 2,4-diacetamido-2,4,6-trideoxy-α-L-altropyranoside (**14**)*

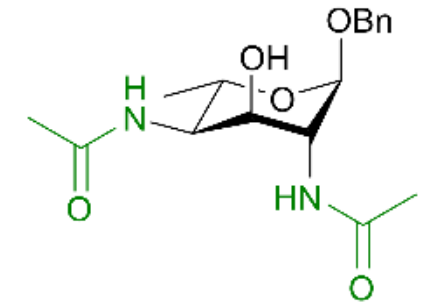

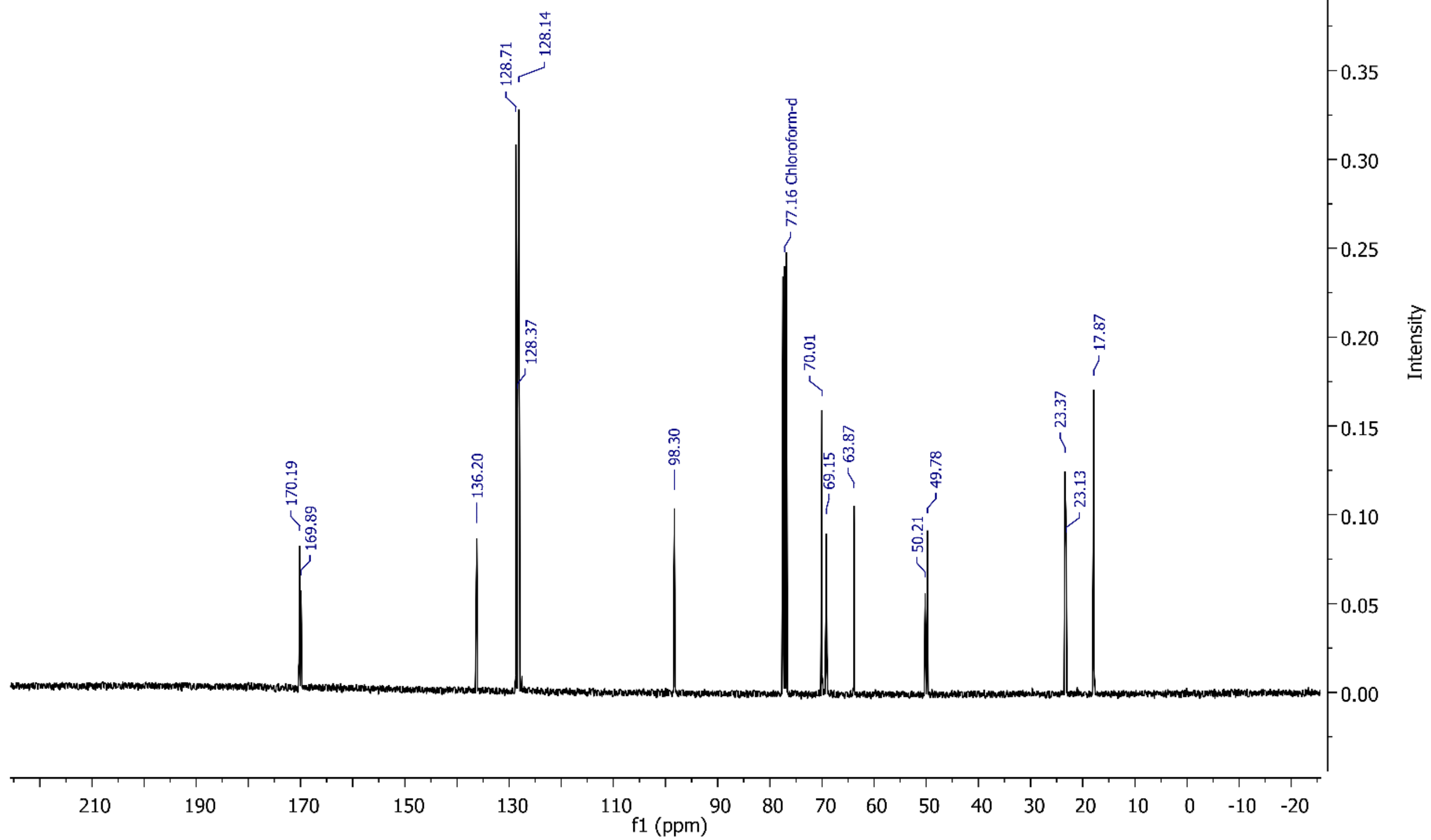

*Mass spectrum of benzyl 2,4-diacetamido-2,4,6-trideoxy-α-L-altropyranoside (**14**)*

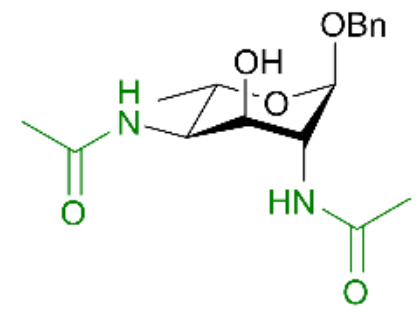

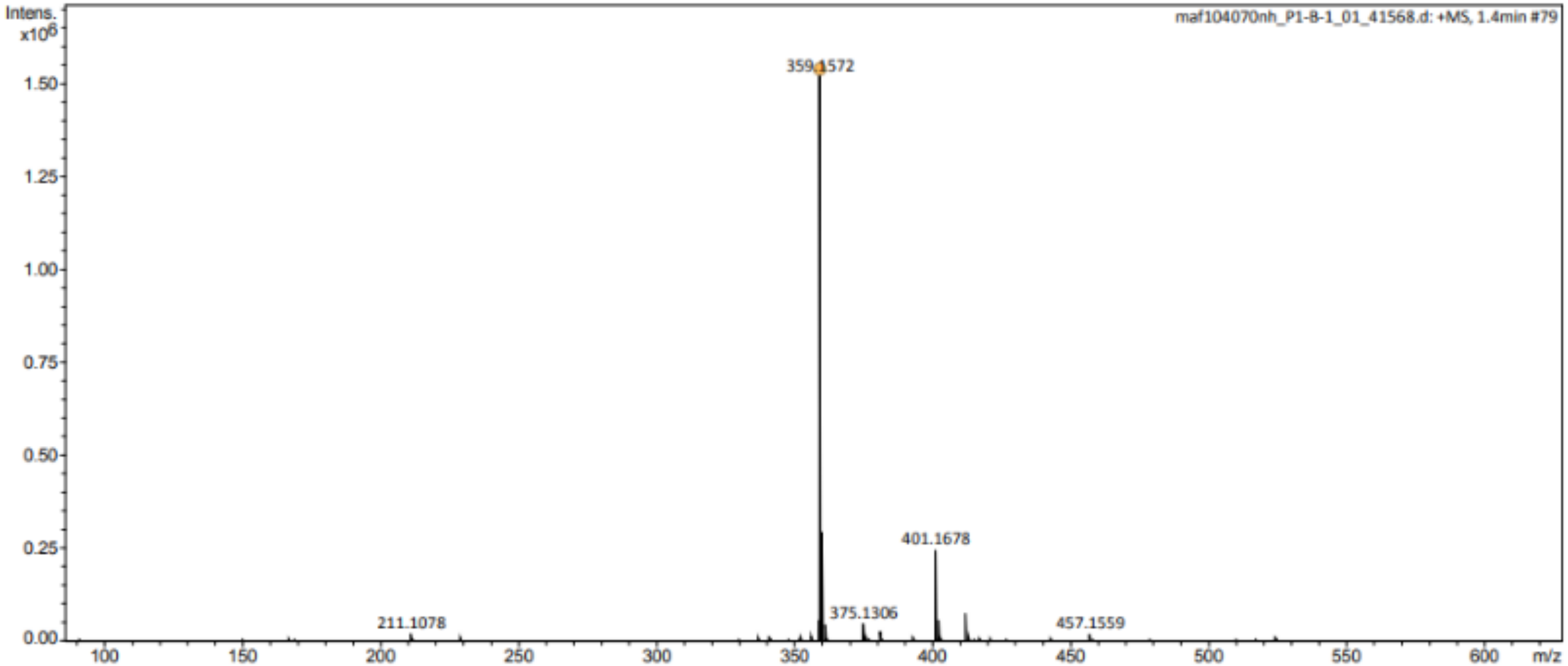

Meas. m/z	#	Ion Formula	m/z	err [ppm]	err [mDa]	mSigma	Mean err [ppm]
359.1572	1	C17H24N2NaO5	359.1577	1.6	0.6	1.5	1.5

*IR spectrum of benzyl 2,4-diacetamido-2,4,6-trideoxy-α-L-altropyranoside (**14**)*

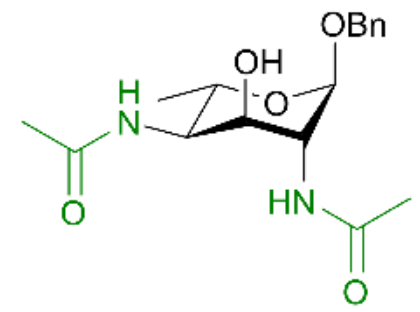

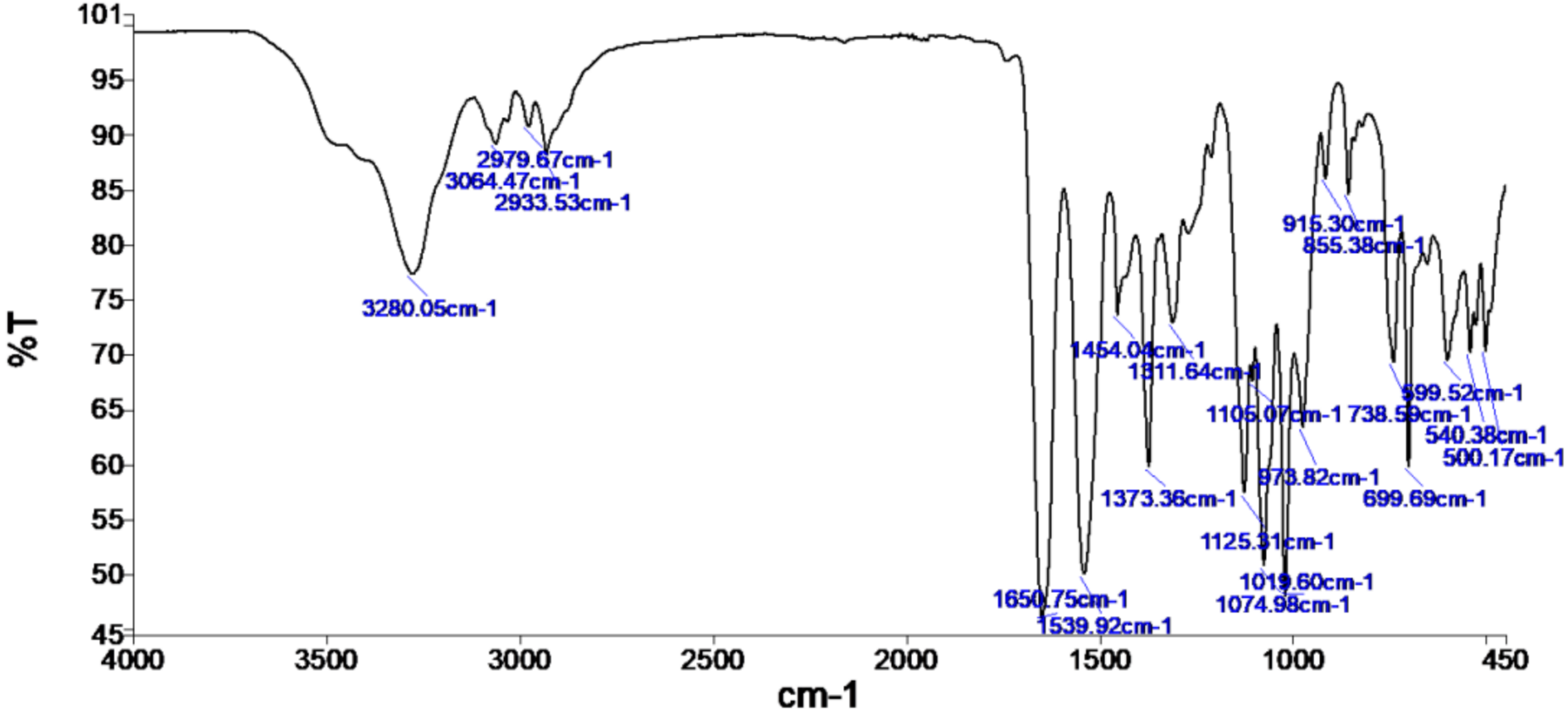

Benzyl 2-acetamido-4-[(*R*)-3-(benzyloxy)butanamido]-2,4,6-trideoxy-α-L-altropyranoside (**15**)

*Hydrogen NMR spectrum of benzyl 2-acetamido-4-[(R)-3-(benzyloxy)butanamido]-2,4,6-trideoxy-α-L-altropyranoside (**15**)*

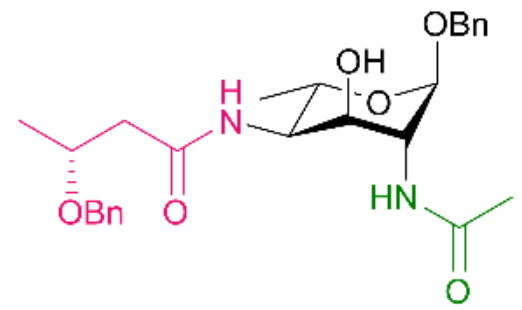

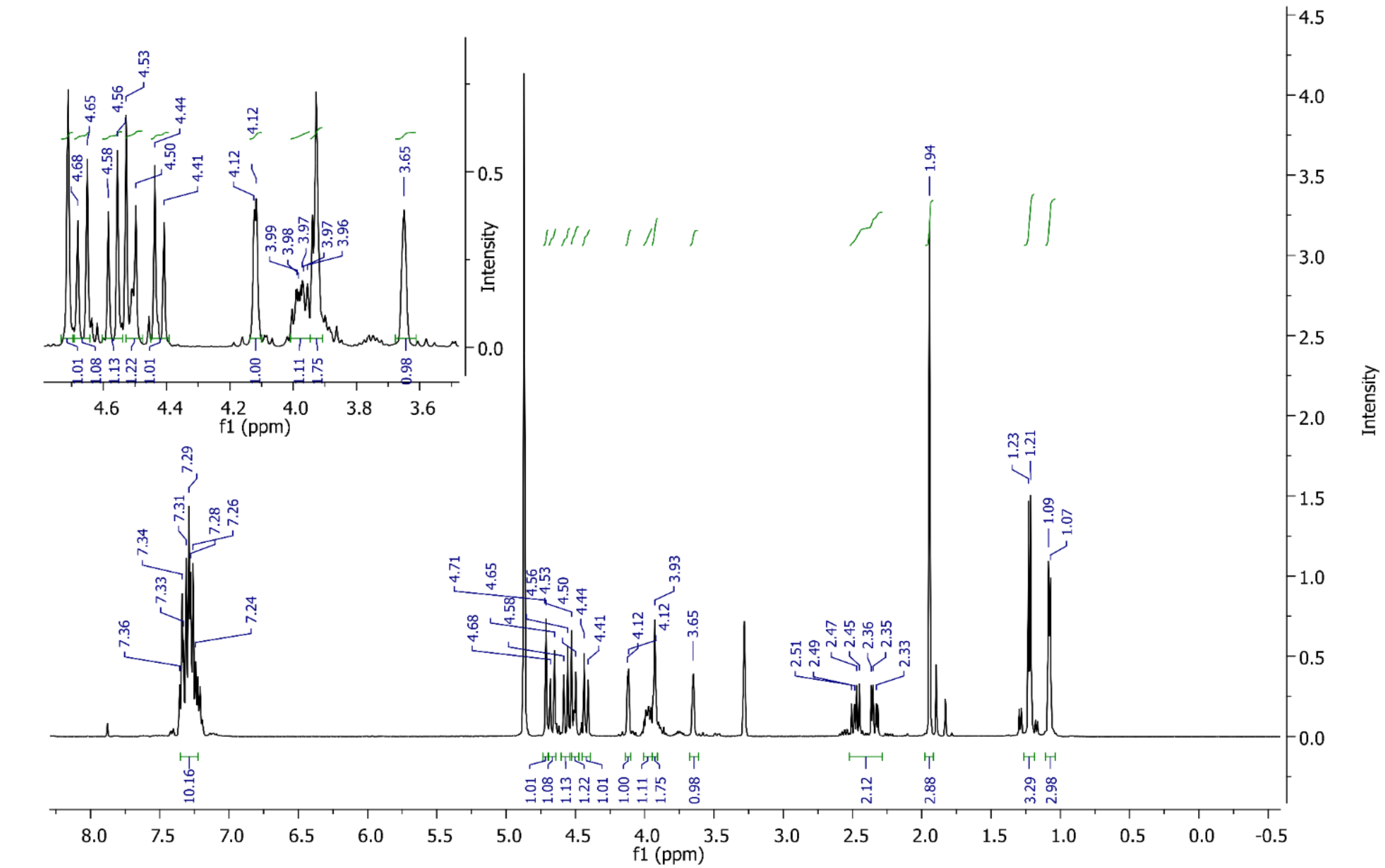

*Carbon NMR spectrum of benzyl 2-acetamido-4-[(R)-3-(benzyloxy)butanamido]-2,4,6-trideoxy-α-L-altropyranoside (**15**)*

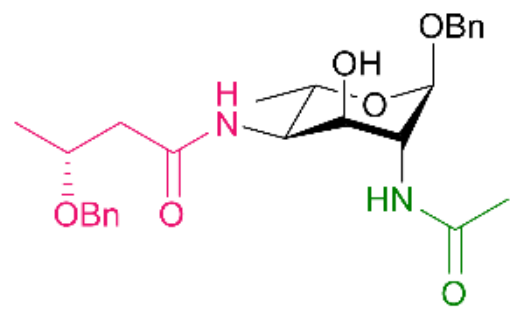

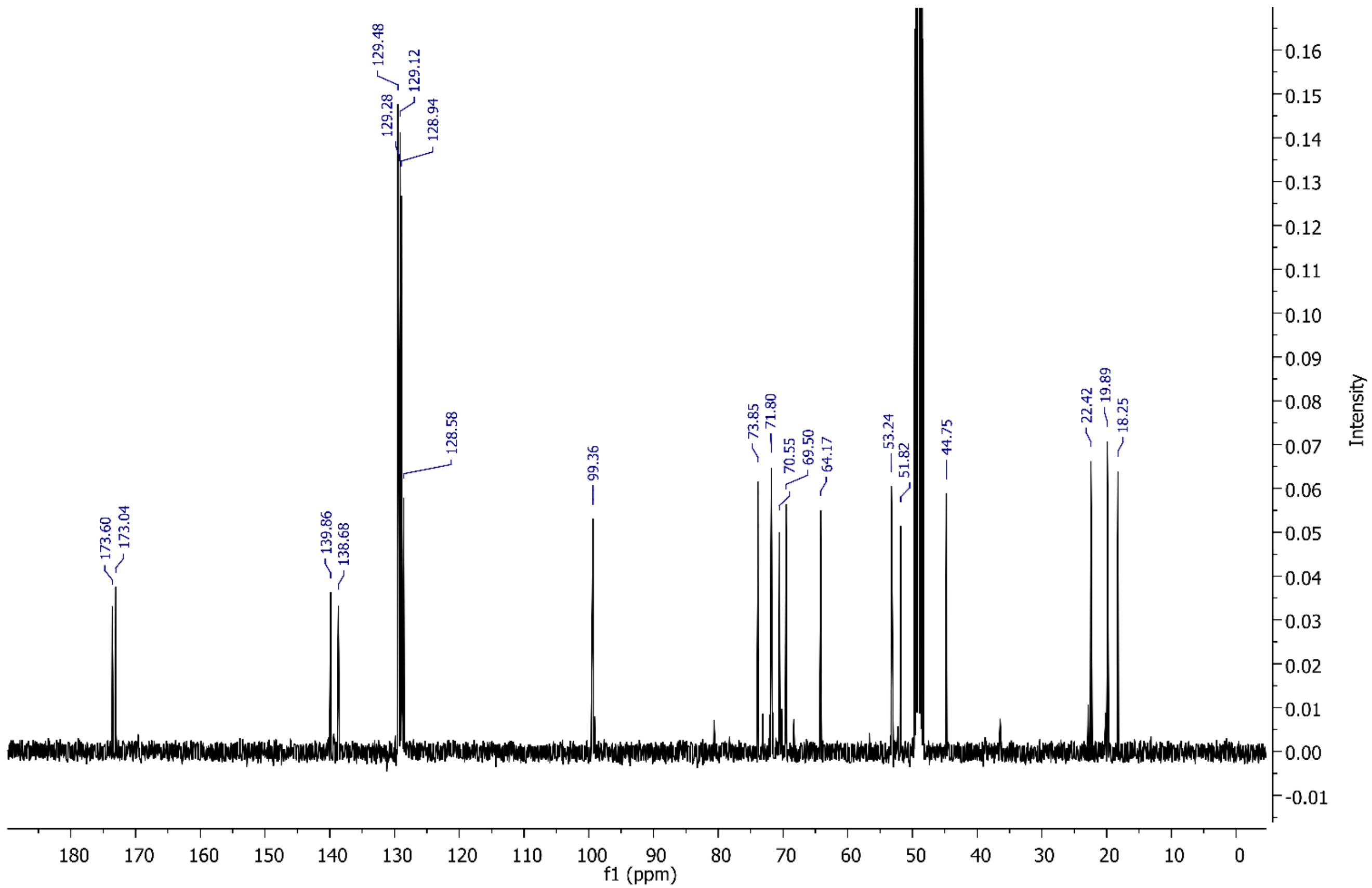

*Mass spectrum of benzyl 2-acetamido-4-[(R)-3-(benzyloxy)butanamido]-2,4,6-trideoxy-α-L-altropyranoside (**15**)*

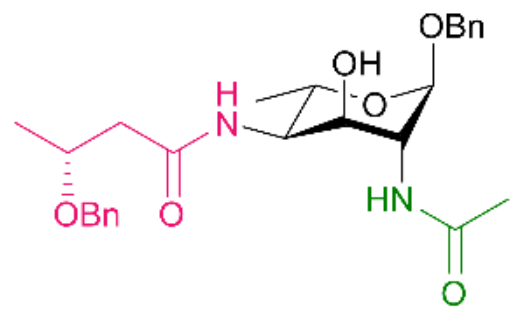

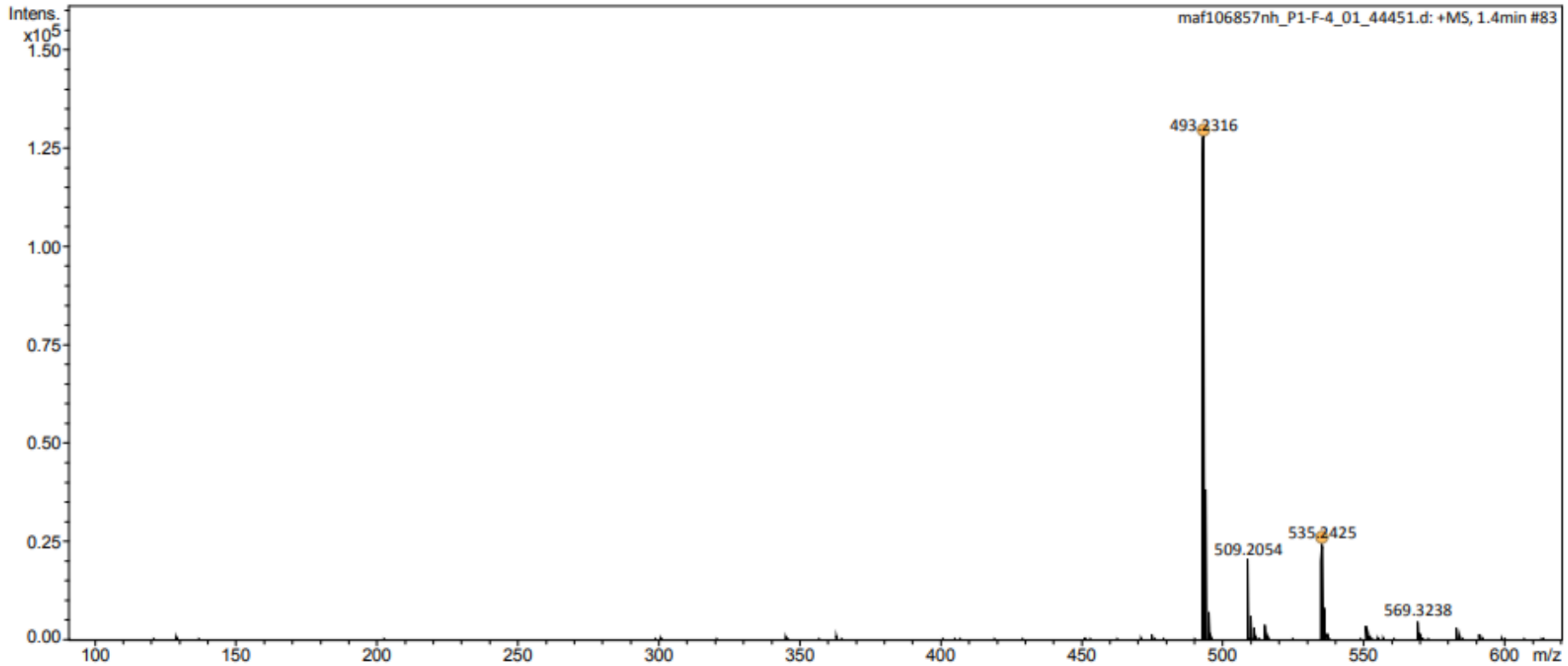

Meas. m/z	#	Ion Formula	m/z	err [ppm]	err [mDa]	mSigma	Mean err [ppm]
493.2316	1	C26H34N2NaO6	493.2309	-1.4	-0.7	3.0	-0.9
535.2425	1	C28H36N2NaO7	535.2415	-1.9	-1.0	13.3	-2.4

*FT-IR (ATR) spectrum of benzyl 2-acetamido-4-[(R)-3-(benzyloxy)butanamido]-2,4,6-trideoxy-α-L-altropyranoside (**15**)*

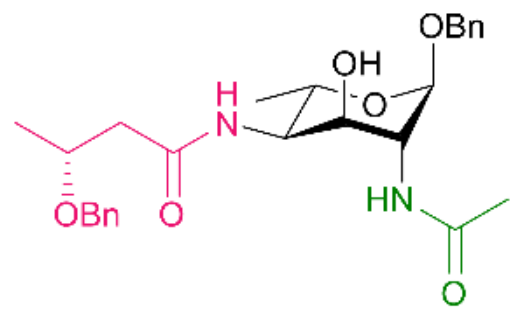

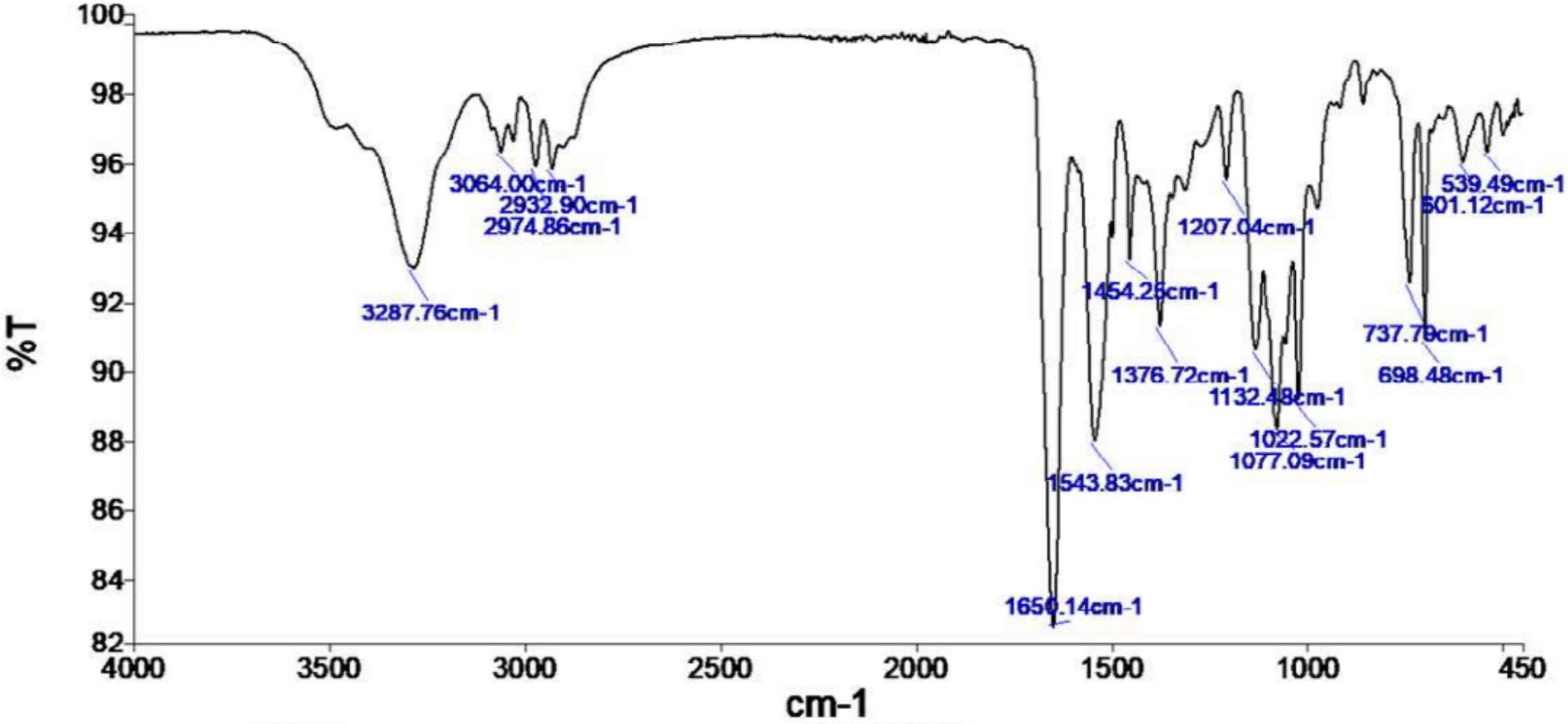

2,4,6-Trideoxy-4-formamido-2-[(*R*)-3-hydroxybutanamido]-α-L-altropyranose (**16**)

*Hydrogen NMR spectrum of 2,4,6-trideoxy-4-formamido-2-[(R)-3-hydroxybutanamido]-α-L-altropyranose (**16**)*

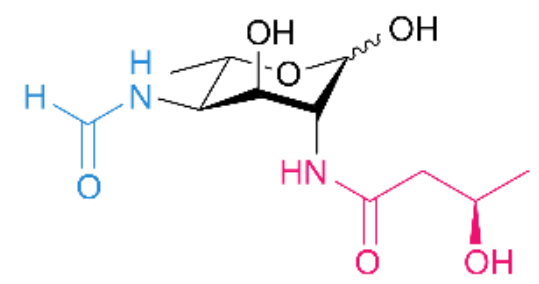

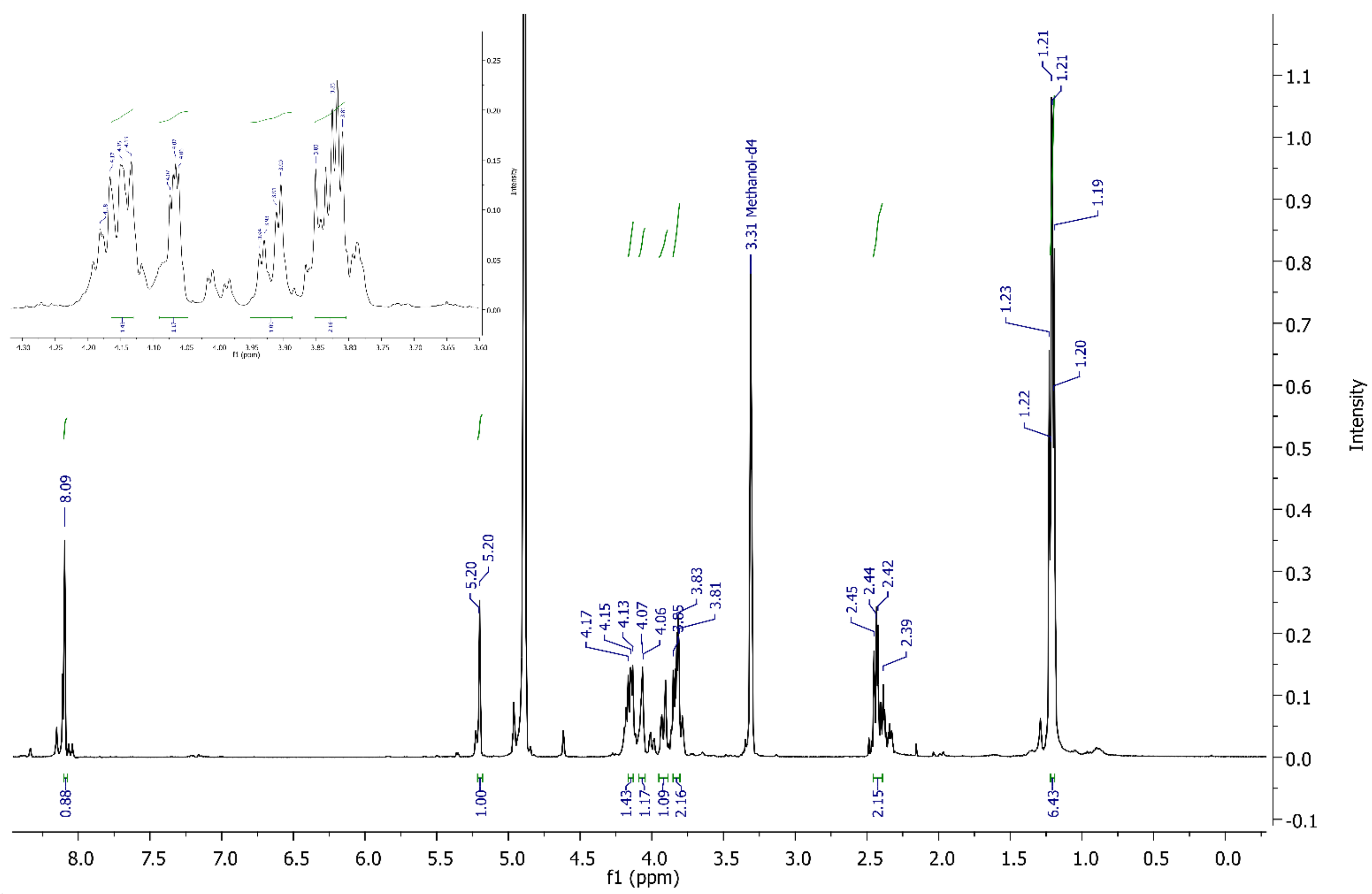

*Carbon NMR spectrum of 2,4,6-trideoxy-4-formamido-2-[(R)-3-hydroxybutanamido]-α-L-altropyranose (**16**)*

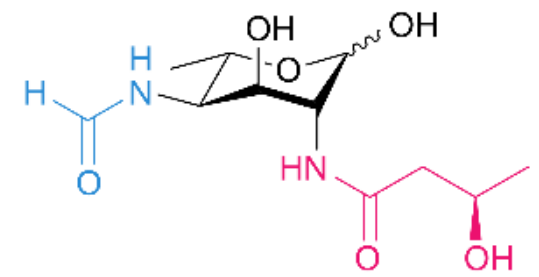

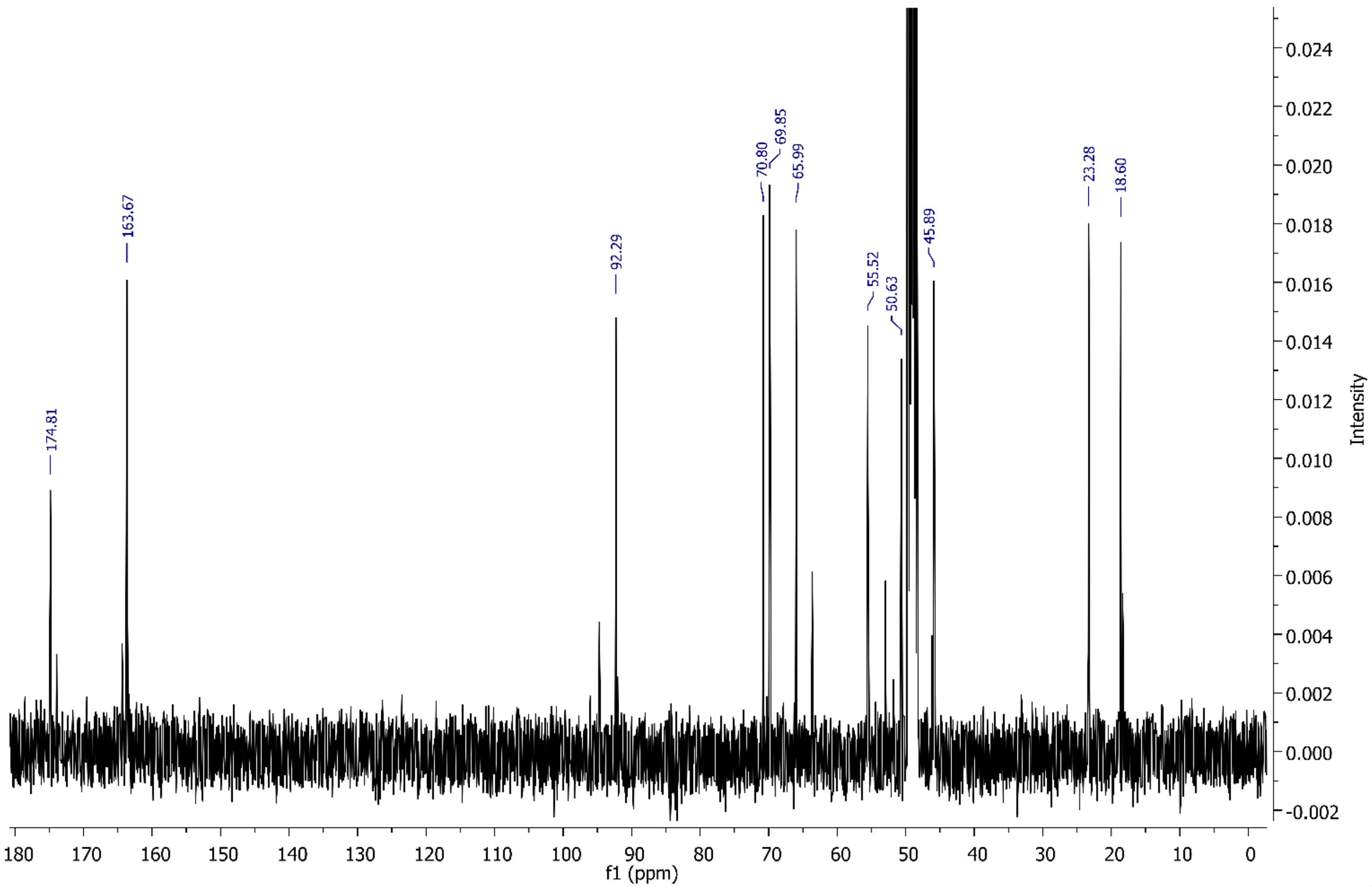

*Mass spectrum of 2,4,6-trideoxy-4-formamido-2-[(R)-3-hydroxybutanamido]-α-L-altropyranose (**16**)*

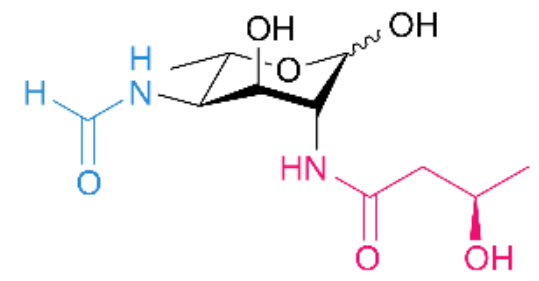

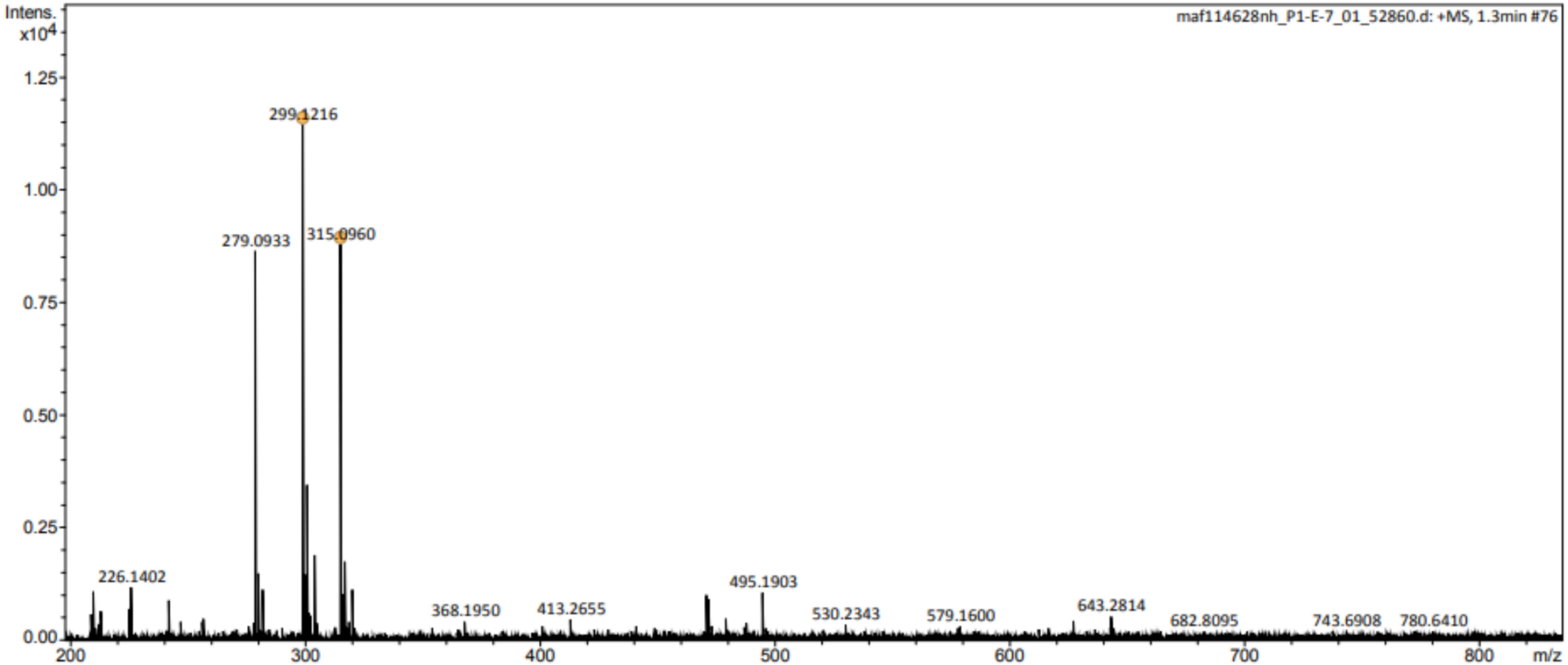

Meas. m/z	#	Ion Formula	m/z	err [ppm]	err [mDa]	mSigma	Mean err [ppm]
299.1216	1	C11H20N2NaO6	299.1214	-0.7	-0.2	11.4	-2.5
315.0960	1	C11H20KN2O6	315.0953	-2.2	-0.7	19.9	-1.9

*FT-IR (ATR) spectrum of 2,4,6-trideoxy-4-formamido-2-[(R)-3-hydroxybutanamido]-α-L-altropyranose (**16**)*

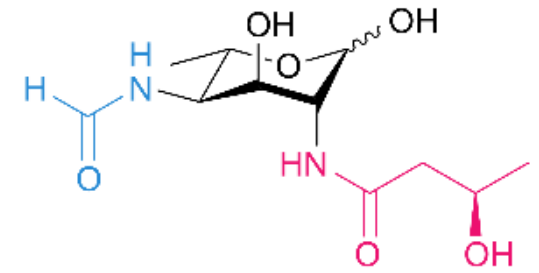

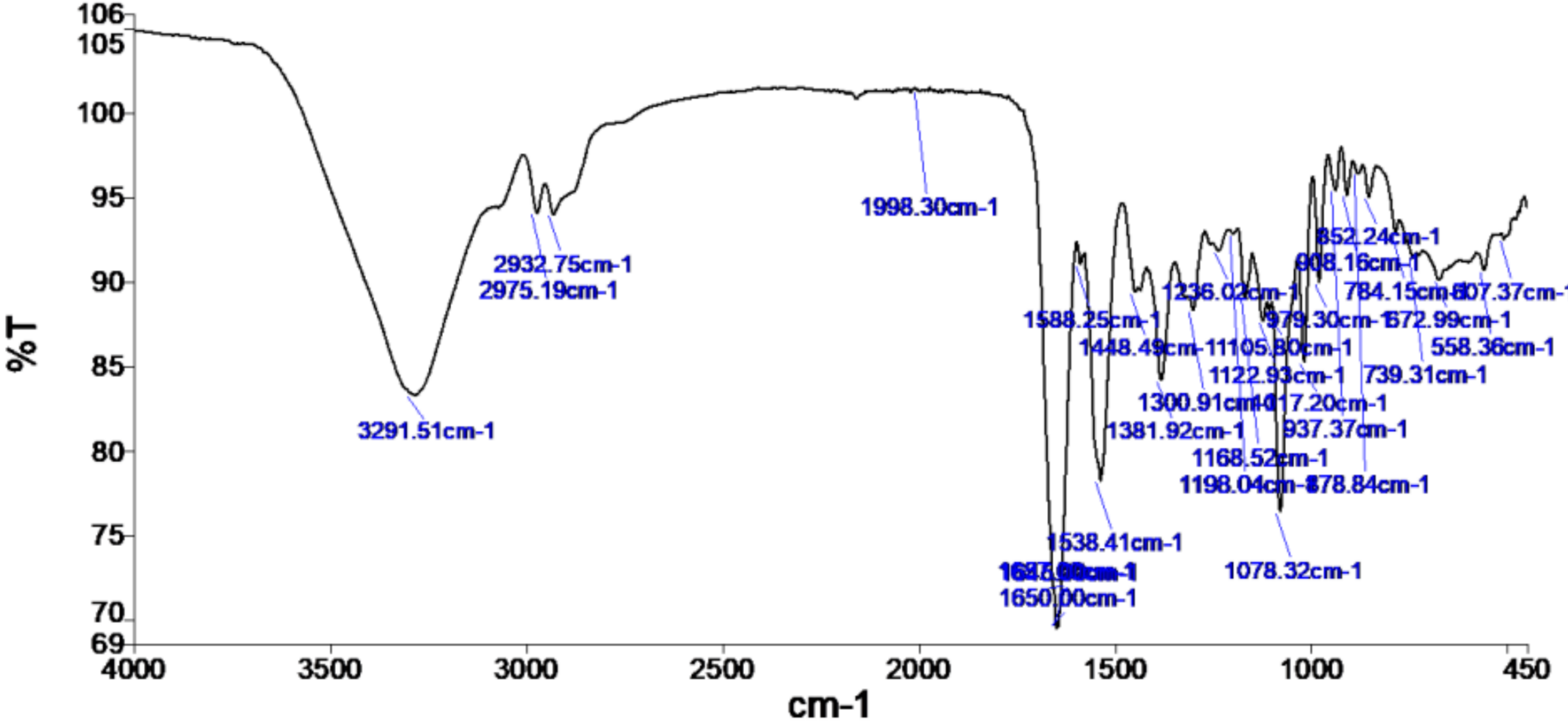

2-Acetamido-2,4,6-trideoxy-4-formamido-α-L-altropyranose (**17**)

*Hydrogen NMR spectrum of 2-acetamido-2,4,6-trideoxy-4-formamido-α-L-altropyranose (**17**)*

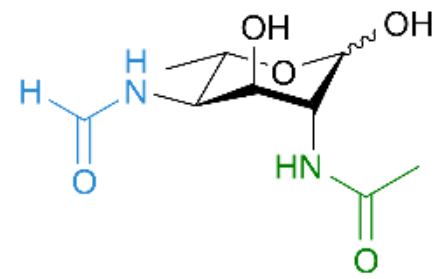

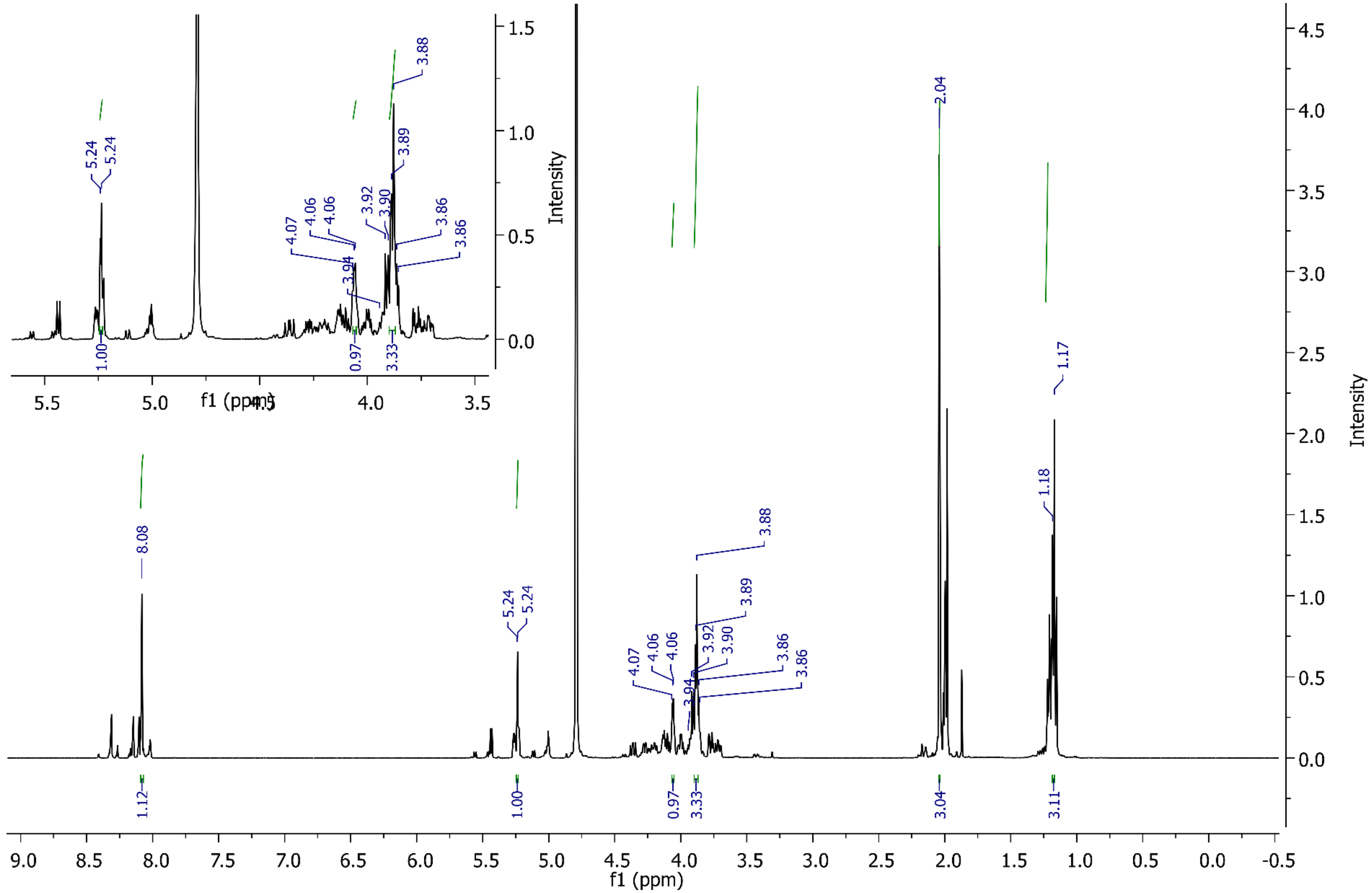

*Carbon NMR spectrum of 2-acetamido-2,4,6-trideoxy-4-formamido-α-L-altropyranose (**17**)*

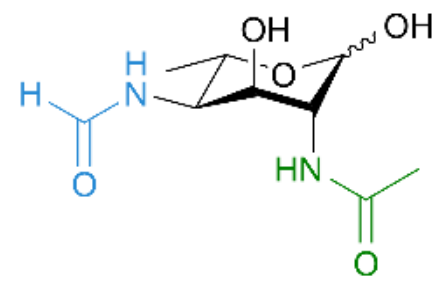

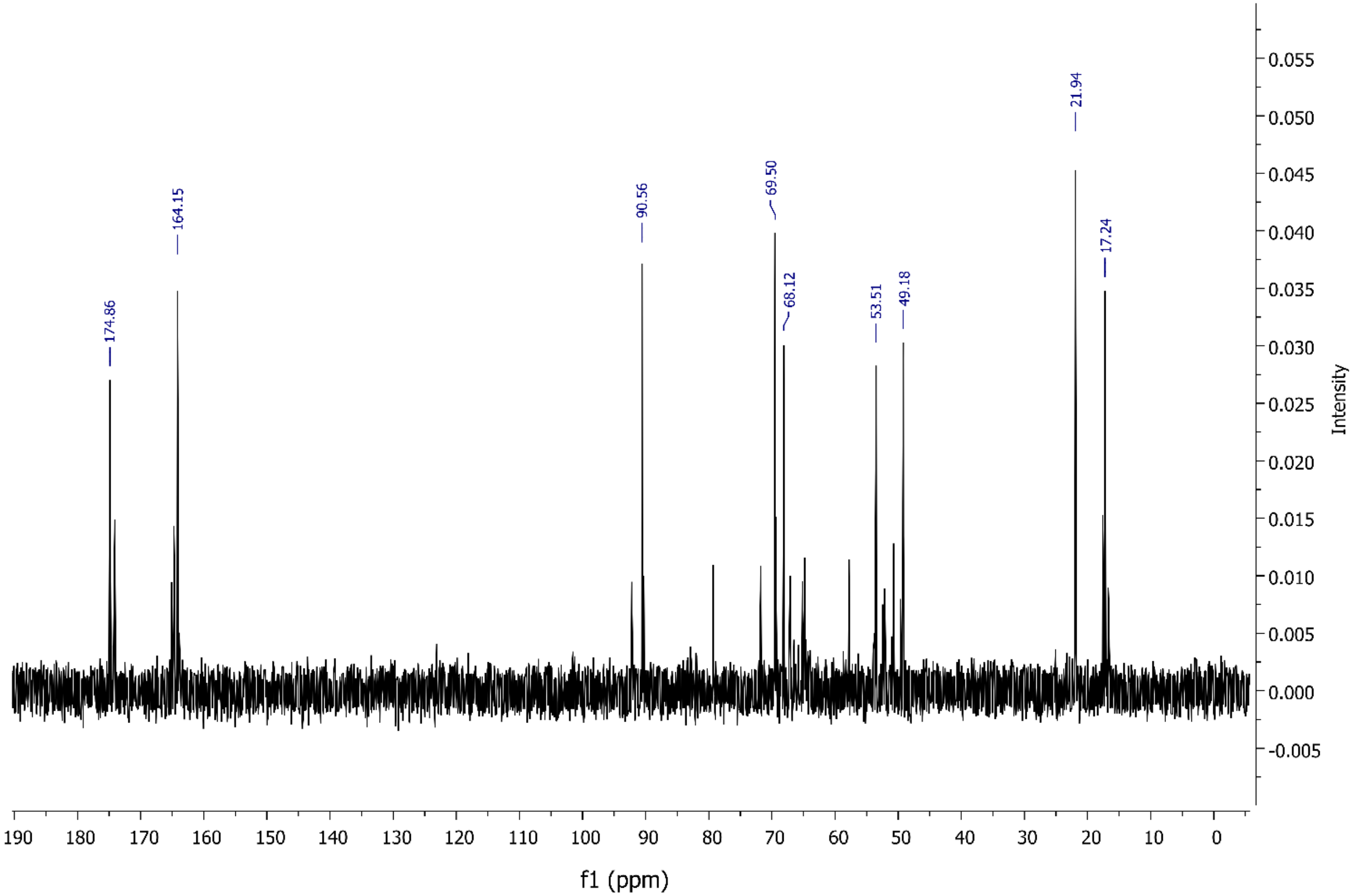

*Mass spectrum of 2-acetamido-2,4,6-trideoxy-4-formamido-α-L-altropyranose (**17**)*

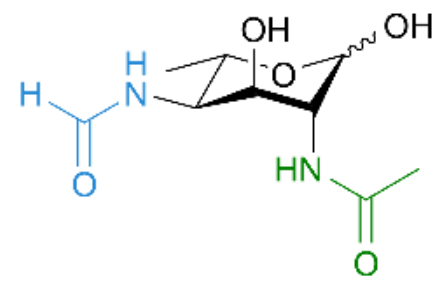

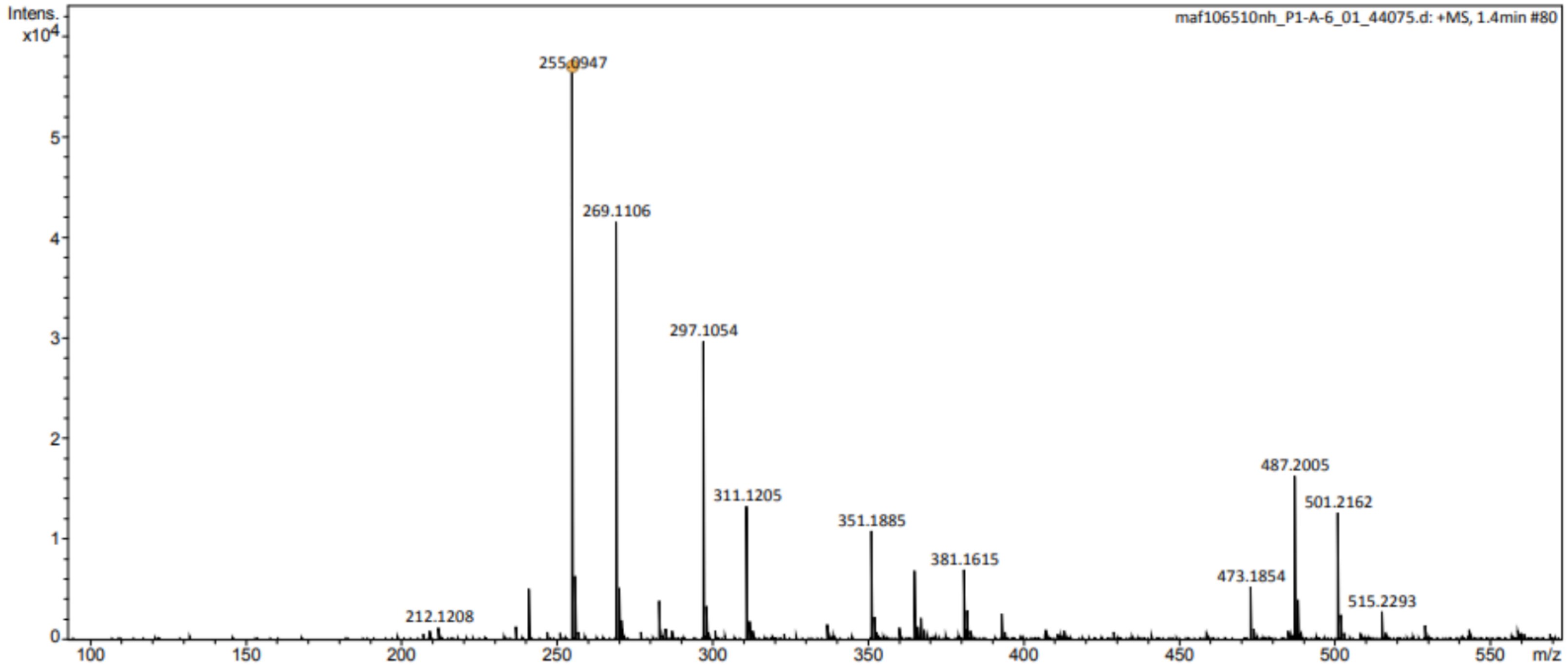

Meas. m/z	#	Ion Formula	m/z	err [ppm]	err [mDa]	mSigma	Mean err [ppm]
255.0947	1	C9H16N2NaO5	255.0951	1.6	0.4	2.9	-0.2

*FT-IR (ATR) spectrum of 2-acetamido-2,4,6-trideoxy-4-formamido-α-L-altropyranose (**17**)*

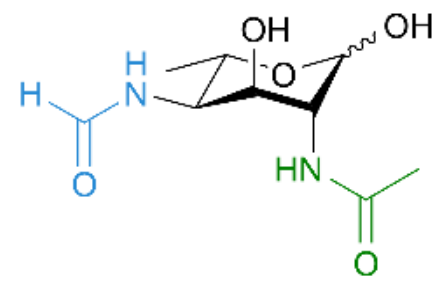

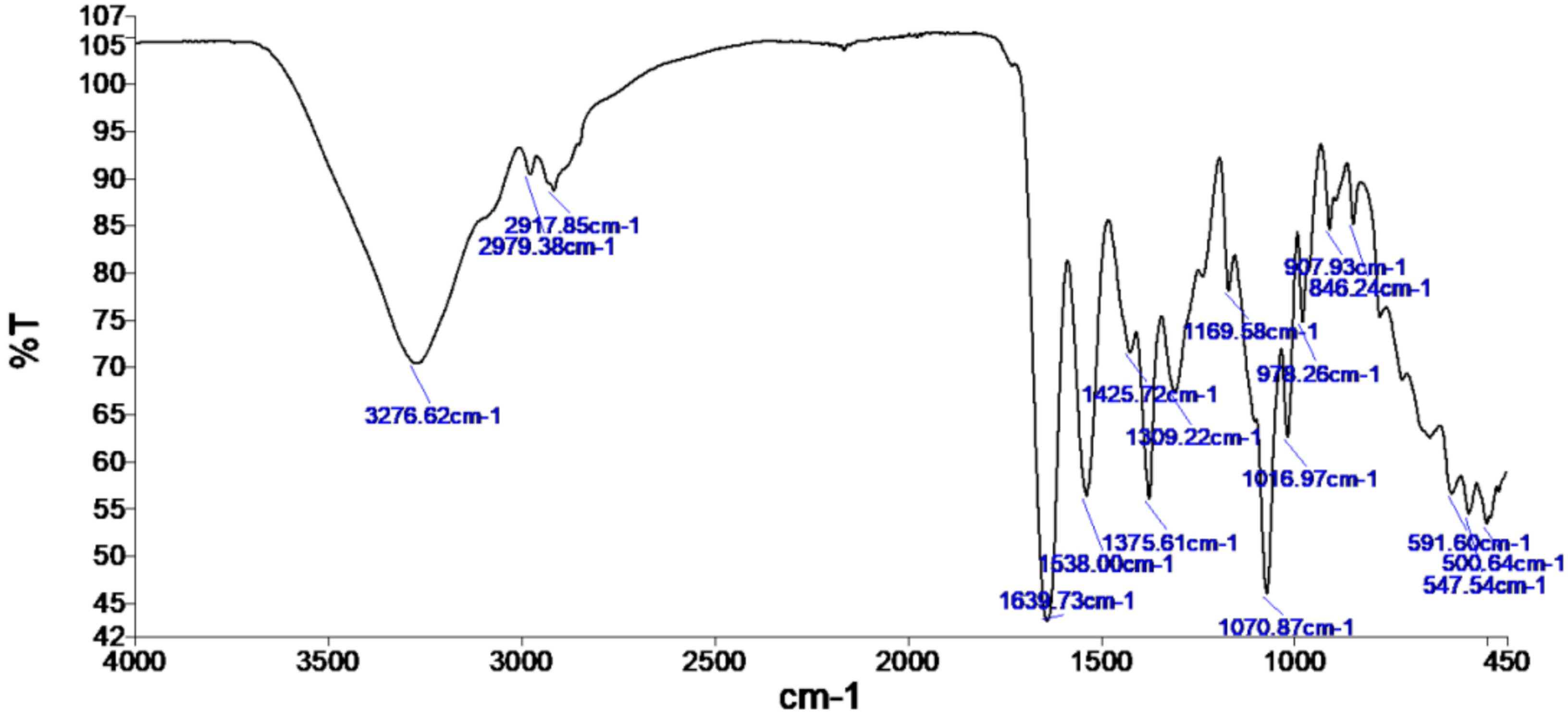

2,4-Diacetamido-2,4,6-trideoxy-α-L-altropyranose (**18**)

*Hydrogen NMR spectrum of 2,4-diacetamido-2,4,6-trideoxy-α-L-altropyranose (**18**)*

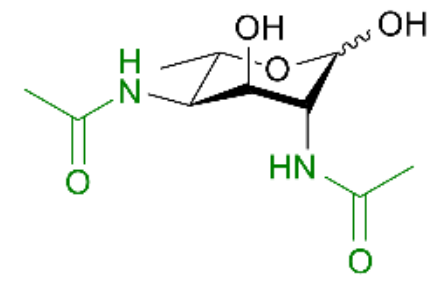

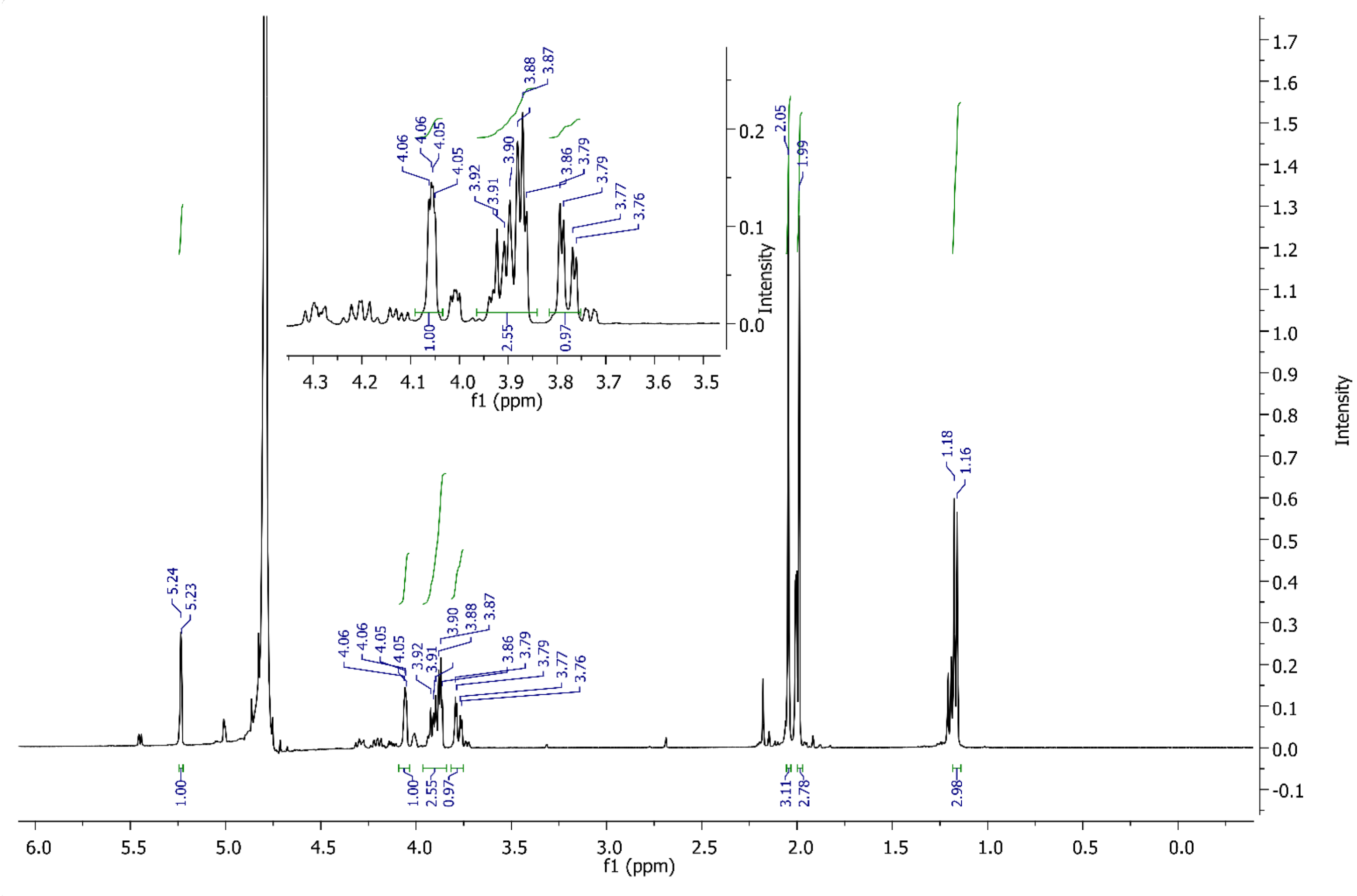

*Carbon NMR spectrum of 2,4-diacetamido-2,4,6-trideoxy-α-L-altropyranose (**18**)*

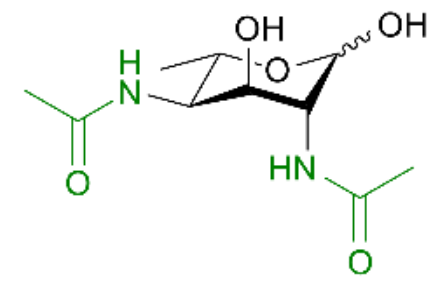

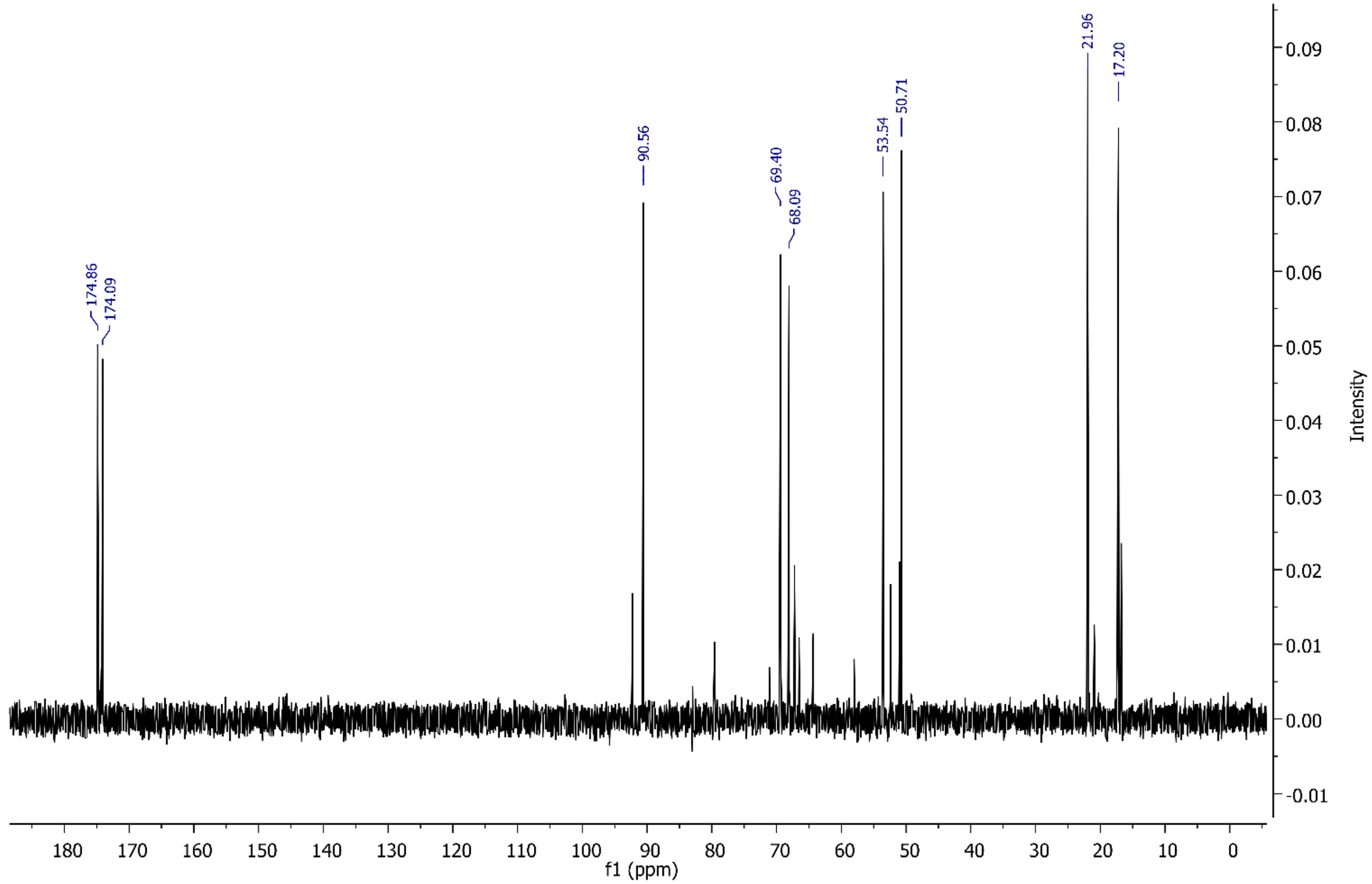

*Mass spectrum of 2,4-diacetamido-2,4,6-trideoxy-α-L-altropyranose (**18**)*

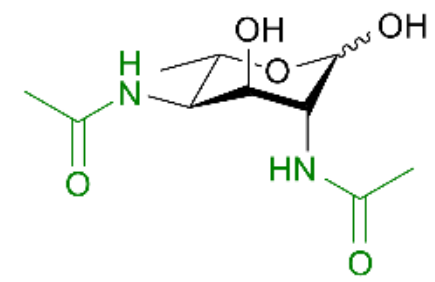

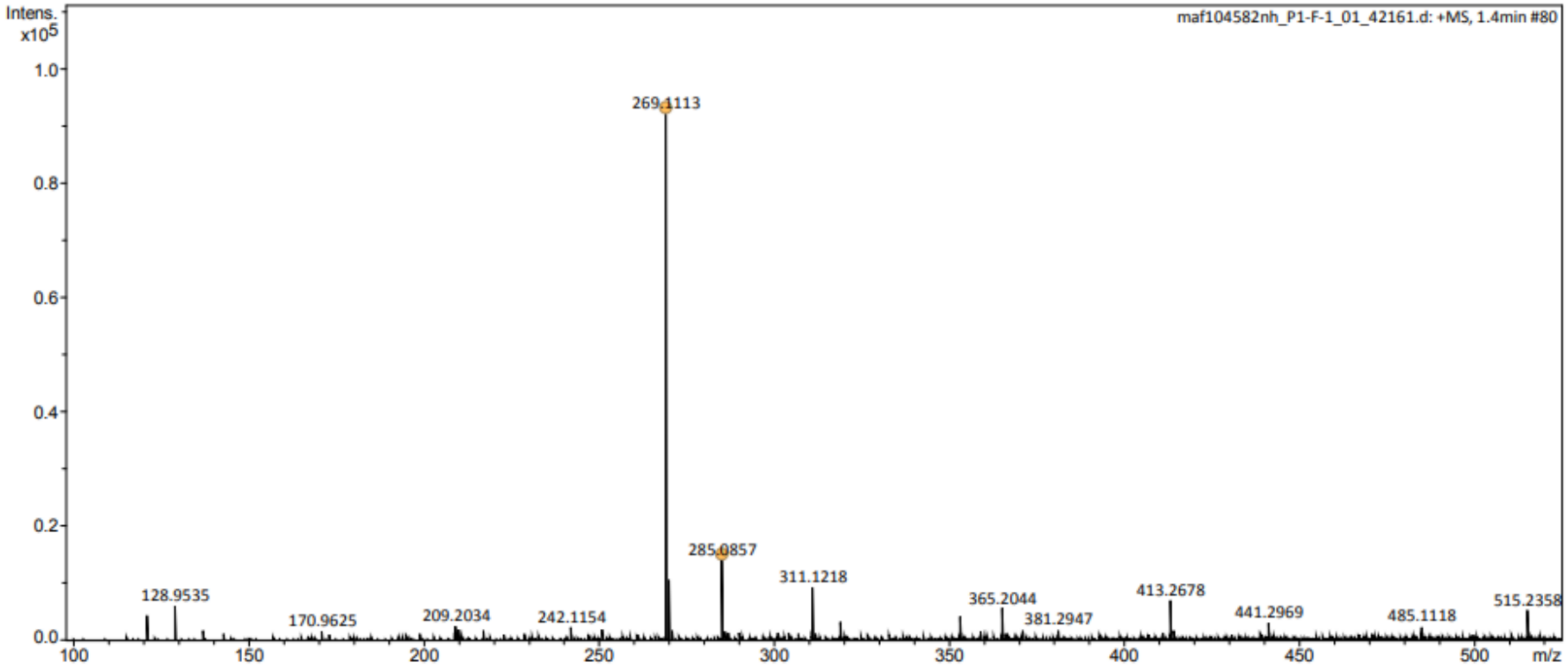

Meas. m/z	#	Ion Formula	m/z	err [ppm]	err [mDa]	mSigma	Mean err [ppm]
269.1113	1	C10H18N2NaO5	269.1108	-1.7	-0.5	2.6	-1.8
285.0857	1	C10H18KN2O5	285.0847	-3.5	-1.0	7.2	-0.8

*IR spectrum of 2,4-diacetamido-2,4,6-trideoxy-α-L-altropyranose (**18**)*

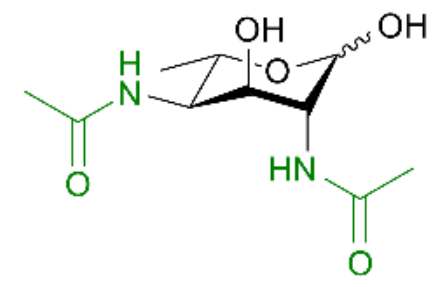

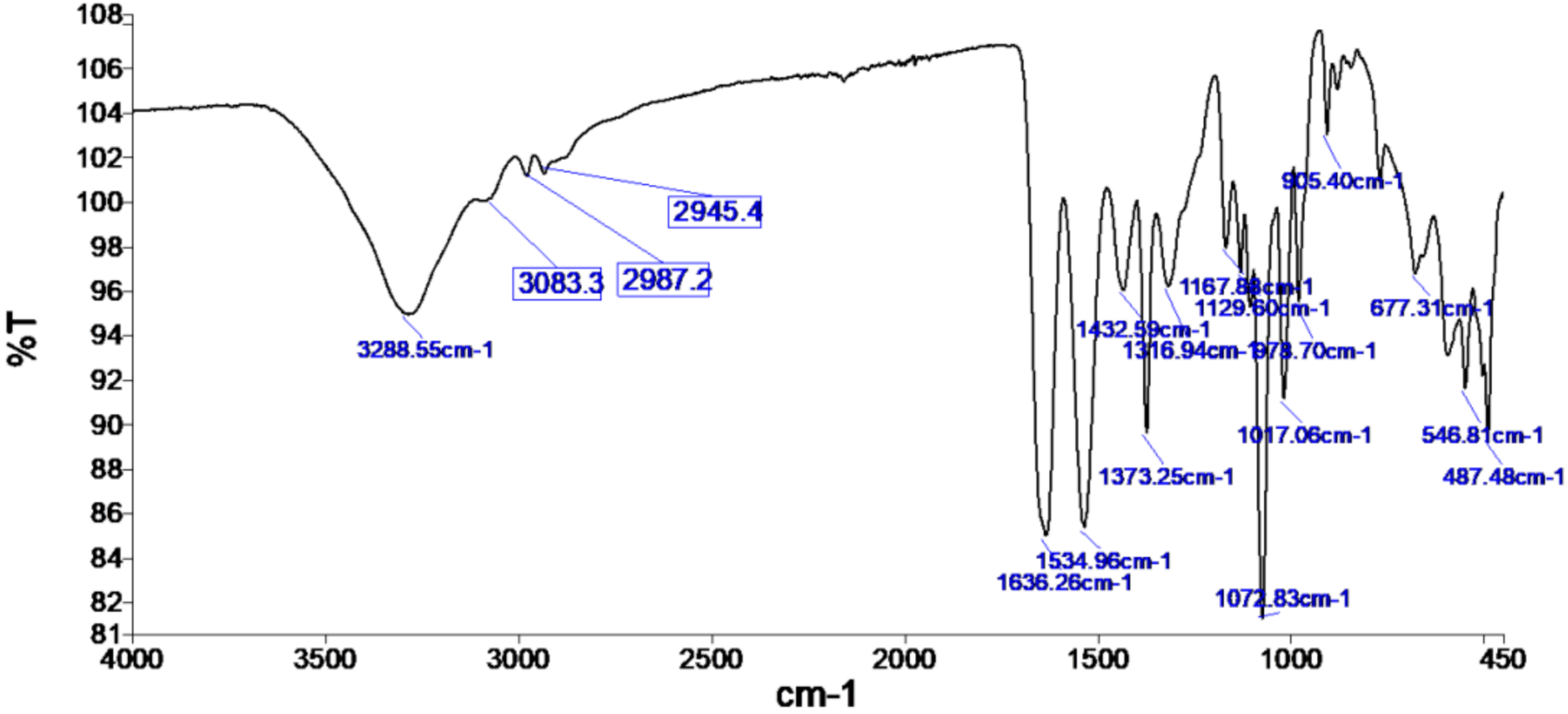

2-Acetamido-2,4,6-trideoxy-4-[(*R*)-3-hydroxybutanamido]-α-L-altropyranose (**19**)

*Hydrogen NMR spectrum of 2-acetamido-2,4,6-trideoxy-4-[(R)-3-hydroxybutanamido]-α-L-altropyranose (**19**)*

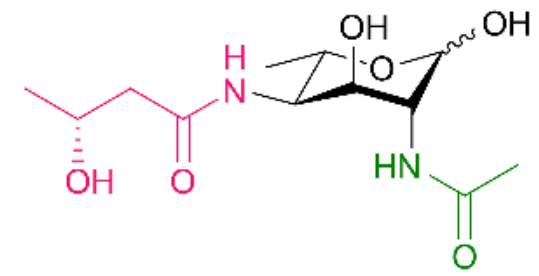

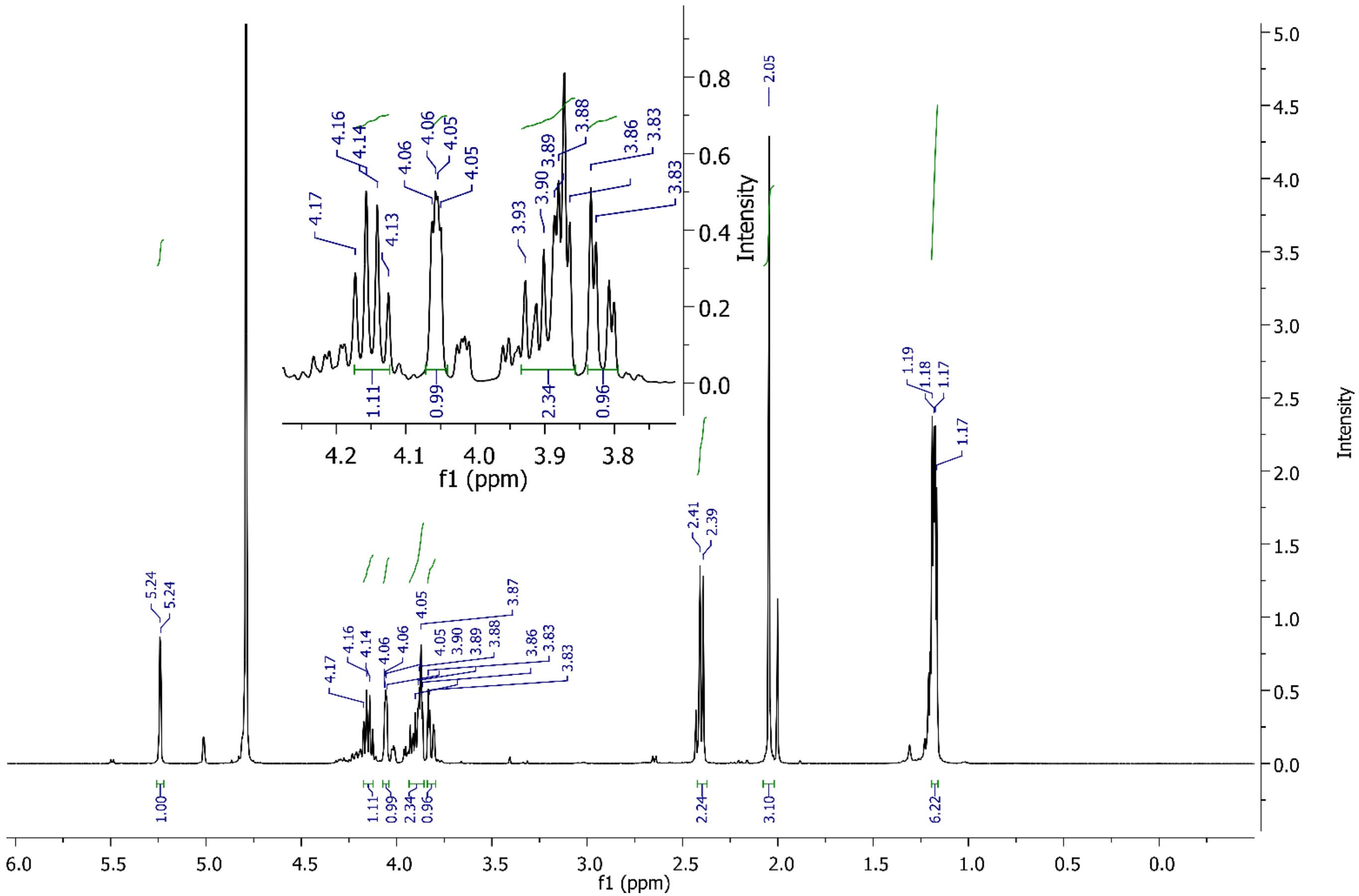

*Carbon NMR spectrum of 2-acetamido-2,4,6-trideoxy-4-[(R)-3-hydroxybutanamido]-α-L-altropyranose (**19**)*

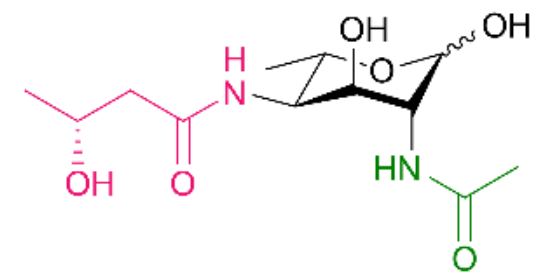

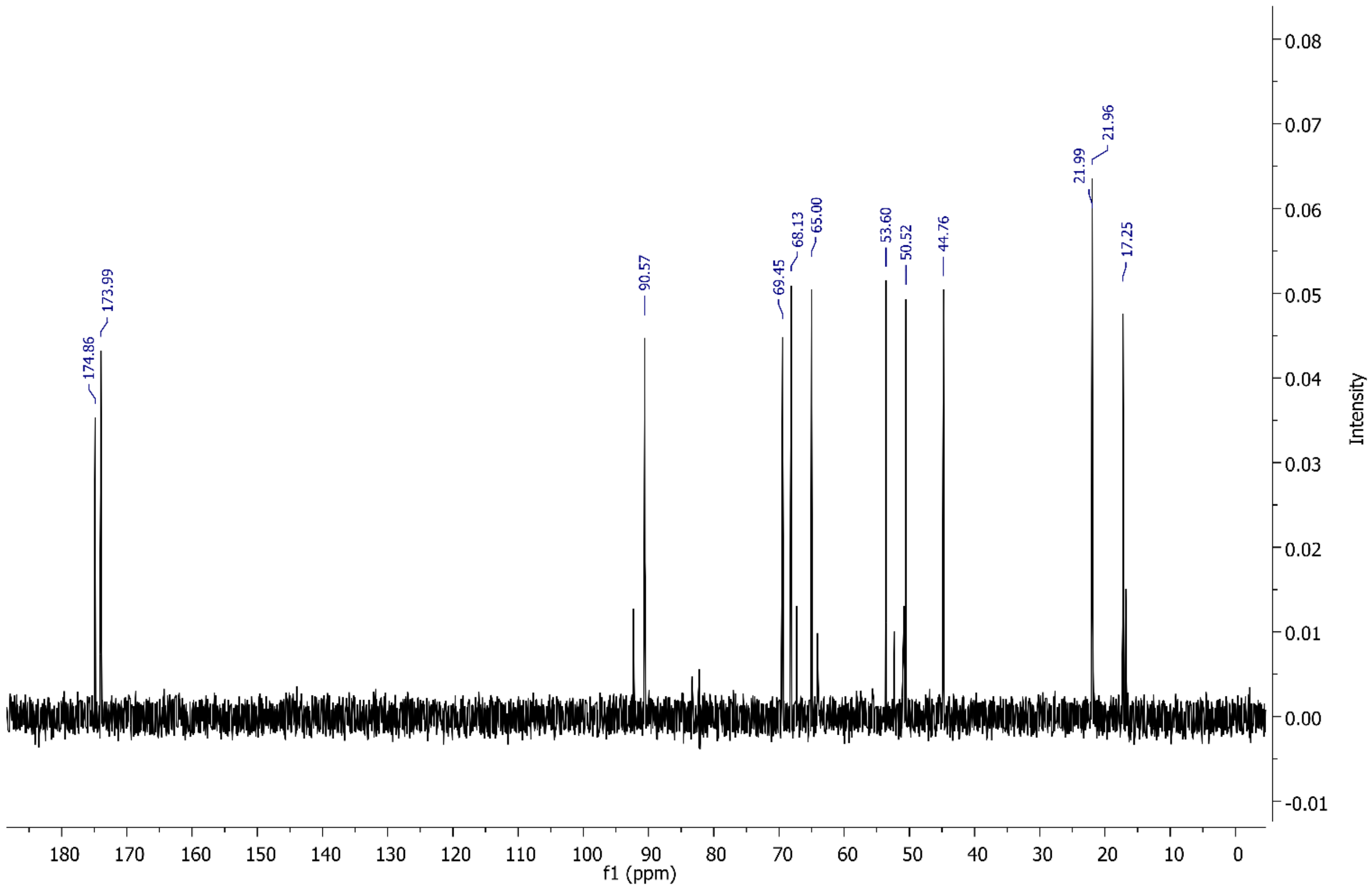

*Mass spectrum of 2-acetamido-2,4,6-trideoxy-4-[(R)-3-hydroxybutanamido]-α-L-altropyranose (**19**)*

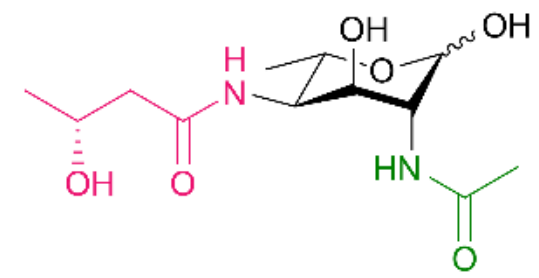

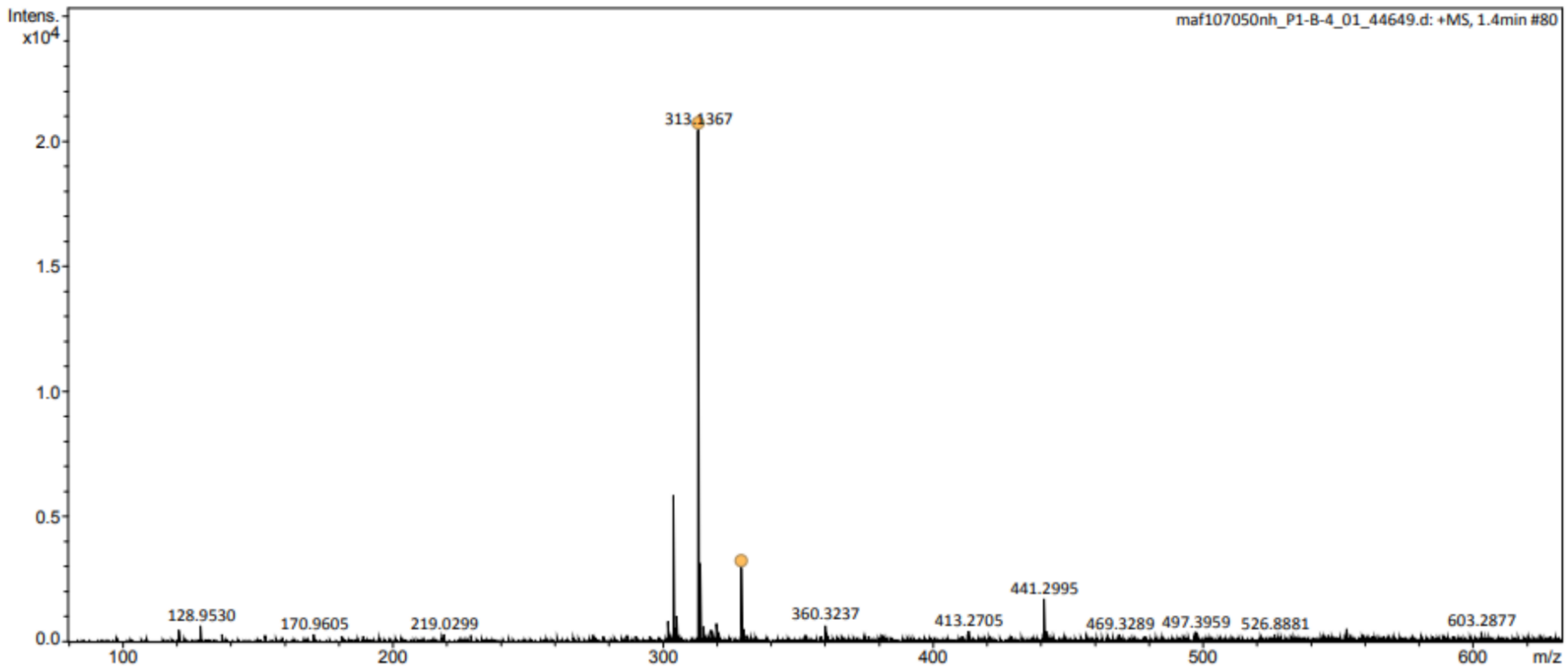

Meas. m/z	#	Ion Formula	m/z	err [ppm]	err [mDa]	mSigma	Mean err [ppm]
313.1367	1	C12H22N2NaO6	313.1370	1.1	0.4	8.2	1.9
329.1102	1	C12H22KN2O6	329.1109	2.2	0.7	21.3	2.3

*FT-IR (ATR) spectrum of 2-acetamido-2,4,6-trideoxy-4-[(R)-3-hydroxybutanamido]-α-L-altropyranose (**19**)*

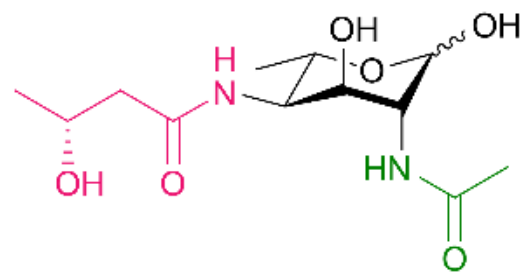

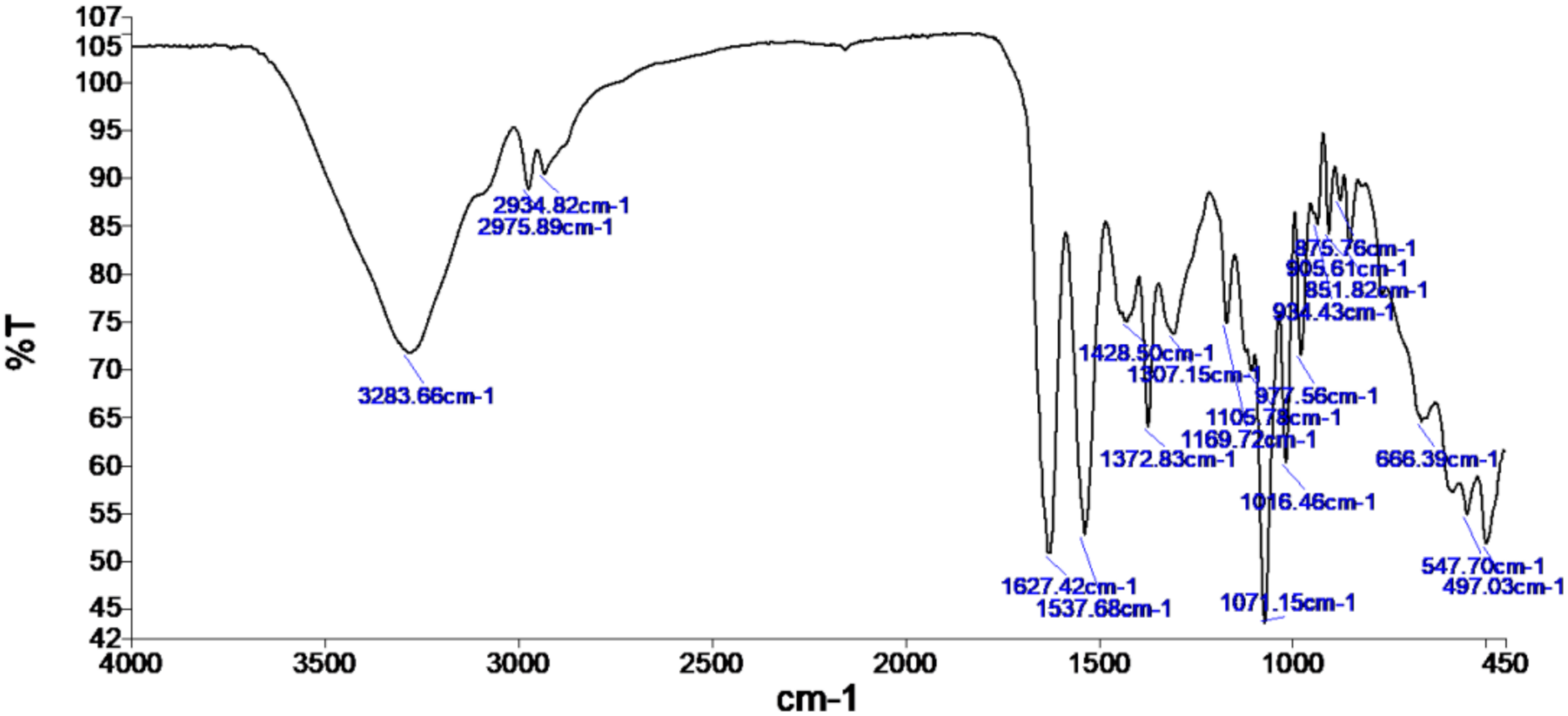

Pse5Hb7Fm (**20**)

*Hydrogen NMR spectrum of Pse5Hb7Fm (**20**) 700 MHz*

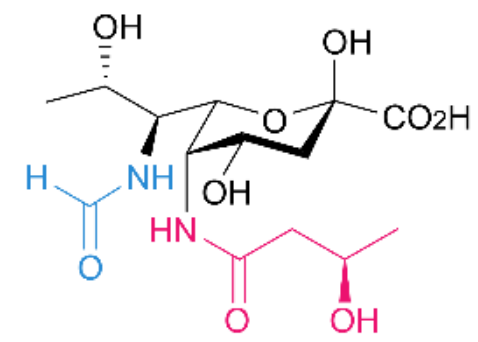

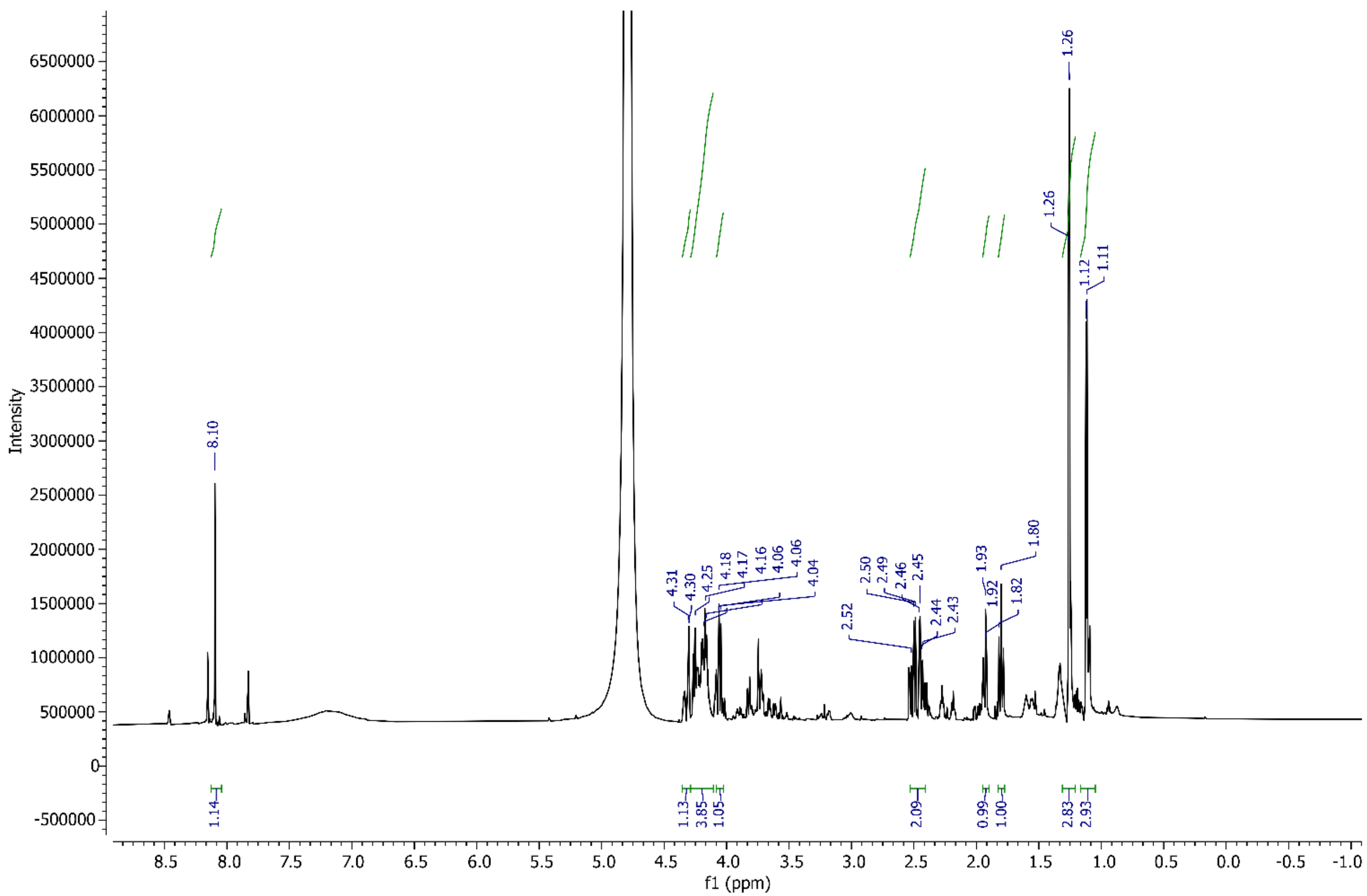

*Carbon NMR spectrum of Pse5Hb7Fm (**20**)*

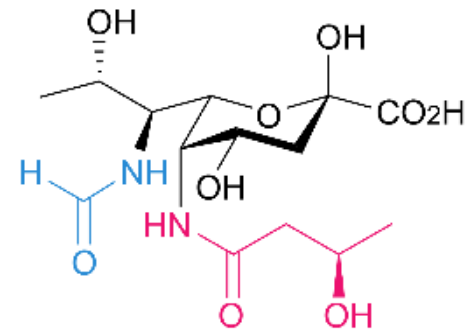

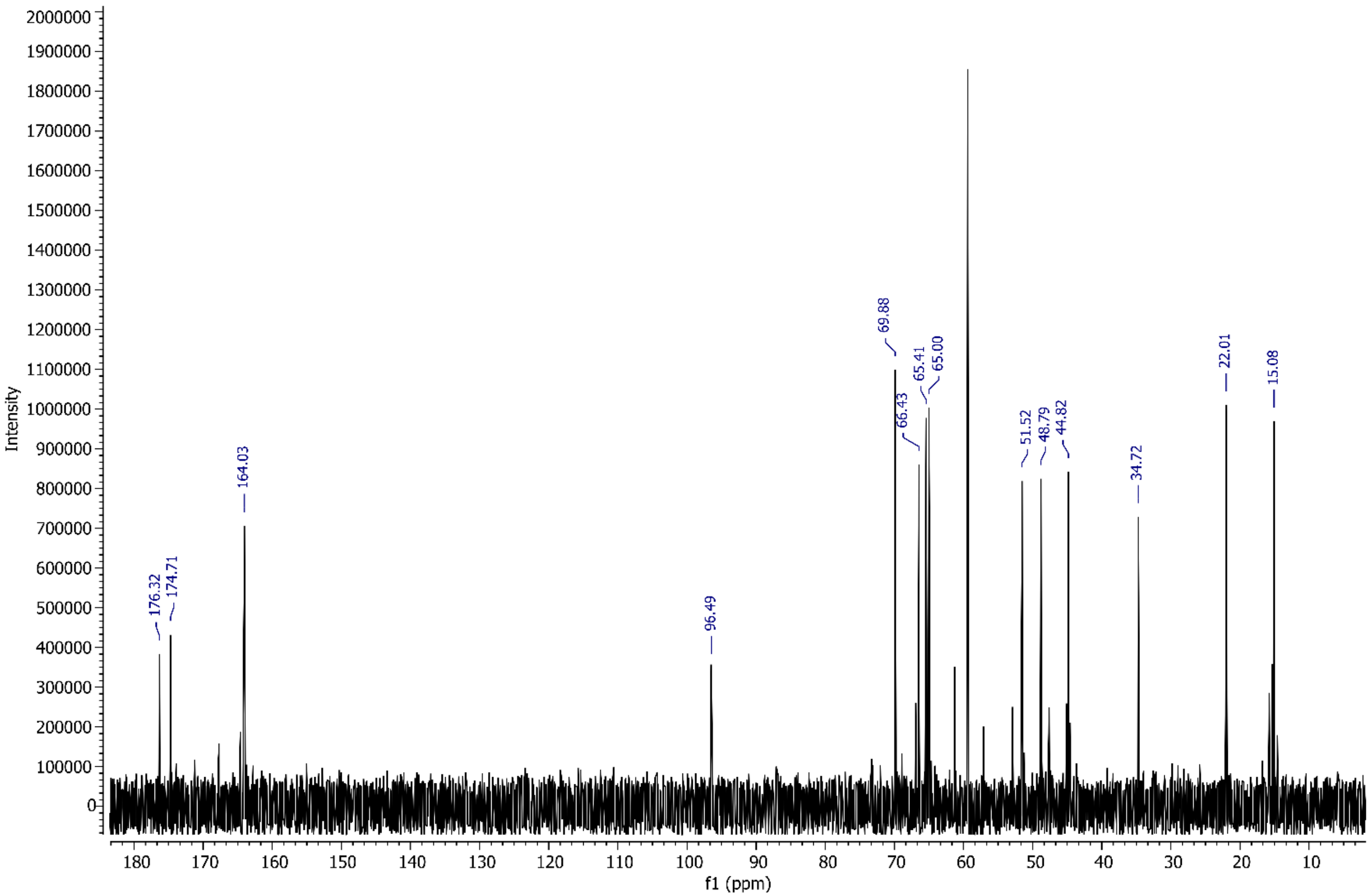

*Mass spectrum of Pse5Hb7Fm (**20**)*

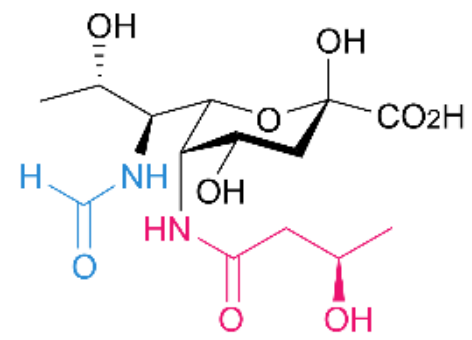

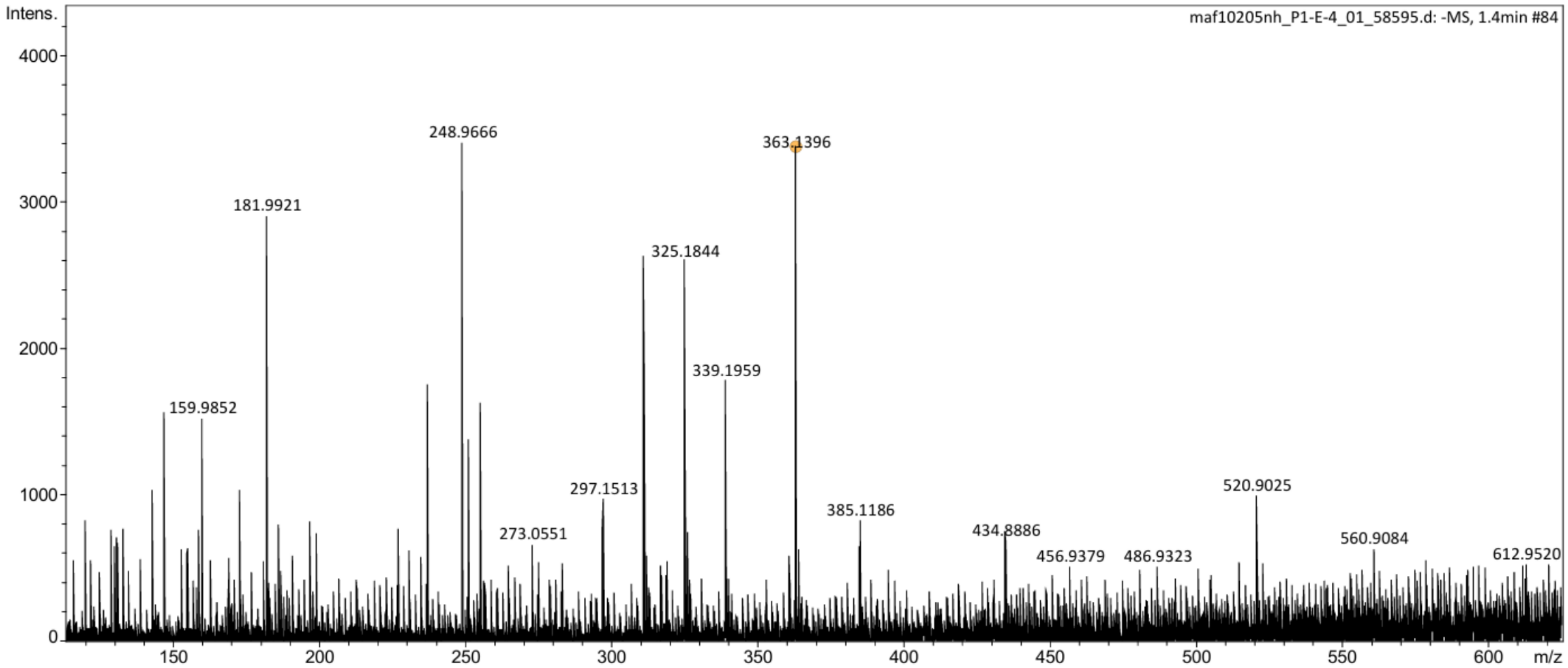

Meas. m/z	#	Ion Formula	m/z	err [ppm]	err [mDa]	mSigma	Mean err [ppm]
363.1396	1	C14H23N2O9	363.1409	3.5	1.3	22.5	3.9

Pse5Ac7Fm

*Hydrogen NMR spectrum of Pse5Ac7Fm (**21**) 700 MHz proton*

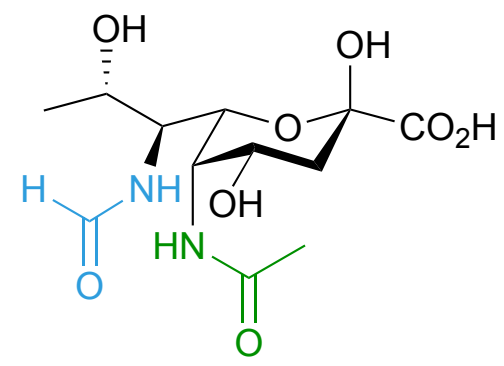

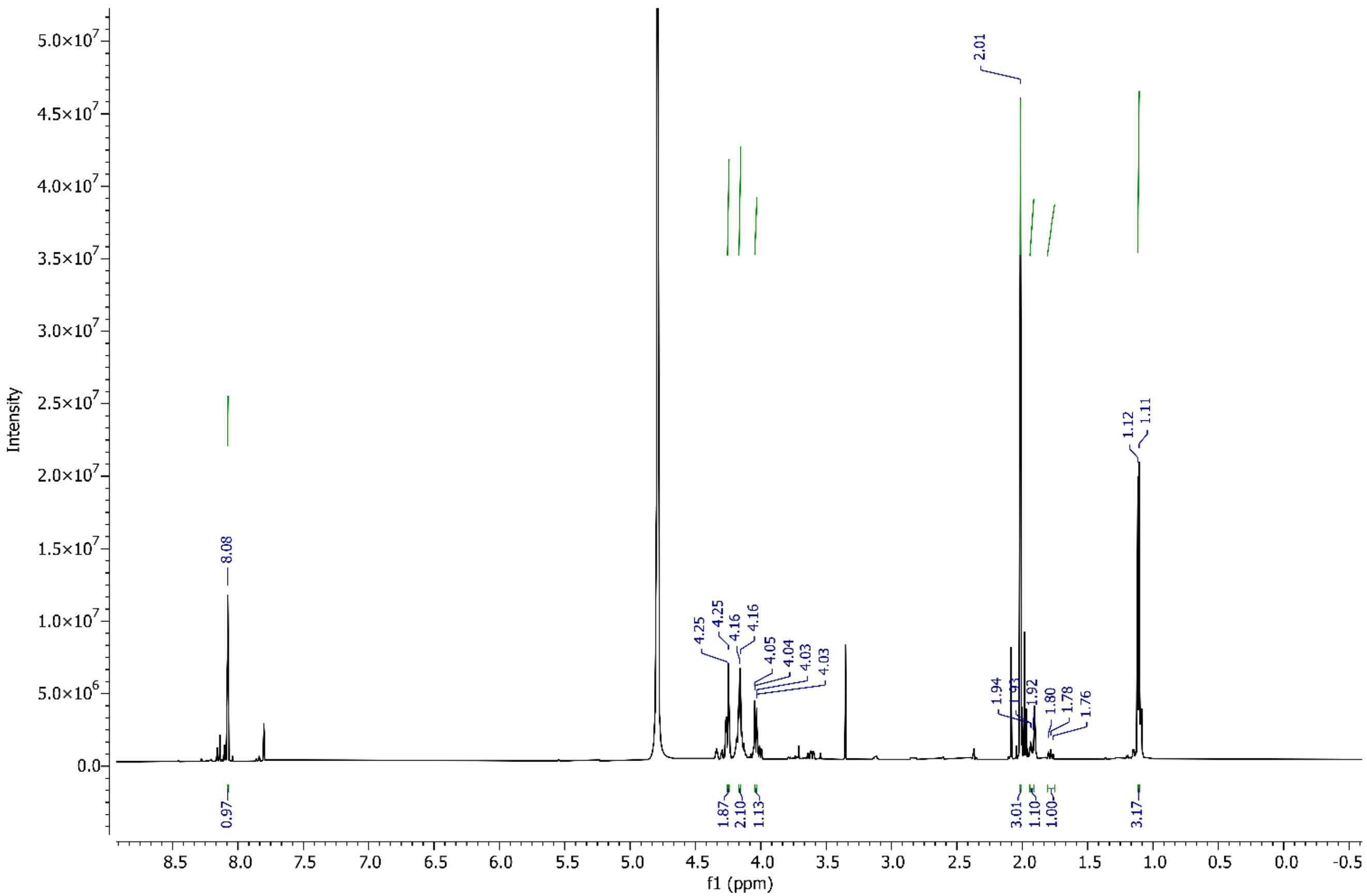

*Carbon NMR spectrum of Pse5Ac7Fm (**21**)*

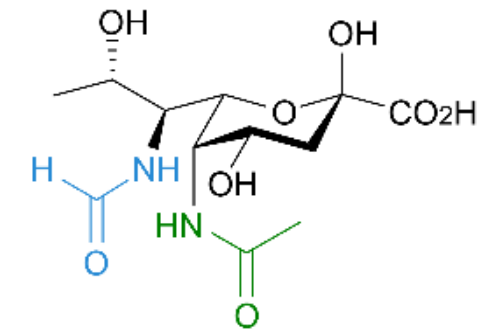

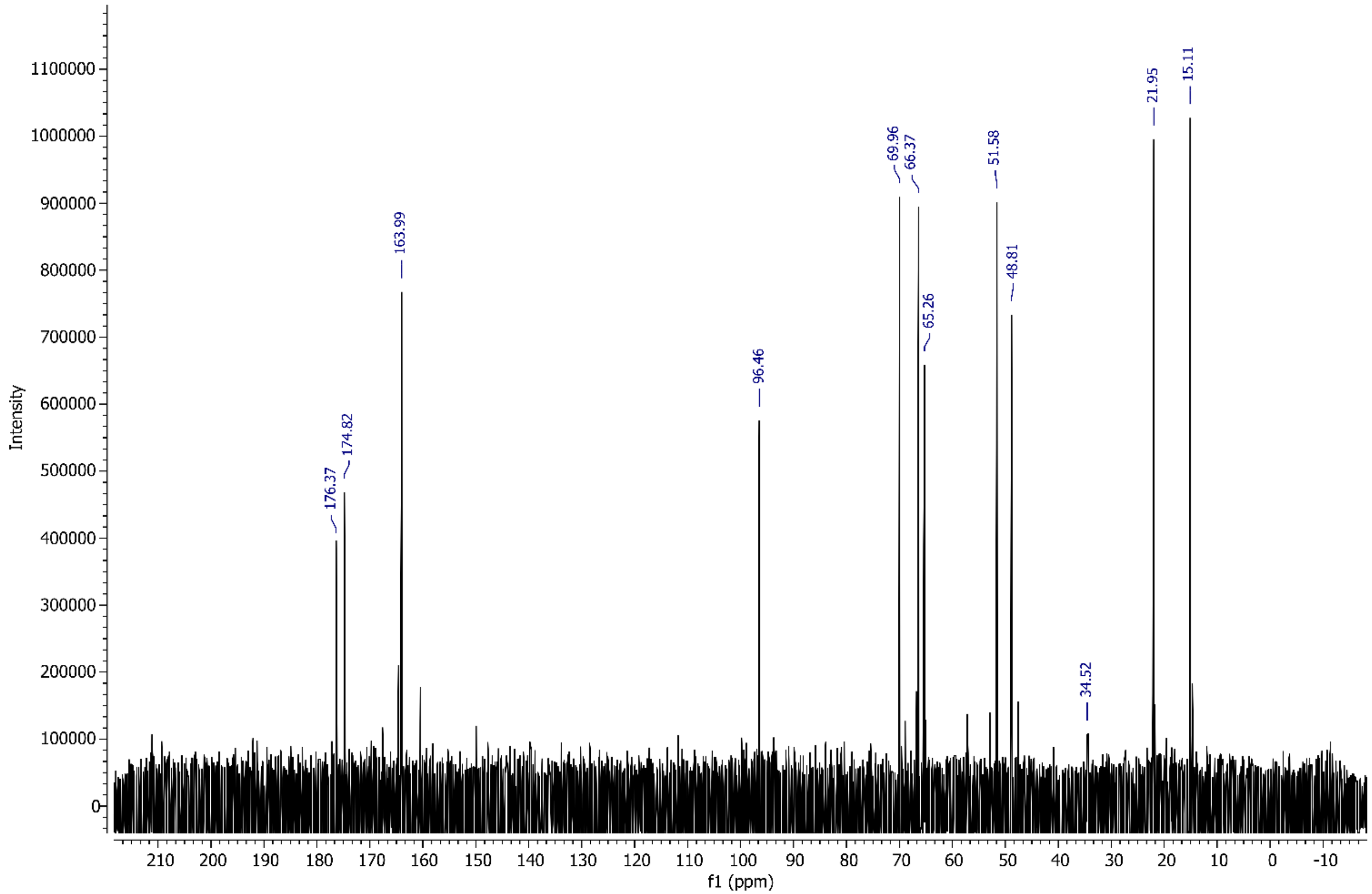

*HMQC spectrum of Pse5Ac7Fm (**21**)*

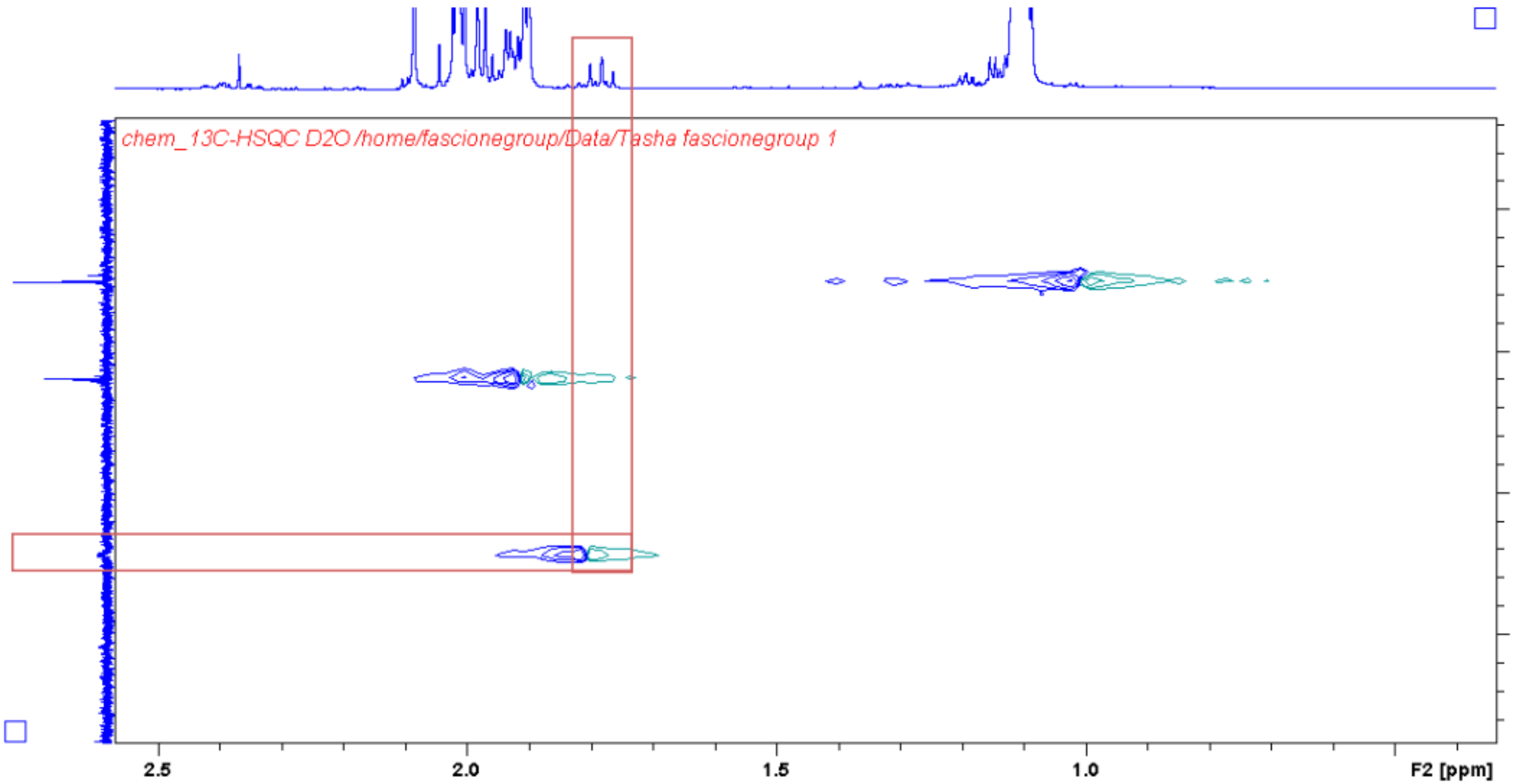

*Mass spectrum of Pse5Ac7Fm (**21**)*

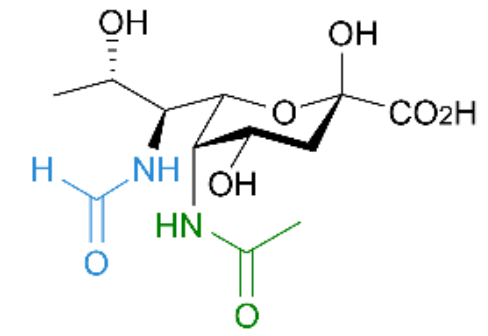

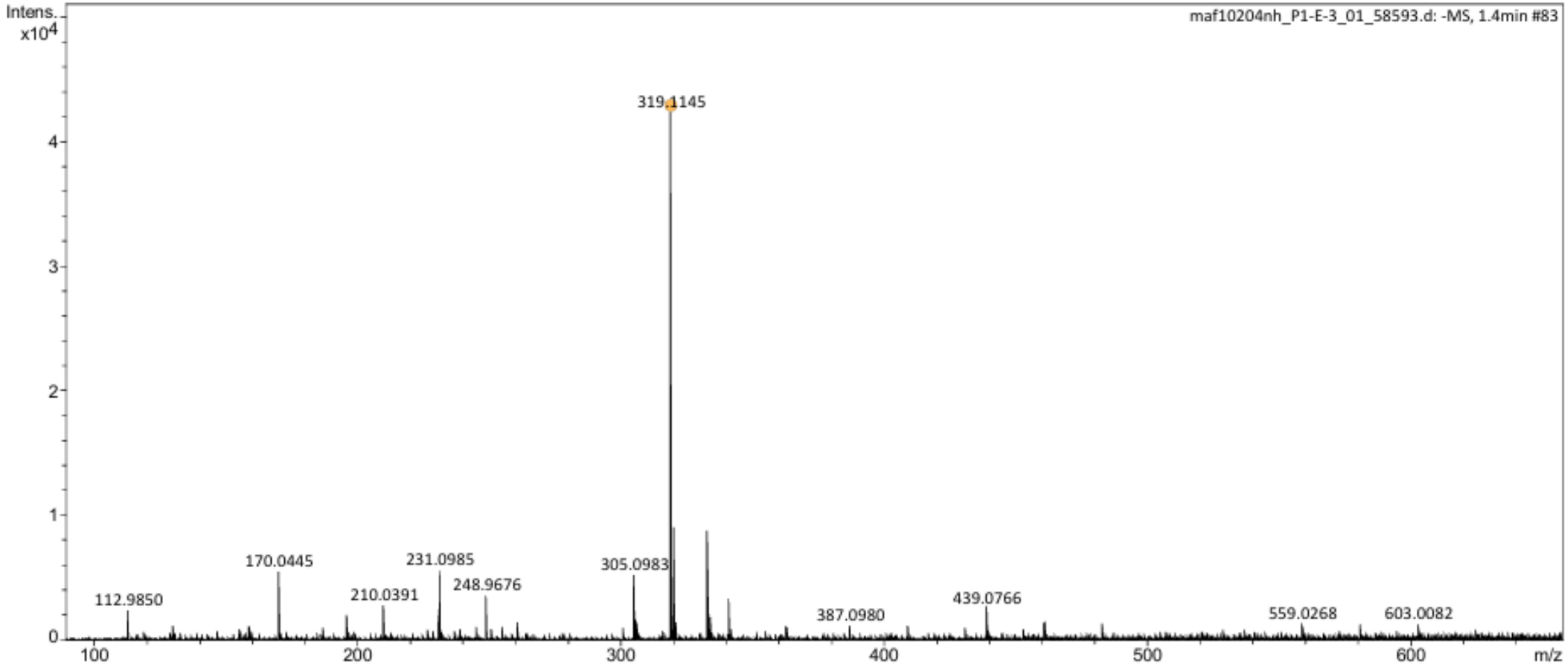

Meas. m/z	#	Ion Formula	m/z	err [ppm]	err [mDa]	mSigma	Mean err [ppm]
319.1145	1	C12H19N2O8	319.1147	0.4	0.1	41.0	-2.3

*IR spectrum of Pse5Ac7Fm (**21**)*

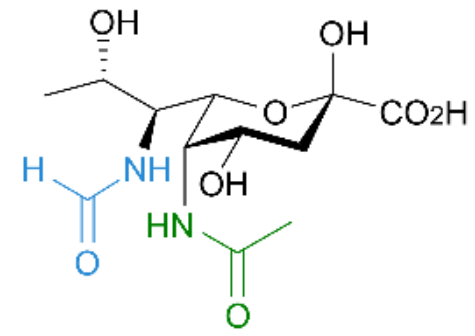

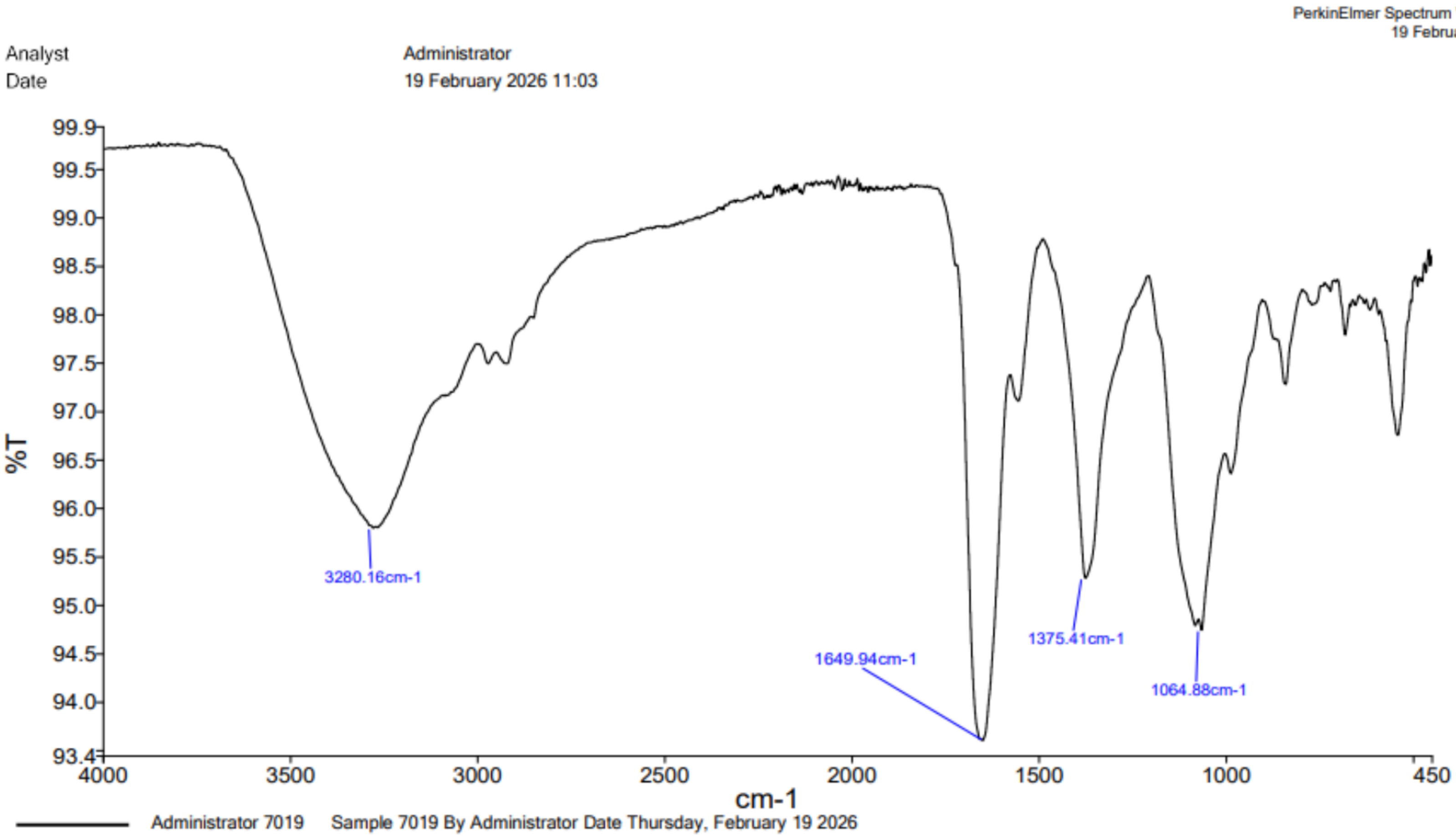

Pse5Ac7Hb

*Hydrogen NMR spectrum of Pse5Ac7Hb (**22**) 700 MHz*

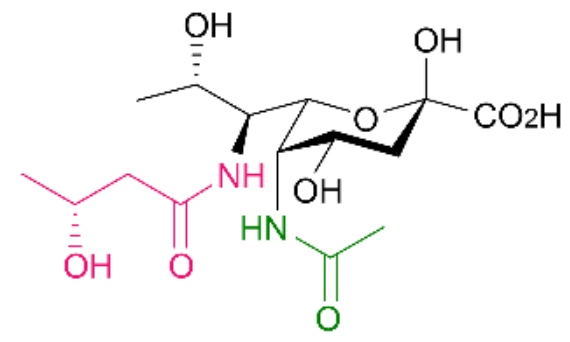

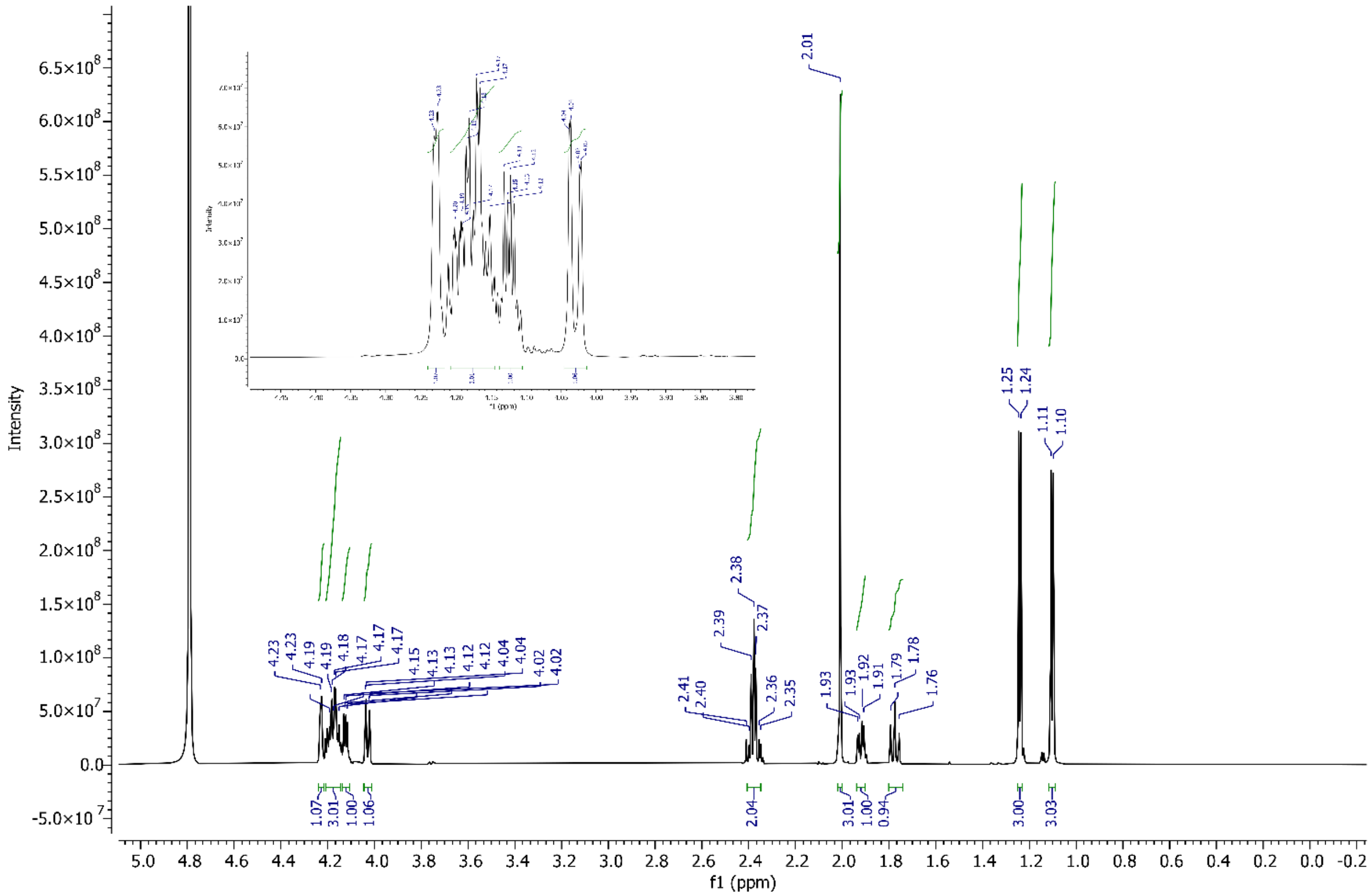

*Carbon NMR spectrum of Pse5Ac7Hb (**22**)*

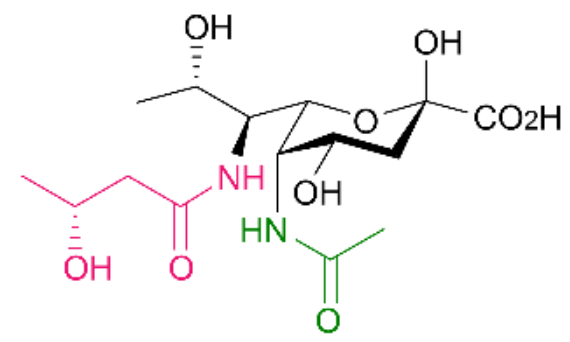

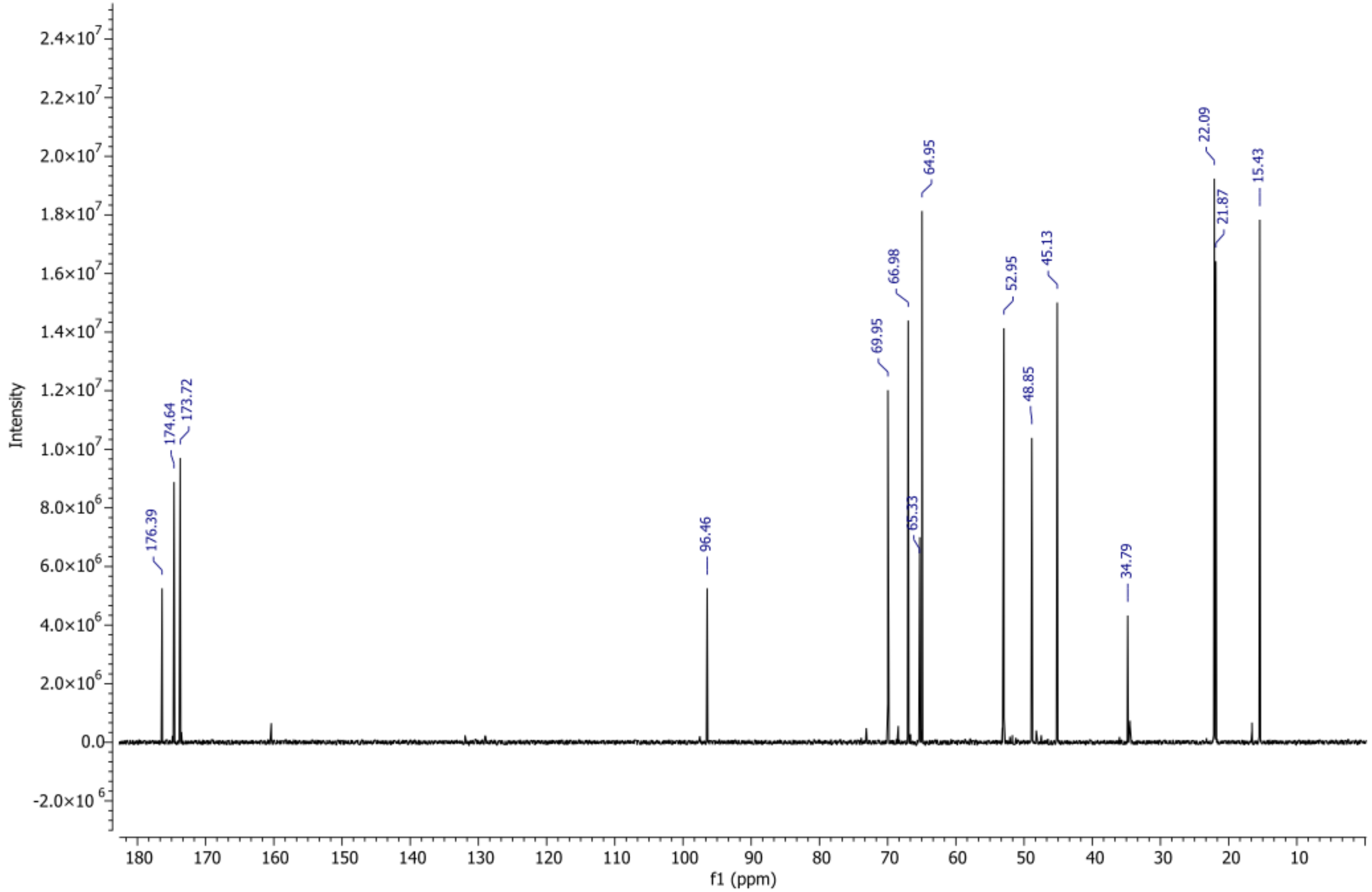

*Mass spectrum of Pse5Ac7Hb (**22**)*

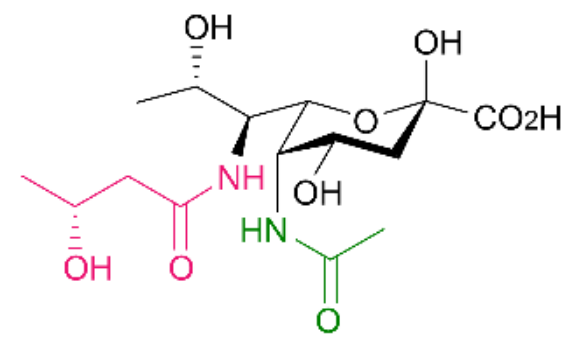

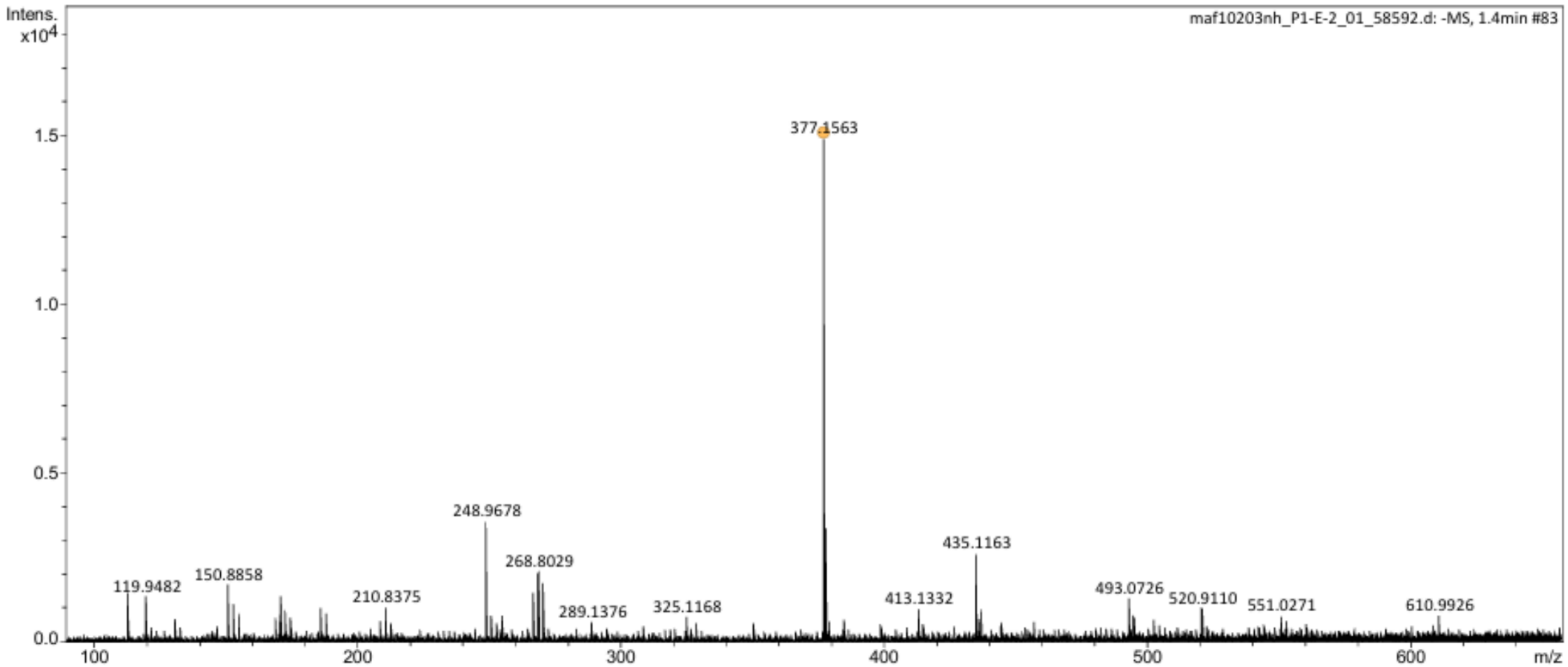

Meas. m/z	#	Ion Formula	m/z	err [ppm]	err [mDa]	mSigma	Mean err [ppm]
377.1563	1	C15H25N2O9	377.1566	0.7	0.3	34.8	-2.1

*IR spectrum of Pse5Ac7Hb (**22**)*

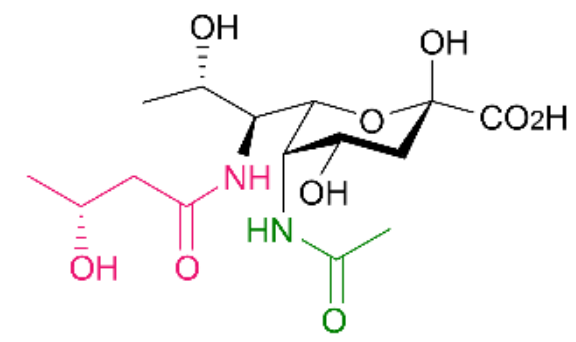

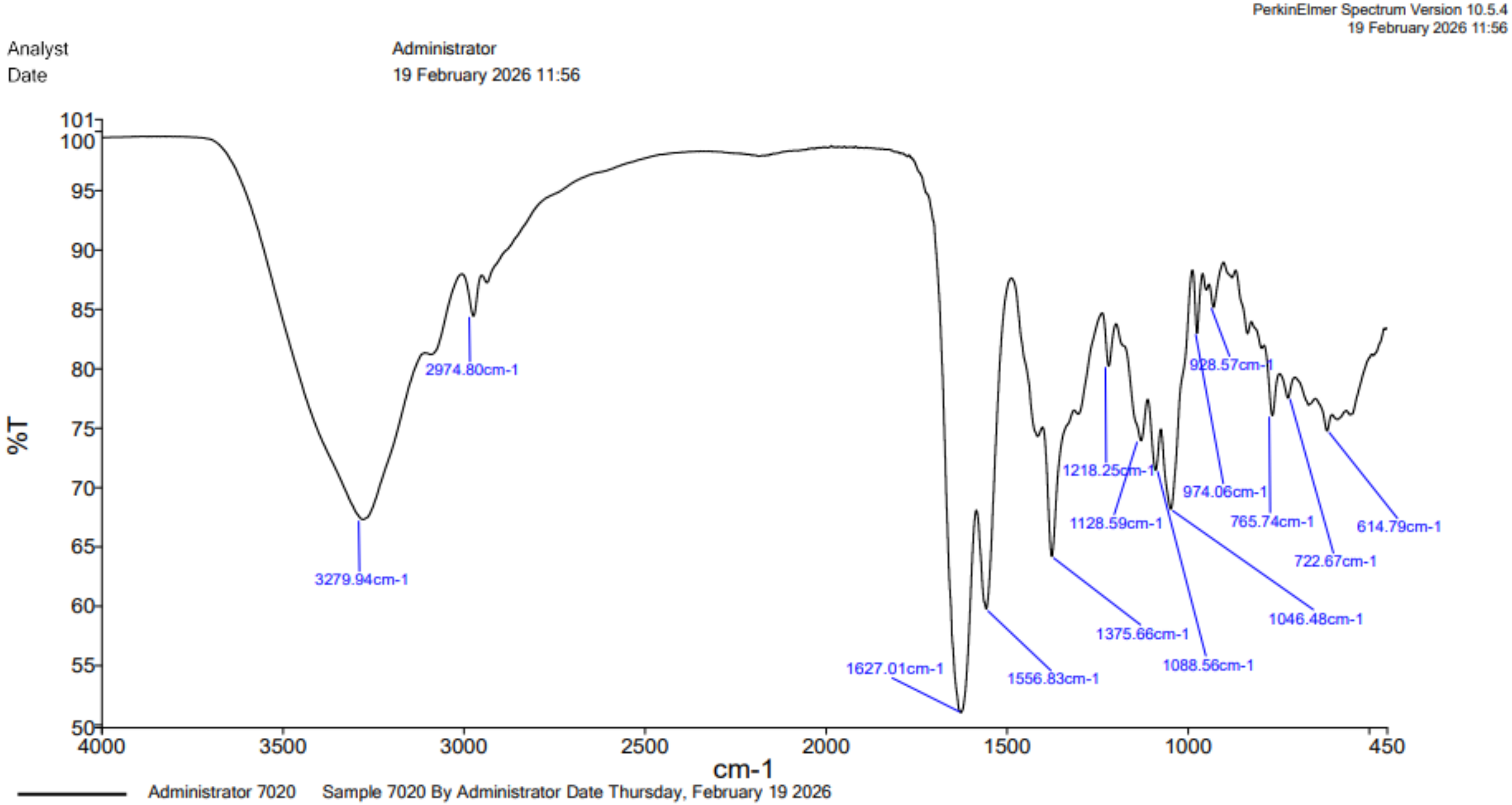

(1) Chidwick, H. S.; Flack, E. K. P.; Keenan, T.; Walton, J.; Thomas, G. H.; Fascione, M. A. Reconstitution and optimisation of the biosynthesis of bacterial sugar pseudaminic acid (Pse5Ac7Ac) enables preparative enzymatic synthesis of CMP-Pse5Ac7Ac. *Scientific Reports* **2021**, *11* (1), 4756. DOI: 10.1038/s41598-021-83707-x.
(2) Niedzwiecka, A.; Sequeira, C.; Zhang, P.; Ling, C.-C. An efficient and scalable synthesis of 2,4-di-N-acetyl-l-altrose (l-2,4-Alt-diNAc). *RSC Advances* **2021**, *11* (19), 11583–11594, 10.1039/D1RA01070K. DOI: 10.1039/D1RA01070K.
(3) Liu, H.; Zhang, Y.; Wei, R.; Andolina, G.; Li, X. Total Synthesis of Pseudomonas aeruginosa 1244 Pilin Glycan via de Novo Synthesis of Pseudaminic Acid. *Journal of the American Chemical Society* **2017**, *139* (38), 13420–13428. DOI: 10.1021/jacs.7b06055.
(4) Niedzwiecka, A.; Pham, Q.; Ling, C.-C. Regiospecific O → N Acyl Migration as a Methodology to Access l-Altropyranosides with an N2,N4-Differentiation. *Organic Letters* **2022**, *24* (47), 8667–8671. DOI: 10.1021/acs.orglett.2c03508.
(5) Lee, Y. J.; Kubota, A.; Ishiwata, A.; Ito, Y. Synthesis of pseudaminic acid, a unique nonulopyranoside derived from pathogenic bacteria through 6-deoxy-AltdiNAc. *Tetrahedron Letters* **2011**, *52* (3), 418–421. DOI: https://doi.org/10.1016/j.tetlet.2010.11.078.
